# Genome-wide analysis of *Tol2 *transposon reintegration in zebrafish

**DOI:** 10.1186/1471-2164-10-418

**Published:** 2009-09-08

**Authors:** Igor Kondrychyn, Marta Garcia-Lecea, Alexander Emelyanov, Sergey Parinov, Vladimir Korzh

**Affiliations:** 1 Cancer and Developmental Cell Biology Division, Institute of Molecular and Cell Biology, Singapore; 2 Department of Biological Sciences, National University of Singapore, Singapore; 3 Temasek Life Sciences Laboratory, Singapore

## Abstract

**Background:**

*Tol2*, a member of the hAT family of transposons, has become a useful tool for genetic manipulation of model animals, but information about its interactions with vertebrate genomes is still limited. Furthermore, published reports on *Tol2 *have mainly been based on random integration of the transposon system after co-injection of a plasmid DNA harboring the transposon and a transposase mRNA. It is important to understand how *Tol2 *would behave upon activation after integration into the genome.

**Results:**

We performed a large-scale enhancer trap (ET) screen and generated 338 insertions of the *Tol2 *transposon-based ET cassette into the zebrafish genome. These insertions were generated by remobilizing the transposon from two different donor sites in two transgenic lines. We found that 39% of *Tol2 *insertions occurred in transcription units, mostly into introns. Analysis of the transposon target sites revealed no strict specificity at the DNA sequence level. However, *Tol2 *was prone to target AT-rich regions with weak palindromic consensus sequences centered at the insertion site.

**Conclusion:**

Our systematic analysis of sequential remobilizations of the *Tol2 *transposon from two independent sites within a vertebrate genome has revealed properties such as a tendency to integrate into transcription units and into AT-rich palindrome-like sequences. This information will influence the development of various applications involving DNA transposons and *Tol2 *in particular.

## Background

The transposable element *Tol2 *from medaka fish is the first functional transposon identified in vertebrates [1]. It belongs to the hAT family (named for *hobo*, *Ac *and *Tam3*) and integrates into host DNA through a "cut-and-paste" mechanism [2]. Recently, a non-autonomous *Tol2*-based system has been developed as a tool for genome analysis of vertebrates and for highly efficient transgenesis [3-11]. It has been used for both gene trap and enhancer trap (ET) screens [12-14] as well as insertional mutagenesis [15,16]. Some of these applications have recently been reviewed [17,18].

One of the features of non-autonomous transposon-based systems, including *Tol2*, is that a transposon integrated into a genome can be remobilized if transposase mRNA is available. Previous applications of the transposon system have been based on random integration after co-injection of a plasmid DNA harboring *Tol2 *and transposase mRNA. Such random integration is attractive for a wide variety of applications ranging from gene discovery to gene therapy. However, the pattern of transposon integration upon remobilization from the donor site can be substantially different from that of plasmid-based integration. For example, the *Sleeping Beauty *(*SB*) transposon has a strong tendency to reinsert during *in vivo *remobilization at loci closely linked to its donor site [19]. Such local hopping could be favorable not only for region-specific mutagenesis [20] but also for region-specific probing of enhancers. However, despite the recent surge of interest in the *Tol2 *transposon system, its integration and/or reintegration properties have not yet been analyzed in detail.

Using *Tol2*, we have previously established a collection of stable transgenic zebrafish ET lines and demonstrated that a single copy of a *Tol2 *transposon-based ET cassette can be remobilized into a new chromosomal location [13,21]. Here, we report the results of a genome-wide analysis of *Tol2 *reintegration in zebrafish initiated from two genomic sites in two different chromosomes.

## Results

### Design of the *Tol2 *transposon remobilization screen

**Figure 1:**
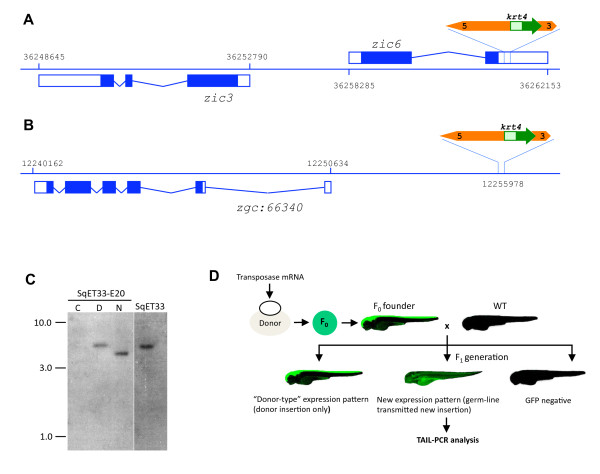
**Donor sites used for the remobilization screen**. **A**: The SqET33 donor site (*Tol2 *transposon insertion in the 3' UTR of *zic6*). **B**: The SqET33-E20 donor site (*Tol2 *transposon insertion 4.2 kb upstream of *zgc:66340*). The green arrow shows *EGFP*; *krt4 *is a minimal promoter; orange arrows represent the 5' and 3' ends of *Tol2*; coding exons are depicted as blue boxes; and UTRs are represented as open boxes. **C**: SqET33-E20 F_0 _founder fish was outcrossed to wild type and DNA isolated from GFP-negative embryos (negative control, C), GFP-positive embryos showing a SqET33 expression pattern of GFP (donor-type, D), and GFP-positive embryos showing a new expression pattern distinct from the donor one (N), were used for Southern blot hybridization. SqET33, DNA from the donor line. **D**: A scheme of the *Tol2 *remobilization screen.

We used two different ET lines as donors for the remobilization experiments. The first line, SqET33, was established during our pilot enhancer trap (ET) screen [13]. It carries a single insertion of a transposable element in the 3' UTR of a novel gene of the Zic family, *zic6 *on chromosome 14 (Figure 1A). The second line, SqET33-E20, was established after induced remobilization of a *Tol2 *transposon-based ET cassette from the SqET33 line [22]. It carries a single insertion located approximately 4.2 kb upstream of a putative gene *zgc:66340 *(similar to Axin-1 up-regulated gene 1) on chromosome 24 (Figure 1B). In both lines, stable tissue-specific GFP expression is maintained through at least four generations of breeding, indicating that it is not affected by silencing or epigenetic modification. For both donor lines we confirmed the presence of a single *Tol2 *transposon insertion by Southern blot hybridization (Figure 1C).

In order to induce remobilization of the *Tol2 *transposon-based ET cassette from the donor site in SqET33 (referred to as the SqET33 donor site), we initially used mRNA containing only an open-reading frame (ORF) for Tol2 transposase. In this experiment, transgenic fish homozygous for a single *Tol2 *insertion were outcrossed to wild type fish. The embryos from this cross (F_0 _generation) were injected with transposase mRNA at the one- or two-cell stage. Most of the injected embryos only showed a "donor-type" GFP expression pattern. However, some showed mosaic expression of GFP in somatic cells, mostly in muscle or skin [13]. We did not preselect embryos on the basis of such GFP expression, but used these observations as an indication that a transposition had been triggered. Because we were only interested in heritable *Tol2 *re-transpositions, the F_0 _generation was not analyzed in respect of transposon integration into somatic cells. All injected embryos were raised to sexual maturity and crossed to wild type fish, and their progeny (F_1 _generation) were analyzed for changes in GFP expression. We identified injected fish as founders (F_0 _founder) if their progeny showed a new GFP expression pattern that differed from the donor pattern. The F_0 _founder fish carried the *Tol2 *reintegrations in the germline. In our screening scheme, only the F_1 _embryos that showed new GFP expression patterns were further analyzed by TAIL-PCR (Figure 1D). Using this strategy, we initially identified 21 F_0 _founder fish out of 282 injected fish. This corresponded to a 7% apparent germline transposition rate (1^st ^screen in Table 1). We assumed that the transposase mRNA used at that point was not very effective in *Tol2 *remobilization. Therefore, in subsequent experiments, we used a modified transposase mRNA containing the 5' and 3' UTRs of the *Xenopus β-globin *gene [6]. As a consequence, we identified 103 F_0 _founder fish out of 268 injected fish in a new round of screening, a much higher apparent germline transposition rate (38%, 2^nd ^screen in Table 1).

**Table 1:** *Tol2 *remobilization screen overview

**Screen**	**1st^a^**	**2nd^b^**	**3rd^c^**
Total F_0 _fish screened	282	268	175
Total F_0 _founder fish^d^	21	103	84
Apparent germline transposition rate^e^	7%	38%	48%
Number of new insertions^f^	23	172	143

^a ^The SqET33 line was used as a donor and mRNA containing only an ORF for the transposase was injected into embryos. ^b ^The SqET33 line was used as a donor and transposase mRNA containing the 5'- and 3'-UTRs of the *Xenopus β-globin *gene was injected into embryos. ^c ^The SqET33-E20 line, a derivative of SqET33, was used as a donor and transposase mRNA containing the 5'- and 3'-UTRs of the *Xenopus β-globin *gene was injected into embryos. ^d ^Fish with transposon reintegration into germline. The progeny (F_1_) of these fish showed new GFP expression patterns different from the original donor one. ^e ^Rates are calculated as the percentage of F_0 _founder fish. Since we counted only F_0 _fish producing progeny with new GFP expression patterns, these rates are likely to be underestimates. ^f ^Some F_0 _founder fish have more than one insertion in the germline identified by TAIL-PCR.

To test whether the donor site influences transposon remobilization, we used the SqET33-E20 line (referred to as the SqET33-E20 donor site) as a donor. We identified 84 F_0 _founder fish out of 175 injected fish, with an apparent germline transposition rate similar to that of the 2^nd ^screen (48%, 3^rd ^screen in Table 1). Because our screening was based on the appearance of new GFP expression patterns, new insertions that caused no changes in that pattern were not taken into account. Therefore, the actual germline transposition rate for both donor sites should be higher.

In most cases, when F_0 _fish (heterozygous for *gfp*) were outcrossed to wild type fish, GFP-negative and -positive embryos (regardless of their expression patterns) were segregated in an approximately 1:1 ratio. However, we observed altered GFP segregation ratio following remobilization of the *Tol2 *transposon (see Additional file 1). Out of 725 injected F_0 _fish (from three rounds of screening), 22 produced more than 50% GFP-positive progeny, suggesting an increase of *Tol2 *copy number. Thirty-seven injected F_0 _fish produced less than 50% GFP-positive progeny, indicating either partial loss of *Tol2 *or silencing of *gfp*. A similar alteration of GFP segregation has recently been described for re-transposition of the *Ds *element in zebrafish [23]. Germinal excision without concomitant transposon reintegration has also been reported for the *Ac/Ds *transposon in plants [24,25].

Interestingly, about 40% of F_0 _founder fish after outcrossing with wild type produced progeny (F_1_) such that individual embryos within a single F_1 _family showed distinct new GFP expression patterns. The number of these new patterns per single F_1 _family varied from two to seven, suggesting multiple transposon integration events in the germline of a single F_0 _fish (Table 2). In most cases, TAIL-PCR analysis of individual embryos from such F_1 _families demonstrated single *Tol2 *insertions at different positions in the genome. The presence of a single insertion in F_1 _fish suggests that transposition occurred independently in separate germline cells, and *Tol2 *was transposed by a non-replicative mechanism, since an F_0 _fish is heterozygous for a single insert. However, in a few cases, TAIL-PCR detected two or three insertions in one F_1 _embryo; this was additionally confirmed by Southern blot hybridization (see Additional file 2).

**Table 2:** Number of *Tol2 *insertions per F_0 _germline

**Number of insertions/F_0 _germline^a^**	**Number of F_0 _founder fish (n = 208)**
1	122 (59%)
2	48 (23%)
3	27 (13%)
4	8 (4%)
5	2 (1%)
6	0
7	1 (0.5%)

Donor insertion^b^	36 (17%)

^a ^F_0 _founder fish were outcrossed to wild type fish and the resulting embryos showing new GFP expression patterns were analyzed by TAIL-PCR. ^b ^The donor insertion retained after reintegration was confirmed by TAIL-PCR. These are cumulative data from three rounds of screening.

About 20% of F_0 _founder fish produced embryos with the donor-type GFP expression pattern as well as the new GFP expression pattern in the same embryo. In such embryos, donor insertion was always detected by TAIL-PCR, indicating that the donor copy was retained after retransposition. This result suggests that in some cases remobilization probably occurs during DNA replication. Transposition during DNA replication has been well described for other "cut-and-paste" transposons, particularly for *Ac/Ds *[26].

### *Tol2 *preferentially reintegrates into linked loci

**Figure 2:**
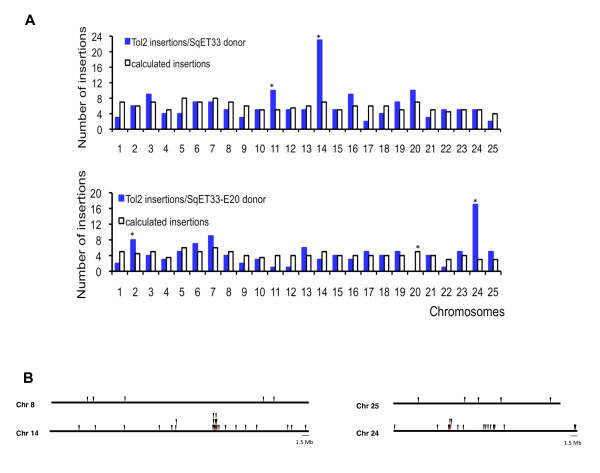
**Genome-wide distribution pattern of *Tol2 *integrations**. **A**: Chromosomal distribution pattern of *Tol2 *insertions depending on the donor site. Asterisks show statistically significant differences (p < 0.0001 for the donor chromosomes and p < 0.05 for the others). **B**: Pattern of *Tol2 *reintegrations linked to the different donor sites (chromosomes 14 and 24). Non-donor chromosomes 8 and 25, comparable in size to the donor chromosomes, are shown for reference. Red triangles represent the donor sites.

By analyzing the chromosomal distribution pattern of the new insertions (see Materials and methods), we found that all chromosomes were hit by *Tol2 *during re-transposition (Figure 2A and Table 3). About 15% of reintegration events occurred in the donor chromosomes. Such a linked pattern of re-transposition was found for both donor sites: 23 out of 153 insertions were mapped on chromosome 14 (re-transposition from the SqET33 donor site), while 17 out of 111 were mapped on chromosome 24 (re-transposition from the SqET33-E20 donor site). We also found that about 43% (10/23) and 24% (4/17) of reintegrations occurred less than 1 Mb from the SqET33 and SqET33-E20 donor sites, respectively (Figure 2B). However, these numbers are probably underestimates, since only transpositions that cause changes in GFP expression patterns were considered.

**Table 3:** Distribution of *Tol2 *insertions on chromosomes

	**SqET33 donor line (n = 153)**			**SqET33-E20 donor line (n = 111)**		
	
**Chromosome**	**Number of insertions**	***P*-value**	**Expected number of insertions**	**Number of insertions**	***P*-value**	**Expected number of insertions**
1	3	0.122	7	2	0.17	5
2	6	1.0	6	8	0.042	4
3	9	0.44	7	4	0.648	5
4	4	0.65	5	3	0.611	4
5	4	0.147	8	5	0.675	6
6	7	1.0	7	7	0.361	5
7	7	0.717	8	9	0.209	6
8	5	0.44	7	4	0.648	5
9	3	0.212	6	2	0.309	4
10	5	1.0	5	3	0.611	4
11	10	0.023	5	1	0.127	4
12	5	0.678	6	1	0.127	4
13	5	0.678	6	6	0.309	4
14	23	<0.0001	5	3	0.361	5
15	5	1.0	5	4	1.0	4
16	9	0.212	6	3	0.611	4
17	2	0.096	6	5	0.611	4
18	4	0.406	6	4	1.0	4
19	7	0.364	5	5	0.611	4
20	10	0.247	7	0	0.022	5
21	3	0.364	5	4	1.0	4
22	5	1.0	5	1	0.242	3
23	5	1.0	5	5	0.611	4
24	5	1.0	5	17	<0.0001	3
25	2	0.312	4	5	0.242	3
Un	15	ND	ND	8	ND	ND

ND, not determined; Un, contig not assigned to any chromosome; *P*-value was calculated by using a χ^2 ^test; n, number of mapped insertions.

**Figure 3:**
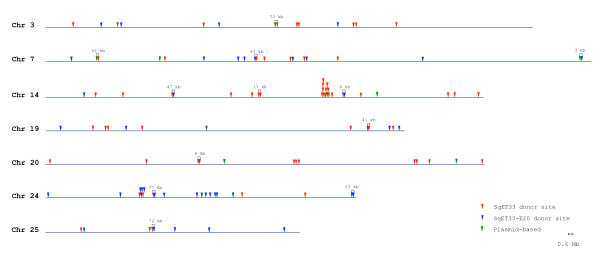
**Clustering of *Tol2 *integrations in the zebrafish genome**. Only chromosomes in which two independent *Tol2 *insertions are mapped within less than 100-kb of one another are shown. Data for the plasmid-based derived integrations were taken from [13] and remapped.

We noticed that some chromosomes were possibly favored targets for *Tol2 *integration, while others appeared to be disfavored. However, with the exception of the donor chromosomes, only chromosome 11 (when the SqET33 donor site was used for the re-transposition) and chromosome 2 (when the SqET33-E20 donor site was used) were somewhat preferred targets. Chromosome 20 was found to be a potential cold spot for *Tol2 *integration (when the SqET33-E20 donor site was used for the re-transposition). However, hotspots cannot be adequately evaluated at the chromosomal level at a resolution of 338 events. In addition, when all *Tol2 *integrations were placed on the zebrafish genome map, there was no indication of significant clustering (except on the donor chromosomes). In some cases, two independent transposon insertions were mapped within 3 to 77 kb of one another (Figure 3).

### *Tol2 *integration into transcription units

**Figure 4:**
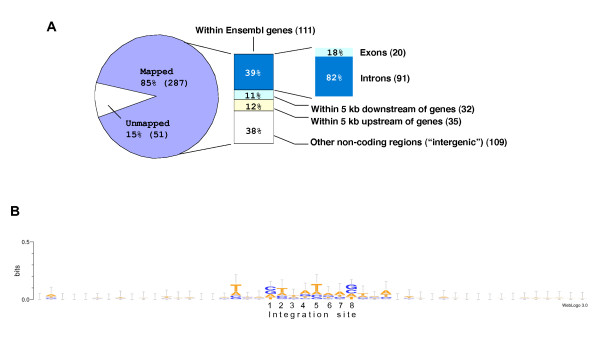
***Tol2 *integration properties**. **A: **Distribution of *Tol2 *insertions in respect of endogenous genes. **B: ***Tol2 *integration site motif analysis. A graphical representation of a nucleic acid multiple sequence alignment (n = 368) was generated by WebLog (version 3.0).

Sequencing of the PCR-amplified regions that flank the target sites from all founder fish confirmed the generation of novel integration events that were not present in the original donor lines (see Additional files 3 and 4). During the three rounds of screening, we isolated 338 transposon integration sites. The genomic positions of 287 of these sites were identified using BLASTN in the Ensembl genome browser (Figure 4A; see Materials and methods for details). However, we could not unambiguously map the remaining 51 integration sites because (i) multiple hits were found that were either very similar or identical to the genomic sequence (26 integration sites), (ii) integrations occurred in repetitive dinucleotide sequences (eight integration sites) or (iii) there were sequence gaps in the database (17 sites). Approximately 39% of the mapped integration sites were found within known or predicted genes annotated in the zebrafish genome. About 82% of these sites were mapped within introns and about 18% within exons. There was a bias towards introns because their cumulative size is much larger than that of exons, so they present a much larger target for transposon integration. Assuming a 5-kb interval as an arbitrary threshold for the regulatory regions at the 5' and 3' ends of genes, the frequency of integration was about 62% for known or predicted transcription units. Furthermore, about 59% (64/109) of the "intergenic" insertions were mapped within 50-kb regions upstream or downstream of known or predicted genes annotated in the zebrafish genome. We also compared the distribution pattern of insertions depending on donor site. No significant difference was found (Table 4).

**Table 4:** Distribution of *Tol2 *insertions depending on donor site

**Donor site^a^**	**SqET33 donor**		**SqET33-E20 donor**	
	
	**Number**	**Frequency^b^**	**Number**	**Frequency^b^**
Insertions in introns	50	30%	41	34%
Insertions in exons	15	9%	5	4%
Insertions in 5' end^c^	20	12%	15	12%
Insertions in 3' end^c^	22	13%	10	8%
Intergenic insertions^d^	59	36%	50	41%
Mapped insertions	166		121	

^a ^For SqET33, donor site data from the 1st and 2nd screens were combined. ^b ^Frequencies are calculated as the percentages of particular insertions. ^c ^We set a 5-kb interval as an arbitrary threshold for a regulatory region at the 5' and 3' ends of a transcription unit. ^d ^More than 5 kb upstream or downstream of a transcription unit. Insertions with no annotated tagged gene were also considered "intergenic".

**Table 5:** *Tol2 *integrations into genomic repetitive elements

**Repetitive elements**	**Number of insertions**
DNA transposon	48
Dr element	47
non-LTR retrotransposon^a^	14
LTR retrotransposon	3
Tandem repeat^b^	16
All genomic repeats	128 (38%)
Sequenced insertions	338

^a ^Include LINE, long interspersed nuclear element; SINE, short interspersed nuclear element. ^b ^Include microsatellites and minisatellites. These are cumulative data from three rounds of screening.

We found five genes (*ENSDARG00000033473*, *ENSDARG00000034820*, *zgc:110750*, *foxa *and *fgf13*) that were recurrently hit by *Tol2*. Two insertions in *ENSDARG00000033473 *(334 kb) were mapped in one intron with a distance of 41 kb between the integration sites. Similarly, two insertions in *zgc:110750 *(196 kb) were mapped in one intron with a distance of 6 kb between the integration sites. Two insertions in *ENSDARG00000034820 *(67 kb) were mapped in an intron and 4 kb downstream of the gene, while insertions in *fgf13 *(228 kb) were mapped in two different introns. Finally, the two insertions in *foxa *(5 kb) were mapped 3 kb upstream and 1 kb downstream from the gene. Four of these five genes (*fgf13 *was the exception) were hit from both independent donor sites. Since the sizes of the targeted genes differ substantially (from 5 kb to 334 kb), the distance between two independently integrated *Tol2 *transposons is more important (in these cases, the distance ranges from 6 kb to 47 kb). We calculated the probability of hitting a similarly-sized locus in the zebrafish genome and then used a binomial distribution test to determine the statistical significance of each targeted locus being hit twice. The *P *values ranged from 3.3 × 10^-6 ^to 2.8 × 10^-3^, probably indicating potential hotspots within these genes (with an exception of *fgf13*, since it is linked to the donor site).

### *Tol2 *integration into endogenous repeat elements

We analyzed the distribution pattern of *Tol2 *insertions with respect to various genomic repeat elements (Table 5); 128 out of the 338 integration sites were found in endogenous repeat elements that are currently annotated in the zebrafish genome. Most of these targeted repeat elements belong to DNA transposons and to unclassified Dr repeats. In addition, our results showed that *Tol2 *was less prone to integrate into retrotransposons (17 integration events) and tandem repeats (16 integration events). Interestingly, LTR-containing retrotransposons were very seldom targeted by *Tol2 *(only 3 integration events). According to Repbase Update [27], 357 different families of DNA transposons and 279 of retrotransposons are currently annotated in the zebrafish genome. Retrotransposons that contain LTRs are more diversely represented in the zebrafish genome than those that do not (222 *vs*. 57 families). The differences in targeting of repetitive elements observed in our experiment could be explained by a difference in either the copy number or the global distribution pattern of each class of repetitive elements in the zebrafish genome.

### Specificity of *Tol2 *integration site

Some transposable elements exhibit a high degree of integration specificity, while others display relatively little preference for a target DNA sequence [28,29]. We analyzed the nucleotide composition over a 48-bp sequence region comprising an 8-bp target site and 20-bp flanking sequences on each side according to [30] (Figure 4B). In addition to the 329 integration sites isolated in this study, we also included 39 integration sites isolated from our previous ET screen [13]. Comparisons among all the *Tol2 *integration sites and flanking DNA revealed no conserved pattern in the sequences flanking the eight base pair duplication at the integration site. The only exceptions were the nucleotides at ± 3 and ± 1 bp relative to the integration site, which were 46% and 40% conserved, respectively (see Additional file 5). In addition, we detected a weak AT-rich consensus that contained a palindrome-like core sequence TNA(**C**/**G**)**TTATAA**(**G**/**C**)TNA centered at the insertion site (shown in bold). However, only four integration sites were actual palindromes.

## Discussion

In this study, we analyzed a genome-wide reintegration of the non-autonomous transposable element *Tol2 *in zebrafish when it was remobilized from two different donor sites. We showed that the genomic *Tol2 *copy can be remobilized upon injection of transposase mRNA into the germlines of up to 48% of founders. Since we selected only those *Tol2 *reintegrations in germlines that caused changes in GFP expression, we in fact measured the "apparent transposition rate"; the actual germline transposition frequency during *in vivo *remobilization would be higher.

We analyzed *Tol2 *integration sites with respect of their chromosomal distribution, integration into intragenic regions and insertion site sequence specificity. Although novel integration sites were found on different chromosomes, *Tol2 *reintegration was not random. Almost 39% of transposon integrations were found within known or predicted genes. Most of them were found within introns, as expected in view of the high intron/exon ratio in the zebrafish genome. If we consider insertions into the regulatory regions adjacent to transcriptional initiation and termination sites, the rate of *Tol2 *transposition into genes was even higher. Since we used the TAIL-PCR method to isolate transposon inserts, we could not recover all possible *Tol2 *insertions in the genome. However, despite of using enhancer trap approach, the frequency with which *Tol2 *was integrated within intragenic regions was similar to that found for the *SB *transposon in human (39%) and mouse (31%) cells [31] and for *Ac*/*Ds *in rice (30%) [32-34] and *Arabidopsis *(38%) [35]. Therefore, *Tol2 *is as prone to integrate into transcriptional units as other DNA transposons.

Our results further demonstrated that about 15% of *Tol2 *reintegrations from a specific donor site were linked to the same chromosome. Such behavior was also noticed in [11], where about 18% of the mapped integration sites (6/34) were located on the donor chromosome. We found that about one third of intrachromosomal reintegrations were located within 1 Mb of the donor site. However, since we selected reintegrations on the basis of new GFP expression patterns, this number is likely to be lower than the actual number of such transpositions (for example, closely-linked transpositions may retain the GFP expression pattern of the donor). This local hopping phenomenon has been described for other DNA transposons [19,36-40]. For example, *SB *is mostly re-integrated within 3 Mb of the donor site [19] and the local hopping interval of the *P *element is within 100 kb [36]. Local hopping was also found for the hAT family. In this case, more than half of *Ac *transposon reintegrations occurred within 1.7 Mb of the donor site [37]. Overall, a linked reintegration property of the *Tol2 *system might be beneficial for setting a region-specific saturation ET screen.

Interestingly, our analysis revealed that some chromosomes other than the donor were somewhat preferred targets for *Tol2 *integration; still others appeared to be disfavored. Such transposon behavior may reflect the spatial chromosomal architecture within the nucleus, if multiple non-adjacent chromosome segments are closely juxtaposed at the nuclear interior or periphery [41-43]. There are many examples of correlations among the intranuclear positions of genes, their clusters and genetic activities, whereas the relative positioning of chromosomes seems to be maintained (reviewed in [44]). Therefore, such a property of transposons may potentially be used for analyzing the spatial organization of the genome.

We found that *Tol2 *differentially targeted the different classes of endogenous repetitive elements. For example, it more frequently targeted DNA transposons than retrotransposons and tandem repeats. The latter tendency contrasts with the profound preference of *Tc1/mariner *transposons for TA-containing microsatellite DNA [31,45]. Expansion of such repeats during replication slippage can cause repeat instability and increased recombination rates (reviewed in [46]), suggesting that these transposons may use the recombination machinery during integration. Like the *SB *transposon, *Tol2 *also avoided retrotransposons containing LTRs. The differences in targeting of endogenous repetitive elements may reflect differences in the copy numbers of each class of repeats, as well as differences among the mechanisms of integration utilized by each transposon family.

We also found that *Tol2 *was prone to integrate into AT-rich DNA regions and that a target site contained a weak palindrome-like consensus sequence. An AT-rich palindromic consensus has previously been found in the target sequence of *Tc1/mariner *transposons such as *SB *in human and mouse cells [31] and the *Tc1 *element in worms [47]. However, in contrast to the strict preference of *SB *and *Tc1 *for TA dinucleotide targets, *Tol2 *has no such preference at the nucleotide level. There is some evidence that distinct preferred transposon integration sites may not necessary match consensus sequences, but rather share similar structural patterns [48]. DNA structural characteristics such as bending and protein-induced deformability play an important role in directing DNA integration [28,48,49]. DNA bending can lead to changes in the width and depth of the major and minor grooves, affecting a protein's access [49]. AT-rich palindromes are particularly susceptible to local melting and have been experimentally shown to adopt a bendable DNA structure [50]. In addition, palindromic sequences have the potential to form cruciform configurations, which are an efficient target for RAG-mediated transposition [51]. Also, AT-rich palindromic repeats are known to be double-strand break hotspots. It has been proposed that DNA bending plays a role in the integration specificity of the *hobo *transposable element from the hAT family, but *hobo *has no strict preference for targeting at nucleotide level [39]. This suggests the likelihood that the target site selection of *Tol2 *is primarily determined at the level of DNA structure, not sequence.

*Tol2 *element transposes by "cut-and-paste" mechanism, which involves the excision and re-integration of the transposon from one site to another, creating an 8-bp duplication of the integration site [3,13]. Previously, we found that in one third of *Tol2 *excision events, reparation of donor site results in different footprints [13]. In our experiments we used extremely high amounts of transposase mRNA (around 9 × 10^7 ^molecules per a single copy of transposon), therefore it may be reasonable to expect multiple "cut-and-paste" events before transposon will finally settle. Such multiple hops could generate double-strand breaks and, as a consequence, the footprints. Our analysis of DNA sequences flanking the integration/target site revealed no signs of the footprints, at least, at the vicinity (up to 900 bp) of new integration sites. All DNA sequence modifications found at these regions exhibited DNA sequence polymorphism between zebrafish strains (data not shown). However, we could not rule out the possibility that the footprints left after multiple "cut-and-paste" events may be found far away from integration sites.

## Conclusion

In summary, in a large-scale ET screen we analyzed 338 insertions generated in the zebrafish genome by remobilization of a single *Tol2 *transposon copy from two independent sites on two different chromosomes. About 39% of *Tol2 *insertions occurred within genes, mostly in introns. Upon remobilization, *Tol2 *showed a preference to reintegrate within the chromosome containing the donor site. Sequence analysis of integration sites revealed no strict specificity at the nucleotide level, but *Tol2 *was prone to integrate into AT-rich regions with weak palindrome-like consensus sequences. This information should be carefully evaluated during the design of various follow-up applications that involve *Tol2*. Numerous ET lines with diverse GFP expression patterns have been generated in this work. They represent a large set of research tools for *in vivo *studies of vertebrate development, and some have already been successfully used for that purpose [22,52,53].

## Methods

### Fish lines

The ET(*krt4*:EGFP)^SqET33 ^line (referred to as SqET33) was established by coinjection of *in vitro *synthesized transposase mRNA and *Tol2 *transposon-based ET construct DNA into wild type zebrafish embryos at the one- or two-cell stage [13]. The ET(*krt4*:EGFP)^SqET33-E20 ^line (referred to as SqET33-E20) was created after *in vivo *remobilization of a single-copy of *Tol2 *transposon-based ET construct from the donor line SqET33 [22]. Fish were maintained according to established protocols [54] and in agreement with the IACUC regulations and rules of the IMCB zebrafish facility. Transgenic and wild type fish were *AB *strain.

### Plasmids

Plasmid pTem03 containing the coding region for medaka Tol2 transposase was purchased from Dr. Koga (Nagoya University, Japan) as a part of the "Gene transfer system" kit. Plasmid pDB600 containing the coding region of Tol2 transposase flanked with the 5' and 3' UTRs from the *Xenopus β-globin *gene was kindly provided by Dr. Ekker (University of Minnesota, USA).

### *In vitro *mRNA synthesis and *in vivo *transposon remobilization

Plasmids pTem03 and pDB600 were linearized with *Xba*I and *Spe*I, respectively, and used as templates for *in vitro *mRNA synthesis. Transposase mRNA was synthesized using mMESSAGE mMACHINE SP6 and T3 kits (Ambion, USA) and purified using an RNeasy Mini Kit (QIAGEN, Germany). For *in vivo *remobilization of a single-copy of *Tol2 *transposon-based ET construct, 50-100 pg of transposase mRNA was injected into zebrafish embryos from the donor lines at the one- or two-cell stage.

### Southern blot hybridization

Genomic DNA from adult fish or embryos was phenol extracted and digested using *Hin*dIII (New England Biolabs), which cut the *Tol2 *transposon-based ET cassette at a unique site. The digested genomic DNA was fractionated by agarose gel electrophoresis, transferred to a positively charged nylon membrane (Hybond-N^+^, Amersham Biosciences) by capillary blotting [55], and crosslinked by UV irradiation. The DNA probe for *EGFP *was labeled with digoxigenin (DIG) using a PCR DIG synthesis kit (Roche Applied Science). We used DIG EasyHyb buffer, an anti-DIG alkaline phosphatase conjugate antibody and CDP-Star chemiluminescent substrate (all Roche Applied Science) to detect the hybridized probe. Hybridization and detection were carried out according to the manufacturer's instructions.

### Identification and mapping of integration sites

To recover genomic sequences flanking the integrated *Tol2 *transposon we used thermal asymmetric interlaced PCR (TAIL-PCR). The DNA was isolated from one GFP-positive embryo after outcrossing of F_0 _fish with wild type fish. TAIL-PCR was performed according to Liu and Whittier [56] using the primers and cycling conditions described elsewhere [13]. The resulting PCR products were purified and directly sequenced using primers 5'-CCCCAAAATAATACTTAAGTACAG-3' and 5'-GTACTTGTACTTTCACTTGAG-3', which anneal to the 5' and 3' transposon ends, respectively. The length of the sequence reads never exceeded 900 bp. A sequence was considered to be from an authentic integration site only if it contained the *Tol2 *transposon sequence from the nested primer to the ends of the inverted repeats. In total, we amplified and sequenced 519 genomic regions flanking the 338 integration sites (see Additional file 4 for details). Of these, 159 sequences were amplified from either the 5' or the 3' end of the integrated transposon only. The sequence reads were then mapped to the zebrafish genome using BLASTN (the latest zebrafish whole genome assembly version 7 (Zv7, April 2007 freeze in the Ensembl genome browser) . In some cases, the flanking sequences were blasted against the unfinished high-throughput genomic sequences (htgs) database or trace archive at NCBI . We considered the sequence to be from a unique integration site if it matched to no more than one genomic locus with 95% or greater identity to the genomic sequence over the high-quality sequence region (a whole length of sequence read). On the basis of this criterion, 423 sequences reads from 275 integration sites could be unambiguously mapped to unique genomic loci. Sixty sequence reads from 38 integration sites were matched to more than one genomic locus with 95% or greater identity to the genomic sequence. In those cases, only the hits with highest identity (>99%) and score over the whole length of sequence read were considered. Thus, 20 sequence reads from 12 integration sites were unmistakably mapped to distinct genomic loci. The remaining 40 sequence reads from 26 integration sites could not be mapped unambiguously. We also had 26 sequence reads from 17 integration sites that were either matched with less than 95% of identity to the genomic sequence, or had no significant similarity to the genomic database. Half the sequence reads that had no hits in the latest version of the database (Zv7) were matched to a single genomic locus with 98% or greater identity to the genomic sequence over the sequence region in the previous zebrafish whole genome assembly, version Zv6. Nevertheless, such sequence reads were not considered to be matched. In addition, 10 sequences from 8 integration sites represented short (<100 bp) repetitive dinucleotide sequence reads that could not be mapped to any location. Ultimately, we were able to map 287 integration sites out of 338 integration events to unique genomic loci.

### Bioinformatics

We defined integration as having landed in a gene only if it was within the genomic coordinates of the 21,322 protein-coding genes or transient EST gene models annotated in the zebrafish genome. The 5' and 3' ends of the genes were considered the first and last nucleotide positions according to gene coordinates in the latest zebrafish whole genome assembly, Zv7. We analyzed the base composition over a 48-bp region encompassing the *Tol2 *target site using the computer program WebLogo (version 3.0) . We also analyzed integrations in relation to various genomic repeat elements annotated in the zebrafish genome. The expected number of insertions in each chromosome (if integration events were random) was calculated as follows: (1) to calculate the expected distribution of insertions, the total number of bases in the zebrafish genome was divided by the number of mapped insertions; (2) to calculate the expected number of insertions on each chromosome, the length of each individual chromosome was divided by the expected distribution of insertions. The total number of base pairs in the zebrafish genome (reference assembly length) according to the Zv7 assembly is 1,440,582,308 base pairs. Integration into chromosomes was tested for statistical bias using a χ^2 ^test to compare the observed number of integrations into a particular chromosome to the value expected if integration events were random.

## List of abbreviations

ET: enhancer trap; GFP: green fluorescent protein; EGFP: enhanced GFP; LTR: long terminal repeats; ORF: open reading frame; SB: *Sleeping Beauty *transposon; TAIL-PCR: thermal asymmetric interlaced PCR; UTR: untranslated region; WT: wild type; bp: base pairs.

## Authors' contributions

Conceived and designed the experiments: IK, AE, SP and VK. Performed the experiments: IK and MGL. Analyzed the data: IK, MGL, AE, SP and VK. Wrote the manuscript: IK, SP and VK. All authors read and approved the final manuscript.

## Supplementary Material

Additional file 1 **GFP segregation in F_1 _generation**. Table S1 shows the number of novel expression patterns and GFP segregation ratios in the F_1 _generation. Transgenic embryos (F_0_) carrying a single heterozygous *Tol2 *insert in their genome (gfp^+/-^) were injected with transposase mRNA, raised to maturity, and outcrossed to wild-type fish. Click here for file

Additional file 2 **Southern blot hybridization of F_1 _generation**. Figure S1 shows the *Tol2 *copy number in F_1 _fish. DNA was isolated from individual F_1 _fish that originated from the same F_0 _founder. The DNA samples were digested with *Hin*dIII and hybridized with DIG-labeled *EGFP *probe. Click here for file

Additional file 3 **Integration sites and genes located near the insertions**. Table S2 shows the integration loci and nearest genes targeted by *Tol2 *remobilized from two different donor sites. Targeted repetitive elements are also shown. Click here for file

Additional file 4 **Flanking genomic sequences of *Tol2 *transposon insertions**. Table S3 shows the DNA sequence reads of the regions that flank *Tol2 *transposon insertions. To obtain the genomic locations of *Tol2 *insertions, the flanking sequence reads were blasted against the latest zebrafish genome sequence database (zebrafish whole genome assembly, version 7) from the Ensembl genome browser . In some cases, the flanking sequence reads were blasted against the unfinished high-throughput genomic sequence (htgs) database or trace archive at NCBI . Click here for file

Additional file 5 **Target site analysis for 368 genomic *Tol2 *insertions**. Table S4 shows the base composition of an 8-bp integration site in 368 genomic *Tol2 *insertions. For the region flanking the integration site only the first three nucleotides are shown. Click here for file
