# Supplementary material for: Genome-wide analysis of Tol2 transposon reintegration in zebrafish
Source: BMC Genomics. 2009 Sep 8;10:418. doi: 10.1186/1471-2164-10-418 (PMC2753552; doi:10.1186/1471-2164-10-418)
Supplement: Additional file 1 — GFP segregation in F1 generation. Table S1 shows the number of novel expression patterns and GFP segregation ratios in the F1 generation. Transgenic embryos (F0) carrying a single heterozygous Tol2 insert in their genome (gfp+/-) were injected with transposase mRNA, raised to maturity, and outcrossed to wild-type fish. [file 1471-2164-10-418-S1.pdf]

Table S1 - GFP segregation in F_1 generation

F_0 founder fish	GFP- / GFP+ in F_1	New GFP patterns	Remarks
ET33-mi1	216/247	3	
ET33-mi2	244/247	2	
ET33-mi3	115/149	3	
ET33-mi4	45/78	1	*Tol2* copies increase
ET33-mi5	90/183	7	*Tol2* copies increase
ET33-mi6	41/25	2	Loss of *Tol2*
ET33-mi7	19/39	2	*Tol2* copies increase
ET33-mi9	104/139	4	
ET33-mi10	61/74	2	
ET33-mi12	171/109	1	Loss of *Tol2*
ET33-mi13	98/47	2	Loss of *Tol2*
ET33-mi15	60/71	3	
ET33-mi16	132/151	1	
ET33-mi17	67/94	2	
ET33-mi18	34/25	1	
ET33-mi19	60/50	1	
ET33-mi20	55/25	1	Loss of *Tol2*
ET33-mi21	30/17	1	Loss of *Tol2*
ET33-mi22	78/31	2	Loss of *Tol2*
ET33-mi23	59/73	2	
ET33-mi24	48/46	1	
ET33-mi25	30/49	1	
ET33-mi26	114/202	2	*Tol2* copies increase
ET33-mi27	22/15	1	
ET33-mi28	109/111	1	
ET33-mi29	38/34	2	
ET33-mi30	70/45	1	Loss of *Tol2*
ET33-mi31	44/35	1	
ET33-mi32	36/28	3	
ET33-mi33	34/78	2	*Tol2* copies increase
ET33-mi34	57/52	1	
ET33-mi35	32/32	2	
ET33-mi36	40/40	2	
ET33-mi37	42/52	2	
ET33-mi38	46/64	1	
ET33-mi39	57/70	2	
ET33-mi40	41/31	1	
ET33-mi41	35/70	1	*Tol2* copies increase
ET33-mi42	98/155	1	*Tol2* copies increase
ET33-mi43	66/69	1	
ET33-mi44	40/49	3	
ET33-mi45	24/40	1	

F_0 founder fish	GFP- / GFP+ in F_1	New GFP patterns	Remarks
ET33-mi46	142/49	1	Loss of *Tol2*
ET33-mi47	108/87	1	
ET33-mi48	86/104	4	
ET33-mi49	68/43	1	Loss of *Tol2*
ET33-mi50	87/79	1	
ET33-mi51	27/22	1	
ET33-mi52	60/81	1	
ET33-mi53	67/49	1	
ET33-mi54	113/71	1	Loss of *Tol2*
ET33-mi55	91/34	1	Loss of *Tol2*
ET33-mi56	34/46	2	
ET33-mi57	49/49	1	
ET33-mi58	77/84	2	
ET33-mi59	87/69	3	
ET33-mi60	16/80	2	*Tol2* copies increase
ET33-mi61	34/52	2	
ET33-mi62	141/37	3	Loss of *Tol2*
ET33-mi63	49/81	3	*Tol2* copies increase
ET33-mi64	31/36	1	
ET33-mi65	47/51	1	
ET33-mi66	48/54	1	
ET33-mi67	42/44	2	
ET33-mi68	68/54	1	
ET33-mi69	31/40	1	
ET33-mi70	36/60	1	*Tol2* copies increase
ET33-mi71	30/76	2	*Tol2* copies increase
ET33-mi72	55/20	1	Loss of *Tol2*
ET33-mi73	59/42	1	
ET33-mi74	50/94	3	*Tol2* copies increase
ET33-mi75	52/94	3	*Tol2* copies increase
ET33-mi76	55/48	2	
ET33-mi77	33/37	1	
ET33-mi78	71/63	1	
ET33-mi79	88/77	1	
ET33-mi80	149/46	1	Loss of *Tol2*
ET33-mi81	35/35	1	
ET33-mi82	60/66	1	
ET33-mi83	30/28	1	
ET33-mi84	200/78	1	Loss of *Tol2*
ET33-mi85	146/170	2	
ET33-mi86	48/80	2	*Tol2* copies increase
ET33-mi87	24/84	1	*Tol2* copies increase
ET33-mi88	52/71	1	
ET33-mi89	77/85	2	

F_0 founder fish	GFP- / GFP+ in F_1	New GFP patterns	Remarks
ET33-mi90	49/40	1	
ET33-mi91	89/89	3	
ET33-mi92	56/51	2	
ET33-mi93	49/70	2	
ET33-mi94	26/30	3	
ET33-mi95	155/22	1	Loss of *Tol2*
ET33-mi96	80/30	2	Loss of *Tol2*
ET33-mi97	36/44	1	
ET33-mi98	30/42	2	
ET33-mi99	26/32	1	
ET33-mi100	63/153	1	*Tol2* copies increase
ET33-mi101	99/53	1	Loss of *Tol2*
ET33-mi102	38/21	1	Loss of *Tol2*
ET33-mi103	30/20	1	
ET33-1	99/128	2	
ET33-3	136/94	1	
ET33-9	72/48	1	
ET33-14	64/57	1	
ET33-15	62/102	1	*Tol2* copies increase
ET33-18	46/54	1	
ET33-24	49/71	1	
ET33-A1	38/47	1	
ET33-B13	83/70	1	
ET33-D10	81/92	1	
ET33-E20	81/89	1	
ET33-F38	88/114	1	
ET33-H8	116/141	1	
ET33-J1	80/90	1	
ET33-J12	47/74	2	
ET33-K11	95/99	1	
ET33-K15	167/125	1	
ET33-K23	34/32	1	
E1	27/9	-	Loss of *Tol2*
E2	39/1	-	Loss of *Tol2*
E10	152/0	-	Loss of *Tol2*
E14	35/4	-	Loss of *Tol2*
E15	109/4	-	Loss of *Tol2*
E16	48/10	-	Loss of *Tol2*
E21	30/11	-	Loss of *Tol2*
E26	99/4	-	Loss of *Tol2*
E27	126/25	-	Loss of *Tol2*
G2	55/6	-	Loss of *Tol2*
G20	98/30	-	Loss of *Tol2*
Gateways-1	33/40	1	

F_0 founder fish	GFP- / GFP+ in F_1	New GFP patterns	Remarks
Gateways-2	66/80	3	
Gateways-3	37/42	1	
Gateways-4	68/43	2	Loss of *Tol2*
Gateways-6	57/56	1	
Gateways-7	67/93	3	
Gateways-8	83/81	2	
Gateways-9	64/64	2	
Gateways-10	28/38	2	
Gateways-11	65/35	1	Loss of *Tol2*
Gateways-12	70/113	2	*Tol2* copies increase
Gateways-13	32/45	1	
Gateways-14	35/33	1	
Gateways-15	48/40	1	
Gateways-16	36/60	1	*Tol2* copies increase
Gateways-17	15/17	1	
Gateways-18	19/19	2	
Gateways-19	37/18	4	Loss of *Tol2*
Gateways-20	14/15	1	
Gateways-21	131/92	3	Loss of *Tol2*
Gateways-22	28/30	-	
Gateways-23	80/90	1	
Gateways-24	66/53	1	
Gateways-25	50/80	1	*Tol2* copies increase
Gateways-26	41/39	1	
Gateways-27	10/11	1	
Gateways-28	28/14	1	Loss of *Tol2*
Gateways-29	33/45	1	
Gateways-30	82/103	1	
Gateways-31	27/19	1	
Gateways-32	35/79	1	*Tol2* copies increase
Gateways-33	106/98	1	
Gateways-34	51/39	1	
Gateways-35	143/140	1	
Gateways-36	28/95	1	*Tol2* copies increase
Gateways-37	51/36	2	
Gateways-38	189/182	2	
Gateways-39	39/33	1	
Gateways-40	24/16	1	
Gateways-41	95/100	2	
Gateways-42	58/87	2	
Gateways-43	47/55	1	
Gateways-44	29/36	3	
Gateways-45	95/97	3	
Gateways-46	99/71	1	

F_0 founder fish	GFP- / GFP+ in F_1	New GFP patterns	Remarks
Gateways-47	42/12	-	Loss of *Tol2*
Gateways-48	49/58	1	
Gateways-49	37/35	-	
Gateways-50	133/71	1	Loss of *Tol2*
Gateways-51	77/75	3	
Gateways-52	52/64	4	
Gateways-53	73/52	2	
Gateways-54	81/81	1	
Gateways-55	30/42	1	
Gateways-56	40/64	4	
Gateways-57	67/84	5	
Gateways-58	79/52	1	
Gateways-59	135/114	3	
Gateways-60	160/118	2	
Gateways-61	104/97	2	
Gateways-62	38/23	1	
Gateways-63	69/54	2	
Gateways-64	42/44	2	
Gateways-65	39/39	-	
Gateways-66	87/80	3	
Gateways-68	20/17	3	
Gateways-69	81/63	1	
Gateways-70	113/100	1	
Gateways-71	133/123	4	
Gateways-72	91/97	1	
Gateways-73	45/50	1	
Gateways-74	54/59	1	
Gateways-75	74/97	3	
Gateways-76	54/63	1	
Gateways-77	92/91	1	
Gateways-78	21/14	1	
Gateways-79	97/106	4	
Gateways-80	16/23	3	
Gateways-81	59/56	3	
Gateways-82	122/123	1	
Gateways-83	147/142	1	
Gateways-84	72/64	3	
Gateways-85	144/134	1	
Gateways-86	89/63	5	
Gateways-87	122/112	1	
Gateways-88	55/54	2	
Gateways-89	202/207	1	
Gateways-90	48/62	4	

Transgenic embryos (F_0) carrying a single heterozygous *Tol2* insert in their genome ($gfp^{+/-}$) were injected with transposase mRNA, raised to maturity, and outcrossed to wild-type fish. The number of novel expression patterns and GFP segregation ratios in the F_1 generation are shown.
