# Supplementary material for: Genome-wide analysis of Tol2 transposon reintegration in zebrafish
Source: BMC Genomics. 2009 Sep 8;10:418. doi: 10.1186/1471-2164-10-418 (PMC2753552; doi:10.1186/1471-2164-10-418)
Supplement: Additional file 2 — Southern blot hybridization of F1 generation. Figure S1 shows the Tol2 copy number in F1 fish. DNA was isolated from individual F1 fish that originated from the same F0 founder. The DNA samples were digested with HindIII and hybridized with DIG-labeled EGFP probe. [file 1471-2164-10-418-S2.pdf]

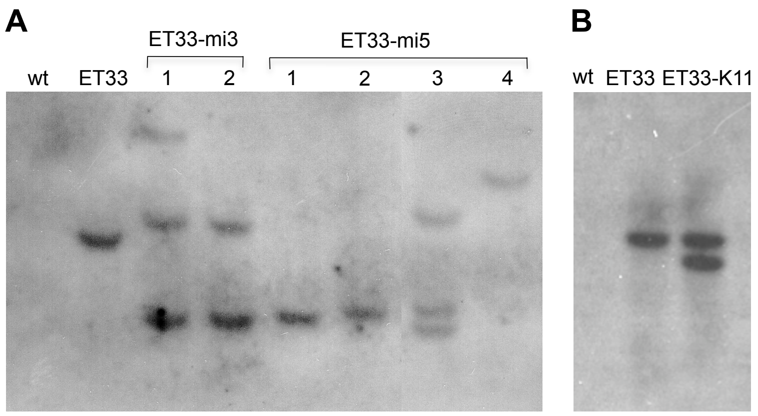

Figure S1 - Evaluation of the *Tol2* copy numbers in F_1 fish

DNA was isolated from individual F_1 fish (shown as the numbers) that originated from the same F_0 founder (shown as ET33-mi3, ET33-mi5 or ET33-K11). The DNA samples were digested with *Hin*dIII and hybridized with DIG-labeled *EGFP* probe. (A) and (B) represent the separate blots. wt, wild type; ET33, donor line.
