# Supplementary material for: Genome-wide analysis of Tol2 transposon reintegration in zebrafish
Source: BMC Genomics. 2009 Sep 8;10:418. doi: 10.1186/1471-2164-10-418 (PMC2753552; doi:10.1186/1471-2164-10-418)
Supplement: Additional file 3 — Integration sites and genes located near the insertions. Table S2 shows the integration loci and nearest genes targeted by Tol2 remobilized from two different donor sites. Targeted repetitive elements are also shown. [file 1471-2164-10-418-S3.pdf]

Table S2 - Integration sites and genes located near the insertions

Donor line: SqET33, chromosome 14 (n=196)			
Insertion name	Chromosome	Integration locus and nearest gene	Repetitive element [a]
ET33 [b]	14	exon of *zic6*	
ET33-1A	22	exon of *si:dkey-42i9.10*	
ET33-1B [c]	ND	ND	Dr000359
ET33-2	24	intron of *zgc:112332*	Tandem repeat
ET33-3	3	exon of *zgc:113411*	
ET33-9 [d]	ND	ND	Tandem repeat
ET33-10	14	intron of *LOC572044*	
ET33-14	3	5.5 kb downstream of *LOC100000689*	Dr000292
ET33-15	19	0.8 kb upstream of *zgc:73376*	TDR14
ET33-18	20	intron of *zgc:110750*	
ET33-24	21	44.7 kb upstream of *tmsb*	
ET33-A1	16	intron of *ENSDARG00000035023*	Tandem repeat
ET33-B13	23	intron of *ENSDARG00000029146*	
ET33-D10	22	intron of *ENSDARG00000027618*	
ET33-E20 [b]	24	4.2 kb upstream of *zgc:66340*	
ET33-F38A	6	3.1 kb upstream of *zgc:112416*	
ET33-H8 [e]	ND	ND	
ET33-H23	16	exon of *a2bp1l*	
ET33-J1	Un	no tagged genes	Dr001186
ET33-J12A	4	2.8 kb downstream of *si:dkey-207j16.6*	
ET33-J12B	18	1.5 kb downstream of *ppp1r13l*	
ET33-K11	Un	0.2 kb upstream of *ENSDARESTG00000012725*	
ET33-K15 [e]	ND	ND	
ET33-K23 [e]	ND	ND	Dr000176
ET33-mi1A	2	intron of *ENSDARG00000008548*	Dr000671
ET33-mi1B	14	41.7 kb downstream of *zic3*	DNA14-5-1_DR
ET33-mi1C	20	intron of *taf1a*	Dr000372
ET33-mi2A	14	intron of *proml1*	Dr000099
ET33-mi2B	10	0.3 kb upstream of *ddx55*	CR1-1_DR
ET33-mi3A	14	2.2 kb upstream of *fhla*	
ET33-mi3B	14	intron of *ENSDARG00000034820*	
ET33-mi3C	3	0.2 kb downstream of *zgc:109934*	HE1_DR1
ET33-mi4	16	7.2 kb upstream of *zgc:56494*	HE1_DR1
ET33-mi5A	14	26.1 kb downstream of *gria2b*	
ET33-mi5B	11	exon of *zgc:77948*	
ET33-mi5C	20	intron of *si:ch211-11m18.2*	
ET33-mi5D	23	0.6 kb downstream of *zgc:165664*	ANGEL
ET33-mi5E	25	intron of *ENSDARG00000063147*	
ET33-mi5F	24	exon of *ENSDARG00000039801*	
ET33-mi5G	16	3.7 kb upstream of *zgc:153234*	
ET33-mi6A [d]	ND	ND	Tandem repeat
ET33-mi6B	10	0.7 kb downstream of *ENSDARG00000027523*	
ET33-mi7A	7	138.0 kb downstream of *ENSDARG00000036664*	
ET33-mi7B [e]	2, 21	ND	Tandem repeat

Insertion name	Chromosome	Integration locus	Repetitive element [a]
ET33-mi8	Un	no tagged genes	Dr000981
ET33-mi9A	18	2.9 kb upstream of *sf3b3*	
ET33-mi9B	Un	no tagged genes	
ET33-mi9C-1	10	17.6 kb upstream of *zgc:112247*	Tandem repeat
ET33-mi9C-2 [e]	2, 18	ND	Dr001213
ET33-mi9D	11	intron of *uts2b*	TDR16
ET33-mi10A	16	intron of *zgc:158866*	
ET33-mi10B	10	exon of *GALT*	
ET33-mi11	7	exon of *ENSDARESTG00000018669*	
ET33-mi12	23	0.3 kb upstream of *ENSDARESTG00000016430*	
ET33-mi13B	5	56.5 kb downstream of *ENSDARG00000042404*	Dr000047
ET33-mi14A	9	intron of *gad1*	DNA11TA1_DR
ET33-mi14B	Un	8.4 kb upstream of *ENSDARG00000031848*	Dr000294
ET33-mi15A	Un	no tagged genes	Tandem repeat
ET33-mi15B	11	intron of *magi1*	
ET33-mi15C	8	intron of *ENSDARG00000068028*	Dr000188
ET33-mi15ix	6	96 kb downstream of *LOC567057*	
ET33-mi16 [d]	ND	ND	Tandem repeat
ET33-mi17A	11	4.2 kb downstream of *zgc:162897*	
ET33-mi17Aix	17	34.5 kb upstream of *ENSDARG00000063068*	Dr000058
ET33-mi17B	13	intron of *zgc:112390*	TDR18
ET33-mi17Bix	8	2.4 kb downstream of *LOC560602*	
ET33-mi18	6	exon of *ENSDARG00000060235*	
ET33-mi19	12	6.4 kb upstream of *hbbe2*	DNA9TA1_DR
ET33-mi20	14	exon of *neurog1*	
ET33-mi21	Un	no tagged gens	
ET33-mi22A	19	3.6 kb downstream of *crtap*	ANGEL
ET33-mi22B	25	3.2 kb upstream of *zgc:63696*	Tc1-1_DR
ET33-mi23A	20	13.7 kb upstream of *cnr1*	
ET33-mi23B	14	intron of *fgf13*	
ET33-mi24	6	95.6 downstream of *hdlbp*	TZF28
ET33-mi25 [e]	7, 8	ND	Dr000313
ET33-mi26A	3	intron of *ENSDARG00000005159*	DNA8-3_DR
ET33-mi26D	6	intron of *zgc:92712*	
ET33-mi27A	11	exon of *gdf11*	
ET33-mi28	14	intron of *fgf13*	
ET33-mi29A [e]	5,12	1.6 kb downstream of *LOC797542*	
ET33-mi29B [c]	ND	ND	
ET33-mi30	Un	intron of *ENSDARG00000061078*	
ET33-mi31	5	intron of *aacs*	
ET33-mi32A [c]	ND	ND	
ET33-mi32B	16	58.1 kb upstream of *LOC795124*	
ET33-mi32C	20	exon of *zgc:136808*	
ET33-mi33A	14	0.5 kb downstream of *foxa*	Dr000134
ET33-mi33C	14	intron of *NUP62CL*	
ET33-mi34	2	8.8 kb upstream of *ENSDARG00000039174*	
ET33-mi35A	7	25.1 kb upstream of *zgc:55686*	
ET33-mi35B [c]	ND	ND	

Insertion name	Chromosome	Integration locus	Repetitive element [a]
ET33-mi36A	24	intron of *ENSDARG00000071685*	
ET33-mi36B	12	0.6 kb downstream of *ENSDARG00000057193*	
ET33-mi37A [c]	ND	ND	Dr000386
ET33-mi37B [e]	ND	ND	
ET33-mi38	14	5.5 kb downstream of *sox3*	
ET33-mi39A	12	141.7 kb downstream of *zgc:113062*	
ET33-mi39B [e]	ND	ND	Dr000221
ET33-mi40	15	intron of *ENSDARG00000039489*	
ET33-mi42	13	intron of *zgc:109897*	
ET33-mi43	Un	0.7 kb downstream of *ENSDARG00000068272*	
ET33-mi44A	14	3.5 kb downstream of *ENSDARG00000034344*	
ET33-mi44B	11	3.4 kb downstream of *phf2*	
ET33-mi44C	22	16.2 kb upstream of *zgc:112958*	
ET33-mi45 [e]	ND	ND	DIRS1a_DR
ET33-mi46 [d]	ND	ND	Tandem repeat
ET33-mi47	19	intron of *ENSDARG00000033473*	Dr000175
ET33-mi48A	19	63.4 kb downstream of *mbp*	Dr000312
ET33-mi48D	14	intron of *gnb2l1*	ANGEL
ET33-mi49	1	intron of *zgc:112094*	
ET33-mi50	2	2.3 downstream of *ENSDARG00000003909*	Dr001219
ET33-mi51 [e]	ND	ND	LOOPERN2_DR
ET33-mi52	11	3.1 kb downstream of *ENSDARG00000070600*	
ET33-mi53	22	3.5 kb upstream of *si:dkey-188p4.2*	
ET33-mi54	3	2.2 kb downstream of *znfl1*	HATN8_DR
ET33-mi55	23	1.9 kb upstream of *zgc:110844*	SINE3-1a
ET33-mi56A	7	intron of *fto*	
ET33-mi56B	19	16.9 kb upstream of *si:ch211-232m10.6*	TDR13
ET33-mi57	11	54.8 kb downstream of *zgc:100947*	
ET33-mi58A	6	114.4 kb downstream of *pou4f1*	HARBINGER3_DR
ET33-mi58B	15	2.3 kb upstream of *sst1*	
ET33-mi59A	5	36.7 kb upstream of *ENSDARG00000067804*	
ET33-mi59B	14	17.7 kb downstream of *fgf13*	
ET33-mi59C	1	2.7 kb upstream of *ENSDARG00000041157*	HE1_DR1
ET33-mi60	3	24.9 kb upstream of *lfng*	Tc1N1_DR
ET33-mi61A	13	92.9 kb upstream of *abcg2c*	
ET33-mi61B	Un	intron of *nrxn3b*	
ET33-mi62B	24	91.0 kb upstream of *tnikb*	
ET33-mi63A	15	110.4 kb upstream of *LOC557900*	LOOPERN3_DR
ET33-mi63B	22	1.4 kb upstream of *zgc:113191*	
ET33-mi63C	13	intron of *zgc:110133*	TDR3
ET33-mi64	15	0.3 kb upstream of *zgc:114089*	
ET33-mi65A	20	intron of *snap25a*	
ET33-mi65B	20	21.2 kb upstream of *stmn4*	Tandem repeat
ET33-mi66	14	19.9 kb downstream of *zic3*	Dr000591
ET33-mi67A	14	85.2 kb downstream of *rraga*	
ET33-mi67B	15	1.0 kb downstream of *zgc:114114*	HARBINGER3_DR
ET33-mi68	2	14.3 kb downstream of *ENSDARG00000052459*	SINE_DR1
ET33-mi69-1	9	intron of *ENSDARESTG00000004124*	Looper-N7_DR

Insertion name	Chromosome	Integration locus	Repetitive element [a]
ET33-mi69-2	9	intron of *ENSDARESTG00000004124*	
ET33-mi69-3	5	3.2 kb upstream of *ENSDARG00000059638*	TDR14
ET33-mi70-1	16	intron of *si:dkey-154b17.1*	
ET33-mi70-2	12	exon of *glud1b*	
ET33-mi71A	12	189.6 kb downstream of *zgc:158829*	Dr000221
ET33-mi71B	Un	0.3 kb upstream of *zgc:77106*	
ET33-mi72-1	20	intron of *ENSDARG00000060554*	
ET33-mi72-2	20	19.3 kb upstream of *si:dkeyp-115a10.3*	
ET33-mi73	7	intron of *orc6l*	
ET33-mi74A	6	0.5 kb downstream of *gbx2*	
ET33-mi74B	14	intron of *zgc:92770*	TDR8
ET33-mi74C	18	38.8 kb downstream of *ENSDARG00000061233*	
ET33-mi75A	16	intron of *zgc:110131*	
ET33-mi75B	8	1.9 kb downstream of *ENSDARG00000025013*	Dr000324
ET33-mi75C	16	114.8 kb downstream of *ENSDARG00000046087*	
ET33-mi76A	14	1.0 kb upstream of *phf6*	
ET33-mi76B	13	37.7 kb upstream of *zgc:153171*	
ET33-mi77-1 [e]	ND	ND	Dr000097
ET33-mi77-2	14	7.5 kb downstream of *zgc:92770*	
ET33-mi78	10	31.0 kb upstream of *igsf4d*	TZF28
ET33-mi79	1	exon of *ENSDARESTG00000016360*	
ET33-mi80 [d]	ND	ND	Tandem repeat
ET33-mi81 [d]	ND	ND	Tandem repeat
ET33-mi82	7	exon of *tppp3*	
ET33-mi83	Un	no tagged genes	
ET33-mi84	2	intron of *pard3*	
ET33-mi85A	20	0.2 kb downstream of *zgc:101095*	
ET33-mi85B	23	13.7 kb upstream of *LOC565797*	HE1_DR1
ET33-mi86A	2	intron of *scinlb*	SINE3-1a
ET33-mi86B	3	65.7 kb downstream of *LOC562973*	Dr000562
ET33-mi87 [e]	ND	ND	CR1-1_DR
ET33-mi88	21	56.5 upstream of *rbm22*	
ET33-mi89A	8	40.7 kb upstream of *ENSDARG00000061989*	
ET33-mi89B	14	intron of *ENSDARG00000061603*	DNA11TA1_DR
ET33-mi90	8	intron of *pptc7*	
ET33-mi91A	11	94.6 kb downstream of *sb:cb363*	
ET33-mi91B	19	8.8 kb downstream of *si:dkey-109n11.1*	
ET33-mi91C	11	120.8 kb downstream of *sb:cb363*	
ET33-mi92A	4	7.3 kb upstream of *si:dkey-3n7.3*	
ET33-mi92B [c]	ND	ND	
ET33-mi93A-1	14	exon of *zgc:63527*	
ET33-mi93A-2	14	intron of *zgc:63527*	
ET33-mi93A-3	14	intron of *zgc:63527*	
ET33-mi93B	4	intron of *celsr1a*	
ET33-mi94A	3	3.6 kb downstream of *ENSDARG00000037916*	Dr000258
ET33-mi94B	4	1.7 kb downstream of *cry1a*	
ET33-mi94C	19	intron of *thrap6*	Dr000292
ET33-mi94D	Un	no tagged genes	HAT1N_DR

Insertion name	Chromosome	Integration locus	Repetitive element [a]
ET33-mi95	7	4.3 kb upstream of *bdnf*	TZF28
ET33-mi96A	17	73.3 kb downstream of *ENSDARG00000070954*	
ET33-mi96C [c]	ND	ND	
ET33-mi98B	21	332.0 kb upstream of *LOC563544*	
ET33-mi99	3	85.9 kb upstream of *nudt1*	HE1_DR1
ET33-mi101-1 [e]	ND	ND	HARBINGER3N_DR
ET33-mi101-2	18	0.1 kb downstream of *icln*	
ET33-mi102	Un	36.2 kb downstream of *ENSDARG00000069561*	Dr000279

Donor line: SqET33-E20, chromosome 24 (n=143)

Insertion name	Chromosome	Integration locus	Repetitive element [a]
Gateways-1	23	54.5 kb upstream of *LOC797284*	
Gateways-2A	19	39.8 kb downstream of *twistnb*	
Gateways-2B-1 [d]	ND	ND	Tandem repeat
Gateways-2B-2	6	intron of *ENSDARG00000023771*	Kolobok-1_DR
Gateways-3 [d]	ND	ND	Tandem repeat
Gateways-4A	17	0.8 kb downstream of *zgc:77069*	
Gateways-4B [e]	ND	ND	Dr001028
Gateways-6	23	4.9 kb downstream of *zgc:153779*	
Gateways-7A	24	56.8 kb downstream of *ENSDARESTG00000001944*	
Gateways-7B	7	3.3 kb downstream of *ENSDARG0000069110*	TDR15
Gateways-7C-1	24	intron of *ctnnd2*	
Gateways-7C-2	24	exon of *ENSDARG00000062934*	
Gateways-8A [c]	ND	ND	
Gateways-8B	5	26.4 kb downstream of *ENSDARG00000013434*	
Gateways-9A [c]	ND	ND	
Gateways-9B	6	1.6 kb upstream of *cyc1*	
Gateways-10A	5	intron of *si:ch211-31p3.2*	HARBINGERN5_DR
Gateways-10B	11	intron of *ENSDARESTG00000019293*	
Gateways-11	12	242.5 kb downstream of *ENSDARESTG00000001338*	Dr000240
Gateways-12A	14	2.3 kb upstream of *foxa*	
Gateways-12D	7	intron of *six7*	ANGEL
Gateways-13 [c]	ND	ND	
Gateways-15	3	intron of *ENSDARG00000070956*	Dr000173
Gateways-16	8	intron of *ENSDARG00000056252*	
Gateways-17 [c]	ND	ND	
Gateways-18B [e]	ND	ND	Dr000679
Gateways-19A	4	2.8 kb upstream of *cmas*	
Gateways-19B	23	6.8 kb upstream of *Q1RLV9_BRARE*	HE1_DR1
Gateways-20	15	intron of *zgc:153368*	Dr000108
Gateways-21A	13	exon of *hif1an*	
Gateways-21D [e]	ND	ND	
Gateways-23-1	13	0.2 kb upstream of *yipf3*	
Gateways-23-2	21	52.3 kb downstream of *cnot6*	Dr000446
Gateways-24	2	0.7 kb downstream of *zgc:85851*	
Gateways-25	7	5.2 kb downstream of *zgc:101613*	
Gateways-26	23	1.3 kb downstream of *ENSDARG00000036952*	
Gateways-27	5	31.6 kb upstream of *ENSDARG00000067536*	HE1_DR1

Insertion name	Chromosome	Integration locus	Repetitive element [a]
Gateways-29	24	31.6 kb upstream of *ENSDARESTG00000025082*	
Gateways-30	24	intron of *zgc;153639*	
Gateways-32	7	intron of *ENSDARG00000008380*	
Gateways-33	24	5.3 kb downstream of *phex*	
Gateways-34A	13	2.6 kb upstream of *ENSDARG00000020147*	Dr000094
Gateways-35	19	exon of *hoxa5a*	
Gateways-36 [c]	ND	ND	
Gateways-37A	19	intron of *zgc:158766*	
Gateways-37B	9	intron of *wdfy2*	
Gateways-38A	2	21.5 kb upstream of *ENSDARG00000057284*	VIRDR1
Gateways-39A	23	12.8 kb downstream of *c20orf14*	
Gateways-40	24	intron of *LOC560346*	
Gateways-41A [e]	ND	ND	Dr000724
Gateways-41B	17	intron of *myo6b*	
Gateways-42A	24	1.8 kb upstream of *zgc:66340*	
Gateways-42B-1 [c]	ND	ND	
Gateways-42B-2	24	intron of *zgc:66340*	Dr000293
Gateways-43-1	10	intron *ENSDARG00000069487*	
Gateways-43-2	14	3.8 kb downstream of *ENSDARG00000034820*	
Gateways-44A	7	intron of *cugbp1*	
Gateways-44C	9	intron of *si:busm1-60j23.1*	
Gateways-44D	18	intron of *ENSDARG00000061397*	
Gateways-45A	13	0.6 kb downstream of *ENSDARG00000060656*	
Gateways-45C	24	intron of *zgc:66340*	
Gateways-46	Un	intron of *ENSDARG00000031774*	
Gateways-50A	17	23.7 kb upstream of *prox1*	
Gateways-51A	1	2.5 kb upstream of *zgc:111983*	Dr000160
Gateways-51B [e]	ND	ND	MOSAT_DR
Gateways-51C	13	41.1 kb upstream of *opn4*	Dr000266
Gateways-52A	4	intron of *ENSDARG00000069680*	Dr000332
Gateways-52B	4	1.0 kb upstream of *si:dkey-217k21.1*	
Gateways-52C	15	0.1 kb upstream of *ENSDARESTG00000003049*	
Gateways-52D	7	0.7 kb upstream of *zgc:162904*	
Gateways-53A [c]	ND	ND	
Gateways-53B [e]	ND	ND	Dr000861
Gateways-54	15	76.1 kb upstream of *cwf19l2*	
Gateways-55	10	13.2 kb upstream of *ENSDARG00000044010*	
Gateways-56A	5	2.2 kb upstream of *zgc:136597*	Dr000133
Gateways-56B	24	exon of *ENSDARESTG00000019355*	
Gateways-56C	24	intron of *ENSDARG00000071593*	DNA2-1_DR
Gateways-56D	Un	intron of *ENSDARESTG00000003539*	Dr001173
Gateways-57A	2	2.0 kb upstream of *si:dkeyp-51f11.7*	
Gateways-57D	16	upstream of *zgc:56148*	
Gateways-57E	25	17.7 kb downstream of *cib2*	DANA
Gateways-57F	22	intron of *ENSDARG00000013834*	
Gateways-58-1	2	0.5 kb downstream of *id:ibd5007*	
Gateways-58-2	21	7.7 kb upstream of *zgc;113348*	HE1_DR1
Gateways-59A	6	intron of *chrm2*	TC1DR3

Insertion name	Chromosome	Integration locus	Repetitive element [a]
Gateways-59B	24	141.2 kb upstream of *ENSDARESTG00000025082*	
Gateways-59C [e]	ND	ND	LOOPERN1_DR
Gateways-60A	16	45.0 kb upstream of *ctnnb1*	
Gateways-60C	7	21.2 kb upstream of *rbpjb*	
Gateways-61 [e]	ND	ND	
Gateways-62	6	intron of *ENSDARG00000059642*	
Gateways-63A	13	26.6 kb downstream of *zgc:101672*	
Gateways-63B	24	intron of *GENSCAN00000013885*	hAT-2n1_DR
Gateways-64A	6	4.0 kb downstream of *zgc:92785*	
Gateways-64B [e]	ND	ND	
Gateways-64C	16	31.8 kb upstream of *zgc:114140*	Tandem repeat
Gateways-66A	7	6.8 kb downstream of *efnb3*	
Gateways-66B	Un	16.6 kb upstream of *ENSDARG00000053299*	
Gateways-67A	19	20.7 kb downstream of *ENSDARG00000059149*	Dr000915
Gateways-68A	8	153.7 kb upstream of *ENSDARG00000069482*	
Gateways-68B [e]	ND	ND	
Gateways-69A	18	exon of *ENSDARG00000058340*	
Gateways-69B-1	2	24.5 kb downstream of *ENSDARG00000039947*	
Gateways-69B-2	6	190.1 kb upstream of *zgc:55448*	Looper-N8_DR
Gateways-70	Un	no tagged genes	TDR3
Gateways-71A	2	29.2 kb upstream of *zgc:85851*	
Gateways-71B [c]	ND	ND	
Gateways-71D [c]	ND	ND	
Gateways-71E	15	19.2 kb downstream of *mtnr1b*	TDR14
Gateways-72	18	intron of *bckdha*	HATN6_DR
Gateways-73	6	0.3 kb upstream of *LOC100002374*	
Gateways-74	24	intron of *zgc:101685*	
Gateways-75B	8	13.8 kb upstream of *LOC793664*	
Gateways-76	18	43.3 kb upstream of *cdon*	TDR13
Gateways-77	Un	no tagged genes	
Gateways-78	19	intron of *ENSDARG00000033473*	
Gateways-79A	Un	no tagged genes	
Gateways-79B	17	intron of *ENSDARG00000027564*	
Gateways-79C	3	85.6 kb upstream of *ENSDARESTG00000005413*	
Gateways-79D [e]	11, 12	ND	
Gateways-80A	3	intron of *LOC557716*	TC1DR3
Gateways-80E	24	8.6 kb downstream of *zgc:122991*	
Gateways-81A-1	21	14.2 kb downstream of *ENSDARG00000070026*	HARBINGERN7_DR
Gateways-81A-2	21	intron of *zgc:101028*	Dr000727
Gateways-81B	2	59.4 kb upstream of *klf4*	
Gateways-81D	2	21.1 kb downstream of *glula*	
Gateways-82	14	51.0 kb downstream of *zgc:100881*	DNA25TWA1_DR
Gateways-83A	5	intron of *zgc:114185*	ANGEL
Gateways-84A [c]	ND	ND	TDR8
Gateways-84B	Un	no tagged genes	
Gateways-85	25	intron of *sae2*	
Gateways-86A	8	208.7 kb upstream of *mecp2*	
Gateways-86B	25	0.7 kb upstream of *glcea*	Dr000284

Insertion name	Chromosome	Integration locus	Repetitive element [a]
Gateways-86C	10	intron of *zgc:112138*	
Gateways-86D	24	intron of *ENSDARG00000027824*	DNA9TA1_DR
Gateways-86E	7	intron of *ENSDARG00000009170*	
Gateways-87	3	13.1 kb upstream of *ENSDARG00000026313*	Gypsy-29-I_DR
Gateways-88A	25	intron of *ENSDARG00000062937*	
Gateways-88B	1	14.8 kb downstream of *hel_dr1*	HE1_DR1
Gateways-89	25	0.1 kb upstream of *rhcg*	
Gateways-90B	Un	no tagged genes	Dr000107
Gateways-90C	17	intron of *slc8a1b*	
Gateways-90D [e]	ND	ND	DNA-2-4_DR

[a] Targeted repetitive elements. [b] Insertion that was remobilized *in vivo* (donor insertion). [c] Insertion is matched to database with maximum identity less than 95%, or no significant similarity is found. [d] Insertion with short (<100 bp) dinucleotide repetitive sequence read. [e] Insertion is matched to more then one genomic locus with similar identity (multiple hits). ND, not determined; Un, contig not assigned to any chromosome; n, number of insertions.
