# Supplementary material for: Genome-wide analysis of Tol2 transposon reintegration in zebrafish
Source: BMC Genomics. 2009 Sep 8;10:418. doi: 10.1186/1471-2164-10-418 (PMC2753552; doi:10.1186/1471-2164-10-418)
Supplement: Additional file 4 — Flanking genomic sequences of Tol2 transposon insertions. Table S3 shows the DNA sequence reads of the regions that flank Tol2 transposon insertions. To obtain the genomic locations of Tol2 insertions, the flanking sequence reads were blasted against the latest zebrafish genome sequence database (zebrafish whole genome assembly, version 7) from the Ensembl genome browser . In some cases, the flanking sequence reads were blasted against the unfinished high-throughput genomic sequence (htgs) database or trace archive at NCBI . [file 1471-2164-10-418-S4.pdf]

Table S3 - Flanking genomic sequences of *Tol2* transposon insertions

Insertion name	Flanking sequence reads from 5' and 3' transposon ends
ET33	5:**TACATGGG**AAGCAACTCACGACTGTCATCCGCCAATGCTTTTTACACGACCGAATTACAGACATTACAAATCACTGTTACAGTGTTTGGGTGAAAAAGTGTCCACGGCGCTATGGCAGGTGTACCAGCCATTTACCAGACTT<br>TTTTGGAAAGGTGAGTTTGGCGCAAAAGTTCTATGGCTGGGTGCGAACGTATGGCTTGGTTGGTTGCATGAGTACTGAGCTGACTCGGGTCTGTAACTGCCCCGTTGTAGCAACAGAGGGTCCAAAAGGGACTGTGGCCTGTGC<br>GCCGTATAGTCCAACGTGTTTTCAAAGGTGTGATGGAAACGTGACCTCACAGCAGACTCGTTTCGCGTCTCGGGAGACAGAGTCTCGTGAGAGGGGGTCGTCATCGTTCGCGTCACTGTGGTTGAAGCGGGTGTGTCTTGATGT<br>TCTGTCACCTCAGGAGACCTGGCCTCTACAAGGTGCTCGTCGTCCTCGCCGATTTTGGCAATGTAGGCCTTGCAATGGAGTTTCATGTGTTTGCGCAAGGAGCTGGGATGTGTGTAACATTTTTCACAGCCTCTGACCTTGCAC<br>GTGTAGGGTTTATCGCTGGAGTGAACGTGAGAATGCTTCTTCCGGTCACTGCTGTTTGCGAATCTTCGATTGCAGCCCTCAAACTCACATTTAAACGGTTTCTCACCTAAACGGGCAGAGGAGGGGGAAACGCGAGTTACGGGT<br>GGGATGTACAGTCAACCATGGCTCATCAGTTCTGTGCATGGTGTCATTAGATGTGTTATGCAGGATGTTGTACCAAAAAAGTGTGCCGCTCCACACATTAAACAGTAAAGCAGGCGAGAACATCACTTTGTTCAGATAACACTC<br>ATGTGCATGCAAAATGTGATAATTTTCTTTT<br><br>3:**CCCATGTA**AAACTGTCATAAAGGCTTTTCTGAGTAGAATAGAGCAATGTGATATATACATGTGTGTCTGTGTGTGTGTATGTATGCATATATACACACACAATACATTCATTCAGTGAGACGTCGGTTATTATTTCTGAAAC<br>TGTAAATAGTTTCCATAACCTTGCAAAAATAGTAGTTGACAAAATATAAATTATATATCAAATATAAAACAAATCTACAAATAAGACCCGGCAGATGAAGTAAAAAATATATAAGTATTATAAATAGATCGAAATAATTATATT<br>ATTTCTGCACAAGAAAACAAAAATTACTGGCGTATTATTTTGAGTTTCCGTAAAGGCAGGGCCTCCGTTTCTGATGTAAAGTAGAAAAAGAGGTTTATCTCTTTAAAATTAATTAATTAAGGTAATAATACGTGAAATCTTTTG<br>TAAAGTACAACTTTTACATGTGTTTTCTGTCACAGCATAAATTTAGGTCGAGGGTTCTTATAATTAAGGTTCTCGTTTGTCTTGATGTGTGTATGTGAAACGAACCCATCAGTGTACGAGGCCTTTAGCTACAAATTATTTTAA<br>GATTTTCTCATCACATTACAATTAAGTTTATAAGTTGATAGG
ET33-1A	5:**CTGGGCCT**GAACCCGCTCAAAGCCTTCTGTGTCTGCTGCAAGGTGCAAACACGCGCACACACACTGAACTTCATTTTGACGC
ET33-1B	5:**GAAATGAG**GGAAACTCTGGGTTCAAGTCTTACATCAAGTCGATTTCTAAAAGGGAGGTAAAATTCTCAGAGTGGGTTACCATGACAACTTATAATGAACCATGATAACATGATGAACCTAACCTGGTCAGAAGCATATTTTC<br>CAACCCAGGCTTATTTTGAAAACGTACCCCCACTTTTTTTCTGGAGAGAGCAAAATACTTCCCAGGAGCTAAGTTTTTTTTTTTTTTTT<br><br>3:**CTCATTTC**AGGGAAACCAGTTCAGAGTTTTTACTTAACCTCTTAGCAAAAGAATGACCTGAATTTGAAGATCAGTTTTTCCATTACTTTTCCATTTCTCCCACTATAATCCAACTTAAAATCAAAAATCCTTTCCTCATTTA<br>GTATTGTCATATAGGAGTGTATAAGATTGTATTGAGTGTGTATGTGTTTGTACACATATTGATGGTTTTATCATTGAGCCTTCAGCAGAATGTTGTCTGTAAACAGACTGATCTCTGTGGAAATTATCAAGCAGGGAAGTCTGA<br>ATCTGAGCCATGCTTCTGGTACCATAGTTTGCTTACCACTAAAAAGTCCACAATTCATAAAAGGAAGTGCTGGTGTGTTGCCAGACAGTGTATTATTGA
ET33-2*	5:**ATAGATAG**ATAGATAGATAGATAGATAGATAGATAGATAGATAGATAGATAGATGatagatagatagatagatagatagatagatagatagacagacagacagacagacagacagacagacagacagacagacaGACAGACA<br>GATCAATATGAATCATTTGTAAAGTGTGTAATCCAGCTGTGGTAATAACATCTGATGTTCCTCTAGGCAGTGGTGTGAATGTCTACTCTCTCCATCCTGGTGTGGTTCAGTCAGAGCTGTTCAGAAACCTCAGCAAACCTGCGC<br>AGATCGCATTCAAGGTCTTCAGCCCCTTCACCAAGACCACCAGTCAGGGAGCACAGACCACCATCTACTGCGCCATACAGCCCGAACTGGACAGAGAGAGCGGAGGATATTACAGGTACCGAACATGGTATTATTGGAAAATAC<br>ACTGCACAATGCACTACCCATTATTGACTTTACTCTCATGTCCCAGTGTGCACAAGAAATACAGCTCTTGAACTGCTTTATAATGATCATGAAGTGTGCTTACTTTAAAGATAGAATA<br><br>3:**CTATCTAT**CTATCTATCTATCTATCTATCATCTATCTATCTATCTATCTATCTATCTATCTATCTATCTATCTATCATCTATCTATCTATCTATCTATCTATCTATCTATCTATCTATCTATCTATCTATCTATCTATCTAT<br>CTATCTATCTATCATCTATCTATCTATCTATCTATCTATCTATCTATCTATCTATCTATCTATCTATCTATCTATCAACCATCTATCTATCTATCTATCTATCTATCTATCTATCTATCAACCATCTATCTATCTATCTATCTATCTA<br>TCTATCTATCTATCTATCTATCTATCTATCTATCTATCTATCTGTGTGTGTCTGTCTGACTGAGAAGGATGTGTGTCTGTTTGTCAGTCCGTCCATCCATCAATGAATGACTGACT
ET33-3	5:**GTTTTAGG**TAAAAGAAATCCATCCTGTAGAGAGAAAACTCAAATTAGGAAAGACAAAGAAATAAAAAAAAGTGTAATAAAAATATTGTTTCCAAATGAACATGCACTTTTTAAAATGCACTTTAGTGTGTTTCTTATGTACT<br>AAAAGTATATTAAAACCGGACTTTTGCACCTCTATGAGTTTCTGGAGTGTAATCCCTTTGCAAAGGATGCTGGTCCTCCACTTTTTGTTTTTCTCCTTTCTCCCAAACCTCTCAAACTCAGTGGGCGTAAACCAGCGGTCTTCA<br>CTTAAGATGCACTTTTGCCCTAAGGACAGAATTTTGATCTTGAGACAAAATCACTTCGACAACATGCACTGTAGCCCTTTATATTTCTAAAGTCTTTAAGTTTCCTATATCTAGAAATGTTTATGCACCTACTTTTATAATATT<br>TCTTTAAATCCAGGAACCCCTCTTTATATCCACACGTAACAGGAAGTCGGGATTGACTTGAAGACGATTCTGAAATATGTCGAACCATATCAGTTAGATTGAGGAGACTTCCCAAAATCACACCAAACGAACATTTCTTACTTG<br>ATTCAGTACTGGTTTGCCTTATTTTTGTGTAAAAATGCTTCAAACTCACGGAGGCAGTTTTTGCTTTTAGCTTGTTTCTTTCTCTTGGAGCTGCAGATGGAGGACGACCTGATCTGGCGTGATTTCTTGCAGCTTTTTTTCTTC<br>ATCCTTCTTCACCCGGTTGGTTGTGATTGTTGTTTCTGCTGTTCTCACGGAGTCGAGCAGATTCCAGAAAGGGTTGAAGAGTCATTTCATGAATGAGGCCGCTGTATTATCTGCATTTTTTGAAGAGCGGGAAGTGGATGAGTT<br><br>3:**CCTAAAAC**CTTTATGAAAGGCAGGATTCATGGTGAAAAGGTTTGGCTATTGTTTTGGCTGCATTTGAGTGTTTTTCTAGTTATGAAGTTTAAGATTGTCTAAGATGTTTTTCAAACATTTTCCTTTTGTGTTCTGCAGCAGA<br>AGAAAAGGCCAGAGGTTTTGCAGCAGCAGGGTGCGTAGATTAAAACCAAAAATATTTGTTCAAATAAAATCCTAACCTTACATGAATGTCACGTTAGCTTCCAGGGGTGTAACGGATCACAGTTTATCCGTACGGATCACAATT<br>CACGGTTCTGAATGCACATGACCCATGGATTAATAACTTTTTTGAACTTGTACGTTAAGCCAACATTTATAACAATTGCAGAGAGATTGCCTCCCGTGTCATTCAAATCATGTGAATGCATTTCGGCTTCCCTGTAAATAATGA<br>TGACGGAAAAAAGTTGTGGGACATACCTAAACGCTTTTTTAGTTTATTAATTAAAAGTGTTTTCTGTTAGAGTTTGTTTAGCCTACATTTATGCATTCAGTGTAAAGCTGCACAATTAAGCTTTAAAATATCGTGATCTATATT<br>CAAACCTGCAAAACATGAATGATAAATGATAATGATTTGCATGTTTATTAATCCTTTGAAGCGCTGCATTCAAATCCACGTTTCATCGAGAGAGAATTTTTACACTTTTTCAGTTAGTTACAAACAATTAAATAACACAGAAGA<br>ACTGGGAGAACAGTTTATAGTTCAGCTATAAATCCTGATGTTTATGGTAAAGATTAAAATCTCAGTTTAATTGAACATGACGCTGGTGAAAGCAATTACATTATGTAACCAACACCATCAAAATTATGTCACTGAATGGG
ET33-9	3:**ATATATAT**ATATATATATATACTCTGAGAATG
ET33-10	3:**CTTTAAAC**TATATCATGTTTAGTTCCTACCCTGCTCCAACATACCTGTATGTTTAAAAAAAAAAGCCTAAAGGACTCAATTATTTAGATCAGGTGTGTTTAATCAGGGTTAAAGCTAAACTCTGCAAAGCTGCCCCCCTCAA<br>GGAACTGAGTTTGGCACCCCTGCACTATATGGAAGGTAGCATCTGAAAATGCATAAGGCACTTTGACTTTGTCTATTTTAATCATTATAAAAAAATATAGCATGTTTCAATGCAAAATACACAGTAGTACATGAAAGGCGACAC<br>GCAAAACTTCTTCCAGAACAAACA
ET33-14	5:**GTGTGAGA**TTGTTAAAACAGCCATGCTTTTCAAATCTGATCAAAAAAAGCATTGTCTTTTGACAACAGTCTGTCAAGTTTATCTAAGAGTATTTTGATGAGAAAGCGAACGGCTGAAAATGGGGCAGAATATGATGCAATAA<br>TCTCTACTGTTTTTTTCATAATTGTTTGATAATTGATTGATTCATTAATTATTTGTTTAATAACACTGTATTACTGACAAGCAAAATATAACTTATTGCTTAGAAAAACATGTACTTTGAAACCTGCTCAAAACCCAGTTGTGT<br>TTGTTTTTGGTACCTTTTTGAAAATCTCATTGTTTCC<br><br>3:**TCTCACAC**ACACACACACACATTTGTTGGTATTGGTGGTTTATGAGGACTCCATTACTCCATAGGCACTTATTATAAGCCTTTACGCTACCCCTAACCCCAACCCTCACAGGAAACAAATTTTGTATTTCAAAAGACCCTGT<br>TAAATATGATTTTTAAGAGTTTTGATTTACAAGGACACAGGTCATGTCCTCATAAACCACCTTAAGTTGTATTACCTAGGTCATATCTTTGTCATTATTCAAATTTGTGTCCTCGTAATCCACCAAAACCAATACACACACACA<br>CATTCTCCCACAGAAAACAAATTTTGAATGTCAAAGGACCCAGTTAAATATGATTTTTAAGCATTTTTAATTGCGAGGACACAAGTCATGTTCTCATAAACCACCTTACTTTGTAATACCTAGGTCATATCACTATCATTATTC<br>AAATTTGTGTCCCCGAAAACCACCCAAACCAGTACACACACACACACACACACACACACACAAAATATATGTGTGCGCACAAACGAGCTGTCATC

ET33-15	5:**CCAAACAG**GTTGCGAATTACATGGGGAGTTTGGGAAGTATTAACCCTCTAATTAAGGCTGGATCCCTCTTGAAGGACATCAAAACAAGATGTATTGATGGGTCAGCGTTTTAAAACTGAAAGTTCACTCTGATCTGTATTTT<br>AAGTAATAATGTTAATAATTAAAATAATAGACATCCATTTGACCACCCCTATAAAGGTTCATACCATTGTGAATGCATAACTGTGCCCGTCAGTCTACGCGAGAAAAAAAGTCATTCAACAGTATGAAACTGGCAGATTCAGAG<br>CACCGATAGACACCGTAAGAGTTCGCAAGGCTGTTTTTCTTATAAAATAGGGGTATGTTTCTACATGTAGCCTAAAAGTAAACTCAAATTAGATGCATAGTTTGTAAAGTTTGACCTAAGATAAGTTGAAATGGCTAGCCTGTC<br>AGAATACACTAAACAACCTATCCAATAAAACACTGAAAACTGAAATACACTTATTTATAATTCAATATAATATATACATTTTTTGAATAAATTAGGGGGTAATTTAAATAAAATACGTGAATTTGATCCACTGAAGCATGTATT<br>ACACAAACCATTACTGATAA<br>3:**CTGTTTGG**TCAGAAATCTTATGCAAGTATGACTTCAAAACAACATCAGCTCTATTTAATTGACTGTGAAAATTGTTTTGCTGTGATGGTTATTGGCTGCTAATATGATTACACCATTTGAATTGGCAAAGAATACTATTTTG<br>AAGTAATATTATTAAGCATGAAGTCTGATTGAGCGAATATATAAAAACTTTGCCTCAATAAGGTTAGGCTGTTATATTTCACTTAATTGTATTTAAATGACACTGTATGGCTTTTGTGCGTTCCATATAAATATTTAGTTAAAA<br>CATGAGTATAGTGCACTTAAAAAAATAAGAATATTTCTAATAAACAATGCAGAACATTAGATTAAAAGCCTCAAAGCTTCGTTTTTTTGACCAGTGTTTGATACAGACGAAATCAGAAGATCAAGAAACCTCATATGTGACTAAA<br>AATTAGAATAAATGCATATACTGCTTTTAAATTCGCTTTAATCCTCACTCGTATTAAAGCTAGCACGATTGCTAATGATTACAGGTGGTTACAATGAAATCTGACAGTCAGCGCTAAACGGAGGAAATCTGTTAACTTGGCGTC<br>AATAATTAGCGACTAAAATTTATGTTAATAAATGAAACTGGACTTATAATGTAACGTTACTAATAAACTCTCTCTCTCTTACCTACTATTCAATGCCAGCTCACTGTTTTTCGGATTATTGACGCAGGTTGATGACGTA
ET33-18*	5:**ATGATGCA**AACAGTCAGCATTAAAAATGAACAGCTCAACTAGATTAATCTAGACAACTAGATTATATTCAGCTCTGTTTAGCTGTCATTAAGTTCAGTTAAAATATGTACAGATCATTAATTATGAAGCAGACTTAGTTGAT<br>CTACAAGCAGTTATAACTACTTGTTTTTTTCCAATAACACTAGTGTAGTTCATTCATTTGTTCGTTCGTTCGTTCGTTCGTTCATTCATTCATTCATtcaatcatttattcattcattctttcatAAATCTTTGGCCTAGTCCC<br>TTTCACTAGTGTAGTTACTATTTAGAAATCAATCTAATTTCTGCATATTAGGCATATTTAAGCCACAACTACGCAAGCCAAAGGCAACAATAGAACCTAAACTCAAATCGGGTGACAGAAATGAAGAAATGTTCTGAATGAAT<br>ACTCATATTTTCTTAAGCATAGGAGGAACTTGAGTCTTAAGCAAGGTTTCCTGTGCATCCCGCTGTGAAGTGATTAAGTATCAGAGATGAAGAGGTCTTTGCAGTGTGTCTGCATTCACCGCATGAATCTGTGTGTGTGTGT<br>TTTGGGGGAAATGCTCTTTCTCCCTTTGGGCTAAATGACACCTGGCATTCCCATTACGGCTCTGAAAAACAAACATACGGCTCTGGTGTACAACAAGCATATCTCCGCACGTGCACGCTCACGCTCACGCACACACACACTGTA<br>AGAGTGGAGTGTTGAAAGAAATGGGTGTTGACGTTCTCAATGAACACACTGTGGTCCTCAGAGACAAACCCTGAAAGACATGTTTATGACATCCTGTCTTGTTCACCCTGTTTCATACTACAGATCTCTCCCTTTT<br>3:**TGCATCAT**TGATTCTTGTATTTTGTCACAGTAAACCTGCTTTGTAAATGATAGAAAGTCGTATGTTTGCGAGCTAACACCAGAGGGCAGTGTTTCATGAATTATTATAGTTCAAGTGTACACAAATCTGTTCCTGGAGGACT<br>GTTGTCCTGCAGAGTTATGGAGAGCTGTATGTATCGCTGATTGCTGTAGCCCTCCACAACCTGTTAATTAGCATCTTTATAGTTACTCAAAATTCCCAGATAAGTGTGTTGGAACAGGTTTAAGTTAAATTAGGACAACCTTGT<br>TTAGTGGATGCTCAACAGACTTCTTTAGGTGATGAATCTCATGGCCCTGTCTCAAATCCAACACCAAAGCTCTCACAGTTTTCATTCAACATTTACATGCACTGATGTCAAATGAGAGGTCCAATGAGAAGTCTTCTGAAATCT<br>AAAACTATGATTGAGAGACACTTCCATATTGCCCTTCCAGATGTAATGGACATGCATGCAGTATGTAGTTGTGATTGTAAATCCACTAGGCCACATGTAGTCATGACKTGAATTGCTGTTGCAGAACTGTGGCCTTTCAGGAGC<br>AAGAGTGGAGACTTTTTTATTGTAGTGGATGCCCAAAAGAGTCCAGATGTTGGTGATGTAAATCCTCAGCGGTCTAATATCACCATATTGGCACCCTAACTGCTTTGACTGTTGGATTTTCTCTCAGTCTCTGTAGTATAATCA<br>ATCTTCTGAAAGCTTAATGGGTCTAATCTTGGAAATTTTCCTCACCAGTAGGGGGCACGTTACTTTACAATTCTCTTCTCACCATGTACTGCCTTTGTCTGATAGCAGCTGACTTATCACCTCATTGCCCTGTGAGGCAGTCAC<br>TTTGC
ET33-24	5:**ACTGTCTG**TGATTTCACCAGGGACAGCAAGAGTTTGCTGCAGGTTTTATCGCAGCTATCTTACCTCACTCAGCCCAGAGCAGAAATGTTAAATACTCTGACACCTGAGTTCTCCAGTGCGGAGGCATTTGCAGATGTTGCAG<br>GGCTCTGGTCCTCAGAGAAGGAGCCCACTATCACATTCGAGGCTGGCGGCTGGTGAACAGAAGCTGTACAAGGGCAGCATGTGGCCCTGAGGTAGAGCAGACGATCACAGAGCTACCGCTGTTCATATCGCTCAGTAAATAGAG<br>CATGACTGACTTTCTGGACAGAATTCAACGCAGAAACACAATAAGTGGCTTATAAACATGAAAAATTTAACTGTCTGCAGCTGGGAAAGCCAAAGAGTGATGTCATGGGATTAAGTTAGTCTGGTGTTTACTGCCTTGCTGTAA<br>ACCGTCCGTATCATCCTTATGACTGAGCTAAAAACATTATTTTCTTTGTTGGCTTGATCAAAGAAGTGTCAACTTGGCCATTCCATACATTGCTTTAGGTTTCCAGGGACGTCTGTGGCAATAATATCTCTGTAGGGCAGAGCA<br>TATTCAAATTGTGCATTTCTTGCCAATGTATTGAAGGGATAGTTTACCAAAATGTTAAATTT<br>3:**CAGACAGT**CGTTTCCAGGTCACATTCTTGCCTGCTGGGTTTGGTTCATTGCACATTTTAGTGAGGGAAATGGGTAGGATAATTATAAAAGCATAAGTGTTGGACTAAAAAGATGAGGATTGCCTGAGTTGAATTTTCACTTG<br>AGTGGAAGCAGTAATCTTTACTGGAGATCATTTGAAATGCATGCGACACCTCATAGCAAGTGTGGGCAATTCATTTTCCCAAGGGGCCACATTAGACTCTGGGGTGGTTTTAGAGGGCCAGACCAATACAGTTAATTGTTTAAA<br>AGAAATAAAAAATAAAAAAAATAATAATAACAATAATTATAATAAACATTCTGTGAAATTACTAAATAAACATTTATAAAAAGTATACAGTTTAAAAAAAAGAATAAATTTTACaCAAAACGTATCTTAAAGACCAGCATC
ET33-A1	5:**CAAACCAA**CGCACACGCACACACACACAAACACGCACACACACACACACACAAAACACACCAATACTCATTGTTCAAGGTAATCCAGGTAATGCATCTCAAGATTCTTTAAGGGTGTGTGTGTGTGTGTGTGTGTGTGTG<br>TGTGTGTGTGTGTGTGTGTGTGTG<br>3:**TTGGTTTG**TGTGTGTGTGTGTGTGTGTGTGTGTGTCATCACGCACTATCTCCAGGATAAGGTGTGTGTGGGTCTGGACCAGGCNCTGCNNGTTTNCAATGTGAANGAGNGTGTGGAGGGCCCGAGCTGCTCGTTCCTGCAGCAC<br>ATCCAGGNCGAGACCGGGGANAANGTCTTCCTGCGGGAGAAAGGCTCCGNGTGNCTGGACCCCGCGTCNNNGAGGGAGGCCTTCGAGCCNNTGTACATCTACATCAGGNNTGTGTGTGTAGATCACACA
ET33-B13	5:**GATGTAAA**TGAGAAATGTTGGCGCTGCTCATGAGTGCCCTTAATTTGCACTCTTCAGTCATTTGTCGTGTGGAAATAGACATGACCAGGTTGTACATACGAGATGCAGAATCAGACACTCAAGACTTACGCATGCAGATTTA<br>TTGTAAGGGGAAGAAAGACCTATTTGTGACTTGTGTAAGAATATTTTGACGATAAAACATCTTTAAATAGAATGTGTATTTTTAAATGATATTAGAGAACATTTTTTATTCAGGAAATATTT<br>3:**TTTACATC**CTTTATTATCTCTGCCAGCAGCAAACTGGCACAACATCTGCTCCTCTCCATATGGAGCTTACTAAACAAGCTGAGAGGAAAAGAGGCTAACATGTCAAAAAAAAAAAGCTAGGGCTAACCCAAAACTATCAAAT<br>ACGCAGGGGTATATACTTGTACGGACCCCGGGAGGGGATGCA
ET33-D10	3:**CTTTAAAC**TATATCATGTTTAGTTCCTACCCTGCTCCAACATACCTGTATGTTTAAAAAAAAAAGCCTAAAGGACTCAATTATTTAGATCAGGTGTGTTTAATCAGGGTTAAAGCTAAACTCTGCAAAGCTGCCCCCCTCAA<br>GGAACTGAGTTTGGCACCCCTGCACTATATGGAAGGTAGCATCTGAAAATGCATAAGGCACTTTGACTTTGTCTATTTTAATCATTATAAAAAAATATAGCATGTTTCAATGCAAAATACACAGTAGTACATGAAAGGCGACAC<br>GCAAAACTTCTTCCAGAACAAACA
ET33-E20	3:**CTGTAGAG**GTTCTGCAGGATGAGGCAGATTAGGGGAATTCTTAGTTCTGATTCAGAGTCTGAAATATCTGATGCTGATTATCTAGATTCTAGACTACGTGTCACAGGGTCAATCTGGAATTGCTGGACTGAGTAGACAACTA<br>GGGTATTACAGGAGCAAGCATTACCCTACTCAATGTAGAGCAAGAAGCCACAAGGCACATGCAATGGTACAATATACTGGATGTCACCACCCTAAACTTTGCCCAAACACCCTAATTACATAGGTGGTGTGACCCATGTATGTA<br>AACTTGATGTTGTAATACAAACCTAAGAAAAAATAAATAATTCATTTGCGCTACATGAAAAAAATAATTTTTGAAAATTGATGAATAATTAAATTCATGGAATAATTAATATATATAATGCTGAAATAAATTGCTTTCAAAAGATA<br>AAGCCAAAAA
ET33-F38A	5:**CCTGACGC**GAATACAAAGAGCTGCACTAAATGTTTTTTATGGTAAGCGCAGACCCGCGGTTTGTCACATTTGATTTCAAAAAGGTTTGTTAAATACATTAACATTATGAATTTGGTTATGAAAAAAACTATGCACTTATAAC<br>CCTCTCACAACGAAAAAACTTGTATCT<br>3:**GCGTCAGG**GCATTTTAGGCAACTGTACAATGTGGCAGTAGGACCACCTGTACAGTATGCCTGGCACTGAACTGTTTTTTACACTTTTGTACAGTTCCTTGGTCATAAATCACTGCGTATCATTACACAGATGGTTCAGAGCA<br>TGGGAATAGCATAGTGGACAGTGCGTCGATAAAGAACAACGGAGCGCACAACGGAGCGAGTTCGAATCTCATTTATAGATCTTGTGTTTTTTCTCTCCTTCTGCAACCTCACATTTTTCTGTATAATATTATAAGTGGCAGCTT<br>TGTCTTTTGTAGGAAAATATATCTTTAATTTAAGATGTAGGACGTCAGTCTTATATATATATTCACGCACACACACCTACATTTTTTAATATAATATATTTTTTCAGGGTTTCTAAATGCTATTGTGTGCATTAATTGCAGCCGA<br>TTTTAAAGCTCCATCAGTCACTAAAGGACACTGATAATATAAAATTTGTCCAAAGTATAAGTGTGCAGGTACAACTTAATTTTCTATCCTTAATCTTTTGGTGTTTCAACTATTAAACTCATTACTGCTTCCACTTACCAGCTC<br>ATCACACACCCCACCTCATCACTATATGTTAGGGGTTAGGGTTAGAAGTAATGATTCAAGTGTATAACACAAATAGTGTAAAAAAAAAAAAAAAAAA
ET33-H8	3:**AAATTTGC**CATCAGCCCAACACAGTTTGGATTATCATAGGACTGTCAGAGGTTTGCCAAACCTGTCAAATGCATGATGGCTCCTGTTTGGACTTACCGATGTCCTCGACTTCAAGTATCCATCAAAACTGTATACATTTGAG

	ATATTTTAGAGACTCTTTGTGGGACTGGACATTATGCATTCTAAATCTTCATCAAAATATGCAAATCTCCTCACTGAGTTGCCTTGGATGATTTCTCAATAGGATCTGGTATTATTAATGTTTTTATTTATGTATTGTGTATGT<br>GTAGCCACATTAATGGCCTATTTTGTAGTCACTATTAAAGGAACATCGATTGATATTGAATACATGTTATGGTCTTTAATCCATCTTCCCATTCTTATTTAATTGTTATTGACAGGAAAGTCTGTTTATCTGTTTTTACTAGT<br>GTATATTGTGCTTACAATATAGAAACGAAGATTTATTTTCTGAGGTTACAGCCAGAAGTCAATTAAATTAGTTTTAAATGACATATGCATGGTTAATTTTATTTAGATCATTAAGTTCATAATACTTAATTAAAATGAGTACAG<br>TCAACATATAGCAATGTATAAGTAAGTTAAGTTTAACAAAAAAAG
ET33-H23	5:**GATCCAAG**GTTTGTAAATTTCCATTCAAATTTGTCTAGTGATTTTAGCCAAAGCAATTGAATTGCGCTGATGCCCAGAACAAACACCTG<br>3:**CTTGGATC**CTCTCTCATTAAAGATGATCTCTACATCTAGAATCTTGCCAAATTGCTGAAATAACAAAAAAAAAAAAAAAAA
ET33-J1	5:**ATAAAACA**CAACAGGTTACTGTGCGCACTGTCGCCTCACAGCAAGGAGGTCACTGGTTTGAGTCCCGGCTGGGTCACTTGACATCTCATTGTGGAGTTTGCATGTTCTCCCTGCGTTTGATACAATTAAATGAACTATATTG<br>GCCGTAGTGTATGAGTGTGTATGGGTGTTTCCCAGTGCTGGGTTGCAGCTGGAAGGGCATCAGCTGTGTAAAACATATTCTGGATAAGTTGGCAGTTCATTCCGCTGTGACAACCCCTGATGAATAAAGGGACTAAGCCAAAAG<br>AAAATGAATGAATGAAGGTTAATGCAGTGGTTTTCAATTCAGGTCTAACAGGTCTAGGCTTTCTAGAAACATCCATGCAGATGTGTTGAGGCAAGTTGAAGATACAATCTGCAGGACAACGGACATCCAGGACCGAGTTTGTGC<br>ACCAAAAAGGAGACAAGGAAAATGTGCTGGTCCTACAGAGCCACTGATTTAGGAGACAATGCTTCCCTCTTG<br>3:**TGTTTTAT**TAATGTCAACACCTACCCCAACCCTAAACCCAACCTCACAGTAACCTGTTGTGTTATAACCAACCCACCTGTGATATAACTCCCTCCCACTTGGAGGTCTCAAAAAATAGGTCTACCTATTGGGCAAGTCCTGA<br>AAAGAGCGTGGAAGAAAAAAGAGACGTGCAAAACGTGTAGTACTGGTGCTCCTCCAGGAACTTGAATATCGCTAATATCTGACATTAATCATAAATGCATGCGGGTCATTTGCACTCTTCACCATGTTAACATTTTTGCTGCAC<br>AGAGACCCGTCATCTGCGTGTGTCAGTCTGGAGATATTTATTAATCCAATTAAATACCGGGCTTTCTGCCGTAGTCAGAAGGGTAGTACACTGATAAAACAGAGATAATCTTCATTGCTCTGAGGCTGTAAAGCACTGATAGAC<br>ACGGAGGTGAAGAGTTGAACTTTTGGAAAATGATGATGTCAAGGTGAGGCCAGGGCAGTAAAATAAACAAGTGTGTTAAGGTGCAGTGCTTAGAGAAAGTCGTGAACGCGCTACCACACAAACCATTATTTTATGGGATTTATT<br>GGGTCAATAATATTTTCCTAAGCACTGATTTTGCTTACGTCAATGAACTTCAATTCATTGACAGTGTCGTGTCACCAACAAATCTTTCTTTAGAGGCGGATCTTTTCAACCCTCTGTTCAAACTATTTATTTAAAATGAGATGA<br>AACAACACAATTGTTGAGGTTTTTAGTGGGAAAACTTAAATATTTTATGTTCAAACCACTTCAATTTGTAAAAACTAATAAATTAACTTA
ET33-J12A	5:**CTCATAGG**TCAGATTCGCAGTCAATCACATTGTTTGACAGCGTGTAAACACTCATTATCGAAACTACATTTTGATGACTGACAAAGTGTTCATT
ET33-J12B*	5:**ACTATACT**TAACATTACTCATAACATCACTATATCTTTCCCATTGCAATGATTCTGATTGGTCCTCTTTGTGCATTCAGTGGGCTAAAATAAAAGCCTATACAACCAAGTTAACTGCTCTTTTTCTAGTTTCACTGTCTAAA<br>CTGGTCTCCTTTCTTCAGTCTCATTTTCAATCTTCTGTTAAACACAGTTCCCAGTACTGGGTTGCAGCTGGAAGGGCATCCACTGTGTAAAACATATGCTGGATAAGTAGGCGGTTCATTCTGCTGTGGCAACCCCTGATTGTT<br>AAAAAAAAACTAAGCTGAAGGAAAATGAATGAACACAGAAGAATGTTTGAAACCAATAACCATTACTTTTCAAGTATTTGTTTTTCCTAAAATGGTCACATTTGGAAAGGTTACAGGTTTCCAACATTCTTCAAAACATCTTTA<br>AAAATATCTTTTAAAAGCCTTATATTGTGCTAAAAAAAAAAA<br>3:**AGTATAGT**TCTCTGTTTTAAATTCATGCCACTAGAATTCCAGTTAAAATAAAGAATTTTTACATGAGAAAGTGACCAAATTACAGTTGTACAACAGCTGTCTAAACCAAAAGGAAATATAATGTGTTCATTTACCAACTATG<br>AGTTAGCATTAATCGTAACTTTGCCATATGTCAATACTGTTACTGTTGATGGAATCCAAAAAAAACTTTAAAGCTGAATATCTCAAAACTCAGGACTTTTTTAGATAAGAAATGTTCTTCTTTTTATAAAAATAGACAAAATTA<br>GGTTTGTCTTCACTGCTCTAgtactattgtcattaaaccgtggaaaccccttttgcaaatttgtccAAACTCTTCAGCTTGACCCTAGTCCTTCTGATTGAGTTCCCACACCGTACTCCCAGCTTGGTGCTGGATACTAGTTCTG<br>GTCAGCATATTGCATACCTGTATTTTCTGTATGTAAGATTGCGCTAG
ET33-K11	5:**CTATTAGA**TATAGACTTTCTAACGTTATGAAAGTTATAACGAGAGAGCGCCCATTGGCTATACAGTACACATGTAATTCTACTAGTCGCCACTAGAGAGCTCTGTGTCGCAGTCATGCGCGCTCCTCCCTCGTTGGCTTCAT<br>TCCCGCGGTTCAAATCAGATAAGATACAACATGTTTGCAGTGACTACTCGTCTAATTTGTCGGTATGATAGCGCCATGTGTCTGGTTAGTTTAAAAAAAATGACGGCTAGGGCTGGATAATGAAATAACAGCACCCTTTATCGT<br>CaGTTAAAAGAACAGTAGGCCTAAATTAAACTCTAAAGACTTTATTTACACTAAATACGACCTATTTTGGAGGACTTGCTGATGCAATGTAAGTGTAATAGGCTAATTCTGCATAAATTATTAACGTCTACTTTGTTTGTTTGT<br>GCTATTGTTTG<br>3:**TCTAATAG**CATGGCATATTTATATTTATATTTCATAACAACATATCTCTG
ET33-K15*	5:**TCCCAGAG**CCGCTGCTTGATCTCGAGTCCTTGCTTAAGGCTGTATCGGCTAGCGCGTCTCTTCTTGGTGGAGCCCATCACACTGCAGTAGATCTGCTGAAAGTCCTGCACACTCCGACCGTGGATGGAGAGGGGCTCCTCCT<br>GTGGACACTCACCCGCTGAGGAGACTGATGCTGCGGGACGGATGACAGGAAGAACAGCCGCGTTCTTCAGGTACCTCTCCTCCAGGGAAGTGTCCGACGAGCTGCAGGTGTCCGGCCTGGCCGCCGCTCCTGGCTCAGACGAAG<br>AGACGCGCTCCAGCAGGAAGTCCTTCAGGTGCCCGGGGCGCGTCCTGGTTCTTCTCGAGCGCCGCAGAGTCATCACGAACAACTTCAAACACTTCTCAAACTGCTAAAACTTTGCCAAGGGAGATTTGTGCAGAAGTTTGTAGC<br>GCGATGATcagcactgattacTTTATAGACAGTTGTGTGGATGTTTAAACCAATTCAAGTTGCCATGGTGACAGAATGACGAGACTCATTTAAATGTAAAACAATAATTGGATTACAGGCGTTAAGTCAAAATAGAGCAGCAAA<br>AGGCTGTGGTGTTTA<br>3:**CTCTGGGA**GCAAGCTGGACCGGCCTGCACTGGAGGAGACCGAGCAACCAGATGGACGTGTGATCATCACAGAGAGCAGGTCCGAGTCCAGCGTCGCTCCTAGATTCAGGGTGGACATCAGCAGAGAGCCACTGCCCAAGGAG<br>CCCCGGAGGAAGAAGCCCAGGCACTGAGCCATACTAGTGCCAGATGAAGGATCAAGGGCTCGTATGTACATACTGTATATATTGTATATAGTTATAGAATAAGTTTGCATGACATCAAATTTTACTTTTCATTCAAGTTTTATC<br>ACATTGCTGGATGTTTTGGTGACGGTCAGTGCTGCTGATATTCGGCTCTCAGGTTG
ET33-K23	5:**GTATTACC**ACACAAAAACAACAGAGAAACCACATACTTCATCCAATGGTAAATTCCTGTGAAATTTCACTGCAGAAAGTGCTGTAGTAATCCTGTCATATTTAGCTTTGCTCATTTTTTTGTGTGGTAAATTTATGTTGTAC<br>TGCTGAAATACCACAGGATTACCACAGGACATTTTTGTAAATCCGGTCGCACACCAGAAGCGAACTTAAGTCCAACTTTTATGCTACAGAAAAGCCCAACAGTTTGTAACGACATAAGGGCATGTGCTAGCTAGACAATATTTT<br>AAATGTCATCCTCTAGTATTAATTATCATAAAAATTCACATTTTCAAAAGAACTCCTCGTATTATAGTAAAACAGATAGTGAAGGGTTAACTAGTGAGTCTTTTCGTCATTTTATTTGATTTCTTTTTTTATTACTACAGGGTT<br>TGGAACAACATTGAGGA<br>3:**GGTAATAC**CAACACAGCTAAATATCACAGGATTGCTACAGCAATTTCTGCAGTACAATATCACAAGAATTAGCTTTTTGTTCTGTTTTTTTCTAAAACATTTTTTTTTTTCCCAGGGCAGGGAAAAATCAGTATGTTCTGC<br>ATTTAAGTCGTTTAATTAAAAAATACCTATTCTTACAAAGAAACATCCCATGTTTTCATATTTATTGAAAAAACTTTTCAGGGGGCAAATGTTTGGGTACTGAAATAAATTTGCAATTGAAACAACAAGATGACCGACCCCCTC<br>CATTACAAACATTACCTGCGGTATTCTATGGGGAAAACCATCAAATGTTATGGGAAAACTGGGTAGAACTTTGTTGTTTTTTGGGACGTTTTACCACATGATTCTGGGCTGAACAGAATGGGATACCCCTGTAGAATTCTGGGG<br>GAAGGGAATCACTTTCATATGGGGGAAGTATTTACCACAGTATTCTGTTATACAATATGAAATATTCCGGGGATATTCTTGGGGAAAAGCGGGGGGAATTAC
ET33-mi1A	3:**CCCACCAG**TTGAGAAACACTGGTCTATTGTGTAAAAAAAAAAAAAAAAAAAAAAAA
ET33-mi1B	3:**GTCGTGAG**CACAGTATTACAAGTCGAAATCGCATAAAATACGAGCATTCGAATCTCTTTGCTTGCACGCCAATTTCTTTTGCTTGTGCACAAAACTTATTGCACACCTCCTCAAATATACACCGCTCAATCGCGGATCTTCT<br>TGTGCGCTCTCAAATAAACGCTGCAACATTATATAATATAATGTAATTACCCCAGGGGTCACAATCAAATAGAACTAGTAACTATAGGCTTCTCTTCAGTAGGTTATGGATAAAATATGGTTATTCTTATGGTTTAATAAAAAT<br>AGATTTTTGGAAATCACTAAGCAACCCCCCTTCATCGTCCTGCGACCCCTCGGGGGGTCCCGACCACCACTTTGAGAACCACTGAGCTACATGACGCCTTAACATTTTGATGTCTGTTAAGTTGTTAATATCAAAATGAAAACG<br>GGCAGTTCCTTATATCATGTTTTAATTTTATTGGTAAAATATTAAAACAACGTAGTCAGGGTGATGTGAATGACTTTTCCCTTTGAAGATTTACCCAACGTATCCTCCGGTGTTTGTTTTCCATATCAAACTTGCGAAGTCTGA<br>ACTTACAAGGAAAGGATGTAGCTCAAATATCTGCAGCCACGTCTACGTTTTCAAATGTCTCCGTTTCCTCCCATCCACAGAGCAGCAGTATTTTAAATGAAAACAGCCTTTTCAATGTTTTCAAAACGCTCCATTTCTGGGGCT<br>CGAAAACTCTGGAGTTAGGTGGATGGAAGGCATAACTGTAGCAAAACTTATACATTTTAAAAGTAAAATGTATTAGTGTAAAAGGGGCCTC

ET33-mi1C	5:**ATCGAGAG**TAACGATTATTGCATCATATACCAAAAGCGCGATAGACCATGTGCTTGTGTGAGGGTGCAACACGCGCACATGCATAAGCACGCGGTCAGCATTCGGTCTCATTCGCACACTGAGACGAGCAGAGGAGCGCAAA<br>CATCTAAAATAATCCTAACATGAGCGCTTTAATGGTCAAATAGGTGTCAAAATGTGAATGCATGTTTAGTGATTATTCATATTAACCCTTATTTGCGTAATAAACAAACGAGTTGAGAATCAAAAGACACGTGAAAGAGAAACC<br>ATTAAAGTGACAGTGCGAAATGCTGCCGCCTGTTTTATCATTAATCAAAGGACGAGAACATCCCTCACTGCTCTTGACTGAAGGACTTTTGTAGCTAAAGTGCTTTTCTTAGGGTGAAGATGCTTAAAGCACAGTTTGTTTAAT<br>ATTTTTATTCTATTGTATTTATTTCTCTTTCCTTTGCATGGTGAAAAATAACTGTGTATATACATATATATATATACATATATATATCTCTAGTTGAAGTCAAAATTATTACCCCTCCAAAATATTAGCAACCCTTTTCCTCAA<br>ATTTATGTTTAA
ET33-mi2A*	5:**TTCATGAG**TGAATGTGAAActgccgaactgcAGTTAAAGTCACCCAAAATTACAGGAAACTCTTACATGAAAGCACTTCATGTAAACACCTTAAAGGTGCAGTAGGTGATCGTCTTCAAAAGCATGTTTTGTTGTGCTGGTT<br>GAAAGTCTCTTCATATTCCAATAGTAATGATTACAGTAAATGATCTAAATGTGTTATATGTATTTTTATATTTTGGGTACAGCACAACACTAAAAAGTGTTCATCCAATTAAATATTGTCAAACCGACATCTCCCATATTTCCA<br>ATAAGCAGC
ET33-mi2B	5:**AATATACA**TTGTAAAAGCGCTATACAAATAGAAGCTGAATTGAATTTAATTAAATTGTTCACTCCATTTAAATGGATTGGCATGAATG<br>3:**TGTATATT**GTGTCAAAGCAGCTTCACATAGAAGATCATAGTAAATTGAAACAGTGTCAGTTCAGTTTTCAGTGTCTAAGTTCAGTTTAGTTCAGTGTGGTTTAATAATCACTACTGAAAGTCCAAACACTGAAGAGCAAATC<br>CATCGATGCGCAGCTCCTCCGGTCCCGAACCATGCAAGACAGCGGCGAGTAAAAAAGTTCACCAATTGACGTAAGTGAGGAATTAAAAAGGCGCTGGAGAAGCTGGACATCAGCGAAGACTCGTCTGTCCCTGGAGCGTCACAG<br>GAATCAGACTCATGCTCTCT
ET33-mi3A	5:**AAATTAAT**GCAAATATATTCAAGCACAAACAGTATAAAGGCAAAGCACAAGAGCTCCTTGTTTCTCAGTTTATTATAGGCCAAGGAAATTCAGCTCTTTGCCAGTGAAAGTCAAGGAAACTTTTGGGAATTCGACATTTGGC<br>TAAAAGTGATTTTTTTAAAAAGAATTTTTCAAACTAAACTATTTCATCAACAGTTTAATTCCCGTCTGCCCAGTGGTTATGTGAAGAAAAGGTTGCTATTGTTTGTCTGGATGAAATATCTATAAAATGACTCACTGGTGGTCA<br>GTCTGAGTCATGATGGATAATGGAAACAGACCCACCTGTCCTCTACTAAAACTCTGAGGCCTGAAATAGCCCTGCCCAGAGCACCTGGCATCATCCACTAAAACAAACCCCTCTCATCTCT<br>3:**ATTAATTT**AATAGTCATTATGAGAGTAACAATGGGCAATTCACCTTAAATTTGGAAAAGAAATTAAGAAAAACAAAAAGAAAGAAGGGAAAGCGCCCAGTCTAGTTTTGTGCACCCAGTAGCCATTTTGTAGCCAAAGCATT<br>TCAAAATGTACATTTTCCAGGGATGTTTAAATATATTACAAAAAAGAACAAAAATAAGTGTAATAAAATTGTAATAATACACGTATACATGTTTTATCCATTAAGTGGAGCCAGTAGTGACTTGCAAATGATAAGTGGGAAGGA<br>AATAAAATGGCACTAAAAAATAGCCTAACAATGTAATGAAAAAACATGACACAGTATCCGCTTAAGTCATGACGTATCGGTGACATATGAAACACTGGGTCCAAGCTGCTACTGATTCAAATTGAGAAAATGTTTGTTTATGAT<br>CACTTTTCAGTCGTATCACACATTAAAGTGAATTTGAAATCAAATCATGGTGTACACAACAGTCTCTGGACTTGCTGCATCACAAACATCCATATTTTAACAATTTTTAAAGAAACGTATTTCTTTCTGGAGTTTGTATATTAA<br>GCATATATATTCCGGTCCTTATTATCATTATTTTTTAAGTATTACATCGCTTTGTAAACGTTTATTATTACCATTTGTTGAGTCTCCCTATACAGTTTTATAGAGCGCCTGGACGCAGCCGCTTC
ET33-mi3B	3:**CATACATG**TATAAATCTTAGTATTACTAATAAATTATGTTATCATTTGTGTTTTACAGCAAAGGATAAGAGTGAAAGGAACTTTGCGATGGCGTTTGTGCGTCTGATGAAGGAGGACGGAACCGTTCTGAGAGACGGCATAC<br>ATGATCTCACTGTATTTAAGGTCAGAAAAACATATGTACAAATATTTAATGAGGCCAAAAAGTGGATTAATTTTATTCTTGAGTTTTAATTGATTCTTGGGTGTCAATTACAAGCGCTGATGTGAGTAAATACTTGTTTCTAAT<br>GGGCTCTGTTTAGTTATTACCACCTATTTATGATTTAAATCAATATAGAAATTAATAGTTATGGACGATGATTTCTGCAAAGCCAAATTTTACATTGATGCAAATATATAAACATGATATGCATGTGATTGTGGGATTAATATA<br>TATTGTAAATACAAATTACAGAGCTTTTATTTGGCAGTTTCTGTAAAAATACTATTTTAAATGCAAAATATTTCATATTTAAATGTATTTAAATGTTCATATTTATTGAAGTATTCTTGTTTTAATTATAAAATCAATTTCAAT<br>ACAATATATAGAATTAGATACACAACAATCCTTTCTTGTGCAG
ET33-mi3C	5:**ACCCTTAA**TGAATTAAGAGACTAAGCCAAAAGTAAATGAATAAATGACAAAAACAAGATTATTCAGGTCAAAATGTTTATTTAAATTAACCAAAAGTTCACATTCAAATTACTTCATGTTAAGTTCTAAACCTCTCAAATTT<br>TGCACAGATGTGTGTTATAAGAACATTTTAAAATAATAGAATTTCTATTATTTTTCTTTTGATATGATCAGCAGTCTGAGTTTAGCTGTCCAGGGTGTGCTTGTCAAACAGGTACTCCGCCATCCTGTTATTTCCAGCATCCAT<br>CTTGGAGAGGTTAGTGATGTGGTCACCAAGCTTCTTGATGGCCTCAACCTGCTCATCCAGGTAGTGAGTCTCCAGGAAATCACACAGCTGATGGTCAAAACATCAAAAAATAATTTAGGATGACACCTTTCTAAAATAGTGATT<br>AAACTATTAAACCACTTTACAAAGTCACATGTAGGCTAATTAAGTGGTAACTCACATGAGGGTCTCCCATCTCAGTGGCGAGCTTATGCAGGTCCAGCAAAGCCTGGTTGACGTTCTTCTCCAGCTGAAAAGCACACTGCATAG<br>CAATCAGTCCATTTCCCCACACA
ET33-mi4	5:**ATGGATGT**TTCCCAGTAATGGGTTGCAGCTGGAAGGGCATCCGCTGCGTAAAAAAAATCAAATGCTGGATAAGTTGGCGGTTCATTCCGTTGTGGCGACTATAATAAATAATAAAGAATAATAAAGACACTAAGCCGAAAAG<br>AAAATGAATGAATGAATAAACTCAAAAATGTCCTGCAGCTGGTTGCATTAAAGTTTTAAGTTTAGTCAACTTTTTTTACAGTGTGTGAGTGTGATATAAAACACACATATTTCATGTCAAAACTAACTTTTATTTTATATGTGA<br>TTAATTCTGATTAATTTTTGCCCAGCACTATTCAAAAAATCTTTATTTTCAATAATTAGCTTCTATTTATGGGGGTTAAATCTGTTAAAGTTAAATCTGACTTTTATGCCAAATTTTTATTGCTTTTGAATAATTTTATTGCAA<br>ATTATTTAAATACTTTTATTCTCTCTCTCTCTCTCTCTCTCTCTCTCTCTCTCTCTCT<br>3:**ACATCCAT**ACACACTCAGTCACACACATACACTACTACTGACAATTTAGCTCATTCAATTCATCTATAGTGCATGTCTTTGGACTGTGGTGGAAACAGGAGCACCCAGAGGAAACCCACGAGAACATGGGGAGAACATGCAA<br>ACTCTACACAGAAATGCCAACTGGCCCAACCGGGACTCAAACCAGCGACCATCTTGCTTTGAGGCGACAGTGCACAGAGCCACCATGTTAAATTAAAAACAAAACTATACAAAACATTTTTACATAAATAAATAAATGTATTTA<br>TAATTTGTATTACATACATATATAATTATTTGAATAAATCATGTTGGACCAACTCAATTGCATTTAATTGTGTAAATCTTTTTTTTTT
ET33-mi5A	5:**CCTAAACA**CATGCTCAGAAATGGCGTAATTACTTCAAAATTAAATACTGAAAGACATTTATATTGGTCGACAACTAAACCCAATATTTAGGGAGGCTGCATTTAAGGGGTCTGCTCTTTGGTTAATACCAGCTCTTCCCACA<br>GGAGAGGGGAATGCAGCAGTCTCTCTTTGCTTTTAATTATGACAGACACTTGTTGCATATAAATTATCCTGGAGCAAGAGAAGCCATCTCTGATCATCAGGGTCATGACAAGGTGAAATAAAACACATCTGTAGTATTTTTTGT<br>CAGTCCCTCGCTCAAACACAAATTTGAGCATGTTGCCCAAGGACTTGATATTCATAGCAATGCTTAGCCGTGCAGCTGCAATCTGTGTTTGTGTATACGTGTTTGATTCCTGAAAGAAGCTTAAAGTTTTATTCTCTTATGGAA<br>AATAAGTGGGTGATTTTAAAAAGTAATACTAAAACCATTGCCTCAAATGTTAATGTAATTCCTTTGTATGAAGTAATTTTTAAGGAAGCACACAGCTATAGTTCCAGACAAATTTAACATGTTACAGTCGCATGATTTCAGTTA<br>TTTTAATGATTCTTACAGTTTACTACAGTGGCCGAGAGAGCTGCAATTTAAGAAAGCACTTGCAATTACAAAAAACAAATTAAGAAACGATCTTGATCAATTTAACAGCACACGTGGTACAAATTG<br>3:**TGTTTAGG**CTTGTCTTTCTTTCAAAAGACTCTGGAAGGACATAAATAATGAACAAGGATACATATGACAAAAATAGAGTGCCAGCTTTACATCCATCATTTACAACTTTTTTTTTTTTTT
ET33-mi5B	5:**CACATATC**ACTGAAAAAGACACATCATTGTGTTACTTAGGCCAGCTGTTAAAGTGGTCAGAAATGCTGACCCACCACTGAAATTTTATTTCCCTCCACTAGAGGGCCAGCTGAGTACAGTAGAGAGATGTTTACCTCGCAAT<br>AGCAATGGCTTTGAAATCCCTGTAGCCCTCAAATACAGCTTTGGAAATGGCAACTCTCTCAG
ET33-mi5C*	3:**AATAAAAG**TCATTCAGCACCAGAAGCAGAAGAACAGCACTCTGCCCACCTTCAGGGTCTTTCTCCCGGTAACACTGGTAGCTGTACTCCACTCCTAAATTATCCAGGAACTGCTTCACCTCCTGCTCGTCCTCGAAGTTGAT<br>TAAGCCGGCCATGTGTTCAGTTTTACtttttcagtttacaGGATACTTTGACACCTGACGCACTCCGGCTGAAGGTCTCGAGGAAAGACACTTTTGACCTTGGTCACTCGTATTGGCTGTACAAGAACTTGCGAAGTTTTTTTT<br>AATAGACGAATAACAACGCGTCCGTACAAAGCATAAGAGATTCGTTTAGTTCAGAGTTGACAAGCGGTTACAGATGTTTATTTTTGTGAAGCACTTACATCAGAACGTGCTTCAAGCTTCCTACTTCGCTTACTGGGACTACCG<br>GGCTTCCGTAGGCCCCGCCTATTGTTTATTGCTGATTGGTCGACTGTTTTTCCTATGATAAAATCCTCTATAAATAGAAATAAGAATGCAATTAACATTTGGTTAATTAAATATAGAATAAAATATTATTTTTGGGGCTAAAAA<br>TGTATTTAAAACAACTGAGAGGAAAATGAAATGTTATAGTAAATGAAATGTAAACTGTGTGCTAATATCACGACTCTTTCAGTCAAGGGTTTGGACCAAATTATTTTTGAAACTACATATTTTTACTTAATCATTTTTACCATT<br>ACATATAATTCAAACTGTATGTGTTTGTTAACAAA
ET33-mi5D	3:**TAACCACA**GATATCGTGTATTGGCCTTTCCTACTATGGAATTCAGTAGATACAGCTTATAAAAGGTCATAATTTAGTTTTGGGGGTCTCCATCAACAGGCTGATATGCATGCAAGGTCAAAAAATGATTTCATTGTCTTATA<br>ATGTGCATTTATTTTTACCGAATAATCCCAACGACTCCCATGTGATTCGTTCAACGATTCATTTATTCACAAACCCCTCCTTAGCGTGAAGCTAATCTGCTGTGATTGGACCAATGACCCAGTCTGCTGTGATTGGTCGACTGC

	GTTCAGTGATAGACAGAAAGAAACGCCCACCACTGTTATGAAGTAGCACAGAGTATGTGAGAGCCCAATGCAGGAATGCAATAAATCAATGCTGTTAAGTACCAGCATATTACTCTACTCTTAACGCTAACCCCAAGTAATAAC<br>AAGGACACACATTTAATATTAGTCAACACAGTGGCAAAAGTTAAACTATTTTTAAAAAATTGACTGCGCCGCGTGTGTGAGGAACAGCTGATGGTGGCCATGGGGACAAAAAGCAGGAAGTTCAGAAATGCATTTTAATCGGTA<br>AAGGAAGCTGCAGGTGTTGCGTTTTCAACATGGTTTTGGACACAATATTTGAATAGCCATAATTGTTTAACCAGAGAGATACAATACAAGCACTGATCTATAATAGATAAGTGATTACAAGACAAGTGGGGCGATTACAACTTA<br>TAACACACAATTAAACACATATTTTGCAAACTACACAAAATGAGGCAAAAATTTTAATTACACTTGTTATGACCTGAAAGAAGAAGAACTGGTCCATTTGAACTGTAACAGTTACTGACAAAGTTCCTGTTAAAGTCCCTTACA<br>TGATAGT
ET33-mi5E	3:**CCGTTTAT**TGTGAAGGATTCTGGGATTGCTCTAGTTTCACATGCTGTGCTACTTAAGATGTGCTGCTTTGTTGTTTGGAGACGGAGGAGCTTTTTTTGTCCTAGACATGTTAATGAGGCCTACAATATGACCTAGTTATGAA<br>ACTTAGTAGGTACAAGGTTTTTTTTCTTTTCCTTTTTTTTTGGACCTCATTCATGAAACAAACAGTAAATTTTTTTGTGTGTAAATTGGTTATAAAGCTATAAATAAGGTTCAACATAGCATTCCTGGATACCTAAATTTATGT<br>AGCTGGAATTTGTAGGCAAAAATTATGCAAATTGGAAATAATCCCATTAATATGTATTAATTAATTTATTTAGCATTGTAAATCATAAACATTTAACTGTCGAGTCTGATGTTTACAAGCATTTGTGAATTTAAAGGAAAGTTT<br>ATTTTAAAAAAAAAAATTTTTTTAAAGGACTGTTTTTTTATTTATTTATTTATTTTTAAGTTTGGGCCCTTTGGGCCCTTGCAGTTTGAAAATTTAGGGGGTTACTTTGATTTCAGGAAATTCCATAAAAA
ET33-mi5F	5:**CACCTGAC**CAATACATTCTGCCAGAGGAGAAACATTAGAAATCATTTGTATAATCATATATAAGATCACAACACAGAAACACAAGTCAATAACACACATAATCTTGAGGGACACACACCTCCACATAATGCTGCCAGTGTCC<br>AGATCAATATGCTGCTGAAGGTCATTGTTGTTCTTGTAGGTTCAGTTGTAGTGAAAACAGTTGTTGATCAAGTTTTCTGTTTGTCAGAGTTATAAACACATGCAGAGCACTGAAGCGTGTGGCGCTTATGTAAAACTCCACATG<br>CAAACACTGATGGAGTGAAGCTGATCTAATATTTTACTCA<br>3:**GTCAGGTG**ACAGTGACTCAAACTCCCTCAGTGCTGCTCTCTGAACCTGGACGATCGCTCACTGTCCACTGCAGATTCAGCAGTGATCCAGAGTGCTGTTATAGTGGAGATTCTCTTGTTAGCTGGTACTTACAGAAACCTGG<br>AGAAGCTCCTAAACTCCTGATTTATTATACAGATGTCCTCCAGTCAGGAACTGCATCTAGATTCAGTGGCAGAGGATCAAACAGTGATTTCACTCTGAGCATCAGTGGAGTCCAGACTGAAGATGCTGGACATTATTACTGTCA<br>GAGTTACCACAGTGGTGGTGTGTTCACACAGTGATAAAGAGTGGTACAAAAACCTCTGTCAGTCAGTCAGAGTCACCGAGACTGAACTGATCCTGGAGCA
ET33-mi5G	5:**TTGGCAAG**TGCTGACAGGCAAAACAGCTGGTGGATTATTAGCATTAAGCTTTGGGAACATTGTCTTTACTAAAGCAGCTGAAGTGACACTGGATATCGTGGTCGATGTGGAACCTGCAAACGACAACATTAATGGTTGCTTG<br>ACTGGCGTGGTGTGAAGGGCAAAGGGGGATTTTTGGGTAGCAGCTTTGGGCTTTCGTCCACGCCTAGTTGCTCCTTTTCTGCTCCAAGCAAGACTGCCTAAAATGGGACGAGCAATCGTCAATGTGTTTTGAGGCTGGGGAGTA<br>ATGAGTGGAGCATTATTATTGGTCAAAATAAATCTCTGCTGTGTCTGGACTGGTTGAAATGGCTGGATTTGAGTTTGGGGTGCTGCCAAAACTTGGGGCATTGCTGGAATAGTCTGAACCAAGGGGAGGGTCTGTGGTTCTGAT<br>GGGGTGGGAAGAGTGTTTGTACTGGATGCTGTGGGGACCAGGATCAGACTAGGCTGATTTCCATTGTTGGACTGGCATTGAAGAAGCAGGAAATTCTGTGGAGGTGGTGG
ET33-mi6A	5:**TATATATA**TATATATATATATATATATATATATATATATATA
ET33-mi6B	3:**GTTATTAA**TTACTCGCCCTTATGTTGTTCCACACCCATGAGACCTTTGTTCAACTTCATAACACAAATGAAGATATTTTAGATGAAATCCAAGAGCTCTGTCATCCTCCATAGACAGCATCGGTCCAGATGTGTTTAAAGTC<br>CAAAAAAGGGACCAAAACATTGTCAAAATATTCTGTAAGACTTTGATAAGTACTTCAACTTGGATCATGAAAAGCTTTAGGAACCCTTTTGTATTCCAAACTAAACTACTTTTTTTGACAATGTCTTCTGGATCA
ET33-mi7A	5:**ATCTGGAG**TAATAAGGTAGTTAATTTTTAACTGTTGCAAATCAGCAACGCCTGCCTTGAGAGGGTTAAATAGTGCAGTGATTATGGGGAGTATAAGTTATGAGAAAGTGTGTGAGGGTGTATGAGAGGAGCAAACAGTCAGT<br>GGGGGGCAAAGTTCAAAAAGGAGACAGCTCTGGGGAAAAAGCTGCTCTTCAGTCTGATGGTTTTTGTCCAGGGGAACCTGAAGCACCTGCCTGAAAATAGGACAAGAAACAGTCTGTGAGCAGGGTGAGAGGAATCCTTAAAAA<br>AATGCGTGCTCGGCGCAAACAGTGTTTATTCTGGATGTCCTGTATGGCTGAATAACAAGTGCGGTTCCTGTGATGCGTTGAGTAGTTTTCACCACCAGCTGCAGTGCCTTACGCTCAGCAACTGAGCAGCTTCCATACCAAACT<br>GTGATGCAAATGGTCAGGATGCTCTCTATGATGCAGTGGTAGAAGTTTACCATGATGGTTGAAGACAGCTGGTTCTTCTTAAGTTGCCTTAAAAAAAATAGGTATTGGTGAGCATGCATGACCAGGCTGGAGGGTTTTGGTGGT<br>CTAGGACAGGTCGTTTGAGATGTGGGTCCCCAGGAACTTAAAGGATGAGACAGGCTCAACGGCCATCCCATTGATATGAATGGGATCATGTGAGCCTGTTCGGCCCTTCCTGAAATCAACAATGAGCTCGTTGGTCTTACGCCT<br>GTTTCACACCGCAAGCGTCAGCGGCGCGTGAGCAGAGCGTGAGCAGCGCGTATGTCGAGCAGTAGCAGCACGCAGGCGTTTGCCTTTCACACCAGCTGCGTCTGCAGCGCGCAGTTCTTCGGCAGCTGCTGCA<br>3:**CTCCAGAT**ACATATTCTATGAGTTGTTGTACTCAGAAGTACCACTGCAGGACTTGGTTGTCCATTAGCAACAAATAGTTTCCCCACTTGCTCACCAGATGACACACACACACACACACAAAAAAAAAAGCTTCTTGGAGTACC<br>ACATGACAAAGAGTCTTGGAAACATGACCTACTCAATGAGGATTTGAGATATTGGATTTGGATCCCATATTGTTTTTGTTGTTGTTCTGCCACTGAAAAGCATTTTGTTACTTTGTTTGAAAAGTTGTATATAAAAAGTTATTG<br>TTCATATCATATTCAGTAGTAAGTGTCTAATTACCACACTGGGCCAATATTGCAAATCTGCAACATCCCAAAA
ET33-mi7B	3:**CTCACACA**CTCACTCACTCACTGTCCTTCAGCATAGTTACTAATTTATCAGGGGTCGCCACAGCGAAATGAACCGCTAACTATTCTGGCACATGTTTCATACAGTTGGCCCTTCCGCTACGCCACTGCCTAATCCCTACCCC<br>TAAACCTCACTCACTCACTGTCCTTCAGCATAGCTACTGATTTATCAGGGGTCGCCACAGCGGAATGAACCACTAACTACTCTGACATGTTTTATACAGCGGGCCCATTTGCTACGCCACTGCTTAATCCCTACCCCTAAACCT<br>CACTCACTCACTCACTCACTATCCTTCAAATAAGTTACTGATTTATCAGGGGTCAC
ET33-mi8	5:**CTTATACC**TGTGAACTTACATGGTAACTAGATGTGTAAGTAGTATGTAACTACAGTATATGTACACACTGGTACATAGTAATTACTTGTGTAATATTGGTGTAACTACACACATGTAACAGCCCACTGAATAGTATGTGTAA<br>GTACAAATGTGTAACAGGACATTGGTAACAACACTAAGAAAGTACACATCTGTAACAAGAATTATGTAAGTGCATTGTGTAACAACAGTATGTACTTGCAGGTGTTTTCTCACTTCCACACCTGTTTGCATACCATGTACTTGT<br>GAACATTGTATGACAAAAATAAAGCTTCATTAAAAGGTCTCACATTGTGATACCAGTGTAATTACAGGGTAAATCCACATAAACTGTCCACTCAATATAAAGTGTAAAATAGTCACAAATTACTCTTTAACTTGTAAAATATTC<br>TCTGTGCAACACTTTATTAACAGTGTACTTATATGGTAACCCCACAGGAACTGTCCACTCAATATAAAGTGTAAAATAGTCACAAATTACTCTGTAACTTGTAAAATATCCTCTGTGCAACACTTTATTAACAGTTTACTTACA<br>TGGTAACCCCAAAGGAACTGTCCACTCAATATAAAGTGTAAAATAGTCACAAAATACTGTGTAACTTGTAAAATATTCTCTG
ET33-mi9A	5:**CAAGCGAC**ACCTGACACCAAATTCACAGCACTTTTCAGTTCTTGCCTATATATATGCATAAGACAAATCCATTACACACATCACTGGAGTAAGTGCTCAGGTGAAACAACGGCTCGCAGTTCACTTGCACATAATTAAAAGA<br>ACTACATATCCCACTAGACATAAAACGCGATTGGAGAATACGGGGGGAGGGTATCTTG<br>3:**GTCGCTTG**TGAGTCCGCTGCCTTTTTGTACTACATTTCCCATGATGCATTTGTAACAGATGGAATTCACTTCAGACCGCCAAGTGCTGTGATTGAGGATGCTCGGGGAATAACTATCATACATACAAACTGTACGTAGCGGA<br>TGCTATGTTTCTCTCTTTGTAGATATGTATAAATGCTTTATATGAATTAAACGTCGGTTCAAATAGCCAGTTAGTTATAGTTAGTTTTACATAGATTTTCGTGTAATCGTATCATTAATTTCTTTTAATATTAAAATGCGGCAT<br>CAATAGTTTTCAGACTGTTATTCTTCATAATGGTTTACATACGTGGCATGAAAATTTGAGTATTCCTCAATATGCATTCTAATTGATGTGCTACTGCCGCCATGTGGATTTATGTATAAATATATTGAATTGATATACACCGCT<br>CAGAAATATTAAACCCCCTTTAAATTTAGTTTTTTATGAAATATTCCTCAAATTATGTTTAACAGAGCAAGAAAATTTT
ET33-mi9B	5:**ATAATGTG**GGTGTGTGCTCCTGCTCACTCACTCTCTGCTGTCTCAGTGTGCAGTGCTGAAGTGGCAGTTTGACCGCTGTGGACACAGTCGATGCGTTTGCAACATTTCTCTGCCTGTCACTGTTCTGCTGCTGTACGCTCCG<br>GCTTGTCTTACAGCAAAATCAGTCTGAAACATGAATATTCACCCATTCCTATGGCCCGCTTTATATAAATGAGTGTCAGTATTACATGCTGATAGTGCAGCTGCATCTGCCTCGTGCCGCTGAAACACAACACTGAACAAAATC<br>CTCTTTCTCA<br>3:**CACATTAT**ACACACACACACTGCACATACACTCACACTCAAAAACATAGACACACACATGCACATACACACTCAAACACAGGCAAATATTTGCACACACATAAACACGCAGATGCTCGCACACACACACACACAATTTACATGC<br>ACACACTCAAACACATATACACATGCATCCACTTACACAAACACACACACAAATGTGCATACACTCTTACACATATACACACAGATGCGTCTTTTCCACATCAGGGAGAGTTATGGTGTGGAGAAGCCCCAAAGAAGTGTCCCA<br>CCCAGACTGCTGATGCCCAGAGTCAAGCATCATTTGGGCTGAAATATAACAGGCTGGCATTACCTC
ET33-mi9C-1	3:**TATACATC**CAGTGGACTCATTCAATCCCATAATACACCACAAGAACACATGTACATTCAATATCCATCCAGTGGGCTCATTCAATCCCATAATACACCACAGCAACACATGTACATTCAATATACATCCAGTGGGCTCATTC

	AATCCCATAATACACCACAGCAACACATGTACATTCAATATACATCCAGTGGGCTCATTCAATCCCATAATACACCACA
ET33-mi9C-2	5:**GAGTTTAA**TGAAAAATAAAACAAAAACAAAACTCAAATCTCCCCATGTGGGAAAAAAACAACATTATATTACACACACAACTAGACAAGATCTGGAACTTTTCAAGACAATACTTTAAACCGCTGTTGTAGAAAAGAAATCAGCAACAGGGAAGGGAACAATCAATAACGGACCAGCAC
ET33-mi9D*	5:**GTAACACT**TTAAAATAATGGTCTTTTAgttaatgtaTTTACTAACACGAATAAACAATCAACGGTGTTTATTAATCTTTTTTTAACATTAGTTAATGTAATGTATAATTTAAGGTAATAGTAGTTCCTGCAAACTCACCGTGACTAGTTAACAATCACATCTTTGGATTTTAATAATGTATTAGTAAATGTTGA
ET33-mi10A	5:**CTTGCCAG**TTGCCCAATATTTTATTTTTCTTTTATTTTTTCTGAAAATCTATAGAGTTTAGGACAATTATCAAATTATGAAGACGTGTTTGTATGAAGACTGTGTATATTTATGCAATCTGATTTGCTGTTTGAGTTTGATTCACTTATAGTGTGCACATCATAAGTGAACATTTCAAAAGAGGTTT<br>3:**CTGGCAAG**TAACATTGGTTCAGTTGATTTTAGTTCTCATTTGGTGACTGGCGAATGTTAATTGTACAGGGTGTCTGCGGGGTCTTAAAAAGTATTAAAAGTTAATAAATCAATGTAGAGAAATTTCAGACCCTTAAAAATATTAAAAAGTCTTAAATGCTATTTTGCAAGGTACTTAATTTTATATCATTTTTGTTTATGCAATGTATGCTAAAGTTTGCCTCAATTAAATCTACGATGCTGTGTGGTTTATGAAATCAATGATATCCTACTAGATTTGACATCATGCTGCTTTGTTTACTGCAGTAACCAAGGCAACACCATTATTCTGCAGTTCTATAGGCGCCAACATGCTGAATTAGCTAAACTTAATATTTTTTATTTAGTATGTGAATAATGTTTTAGTGTTGGATTAGTTTCTTACATTAATGTTTAGTAAATATACTGTACGTTTGCCAGTGTTTTTGGTTGAAGTGGTTTTAAGGTCTTAAAATGTATGGAAAGAGTATTAAAAAAGGTCTTAAAAAGTATTGAATTTCACACTCTGTTTTCTGTATAGACACTGTTGTAGACTGAAGTATAATTTCTAGCTCACTAATATTAACAGATGGTC
ET33-mi10B	5:**CATCTTTG**TTAGCGACGCATACTGCGTTTTTCTTGCGCAGCGTTGTTTACCTCAGGATACTTAGTGAGGGAGAGCAAATGAAAACTTGAAACTCTAAAAAGAAACAATCAACGAATTCCTGTTTAATGTTTCATAACGAGTGAACAAGTAGATTAAGGACAAGCAGGAATAATTTTATTGTACCAGA
ET33-mi11	5:**CTAAAGAG**CAATCATGACGAGTGGGTATTGATTGGTATTATTATTTTTATTTTTGAAAGATATTATATCAGCTTCAGTTATCATTTATGAAAGTACTTTAAGTTAATCCTCATTTTAAGAGCAG<br>3:**CTCTTTAG**TTGTCGCCCGGAGCGCAAAGCTCAGCCTAGCGTTAACTCCCGTCCTTATGATGGCTGGCGGTCTCGAAAATACTAGGAAGCTTTACGACAACACCGCATCGGAACACAGCAGCTCATTCTTTATTACGTCACACGAAGGAAATCACCACACATAAATAGTCCAAAAGATAAACCCCTGGAACACATCAGATTTGTTTTATCATATATGGCTCGTTTTTCATTCACAAACATAAATTCGCAAATAATAACCCACCCGTCGCTCATTTTGGAATAGAATTTACTCCCTCCTAAAATGTTATGCATATGTATTTAAAGAAGATCCTCGATTGTAGTTGCTTTCCTTATGGTTATTTAGCTATCATTGTAATTTCATTCGTTTAATTTTGTTTACTTAATTTAGTATTTCTAGTATTATTTTTTTATTATTAATAATACAATAATTCTTATTATATTTTTTTATTGTTTCCCTTTTTGCCCAATGGTATGCTATTGGCGTTTGTATGTGATTATATTTTTTCTTATGACTATGTATATTATTAACCTTCTTTAAGATCTGTCTCACTGTCT
ET33-mi12	5:**TCCAAGAA**TGCTAAACACAGTAAAATTACTTTCGTTTATTCATTAAATGTCTTCAAAGCGGTGATTTTTAAAAACTGTTTTTGGCTTTTGGTCGTGATCGATTCCAGTCTTTGTTTTCTATGTGCTTCCCATTTTAACTTATGGAGACGGCAAGTTGTGTACGACGACACGCGACACAACGTAAGTGTCAATCATCAGACAAAGCCCCTCCTCCTCTGATGACATCACTAAGCATGACATACTGCAGTTCAGCTGCACTTTCCATGACTGACTGCCTAAATTACAATAAATGTATAGGGAAAA<br>3:**TTCTTGGA**CCATTAACAATCATACATACTCTGCTGATGGACATAGACTATCTTTGAAGGGTTGTCCATTTATATAAATTATGCTAAAATAAATGTCCTAAATCACTCTATATTAATTTTAATTTTAATTAAATATAAAAATGCTCATTTTATATTCTCAAGATAATTATTTACTTAATATTTTACTTAAGCTGCACCTGTGTTTGTTTGCATCCTTGTCTAAACTTTAAATGTAATAAAACACTACAACAATAAGCTAACAGCACGGCTGACAAATCTCAATGTGTGACTTTGATGAAGGACGTCATGCGGCTGATCTGTCAAAGTAGCTTTCCAGGTGGTCTCCGCCTCCCACTGGCTCCTGGCCGGAGAGCAAAGTGCTGGGCAAAACACAGCAGTCTCAGTCTTTCTGTCTTCAGCTTTGAAGCTGTGGGTGTCTTTGAAACTAACATCAGCTCTGTCTTCCTCCTCATGCTCTTCATCCCCGCTGCATGCATGGAGCTTTTAAAGGAGAACTGCTAAATTACATTAACC
ET33-mi13B	5:**AATATGGC**AGTTTGATATAGTACCTAATTGTTAGAGCTTTTGTTAGTTTTATATTGGTTCATTTATTCAATGTGATTTTATTATATCCGATATTTGTAATTCTTTAAATTATTTCAATATAGGCCTAGCTTAGGCTATTTTATCTAGATATGACTATTGTTTTGAGCCTACAAAAATGGACATGAAATTTGTAAAGTTTAATGTCTTTCTGTTGTATTCATATTGTGTATTATCTCCAAAATAAATAAAAACAATATCAGCAGAGCTATGAAGGGAGCGATCTGCAAATATTCAGCTGTTTGTTCAGCTTTTACCCAATAACTTTTTTTTTTCTGG
ET33-mi14A	5:**CAAATCGA**TTAAGTTTACGTTATCGATTTTACAAATTTAAGAGGATTAAACATTAAACAATTAAGTTGTCGCCAAAAATGGCAAGAATTTAGATTTTCAGCTCATTTTAAATAGCCTATAGTTTGAATTTCATTTAAAGTAAATAAAAAAAGGATAGACAATTAAATTCCATGTTTTAAACAGTAGCCTAAAGCAGATTGTGTTGCATCAGAA<br>3:**TCGATTTG**TTGTGTTTGGACAACATAAATGAATTGTGTGGAAGCCATGTAGTTTTCCCGTTGTATGCTGTACATTGTAGGCTATTATAAATTAATTCAATATTATATGTAGGCTATAGGTTTATTATAACAACTTATTTGCAACAATCAACAGATTTATTTAAGTAAAAGGCAGAAACATTCAGTTTTCTATATCAGGCATCTGCTGTAAATCGTCGCTCACCTCTTGCATACAGATTAGAGAAGTCCGTCTCTGTGCGTCTGAAGCGGGAATCTCGCTCGTTGTCCCGGGTTAAAATACTCCTGGGCTGCTGGTCGTTAAACAACCCGACAATTCGACCCTTGTCGTCAACATTATTGTTCTTCTGCAAGAACCCTATATCATTACACAGAGAAGCAGGCAAAAATGAGCTACCTTTTCCAAACATAGCAGCTGATAATTGAGCACA
ET33-mi14B	5:**GATTTAAT**TGAACAGGAATCACAGCACTGGTTTTCTAATCCCCATACACAAGAAATAATACAGTAGTGACTGCAGGTTAGAGGACAGTGGAGTAACAGTACAGATACTGCACTAAACATGTACTCAAGGGAAAGTAAAAGTGCACATCTTTAAAACTACTCACTAAATTATAATTCCTGAAAAAAACTAGTCTACAGTAATTTGAGTATTTGTAATTAGTTGCTTCACACCACTGCTAACCACAGTGGCTGGTGATCAAAAAAGCTAATATCAAGCCCTGTTGTCAAACTGCTGTACTTTATTGTGTTGTCAATATGATCACGTTTATATAGGACTATTAGTGTGTGTGAGCCATAGCATTCAACATTTGTATGCTTATAACCTCCTCTGAAAATAGTGGCGGTCGTGAGTCACTGGCATTGTTATTCAGGGGGTCATAGGATGAAACGTTTGGGAAGCTAACTTAAGTGTGTGAAATAACAAAGAATGTGCTGCAGTTTATTACAAGGCTTCTGCAGTGTAATATACAACACCAACACGCATAAATATAGCACTGCAAACGGATGCAACGATTAACGTTTTTACTAATAACCGTGATTTAATTCATCACAGTTAAATAATCGTAAGGCTTCTCAACACCGTGTTTTTGCACGGAACAAAACTGCAGCAACTAAACAAAGTGATGCCAACTGTGAGCTAGTTTGTACACCGAGACTAATAACCACGCACACTGGACTGACTATGACTGAGGCTATTAATTAAGGCAAGCTCGTTTAAGTTGGTTATTTCCAATGAAGGGACGCGCACGCAACGCAATGCGTTCACATTTTTTAGGCGCACCTAGTTGAGATGACGGGGCTTTTGTGACTGCAGGAACTGCATACGGCTGA
ET33-mi15A	5:**ATACTGCG**GTATAGTGTGCTGTTACTATAGTAAACGGCTCGGTAACAGTAGTACAGTGTTCTGGAACGGGTGTGCGGTACCGGTCGTGCAGTGTAACGGGTCCCCGCGGCTCCGGCAGCAGGTAGAACCGAATCTCAGCAGCTCTGCTCACGGAGGTCCAGATGAAGCGGCGTGTGAGCTGATCCAGAGTCAGCCCGAACTCCTGCCGGTTCTCCAGCCGCAGCCACAGAGTCTCAATAGTGATGCCGTCAAGACCCTCCAAAGCCACCTCATCCAGCACCGCGGCCAGAGGGTCCAGCAGATCCATGTTCAGCAGCTCTATTATTATTAATAATATTACTGTTATGCTCAGCGCGCTCTGCTGACAGACAGCATTCGAGTGCGTCTACTTCTTCTTCTGCTGCTGCTGCTTGTCTGCCTGTGGCTGTGCACTACTGCACCTGCCGGGCACCGCCGGAACTGCAGCAGCACATATAACATTTACATGGTCATTTTACTGGTCTTTGCTTGCGCGATACACACATCATTTAGGTTTGGGCAATACATTTCCAGCTTAATTTTGCAACTCATAATGCGTTAGAAATA
ET33-mi15B	5:**TTCTTAAT**AAATACAGATGCCTCTAGTATAATTTCACAAAGCATCAGATAAAAAAAAAAAATTAAAATCAACCCAAACAAAATTAAAAAAAAAAAAACTAGTTAATTTTGCCCCCTTGCAGGGTTTTTTAACCAAAAAAATTTTTC<br>3:**ATTAAGAA**AGACATGACAATGGCTCTTGTGATGTTAGGTTGTATTTTGAAATAGACTCTAATACATAACATTGCATCTTATCTTGAACCCTATTTACACCTGGCCTGGTGTCTTAAAAAAGTTGCTCTGTTGAAATCACTGAAGATACAGGTTTAACCAGATGTGATTGACAATGGCTTTTTTTTTTCC
ET33-mi15C	5:**GTCCCCAG**ATCTTAATCCAGTAGAGCACCTTTGGGATGTGGTGGAACAGGAGAACGGCATCATGGATGTGCAGCCGACAAATCTGCAGCAACTGTGTGATGCTATCATGTCAATATGGAGCAAAATCTCAGAGGAATATTTC

	CAGCACCTTGTTGAATCTCTGCCATAAAGGATTAAGGCAGTTTTGAAGGCAAAAGGGGTCCAACATGGTACTAGTAAGCTGTACCTAGCCAGTTTAATATTTAAAAAAAAGTTAGTGGTGAGTTTATGTATATTTTTCTTTTCCTAAAAAAGCTAATCTAATGCTAACTATAACAACAGAACAAA<br>3:**CTGGGGAC**TGTGGAGGCCATTTGAGTACAGTGAGCTCATTGTCATGTTCAAGAAACCAGTCTGAGATGATTCAGGCTTTATGACATGGTGTTATCCTGCTGGAAGTAGCCATCAGAAGATGGAGACACTGTGCTCATAAAGGGATGGACATGGTCAGCAGCAATACTCAGGTATGCTGTGGAGTTGACACAATGCTCAACTGGTACTAATGGACCCAAAGTGTGCCAAGAAAATCTCCCCCACACCATTACACCACCACCACCA
ET33-mi15ix	5:**ACCATGTG**CCCCCTAACACTGGCATGTAAAATAATGGTGGACGTCCAAATGACAAACCATCTGGTGTGTCCCTATTGAAGTACAGAATGGTGACAATGATGGCATTTATAATAATGTGTGAAATATCAGGTCTAATCATCACTGACCTTAATTTTCACTCAGTTCCATATCTTCGTGTGACATTGAAAAAGTCATTTTTTTCCCTCTGGTTTTAATGGATTTCTGCTTCATTTTTTTGTTGTTGCTCTGGTTCTAACTAAAAGTGGCCATTCTGATGTTTTCAAAGCCACACTAAATGAGTTTTCACAGTCATAAGGAATGTTGTGTACATCAGCAATCTTGTAGCGATAGCGGGTGAAAGTGTGGCAAATAGCTGTTCTGAACTCATCATGTTTGAAATGTTCAGGGATTTTAATACAAAATGCGCCATGTA
ET33-mi16	5:**TATATACA**TATATACACACACACACACACACACACACACACACACACACACACACACACACACACACACAT<br>3:**TGTATATA**TACACACACACACACACACACACACACACACACACACACACACACACACACACAATATATATATATATA
ET33-mi17A**	5:**CCGGGTTG**TGGATGCACGACATAGGATGAATCCAGGCGTCCCTTTCAATGTTATTGTTGGGAAGAACATCGTCCTTGCCACGGTTCCATGTTGGCATTTCTCACCGAGGCCTGTCACATGACACGCATCGCAATGCACTCGAGGGCACGGATCCGATTCCTGGGTTCTGATTGGTTGCGTGGCGGGGGCTCGCCGCAAAGCGAAACGCGTCATTGGTTTGGAGTAAAACAAGCTATGCATTATTTATAAGGATAGGAAAAACATCCAACGAAAGCAAAAAAAAAAAAAAAAAAAA<br>3:**CA**ACACTTTAATCAATATTCACACGAGGTAACGAGAAGGAAAACTATCGTCTCGCGTTCGATAAAACAGGGCGAAGTCGATTTTATTTTACCACAGGGTCAGATTCGATCCAAATGGCTCCACTTTGACGAACTTGACACCTGTTTGCGGAAATATCTTAACCGTGTTTTCCGAAATTGTGCAGTTTTTCTGGGCTGTGTGCAGAAAGCATGTGTGCAGGCCTGTTTTCCTGCCCTGCTGACGCACTTTTGAGAAACACATGTCTGGAGCCTCGTGTTTGTGTGTGTGAGTGTTGTAGTGGAGCTTTGCTGCTGGCTTTGATGGGAGTTTGATTTGCTAGTTTGCGTTTGGTGGATTGTGTTTATGTTGTGGTGGTGATTTG
ET33-mi17Aix	5:**TTTCTAAG**GTAATCTGCCACGTTTTTAACTGTGCAGAGTTGTAGCTGCTTTACTAGGCCTACTACACTGCTGTATTTCAATACTGCTTATTTTGGTGGTGATTGGAGAGACAATTTCTTTCTGAGGTGGTTCTTGATGAAAAAAGTTTGAGAACCACTGGTTTAAACAATAGTCATTATCTTTAAATAATCTTTAAAAAAAAAA
ET33-mi17B	5:**GTAATTAG**GCAAGTTATTGTATACTGAGGGTTTGTTCTGTAGACTATCGAGAAAAAAAAAAAATTACCCAAAGGGGGCTAAAAGGGCCC
ET33-mi17Bix	5:**AAATCTTA**TTACTCTTTTACGCTCTTTGAGAGTCTTGGCTGGATTTCAAAATGATATTTAGGCAACTATGAAGTAGCAGTGCCGTCTGTGTACCATTAGATGCTTGATAACAGATATAAGTTGAGTTTTGGGGGGGGGGGGGGGGGGG
ET33-mi18	5:**GTGCTCCC**ACAGATGTACAGGAAGTCCAGATGCTGCTGAGGCTCCAAAACAAGAACTTGGTTATCAGTGTCCAATGATGTTCCCGGTTCGTTCTCAATATCACAGTTACACGTCTGACAATCTGGACTGCCAACTGGTCCCGTTTTTAATTCCCATAGTTTGGTAAGATTTTTGTCCAAGGCTTCAACTACATTTGAGAGGCCACATAAACTTCATCAAAATATTCATTAAAAACTATGTTCTGTATGGCATTGGTGGTTTGGAAAAAGTAAACTGGGTAGGTCACAAAAAAGTTTACATTCATTTGTTGGATGGTAGGGCATGTTTTTTGGGCACAGGTCAAATGTATCTGCAACCAAAAACATGTTAACAAGGTCTTTGGGCTGATGAACATCTCCACTGTCCTGCTGTCACTGCTTTACTGCTAACACATGATGACAATAACCAAAACCTTTGAATGGAAAAAAGACAGTGAATGTGAGATGAGCATGTACACTGTAAAAATGCTTTCAGCTTTATGTGAAAACAATATGCATTCCATCTCTTTACAGTCTCTTGGCTGAAGGGCGGCTA<br>3:**GGGAGCAC**CCAGTAAGGGAGTCAGTAACAAGCTTGAACTTGATAAAGGCAACATAGCCTCTAGCCCCGAATGTTTATTTAATAAAAAAGCCAACTCACCCTCGGATTGTCCAGACTGCGTTGCCAGTCCTCTGGGAACGAAGGTCACTGTGGTAGACGAGGGTTATTCAACATACTTCTTCACAGCAGCCACCGTCAACAGCAC
ET33-mi19	5:**CAGATATG**TTTTAGAAACTCTTCAGAGCTTATGGAAACCGTCCTGTGTTGCCGTTAGCATCCGGGCAGAGTAGGCTAGGCTGCTCCGGGGATTCCCTCGCTAGTTTGCTTCGAATTAAAGGGGAAGGGTCGATTTTATTTTACAGATGGGTAACAACGCACAAAATGGCATTAAGCATGACAGAAATGTGTTGAACATGTACATTTGATGTTTTTATG<br>3:**CATATCTG**GTGAGTAAATGAACATATGCTTACTTGTAAAAGGCTTCCTGACTGAAACATATGATTGTTTGTTATTCGTGTACGTTAATTTAGTTCTTTTAAACACCGCGGCCCTTTTAAACTGGTTCATTCGTGGCCGTCCATATACCGTTAGTCCATAGACTCGCGTGATAACGTACGCTTGAATTATATTAAGTGTTGACAACCATTAAAGTCGGAATGTTAACATTATTGTCTTTAAATGACCGCGTTTGCACTTTCTTAGGCATTTAAGGTTACCGAAGTCTCCCGGAAGTTGCGGGACTAACGTTAACGTTATCCCGCAAATGCACAGAGACTCCCAGATGCCCAAGTAGATGCCCCGCAAATGAATGAATGATAATCTCCCGGAAATCGCGCGTCTCCCGCCCGGTCATTAAACACTTTTCACACCCCTCTCCACCGCATCCCTCCCAGCTCTTCAGGTACGTCGCGCGCAACTCATCCCATTGTTCTTTAGGTACTCATCTCTCCCGCTACCTCCCGCAAATCAACTCTGTATCCCC
ET33-mi20	5:**TCTCCCAG**CCCACCAATAAGGTGCGTATTGCGTCACGCAAATAACTTGAAAGGACTTTGCACGACTGATGTTGTTTTTTGTTTCTTTTCTGGCGTATTCCTAACCTGATTTTGTAAATTTCTCCAGGTTATCAACAATGGAGATCGTATACTCCGATATGGAAACCTCAAGCTGTGACTACTCCTTTTCGCACACGGATGATGAGGACTCGCGCAGCAGCCTCCACCCCGCGTCCCCCGCGTCCTCCTGCGGAAAACCACCTGCGTCTCCAGCCGGGCTCCAGCAAAAAAAAAGGCGCAGGGGGCGCGCGAGGAACGAAACCACTGTGCACGTCGTGAAAAAAAACCGCAGGCTGAAGGCCAACGACCGCGAGAGGAACAGGATGCACAACCTTAACGACGCATTGGATGCTTTGAGAAGCGTCCTGCCTGCGTTTCCTGACGACACAAAGCTGACCAAAATTGAGACTCTGCGCTTCGCTCACAACTACATCTGGGCACTTTCGGAGACCATCCGGATCGCAAACCAGAAGCAGGGCAAGTCAAGAGACGGTCCGCTGCTGCTCCCCGGACTAAGCTGCATGGCAGATGCACCCAGCCCCGGCAGTGACTCTTGCTCCTGGCCGTCGGGGGCATCCTCGTCGTCTTCATCACCGTCTTACTGCAACTCAGACCCGGGCAGCCCCGCAGCCATGGACGATTTGGATACTTGCAAACCGACGTAGTGTACAGCTGCCGCAACTTCGTGCCTAGCATCTATTAATGTGACTTTAAGCGTTCTCATGTCGGTATA
ET33-mi21	3:**CATAAAAG**TAAGTCCAGTTAAAATAAAATTAAATGAATATTCATGGAATGTGAGAGCGAATGGGTGTTTCCCAGTGCTGGGTTGCTGCTGGAAGGGCATCAGCTGCGTAAAACATATGCTCGATCATTTGGCGGTTCATTCCGCTGTGGAGACCCCTGATTTATAAGGAACAAGCTGAAGGACAATGAATGAATATCCAGCAGAAAGGATGAGTAAATATGCGTGTGTTTGCACCTGTACGCAGGTGTTTGTTTGTTTGTGTGTGCGTGTGTGTGTTTCTTTTTATACATGCACGAACCAACATCAAAACCTGTGAATGTGTTATTAAAATATTCAAATATCTTCTTCCAGTGTGCCGTATATTACCATATAAGAATATATTATACGTCACATTCACTTCTGTGAAAAAGTCTCAATGGGACTCATTCCTGTCTCAAAAAAGCCCTTTTACCACATTCATCACACTACTAATGTATTTTTCATTTGTATTCTCCTCTCCACTTGACATCCGCGACTCTCATAATGGACAAAAGTGTTCGTGCCGCCCGCTCGCTGCTCAGCCCAAAGCCAGCTTTACAAACCGCACACAATTTCAGCCAATCCGCGGCTCTGT
ET33-mi22A	3:**TCAACATG**TTTAACTTTTTTTCTTCTGTTGAACACAAAAGAAGATATTTTGAAGAAAGCTGGAAAGCGGTAAGCGTCGAGTTAAATTGTATTTGTTTTTCTTACTAAGTCAGTAGTTCAATAAATAGTATGTAAATTTTTATTCTGGGGGTACTGTCACTTTAAGAGAGCATGCGCGCTTTCGGTTTTTTTAATCCTTCGTCTTTCTACATCTCCACCGGCCCTTCTTTCCTTTTCTCTGTGGGATTTTCCTACTATGGTGTGATAAACTGCACAGATACTCAATGTTCAAACACATTTAAACAAGTCTATG
ET33-mi22B	3:**CGTTTTGC**TACTTCAAAATGCAACCATAAATTGTACTGTGTACCAAAATATCCTGAGAGAGAATGTCCAGGCCATCAGTTATTGACCTCAAGCTGAAGCGAATTTGGGTATTGCAGCAGAACAATGATAAAAAGCACAGCA
ET33-mi23A*	3:**CCTTTGTC**CCACAAAATCAAGTGTGATTTTCAAGAAAACAAGCTAAAAAatctccATGGCAGGTTATTTTGCTTGTTTGACTGAAAAACTCACTTAATTTGGACTCATATGTTTTGCTTGTCTAGAAAACGCTGAAGAGTTTTCAGATATTTGGACTGTAAACAAGACAAAAACTCCAAGTAAGGAAACATTTTTGCATGAGTTCATGCAGGGACTGTCTGATGATCATCTGATAAGCTGTTTTATAAGCATATAAATCAAGATGTAGTCAAATGCACATTCAGCGTTTTGAAGAACGGGTCGATTCAGTCGTCTGTATCGCAGTGATGGTGCATTGCAGAACATTTCCAGTAAAGGAGCTCCCTAAACTGGGAGCAGCGGCACACCCACTGACAGCAAAGCAGACCTCTTCAGTGGCATTCAAGAGCAC

	TGTGAGACCATAATGCTTATTATGAGCACATTTGATATATGGGCTGAACACAACATATTAATTTGATGATGTCTCACAGCAATCTACACTTATATTTATTTATTTGGCAGATGCTTTTATCTGAGAAAAGCTTGAAAAAGAGGA<br>TAAAAGAAGTTAATTTAGCATTTTTTAAATATATAGATGAGTATTGAGTGTCTGTGGGAAAGTGTGTGGTGCATATGGAGAGTAAAAGAAAAAGACTGTCTCCTACAGCTGGTTTGTCAAGCCCACTCACCTCTGTGAGGTACT<br>CATTGACCTTCAGCAATTTACATTTATTTATTTAGCAGGCGCG
ET33-mi23B	5:**GTAGCTGG**GGTGGATGCAAAACACACCATGCTGTAGGGTGAGCAACAGGATAGCTACTATGAGTCAAAACCGAATCATCACATCAAATGCAAATGGAATGAAATCACTGCCGTCCATAAAATCGCTTGTAGATTTTTTATAA<br>GGTAGGCTATATTAAATTTTATGCGCGCATTTTTTTATTTCAACTATCTCACAAATTAAATG<br><br>3:**CCAGCTAC**TGCAAACGCTGGCAACAGGCTAGTGCATCTGTGAATATTCGAGCGTACAGTATCAAAAGGTGGTTCCTCAGGTTATTTCAGCGTTCGCGTCCACCGTGTCACCGCTGACGTCGGCTGACACTCGGTGGAAGCCC<br>CTCTCCTTCCCTCGCGCTTTTTTGTTCTCCGGTTTGCTTCATTCGCGAGGGTTCGTCGTGTTTTCCTCTCCTCGAGCTTCATGCGGGAGCCGCTTTTCTATATCAATGTGCATTAATGTAGGAGACAACGTGGAAGAGATAAGG<br>TGAGATAACCTTGTTGATAACAAAGAGCACTACGCGAAGGTCTGCGTTTGATTGTGACATTGGCGGACTTGTTACTACCACGCGCTTTGAAAACTCGACTACAGTTGTCTGCATGTGTAACTGCATGTTGATAGATGTCGATAG<br>AAGGTACGAGAACCCTCGCTTTCCTTATTGTGTATATAAATCACGCACTTTTGTTTATTTTAATGCATTTCTTGCAGAGCTCTTATCTCCTCACCTGGCACCTTTGTTTTGGATTTGCCAAGGATTTCCTAAAGCGGCAGTGGG<br>CATACACGACTGAAGGATTTCGGGTTTTAGTCTAATTCGGGATCTATACATCCAAACTGGGATCTATGAGCGGAAAAGTGGCTAAGCCTAAAGAAGAAAAGGATGCTTCAAAGGGTAAGGAGAGTCTTAAACCGTCTCCGTTCA<br>TCACAGGTTTAAAGCGACCGTGAGCTCTCATTCATCTCCCACTCGTTGCATTGTTACTCAATTTTGAAAACATAAAAGCAGTTATGGAAATTGGCCAATATCAATGTGCAAACGTTGGTCTAGGACAAACGTTT
ET33-mi24	5:**TCCACCAA**TCAGGCCTGTTGGGTAGAGTGGCCAGACGGAAGCCACTCACCACCTGGAATTTGCTAAAAGGCATCTGAAGGACTCTCAGACCATATGAAACTAAATTCTCTTTGGAGTGAATGCCAGGCATTATGTTTGAAAA<br>AAAAACAGGTGCCGCTCATCGCCAGGCTAATACCATCCCTACAGTGAAGCATGGGGCTGGCAGTATCATGCTGTGGGGATGATTTTCAGCACCAGGAACTGGAAGGCTAGCCAAAATAAAGGGAAAAATGAATGGAGCAATGTA<br>CAAAAACATCCTGAAAAAAACCCTGCTTTAAAGTGCTCTTGACTAAAGAGAAAGGGTTGTGATGCTGTCTATGTGTGTTTCACTTGCTTGCTGCTGTGCATATGTGTTTATACAGGCTGTCTGGACTCTGTATAGGTGGGGGT<br>TTTTGGTTTGAAAAACAATTGTGTTGTGAGGAATTTTCCCCTCTTTG<br><br>3:**TTGGTGGA**TTGCTGCAGAGATGGTTGTCCTTTTGTAAGGTTCTCCTTTCTCTTCAGAAGAACGCTGGAGCTCTGACAAAGTGACCATCGGGTTATTGATCACCTCACTGACTAAGGCCCTTCTCCCCTCATCACTCAGCTTA<br>GATGGCCGGCCAGCTCTAGGAACATCCCTGATGGTTCCAAACATCTTGCACTTATGGATGATGGAGGCCACTGTGCTCATTGGAACTTTCAGAGCAGCAGAAATTATTTCTGTAACCCTCCCCCAGCCTTGTGCCTCAAGTCAA<br>CCCTGTCTCGGAGGTCTACAGACAATTCCTTAGTCTTCATGCTTGCTTTGTGCTTTCACACACTGTTAACCCTGAGACCTTATGTAGATAGGTGTATGCCTTTTCAAATCATGTCCAATCCACTAAATTTAGCACAGATGAACT<br>CTAATTAAGCTGCTGAAACATCTCAAGAATGATCAGTGGAAACAGAATGTACCTGAGCTCAATTTAGAGCCTTACGGCGCTGTGAATACTTCTGTAAAAGTCATTTTTT
ET33-mi25	5:**ACTATATA**TACACCTCTCAAACACACACGTTGCTGA
ET33-mi26A	5:**CGCGGATA**TAACTGAGGATGCACGGGTGCTGAGGAAGGGAGGGAGGTGTCGCGGACACCGTGCTCTCTATTCTGCCGCCCTATGTGCGGATATAACCGAGGATGCGCGGGTGTCGCGGACCTCTATTCTGCCGCCCTAAGCG<br>CGGATATAACTGAGGATGCCCGGGTGCTGAGGGAGGGAGGGAGGTGTCGCAAACACCGTGCTCTCTATTCTGCCGCCCTATGTGCGGATATAACCGAGGACGAGCGGGTGTCGCGGACCTCTATTCTGCCGCACTATGCGCGGA<br>TATAACTGAGGATGCGCGGGTGCTGATGTGTCGCTGACGCCGCCCTCTCTATTCTTGCGTCTATGCGCGAATATATCTGAGGACATGGGTGGTGAGGGAGGTGTCGCGGACATAACTAAGGACGCGGGTGGTAAGGGAGGTGTC<br>GTGGATGCCGCCCTCTCTATAGTGCCGCCCTAGGCGGCCGCCTAGGTCGCCTCTATGGACGCGCCGGCCCTGCTTAGGAGCCGTATCAGCAGATATTAAGCAGACAGTCTACTAATACACAAATGGACCTTCAAAGTAAAGTGT<br>TACCTAAAAAGCACACATTTTAAAAACTACTTGGTATAAACAATTCCTGAGAAAACTACACTTTACAATTTAGTTGTCTAGAGAGTTATCTGTGGAGGAACAGAAGGCAA<br><br>3:**TATCCGCG**CATAGGGCGGCAGAATAGAGGTCCGCAACACCTCACTTCATTTTCATAACCGGTCACTTTCGTTTTCACATTGGGGTTGAGGGCGGCGTGATCGTCCTCTGCCTAGAGCGGCAGTTCAGCTTGCTCCGGCCCTG<br>CTCCTAAGAAACAAAGCATGTACATGTCAACTTACACTAACCCTAACCCTTACCTAAACCTAACAGTCTACTTCTAATCTAATGAGAATTAGTTGGCATGTAGATGTAGTGTAACTTAAATTCAACAAACGGACCATCAAAATA<br>AAGTGTGACCCATTTTTAGTGCTAAAGTAGTAATTTTACTGCACTATTTTCCTTTAACCTGCCTTCATTAGTTTATATGTTTATTTATTCTTGCCTACAGCGATTGGCTAAGTGTAATCAGTCCCTTAATTCCATTCAATCAAA<br>GAAAAATTGTATCAGATCGACCTCAAGACACAGATGCTTAATAAACACAGCAAACATACAGTGTGGAAAGCCTTAAAAGTGCCCAAAAAGATGAGAAATCGTTACACGCAGTGGCTGAGAGACTATTTAAACTAACATCAATGA<br>TGAAAAGACGAAGTACGTCTACTGAATTCACTTTAAACAAAAACTAAATGCACTACAGAATGTTACGTTTTCACACAAATCACAGGATTTCTATCAGTGCTGCAGGTGCTTGCTTTGTGGTGCTGGACACTCATAGCTTGTTAG<br>CCAGGATTAAAGTACATAACAGTAGCAAGCTGGGACCCTCAGGATAACTGGATTTGACTCCGCATGTAAAAGCATCAACTTATTTTATATTTGCATATATTAATGTAATGTGATCGCACATCAGAT
ET33-mi26D	5:**CTCTGAAC**ACATACGCCGTGTTCAGCTCGTTAGTGCGAACTGTAGTTTCTCGCCGAGTTTGAATTTTAATAATTAATCTTCGCTTCAACCCCTTCGCCGCTGAAACCGGGGACTGTGTATGGAGCTCACTGCTAGAGACGAC<br>CATGTGGACTCTTCTCGCCTCGGACCGTAGACTTCAGATGGCCTTCTCTTGCCTATTATAGTCCGCTGCATGGTGATTTGGGGGAGCTACTGTGTTTTTATGCTCTATTTATGAGGATATGGGGCTTATGACTGTATCCTGGGA<br>TTTCAGACCCGGACAGCTGCAGATCTCCCTTCAGCGTCTGGACTTCTGTTGTGATACACATATATCCAAATATTACACGCACTGATATTAAAGCCGCTGCAAGACGTTAGTTTGGGTGATTGAAAGCCTGACATTTAACGTCTG<br>CCATCTGTATCATACAAACATTTGGGAACATTCTAGTTTGTTTTTGTTTGGTTTTGACCAAAAATCGTTGGAGCTGACCACAGACCTCATGTGGACATCTGATCTCGCTCCTGTGTTTACGTGTTTGCAGTATATCAGTGTCCC<br>CAAATCATCTGCAAACACTCTTTAGATAATTGCATATCGGGTTCCGTTGGAGGAGAGATCACATTGCAAACATGAATTA
ET33-mi27A	5:**CTTATTTC**TCTGGGACTGTCCGGAAGCGATGAGCCGAACCTCTTTCTAGCCCCACTGAGCGAGATGTCCTCTGATATTGGAGTTTCTCTCTTTGACGTGGATGATGTGGAGTCTTCTGAGTGTTCAGCGTGCGTGTGGAGAG<br>AACAGAGTAAAGTCTTGCGACTGGAAACAATTAAATCACAAATACTTAGCAAACTGCGCTTAAAACAAGCACCCAACATCAGCAAAGAGGTGGTTAATCAGCTTCTTCCCAAAGCACCTCCGCTGCAGCAGCTGTTGGACCATC<br>ATGACTTCCAAGGGGACGCATCTTCCTTAAAGGATTTCATAGACGCCGATGAGTATCACGCCACCACCGAGTCCGTCATCACAATGGCTTCAAAGCGTGAGTTACAAAACTTCAAGCTTCAAGCTATCTTTGCTTAAAATGCAT<br>AACTGATGTCTGTTTCATTCACAACACTCTCGCTCCAATTTTCTTTTTTCCTTCAATCGCATTTACACAACTGTTTTGCACAGTTAAAGGACAGCCACAAATATGCCACAGCATCTTTACAAACCCTTAAAGGATGACATGAAA<br>GAGAAAAATGTGTTTTACGTAATTTACAAATATTTTTCAAATACAATAATACACATAGCCTATGATGCGAGATCTCTAACCATTTATGAAAGCCGTCTTTGTTGCGCCTGTTACTGTTTACTTGAAAATAATCAATATCTTTAG<br>GCAGTTGCATTGAATGTGGTTTACATTTACGTTATGCTAGTTACAAAAACAACAACAACAACAACAACAACAACAACGGAGAGCTATACAATATTTTTATATAATAAATGGTGCACAAACATGCTTTCTTGCTGGTCAAGAAAC<br>AGAGTCCCAAAGTAGCCTAC
ET33-mi28	5:**GTCTTCTG**AATCATTTTGTAATGTAAATCCTGTCAGTGACTGCAAGAGCGACAAGTTACTTAATTTGACAGCTCAACCATTGATTTCGGAAAAGGAAATCCACTCAACGTGAAAACAAACACAGTTGTTTAAATTAATTGTG<br>GTCTCTGACTGCAAGTGCCGAAATCCGACTCATTCCTGCGAATCGGTTCCTTCGAACGGTTCAAAACAACTGAAACGATTTATAGTGATTCTTTATTTCGTTATGTAAAACAAGCCTACTTTATGTCATTTCATTATTGCATTT<br>TATGAATGTGGTGTTTAACGTGATTGCATACTAAAACATAATAAAATATAATTATAAAACGTAGGCAAATTATTTGCCCACATTTAAGCAAAAGTGTAACCTAAACATATTTTCAGCGTTTTTATTGGTGGCCAATCACTTTTC<br>TATCGTGCTATTGGAATAAAACAAGTCAAATCCTGACTCTAAATGCTTTTAAAGATATCACCCCAAAACAAATTAGCTAACTGTCTGGTTACAATGTCTGGTCGATCAATGTGATCAATTGATCAAGTTACTTTATTAATTCAT<br>AAAACCTCAATGGACTGTTAGACGCAGTGGGTAGGCTATCTTTTTTCTCTTTCCACATACACACACACACACACACACGCACACGCACGCACGCACGCACGC
ET33-mi29A**	5:**C**CAGTAGGTTGTCATCTGTGCACAGAAGGGTTTGTAGGCAAAGATGTTATAAGACCGCTGTGATGTGCTGGCACTATTTGGCCAAGTTAAGGGAGTTAATCAGGATGTCTTCAATATAGACAATAACAAAACAATGGAGGAA<br>CTCACAAAACACCTTGCATAATGAAAGATGGCTGGGAAGATGGAGAGTACATACAGCATGACCTGGTACTTGCAGTACCAAAAGGGTTGACGAAAGCTGTCTTTTACATCTTCCAGTCGGTAAGTAAGGCAACTGGACTACCTG<br>GGAGTTCAGTGTGTGGTATTTAATGCTCTTCAGCTGCTTCAAAATGCAGCAGCACGAGTGGTCTTTAATGAAGCTAAAAAAGCACATGTCACTCCACTGCTCATCTGTTTGCACTGGCTGCCAGTTGCTGCTCGCATCAAATTC<br>AAAGCTCTGATGTTTGCTTACAAAGCGACCTCTGGCTTTGCCCCTTCTTATCTGCTCTCACTTCTGCAGATGTATGTGCCCTCTAGAAACTTGCGTTCTGTGAATGAACGTCGCCTCGTGGTTCCATCCCAAAGAGGAAAGAAA<br>TCACTTTCCCGAGCTCTCGCGTTCAATCTGCCCAGTTGGTGGAATGAACTCCCTAACTGCATCA<br><br>3:**GGACATCC**AGGCAGTAGAAGACCCTCTGGCTCCTGCAAGCTTGGTATTGGTGGCACAGTGGATGCCAGTGGCCAAAGATGTCATCAGCTCCATCCGTCCCCAACATCACATAAGGGTATCAGGAGCCTCCTCTGGAGAAGAC<br>TGTAATCTCAGGCAGTCACCACAGCCACCTTCCCCACCTGAATCCTAATCAATCCGATCAAAATCCCCTGCTTACATTTTTATCGCACCTCTGCGCTCCCTCATCAGTTCCTTTTATTAACAGTCACTCTGCTCTTACCCAGAG

	TTTGATCTACGGTTCACACCAATGAACTTACCTGACTCACCCATTGCTACTTACCTGTTTGCTGTTGTTGGTTCTTTGTGTCCTGGTGCCTTTGTCTCCTGGTCTCATTGTTTTCTGTGTTCTCCTAGATTAATACCACTACCT<br>GCACACAAAGACTCTAAAATAAGCAACAGGCCAATAATGATTACCTGCTCCACTCAGTGCGATGTTCAGACCTAACTTTGTTAACCGCATCAGCTAAACTCTCCCACTCTATTTTTTTCTTTTGTTATTAATTCTGGAGGACAA<br>ACTTGCAAATAAAACAGTTTTTCTCTGGTCTACCTCCGAAAACAG
ET33-mi29B	5:**ATTATTAG**TCCCCTTTGAATTTTTTTATTTATTTATTTTTTATTTCCCAAATTATGTTTAACAAAGGAAGGACATTTTCACAGTATGTCTGATAATATTTTTTATTCTGGAGAAAGTCTTATTTGTTTTATTACGGCTAAAA<br>CAAAAAGCAGTTATTAATTTAAAAACAAACACCATTTATGGTCAATATTATTACCCCCTTGAAGCTAATTTCTTTTCCAATAATGTACAGAACAAACA
ET33-mi30	5:**GTCAAACC**TGCTCTGTAATCAGCTGTAAATCTCCAGCAACTGCTTTCAGAAGAACTACACAGAGCCAAAGTTCACTTACAAGCAACACGAAAGATTGTGGACGATTTAATCGATACAATCCTGAAATCTTAGAGAATTGTTT<br>TGCTATTTGACAACTTTTTTCATTGCTTCTCAGTGATCTACAGCTCTGTGGTAATGCTGATCTGAAAGCAGGTGAATATCACATTTAATAACATTTTAGTCCAATCTCACCTCATCATTTCAGTTGAATGTTTGAATTTAAGGA<br>CAGGAGATATTATGCTAAAATAAATGTGACAGTAAAATTACAGGATTAAGTCATGCATTTCTTTATTTTAAAAGAATGTTTATTGAGAGAACACTAGTGTATATCATAACTCACATTTGGATACACTGTAAAAGACAGGAGGAT<br>ACACTAAAAACATCATCAAGCAAATTCAACTCAATTTTATTTAGTTCCTAGTTTCAGACATTTTCTAAAGC<br><br>3:**GGTTTGAC**TCCCTCAATTAACACGAAGATCGCCTTTGAGCTCTACTACCAAGTAAGGGTAATTTTGGCTAGGCATACAATGATAACCGGTTTCAAGGTTTACCGTGGTTTGGAAAAGTCACAGTTGTAAAACCACAGGAATT<br>TTCTTTTATACTGTTCCTGGGCATATATAATTTTATTTTATTTTTTAACTGTTTTTTTTTAAGGGCAACAGTATCTCCAGCAAAAAAGGACCCTCCACATTAAAGGCTACTGGTCAGCCCTCGATTCAGCAAACATCTGAAAAA<br>CCTAAAATATTTTTATCCAGGGTTATCATACAGTCAAAAAATTAACCATATAATTATGCCTACTGTTGGAAATTGCAAACGCAAGCCTCATGAGGGTGACTTGTACCAAATTGTACTGTACTATACTCTACAGTTTACTCAGTG<br>TAAACAAAGAAAAATCCTTCATGTTGTTCTAACCTCATGTGATGCATTTATTAAGGAAATGAGCAGTGTGATCATTCTTACAAACATCTCTTTTAATGGTCAACCCACAAAAAGAAAGTCCAGGTTTTATAAATGACATGACATG<br>ATCTGTTTACTCTCAAACCTTCCTGTCTAATGTCAGTGTCTGCTCAAAGCCACGAGTCTTCTCTCAATAATGCAGTGGTGACTTTCTTTTAAATTAAAATAATGCATGACGTAATCCTGTAATGCAGCGTCAAATTTTTTGCAT<br>AATATTTCCAATCCTTATATTCAAACATTCCAATGAAGTGATGAGGTG
ET33-mi31*	5:**TCCCCGAG**ACAAAACAAGACACGAGATTTGATTTACGATAATGAGACCTGATGAGATTTTTACAAAATGCTTAGGAAATCTTCAATGATGAAATATATGAATGGAAAACACCCATACTTCTCATCCATGAGCAGGATTTTTG<br>CAGGAGTCCCATATAATGTAATGAAGACAACAACTTTCGTGATTCCACCCCTCTAGGGACAAAAATTAAATTCTCCTTTATATTAAAATAACAGGAGTTTGATAGTAGGAATTTGCACCTTTAATCTTTCAAATCATATCACATT<br>CACCTTTATATATGATTCTGTTAAATGTTTAATTTAATCTGATTGGTTGACAAAAATTTTCCACGAACAGCTGAGGTGATTCCAGACAAGTCTTGACATCTTTACAGATTAGTTTCATTTCACTTTTTTAGAGCTACTACCAAT<br>TAGAGAGAGAAAAAC<br><br>3:**CTCGGGGA**GTATGCATCTCATTGCACGCCTAATGAGAAATATTTAAAATTTATTATCATGGCAGTATGAAGCAGAGTAAGGTGAAAGCTGTCAAAGCGAAGCAAGAACGCTGAAGTGATTGCTGATAAGAGATGGGGAGGGA<br>GGTATTTACTATGCCACATTTGACCGCTTACGTTTTTTTTCTGGTTGTGGTCATTTAGGGAAAAAGCAGCACTGATTTATCAAACTGAATACTTTTTAGGGTTCCGTTATAATGCTGAACTGCGCAGTCTAATAAAACGCACA<br>ACATGTCAAAGCATCGTTGGTGTTTTGCTGCTTTATATAAAACTATAAATCAAGGGTGAATGATGTCATTACAGTGCTTCCCACAGGTTTGAAATATACTTGCGGTTGTAGCTGGATTGAAACACACCTTTCATCCacagcaca<br>taaatggtcaactactagGGGTGTCAAAATGAATTGTTTCTTTAGTGCACCGCGATGCAAACACAAACAATTTGGTACTGGTTCAATAATTATCATAACCAGTTATTACGTACTGACGTCATTTTACACATACAGTTTGTGTTA<br>TAGCGGTAGCTCACTATGGCAAAGGAGGAAGCCGTGAGTAGTTAAAACACTCTAACTACTTAAAAAGCAAATAACGGTGCACAGTTCACTGCTTGTAAGTTTGCTGGACGGTTTTTCACTGA
ET33-mi32A	3:**GTTGTATG**TGACGACGTGTGTAACTGTGTGTTGTATGTAATATTGTGTTGTATGTGGCGGTGTGTGTTGTATGTGATGATGTGTGTAACTGTTTTTTGTATGTCATATTGTGTTGTATGTGGCGGTGTGTGTTGTATGTGAG<br>GGATTGTGTTGTATGTGATAGTGTGTGTTGTATGTGATGGTGTATATTG
ET33-mi32B	5:**TTTATAGT**CATTTTAAAGGTTCAGTAGCCTAAAAAGCGAATACAAAGTCATCTTATTTAATATAGTGTACACTCTTATCCCAAATAGTTGACTTTACTAGAACACTGCCTGCTTTAAACTCTCAGCTTTTACTCAAGGAGAC<br>ACTAATTAACACACATCTACATAAAAACGAGTTAATTCAACTGCTGTTTATGGTGCAAAAGTAAAGTAATGGCGCATTTGCACACAATAAAGTCAACTATTTGCTTAACGAGTGCATCTGTTTGGGAAATGAACAACTTTCGTA<br>AACATCAACATCACAGTCTATAATGCATTTACAGCCTTTATTTATCAATCCTGTCAAATTTCTGAAATACAAAGTTCTAGACTGAGGTTACTGCATGATTCCCCGTATAATGTGCAGGAGCCCGAGCTGAACCTCATAGATGTT<br>TCTAAAGCGTGTGTATGAGCTCCAGAGATGCGCAGGAGGCAGCTTTACCTGCTCTGCGAGCCGCTCCTCATGCTGTGCTCCTCGGGGAAACTGCAGATCCGAGCCGCGGAAAGTTGACCGAAAAGCGCAAAAACAAACCGGAGC<br>TGCGCGGAGACTCTGCGGGATCCTCCAGACTGACTCTGCTCT<br><br>3:**ACTATAAA**TGAAGTGTTTTGCTCTCTTAGTTCTCTTATAGCCTAGTTTCAACATGACTTAGGAGTGTTAGAGCAATTCAATGGCAGATTCTTAGCTTTTACATTTTGTTTAGTAGGTTGCTTTCTGCATGGAAAGCAATGGA<br>CTTTTCAGCTGTACATTCAGGATTATTTTGAAGTCGGATCTGTGATTTCTTTCGGAGCTCTTCCATCGCAAACGCCTCATATTGAGGGTTTTATCCTCGTTTTTAGACTACTGAACTTTTAAAACGACTATGAAATAACTATTT<br>CAGCGCATAAGCTCTCTCATGCATTCTTGTGTATAGCTGATATGAGAAATGCTGGAAATGTGCAATAGTTTAAGCAATGGCAGATCTTTAGGATTTAGATTTTGTTTAGCAGGTTGCTTTCTGCATGGAAAGCATTGGATTTTT<br>AAGCTGTATTGGACATTCGGGATTAGATTTTACCAAGTCGGGTCCGTGATTTCTTTCGGAGCTCTTGCATCACACATACTACATATTAAATAAGAGGATATTATGTTCGCTTTTAGACTACTGAACTTTAAAAATGACTATAAA<br>TGAAGTACTGTGCATGCAAGCTGTTCTCGACCTGACTCTAGAAGTGTTAAATGTAGACTAAAGTTCAACATGAGCAGATTTTTAGCATCAG
ET33-mi32C*	5:**CCTAATTC**CATGATTGCAAACGAGAGCTGATTGGCTGTTGTCTCTTGCCCTGCAAAAGAGCAAATTGCTGGGTTGCACTTTATTTTAAGGTTTCCTTGTTACAGTGTACCTATTCTTTTAACTACTGAGACATTGTAATTAA<br>TTACCTGTACTTAACCCAGGCTCATTCTGATTATGTACTCCTATAAACATTTCTGGAGAGCGCCAAATATGTCCTAGGAGCTAACGTTTTCTTTGCTGTTTTTGTTGTCACGAATCCACCAAAGGCTGCTGTGTATGCTTTTTC<br>AAATCTCATCAATTTCTCTCAAAAGTGCCATTCACGCCTGCTCTTCTCACGTAAATCCACCAGTGGCCTCTGTCGCCTGACTGACCAACCAATCATCCCCAACAAGCCCCCCACCCCCTCAACCTTAACCCTTACC<br><br>3:**GAATTAGG**GAGACATCCTGAGATATATAAACGGTAAACACTTAAACATGGGTAACAATCTAAAATAATTTGAGTAACAATTTACACTATTAACTAGCTTATTACTTGCCTATTGTTATGATATTAACTGCTTTTTGGTAGTA<br>TCATCTTATTCGACATCTCTAAATGTAACTAGCTCTTAAACCCAACTACTACCTAAATTAATATGCAGATAATTGGAAGTTTattgACCTGATAGTTTTAGTTAATGGCTTGTTAATAGTGTGaattgTTCATCAAAATAAAGT<br>GCTACCGAAATTCTGAATGGTTCCAAATATTTTGTATTAAAGTGCTTTAGAATTCAAACTTCTTTTCATTTCCAGAGCTAAAGCAGAGGTGGATGAAGTTCTTGGAACCAAAAGAGACATTTCATATGAAGACCTTGGAAAATT<br>CACTTATTTGTCTCAGGTTTGTCCACAGAGTTGTTTCAAGTGACATGAACAAAGGTTGCTTTTAGCATCCATTTCTTTTAATGGTCTTTTGTGTTGTTCAGGTGTTGAAGGAAACCCTGCGTCTGTACCCGACTGCGCCAGGAA<br>CCAATCGCTGGCTTCATGAGGACATGGTCATAAACGGCATTAAAATCCCTGGAGGCATTTCTGTTATTGTGAGTATCACCACATTCCTTGTAAGACCGTTCTGTTCACAAAAGATACCAGCCTACAGAGGCTAGTCATACAAGT<br>ACAAATCCTTTAAATCAGGGGTTTCAAACTCAT
ET33-mi33A*	5:**GTGTAAAG**GATGATCAAAAGTGTAATTGCTAATGTAATTCCAAAAATTAATTAGCCTACAGATTTAATCACATGCAGGTATTTAATCAAGCATATGTGCACTGTAAAAACATGCATTTACTCAATAAGTGCACTGCAATGGA<br>TTATTAATTTCAGTGTAAGTACACATCAGTTAAAGCCACTTAATATAACGTGGTACCTGAAAAAATCTGCTTCTTTGTGTGTGTGtgtgtgTACTTATTAATTGGTTTGTAAAGGTGGCTACCAAAGGGCTGCTAGGTTTCTGC<br>ATGGCTGTCTTGTTCGGGTCATTTTCAGGAATGTCTGGACAGGAAATGATGTTTATCTTTTACAATGTGAGCCGGACAAACCCTCTGTTCAATTGATTCTTCCCCAAACACTCTCTTGAGGAATGTAAAAAATGCA<br><br>3:**CTTTACAC**CTTAATCCATCCTTAAACCCACTTATACCACCAAACCTGTCACTAAACCTATACTCGTATCTGAAAAGCATTATAAACACAGTAAGTACGTTGTATTTATTTTGATGCAAGTACATAGTATTTAAGAACACTTT<br>ATATAGCGTGGAACCATAACTAATTAGACAATATGTTATGAATTTTTATTTACTTGTAAATTGATTGGAATACACACACGCATAAGAGAGAATGTAAATTTTATAGAAATGCACTTTAATATACCAGTAAATACTTTTTTATTA<br>TAATAACACAAACACAAACAGTAAAACAACAACAGTAAAATTAAATATTAATTATTTACTAAATGAAAATATATTTTTATATATTGGATGTCACAGTGGGTGTAAATTTAGGTGTAGTATGCATATTAATTGTTGTTTGATTTA<br>TATATTTATAATTATGACTAACATACTTTAAATGGAAGAAATATAAAGTATTCATTAGCTGTGTCATTTTGTCTTCAAC
ET33-mi33C	3:**CTGTACAT**ACTTCATACAACAATATACCAGATACTATTTGCTATAGTATTCTGTGGTACTTTAGTATAGTATTTCAAGGACAAATTTTGATGGTATATTTGCCACAGTTTTGTTAGATATACAGAGCAGTAATCCCAATACC<br>TTGAAACAGTGATATTTTTATCCAAGGTTATCATACTGATATACATAATTTTATACCGGCCCATGCCTAAACCCTGCTTCATACACATAGATGAATTTGAAATATATTCCTAATTAATATAAAGCTGGAGCAG

ET33-mi34	5:**CTTTTCTG**CGCCCGGAGCGGAGATGACAGCGCTCGCATTTTCCGCGGCTCTGTCGAGAATCTCTGGTCCCAGCGAACGGACAAGCTCCATCTGAACCATCGCGGGGAATCGGGCTGAAAGAGACACGTAGGCGGGAGACTCG<br>AGGCAAAATGCGGCCGCTGTTCGTGGTGCTGAAACCGCTGTGTTTTCTCGCCCTGCTCGGCTACTGCCCTTCCGCGGTGAGTCTTACTGACTTCACATACACAAAAACTTGACATGTCTGAGTCCGGCTGCTAAAACAGCACTG<br>AGTCAAAGGAACAGTGTGTATGAAAGGCTGAGTGTGGACGCAAAGTAACTAATAAGGCTCTCGGTGTCATTTTTGTGAGGTATTCTTATGTTAATGCGATAATATCATTTAGTGACACCCTAAGTCTAGAGAGAGGGAGTTAAT<br>ACGATGCAATGCAACTAAGCTGGCTGGATTTTATGTAAATATCCATAAGGGAAAAAATGTTTTGGTAAGAACATTATGTTATACTGAATGGACGACATTAAACGATTCAGTTTTCAATCAGTTTTATTTCCGGCGTTTAAACGG<br>TTGTAAGCATCCACATGTACAGTACTGAGCATTAGCTGTTTGTTGCCTAAATGCATAGTAAAAAGTAAAGTATGTGCAACAATGCATTTTGTACTCCGTTTTTTTTTT<br>3:**CAGAAAAG**TTTGAGAGATGTGTGGAGACTCTCGGCGGATATTACATCATCGCTTCACTGTGGGGTTACTCTACTTATCTGCCAAACTTGATGTGTGTAATAAGTTCACGACTTCTATATACTTTAGTTAAAAGCTAAAGACT<br>AAGGTTTAGGCTGTATGAACAAAATGTCCACTATATCTCGTTCTCATAAGAAACGAATCTGACTCCTTTAACCTGTTTTATAGCATATTTTAAAGAAATTATAATAAGAATTGCAATTTAATTATTCTTTTTAAGGAGGCAAAA<br>ATAACCAACAGTACATTAACTGGTAATTACTTAACCATATGTAAAACTTTATTTTTAAATGATATAATAACAAGCATTATCACACATTTTCTAAACGCCAAATATCATCAATTATATCAATCATGTTTCTACAATGATTTTAAG<br>ATTAAAGTTTCTTGTGACAATTTCGTGACAATCAACCAAAATGAATTCACCAACATGAATTACCTCAAATAAATCAACCAAAATGAATCAACCAAAATTAATTTACCAAAATATATCAAGCAATGAAGCAAAATTAATTCACCA<br>ATATGAATCATTCATTCATGTTCTTTTCGGCTCAATCCCTTTAATAATGGGGTTGCCTCAGCAGAATGTACAGCCAACTTATCCAGCATATGTTTTACACAGCGGATCCCCTTTCAGCTGCAACCCATCTCTGGGAAATATCCA<br>TATACACTCATACACTACGGACAATGTAGCCATTCCAATTCACCTGTACTGCATGTCTTTGGACTGTCAGGAAAACCGGAGCACCTGGAGGAACCCCACGCAAACACGGGGAGAACATGCAAACTCACACA
ET33-mi35A	5:**TTCGTAAC**ACGGATGTGCACAAATCTGTCATGCTTGACATCACCCCAAGTTGTGAATATTACGCGTATAGCAGGCCTTGTTTCCGAAAAAAACAATGGTGGATGTCAAAGAGCCAGCAACCTTTGGACTGAAGTTAACATGA<br>ATTGATCCATCACTGTCACAGAAAACATGGCCAGAGGAGGGGCATCATGATGACTCAATTTAAAACCTGTCATCCTTCAGCTTCCTGCCGTTTATGAATAGGGCCATAGTGAGCCTTTTGAGGTCGTAATGCGTGTGAAGTGAC<br>CTTGTCCCCTTGACTAACCTCCTCCGCACACACCAATCCCCCTCCCCCAAATTCACCTCCCTGAGAGCAAAGAGGACTTTGGAGTGGTTTTGACGGCTGGTTGAGCCCAAATGTTGGATACAAGGTTGGCAAACAAGCTTTTGG<br>CGGAGTGTTTGATGTGGTCGGGTCCCCTGCAAAACACAAATGAAGTTTGAAAACTGACTCCCTGATTATCATTTACCATGTGTCTTCACGGAAAATCCAAAACAGACCACAGACGTGGACTCCATCAGGCAGCAGTGAGGTTAA<br>ACAGCAAATTTGTAATTATATCAAAATTAGGAAAAGAGATAATTATAGCCATACAGGCTTATTTGAGCAGTGACAATCTTGCACAGTCCTCCACCACCGGGCTGCGAATTGGAATCGGTCTGTGGATCAATTG<br>3:**GTTACGAA**TAGTACCTTCAGTGTTTTTCTACACACACACA
ET33-mi35B	5:**ACCACGAA**AACCCAGCACAAACACCTGAA<br>3:**TTCGTGGT**ATTGTAGTTTTTTATTTCACTAGTCCAGTTGTAATTTGCAGTATTAAAACTACAAATGAGACCAGCATGCAACAGCCAATCAGCTTTCTGTTGTTGAACTCAGTGCATGCGGTGAATGTGACGTCATCGCTGTG<br>TTTGCCAAGGTTCGCTTCTGAACAAGCTAATCACCTCTGAACAAGCTAATCAAGGTCTTACTAGGTATACTTGAAACATCCAGGCAGGTGTGTTGAGGCAAGTTGGAGCTAAACCCAGCCCTCCAGGACCTAGACTGGTGACCC<br>CTGGTCTAAACAACCCAATCAATTGTGTTTTGTCAATATTTATGTTTGTCTTGTGTACTTGTCTTGTTTCTAAATGTGTTTAATTTGAAGTAAATGTTTTAAACTCCTCTGATGTGGTTATTACTCTCTGTTACTGATATTTAA<br>GGTCTTTCAATTGGTCAGAACAGCACCTAATACTGAGATACCTAATATAGACACCAAATAAGACCTACAGTATCAATGATTCAAAAACTTTGTCCAAGAGACCCTTATTTAAGCATTTGAAATCTTTAATAGCCCCTTATTCCC<br>TACAATTCAGTGTTTATTTGTCAATATTTATGTTTGTTTTGTGTCTTATTGTAGTATATGTTGTATGTTGTATTGTTGTTGCTTGGTTTTCGTAGTATTGTAGTTTTTTATTTCACTAGTCCAGTTGTAATTTGCAGTATTAAA<br>ACTACAAATGAGACCCTCCAGCCCTCC
ET33-mi36A	3:**CATCCCTG**ATTGTGTAACAGCTTTGTTTAGCTCAATTGATCATGTTAATAGTGTTATGCGTGGACGGAGGGTGAAGGAGGGTCATTTAATTGGAGAACCTTCAAGGTTGCTTGTTGCCTGGCAGAAATCCAAGGTAGATTTT<br>ACCTCAGCCCAATGTGCTTTTTCTCCTCCTTCCTAGAAAGGACAGAGGTGCTTGCTGAGGAGGAAAACATGCTGGCTGTGCAATACCAAAGCGTTATAATCAACGTCAACAACTGACGTCACAAACCAAGCATGCTTCATGAAT<br>TTCAAATGAACCTTTGCTATGAAACGTACACATCACACCTTCTCAGCAAATCTGTCGCAGAATAAGTCAGTTGACACAAATGAAGTGTTGGCAATGTGGTCTCAAAAACATTTTAAAGCTTTCAATTAGATAACCGAAAAGTGG<br>AAAAGAGAGCTGAAGGACATTTATGTTGCAGCTGTGCCACAAATGTGAAGCACAAATGCTGAAACACGCATCAATGACATTCAAGGACATCTCTTTTTACAGGATGAATTCCAGCTTAGGCCAAAAAAAAAGTAATTTA
ET33-mi36B	5:**CATAACAG**TTCATTGTAAACTTTTTATTTTGCAAAATAGTTAGTGTTAGTCTGATAAGAATGACTTTAAAATGCTATGAATTGCTTATTTTGAAATATACTGTAAGCATATCCGCTTGTTAAGTTGTGACGTTTGGAATACT<br>GTTTTCGGGTCCAAGACGCTAATCATTTGAATTGAGAAAATATTAGTTTATGATTACTTTTTAGTCATATCTCACATTAAAGTGAAATTTATATAAAATCTTGGTGTACACAACAGTCTTTGGACTTGCTGTGTAATGTGAAGT<br>GGAATGCTGACAAGGGAGATGCGGATCTAAAAGCAGGTTTATTTAATAGAGATGATCAGGCAAGCAACGGTCACAATCAGGAGCAAACTGATGTATACAGGCAATCCAAAGTCATAGTCAAATACACAGGCGATTGGTCAG<br>3:**CTGTTATG**TAAATATGTGATAAATATATCAATTTCAAGTTAAAAGCTATAAAGGTTAAACATATTGAAAAATAAAAGTTAAAAGAGTTGCAGAGATTTTGTCATGGCAGTATGGCATGTACAAAAAGTCAAATCACATTTTC<br>TTGTCTTTGTGTTGACATAAGTGCAGCAGTTTTCCTTATTATACTGAGTACATTTTAATTGCACTGGCTCAGCTCAGCGATTGTCAACACCATCTTCAAGTATGCTTCATGTCTGAGTCGGTTCTTCACTTTCAGTAGCTGCAT<br>AGAAAGTCATTTTTGTTGAGAAATGATAACTTGCAAGATGCTACATAGACTGGTGCATGTAAAGTCCTGTCATTTGGGATACCTTTGGGATATGCAGATAATAATACATAGTTCAAAATAATACAAATAATACATAGTTCATAA<br>CATATAGCTCTCACGTAGTTTTTGGTTACAAAGTTGGTAAAAAAGATGTTCTTGATGATTGAAACGCATGCTTTATTCTCGGGCACTATGTGCGACTACTTTTCTTGTGGGGCTGCTGAATCCAGGTGTGATTTTGACATGAAT<br>GAAGAGACTGGAGGGCGATATGCTACAGCCATCTGCATTCAAAAGGCGCACGTGCCTGTAACCTAAAGCACA
ET33-mi37A	3:**GCTTTAGC**ATTCATGCAGCTGCTTTATCCCAGAGACATCCAAAGGCAGAGGCACTAATCGCAGCTGAATGTTTACACATTTGACGACACTACAAATTCGGTGGATGGATGAGTATTAACTTTGATGAAAACAATTTAGGAAC<br>ACTTTCGCATGTAGTGATGTACATAATATGTGTGTGTGCTGGCATTCACCAGAGCACTGCTGCACTTCACGTGCACACGCAGCAAGCAACTGAAGCAGAGCTTGAAGCTAAACAAACTGCAGTTTATCATAAGCATTTGATCGA<br>TCATTTTTTGCACAGATGGCATTAGGAAGGAACATAGAAACGATATCTGTCTAACATCTAGCAGCTAAATGTGTATGGAAAAATATTCAAAGGCTTTTATTTTTATAAACAGCACAGATGTGAATGAGTCTGACTGTACTGATT<br>GGCTAAAGTAGAGGCGAGTTCTAATCGTGAATGCGCTCTTTCTGGAATTTTTAGTTCTGCGTTTACACAAAGCAGAATTCCAGCATATTACCAGTAATGTTACAACTCTCTTTCTGGAAAATTGCCGGAACTAATTTATTGGTA<br>TTTTCAAAAAGGG
ET33-mi37B	5:**TCAGGACG**TCAGGACACATGATGGTGATGGTGAAACCCAGACCGTGAGCCAAGAGCTTGATGGATTTCTCGCACCTCTGCCTTCCCTGTTGGCCAAATCTGTGTCCACAGATCAGGGTAACAGGAAGAGGAAGCGGCAGAGC<br>GGCAGCCAGCAAACACCAAAAGATGAACGTCCGTCCACCTCATCTAGAAAAGCTCTCAAACGCTCTCGCAAAGGTCAAAATGGCTTATTGAAAAAGTGATGATGATGTTTCAGTAATGCTCTTTTACAGTTTTCTCTACTGCTG<br>CATGTTTTCTTTGAACACACATTTCTACTGCTCCTCT<br>3:**CGTCCTGA**ACAGCTGCCTGGATGAAAGCAGAACTCCTGGATCTGGAGCGGCCTGAGACAACGACAACAACAACAACATATCACGTTTATCATAAGATTATAAGAATTAATCAACAATTAACTTTAGTAGCTGCATCTACAGA<br>GAATAAAAGACATCTGATCACTTATATCGGGGAACAGTTGTGTTGAAATGGGTTCATTTGTACCAGTTTATTTACATCACATTTCCCCAAACATTACACAACATCATATCATGATTGATCATAAACAAATAAAAACTAAAACAC<br>TCACTGCGGTTTTCACCCATCTGACAAACTCCTCCAAACTCCAGCAAACCTGAAAACAAGAACAAAGATCAAATATCAATAAAAATGTCAATAAAAACAAGATCAAAATCAAAATAAACACAACAAAAGAGCAACAAAACGTGT<br>CAAAAGGAATAATTTCAGCTCTGAGCGTCGATCTCCTTGTAAAATAAAAGTCTTCTGTCAGAAATTCTCTGGTCTCCTTGAAGAATCGCAGTATCAGCACAGTAATCCTCAGCAAATGTCTCAGAAATTCGCCTCTCCTCAATC<br>AACAGATAGCGGCGAGTCTCGGTGTTTATATATGGAGTCAAATCATAACACGCGTTGCTATAGCAACCACAATTTGAATCGCCAATCTGAATCACGTGAGTAAACGATTCATCTCATCTCAACATATAAATAATAAATAATTAA<br>TAATAATAATAAACATAGTTAAAATAATAATAATAATAATAATAATAATGAAGTTTTAAATCAATTAATTTATAATTCACGAAACTGTAATAATAATGCTAAATGAGCAAGATTGGTTTGACAAGGTTTTGACTTTGTAAAATG<br>TACAGCTATTTAGGGAATTAATAAATTAAAATAATAAAATTACTCATATATTTTCTATTA
ET33-mi38	3:**TTTCAATC**CTGCGTCAGAGAAACTCACTGCACCTGCTCCACCCATGAGAGCAGTGCAGACGTTAAGGGCAGGGTGAGTTCCATGTTTAGGGAAATTTCCACACTCTCAGTTAAGGCTCCGCAACTCTACATGGGGTTTCACT<br>CAAATATCATAATGAGTAATTTCCCCTCCAGGCCACCACTTCTCAAGAGGAGCTCTAGGGACAACTTCAGTAAATAATCGCTATTGCAAAAACTACATTTAGGCTATGAAGGGTTGACTTGTGCCAGAGAGAGTTGTGTTTAGT<br>TGTGGGAACTTTGTTGACAGGAATTTCTGAAATATAAAAAAGGAAATTTCCTGATACAAGTTAAACCAAACTTAAACCTCTACAAAACAGTACACAATCCAAGTCAAAAGGCACAAATTTCACTTTGAAATATCATGTAACTCT<br>TTGGCAGATCAGTTTTTCTTCAATAAATGCCTCTCATTAGCCAAGGTCTTTTCATAAAAGAGTCTGTCCCCTTGTATTAAGACTCTATTAATTAGAATTATTTACTCTGAACTGACATCAATACAAGTCACTTGCACCTGGACT

ET33-mi39A	5:**CTCATTAG**ACTAATCATCATTGGTGCAGTGCTTCACTAGTGGCTCTGGGAAGCTTTTCCTGGC<br>3:**CTAATGAG**TGATTAATTAGGGAGCAGGCTGTGGGGAAGGGGGGGGACTGACCTCTCGGAGAGGCCCAGAGAGAGAGATAGAGAGAGAGAGAAACAGAGAAAGTGTCTTATAACCTGGTAAAGAAAAGATTCAATACAATGTTATGTGCAG
ET33-mi39B	3:**TCTAGCAT**TCTTGTTACTGAATTATATTCACATATCAAAATGTTGCAGAATGAATCGTGGTGTTTCCATGGCGCAGAGGTGAAAGCAATACATTTATGATCAATAAGCAGCCCCAGATAGCACAGACAGCTGTCATCTCTTACCTTATGCTGTGTGCTTCAGGGCCATTCTCCTCTGTTTTTGAGATGATGTGCATAAGTAATCCTTTTATATGTCTGATTTGGTCCAAACACAGTGAATTATGTTTGATCAGACAAAAGAATAGTGAAATATGAAAACAATAATCGATCGCTGTGGCTTGTACAGCACCTCTCATCAGTTGGAATACAATACCTTTTGCATAGATTATGGTAGAGACATTTGTCTTTTTTCCTATGATTACAGACATGTGCAGGAACCACCAAAATTAATTACAGCAC
ET33-mi40	3:**GTCTTGCT**GTATAAAAGAACTATTTCAGGCAGTGGCACTGAGAAGGTCAGATGAGGATGCCAATGGAAATGCTGAAAGGGAGGGAGTCTGTGTGCGTCTACAGCAGTTACGCAGCTGGAAAACATACTTTTCATTGGTTTGCCGGCCAAACCTGAGCCTCAGACGTTTGCACAACCACAAAACATCTATTTTTAGCCCTCCTCCTGCAGCAC
ET33-mi42	3:**ACATTGAA**CATCACAACTGCATCACTGAAACCCATTTGAAGAGTATCAACACTCACGCTGAACTTCATTGATCAGGTCCCTGTATTTGCTCCTGATCTCTCTTCTGCGTGAAGGATCATCATCTTCCTGATTAATACCAGAGGGTCCGCCACCAATATCATCTTCA
ET33-mi43	3:**CATTAATG**AGAGCTTCATTGAACCTCAGCTCATGATCTCAGCTTGTGAATATTTTGCATTTGTTGCTGACAAAACACTTTTCAAATGGATTAATGTGAATTGATAACTTGGCCCATATTTATATAAATATATTTAAAATAAAATTAATGTTACATCTTAAAATTTTTTAAGGCGGGTTTTCAGTTCAAGAATTTTGGTTTATACTCTGTTTCCACAAAAGCATACTGACATGCTCATCAAGGAGTTTACAGAGCATTAGATCTGCTGCAAACTCATATGAAATGCGCGACATAGAGAGTGAAACTCATGTACTCAGAGTTTTAATGTATCTTTTAATGTAATGCAATTTACCACACCTTGTCTGCAACTGTCATGATGCGTGATATCTTCTGTCCAGAAGCACCTCCTTTGCTGCTGAAAACCTTCTGTAGATCAGCAGTGTATTTGTCCAGCTGCGCGAAAAACTTTGACAGAAGAGGCACAGTCGTTATCCTCATAAATTCTGCGTTTATCTGAAAGAAATAAAGCAAAGGTAGATGATGAAATGTAGATGTATTGTGGAGAAATGAATTGAAATTTAGAAAATAATTGTGTAAATATCACCAACACTTACTTCATTGACTGTGAACAGGGCAGGCCATCTACTTTGTAAATCAGAAACCATTGGCAAGTCCTCAACCAACTCTTGTCGCCTATAGGAGAAGGTCAAGTGCATTTTTTCTTTTATGACTGCCTCATTATTATTTTTTTTCACTTCAGTTAAGAGAGCAATTCTTTCCTTCTCCATACTATCAGCTGTTTCTCCTCTT
ET33-mi44A	5:**ACAATTAG**CATCCAAATTAAGAGTATAAAGAAAGAACACTGGCCCTGAGATTTATTGCCCTTCCCAAAATGCACTGCCACAAAGGGCACAGCAGGGTGACTGGGTTCCAGCCCAGTATTGACAATCCAAGTGTTTTCTTGGCTACAATGAAGGGTGTCTATTAAAAAGTTCTAATACAATATGCTTTTCTGTCATTAAGTAGCCATATAAAAATAGCATTGCACATATGAATTCTAAAGACACATATTTATGTTTCATTATTGTTATTAACTCTCCAGAAATTTGAGTTTGCCCTCCAGCTATTGCTGCACGGGATTCAAAACTGGTCCCAGAGGTTGGCCTGAATGAACAGTGGGTCACAATGAGCCCTTTCCCTGCTGCTGGAGCAGCTGCGGTCCTTTTGATAGAATGAGGTCAATTCAAGAATGGAGATGGCTGGAACTGATTGAGGGGCCTACTCGAAGGAATAAGGTACAAACTATTTTGTATCGGTAGTAGCACCCTCCCCCCATGTTCCCCTCCTCCCGCTCTTATTTCGTTGCATGTCGGTGTCAGGAATAAATCATTTAAATTGTTCTCTGATT<br>3:**CTAATTGT**GCAACACATAGAGTTGCAAATAAGGATTAGGGCCACAGCGGGTGTGCTAGAAGCACAGTAAAGGACATGTTAAATGGATATTAACCAAATAATACGTGGCAGCATTTACTCATCTCAACTTACTTAGACATTTAAAACCGGAGAAAAATAAATAAATAGACTTTAATAAAACTAATGTCCTTATGGCAAAAATCCACCTAGACAATTAAATTAACTCAACTACATTCATATAAATAGATGAGGTAGAAACATCCGCTTAAGGCAAAAATTCATTCATTTTCCTTAGGCTTAGTCCCTTTATTCATTAGCAGAATGAACCGCCAAATTATCCAGTATATGTTTTATCCAACAGATGCCTTTCCAGCTGCAACCCAGTACTAGGAAACACCAATGCACTCTCACATTCACACACATACACTGCTTCCAATTTAGTTTATTCAATTCACCTATAGCGAATGTCTTTGAACAGTGGGGGAAATCA
ET33-mi44B	5:**GAAAGCAA**GATGCAGTAGTATTAACACTAGTTACTCAATGTCAAAACAACCGCACATTTGAAACAGGAGTAAACTCACAACGCTGACACGTGAGAAATGTGTTCTCTTTTTCAAATCGTGAGCTTTACGTTTTCAAACCATAAGATTAAAAGCATTTACTGTGACGATACAAACTCAATTACAATGGCAAATATATATATATATATATATG<br>3:**TTGCTTTC**TGATGATTTCGATGTCATACGATAGCGGTAATGCGTCAAAGAAACGGTTAAAACCTCACAAGCTCTTGAAAAGCACCGACGTCTCTTTTTTCCGTGTCAGAAAACGCCATGCATCAGTGATCAGCGATTACCGTGGTTTCTTGAATCAGCTGTGGCACATTAGTACAACTGTCTCAGCTAAACTTTCTGCATGGCTCACGATTAAAAGTTTTAAACAAACAAAATGGTGTGTAGTTATGATGCATGTTGTCCTATTTCTTCATTACAAATACTGTGCCCATCTGCAAAATTGAAAAAATAAAGGCTGATAATCAGTAATAGTCCTATAAAAGGTGTTAACTGTTAGTCAGTAACTGATTTTCTCTAACCTTAAGTTGATTATTTTTGTGATTTATTGACCTCTGTTGTATTTACTCCAACATTATCTAGCCACTTTAAATATTGTTAATCCTGCTGTACTCATCACTGTATACATTACTGTTCTCTG
ET33-mi44C	5:**AAAGCGAT**TGATGTGTGGGACTTTGTCAGAGCTTGACAGGAACTTTACAGTACACAGTTCCTGGATCAGTTTCCATTTCACAAGTGTAAGTGGGTGCGATTAAAATTGGATCTAGGAACGAGGAACCTGAAACTGCTGACACACTAGTAGAAGTAGGGGTGGGTATTTCTAGGAGTGGGTGGAGTGGACTAACGTGCACTGACAAACAGTGGCGTAGTGCAAAATGCGAAGGCCCCCCTGCAGGGATCACCGACGGGGCCCCCTGAGGATGGGGGGGTGAGAGACAACACGGGGAA<br>3:**ATCGCTTT**TCTGATCCTTTCTTCAACAAATGGCAGGTGAATCCCTCATTGTAAGCGTGTAGATCGCTGAAGCGCCCGTCAGAAAAATGACAGGGACACAGCTCAGGGCTGGGGCTATAATGCTGAGGTATTTTTGCACATATAATCTTCAACCACTGACTCTTCACAGTCTCATCTTTGGGTAAAAGAAAAATAGCACTAACTTACTCCTCACACTTCAGAGCACAGCGTCTTCACGACATGGTTCAGCTGGGATCTGCTGCAGTGTTTGTCTTCCTCTTTTTTTCTGCAGTGTTTGTGGGCGGAGCCTGGGGTTTCAATTTTGCCAGGTATGCACACGCAACAAATAGGCGGGGCTTGAGTCATCGATGTCAGGCTGACATGGCTAAAGACTCGTTACCCCCACAATTCATTTGAACCACTCTGAGTCGACTCTTTTATAGATGAATCAATAGTTTTAAACGGCGCACTTTCAGATTTAAGCCTTAGCTGGATATTTCACTTCACTCAGAGCGGTGCTACATGAAAGTCTTTCTCAAAAACTCATAATAAG
ET33-mi45	3:**CACCCAAC**ACATTTGCAAGGTTTTACAATCTGCGAGTGGAGCCGGTTTCCTCAAGGGTATTAGGTAACCCTTGGTGATTGAGGAAACAATTCGGTAGGGTGTTGAAACACGCTTGCTGCGCCATTTTCCCTAACACGGAGATACGTGCGCCTTTTTATCTGTCAGTAAAGTTCCCCGTCAGGTGAGCCCTGCAGATTCCTCCGTGGCCCCCAGCAC
ET33-mi46	5: **GTGTGTGT**GTGTGTGTGTGTGTGTGTGTGTGTGTGTGTGTGTGTGTGTGTGTG
ET33-mi47*	5:**CTGCTAAG**TGACATTACATTGCGTTTCCCTCTGTCTTGTTTATCCGTGCTTTACCCTAGCCTTGTATATCCGTGTTCTCCTGTTGAGCCGCCTGCCTTATGACCTATTGCCTGTATATTGACTACAATTCTGGATTTGCCTGCACATACCTGTTTACACCTGATTTGACCATTGCTTGCCTGACGCTGAATAAACCTGAATATTAAATCTTACCTCCAGTTGTCTCTGTCACTCCCC<br>3:**CTTAGCAG**TAAACAAGActcggccaacAGGATGGGTGAATGAGTGGCTTATGAAGGGTGTCTAATCAGTCTTTGAAAGTCCTCAGGTGTGCGAATGTAATCAAGCTAGATGAGTGAGCAGGCGTGAGCGTCTGGGAGAAGTGCATGTATGTCTATGTAGTCCAGAGGAAGGCGGAAGTGTAGTCCATAGTGTTCGTGTGAGAACCAGCGATCTCCGAAGAGCGATCAGTGGTTATGTGACAGGTGTTGTATACTACGCTACAAAAACCTTGTAGTAAACTTCAGTACGTTTTCTGATGTTTTACAGACTTATTTCTCGTTATATGTATTTATTATTCATTCATTCATTTTCTTTTCGGCTTAGTTCCTTTATTAATCTGGGGTCGCCAAAGCAGCATTAACCGCCAGCTTATTCAGCACATGTTTTACGCAGCGGATGCCCTTCCA
ET33-mi48A	5:**ACTCCGAG**TTTACACTTAACCTCCTTTCTGAAACAGGCCCACGGTCAGCGATTCAGGTAAATAAAAATCTTTACTTTTCAATTAAAGCCACCACAACACTGTCGCTGTGTGTGTGTGTTCTCTTCTCCCTCGACTGCTCCTCCTGTTCTCCTTAAAAAGGGAGTCTCTCTGGTCCAATCACTAAAAAGACAGGTGTTATTTATCATTTCTGATTGACCTGCTGATTAGCCGCCAGGCTCTGAGCATACTCTCCCCCCTTATTGGGTGTTCGATCCCGCTCACCTCCATACCCCCACCGCCCGCCTCAGGCCGGAAAGTCTTCCGGCCTGCAATCACCCATCACGGGTTGGGCTGGCCCAGTCGCTGTGGGACCCAATTTCAA
ET33-mi48D*	3:**GTAACCAT**TCACTTCAATGGTATTTGTTATGCCTACTATGGAAGTCAATGGTTACAAGTTTTCAGCTTTCTTCAAAACAATGGTTACAGGTTTTTAGCTTTCTTCTAAATAAGATGAATAAATCAATTTTACAACCTTAGTT

	TCCTTTTTCTCTTTAACAAAAAGATGTTTTGAAAAAAGCTAAAAACCAGTAACCATTCACTTATATTATGTTTTGCCTACCATGAAAGCGAACGGTTACAGGTTTTCAGCTTTCTTCAAAACATTGTTCAAGAAAAAAgaaaaa<br>ttaaggtaaaaaataatagGTTTATTCAGTTTATTTTGAATAGAGCTAAAAACCTATAACCATTGACTCCTATTGTATTTTTTACTACCATCAAAGTGAGCTGTTACAGGTTTGCAGCTTTCTTCAAAATATATTTTTTGCTCA<br>TGAGAAAAAAAAAGGAAAAGGAAGGGAGGTTTGGACCCACTCAAGTTTCCTATTTGGGGTGAACTAAACCTTAAAAATACAAACTCGACAAACACTTTGCTTGTAGTAATATTCACTAAACAAAAATTATTCATATATATATAC<br>ACTCACCCAGATCTTGATGCTGGGTCCAGTGGCAGCGCACAGCCAGTAGCGGTTGGGGCTGAAGCACAGGGCGTTGATGGTGTCACCACCGTCCAGCGTGTAAAGGTGCTTGCCCTCATTCAGGTCCCACAGCATGGCCTGTCC<br>ATCCTAAAATACGAGCACAG
ET33-mi49	5:**CATATCAC**TGGCACTACACTTCCTACACTTCAAGAACTGTACTCTTCCAGAGTGAGTAAAAGGGCTCGCAAAATCACTCTGGACACCTCGCATCCAGCACACTACCCGTTCGAACTGTTACCGTCTGGTCGGAGCTTCAGAG<br>CACCAAGCACAAAAACAGCCAGAGACAGGAAAAGTTTCTTTCCTCAGGCCATCTACCTTATGAACAGTTAAATATTCCCCACCTGTGCAATAAATATGTGCAATACTTTCTTATACGCACTTGTACAAAGCACCTTATATCAAT<br>ATACAAAGCAATTCTTTCTCCATTCCCATTTGTACACAGCAC<br>3:**GTGATATG**TTCAGCAGTCCGGACTATTTGCTGTAGTCTAGGGCAGGGGTTTTCAAAGTGTGAGGTGCGCCTCCCCTGGGGGGCGCTAGAGCATGTCAGGGGAGGAGCGGGAAAAAATATTATATAATAAAAATATAATTATT<br>AAGTTTAATTATTATATGTATTTTTATTATATTTAAACGTTTCAATTAAACAAAGCTAAAAAAAAATAATACTTCGAAAATAAAAAAACCTTTTTTACCCAAAAGGCCATAGCTGTGAATTCGCTTCTGTTTGGCAAGCCCGCC<br>AATACAGGTATATGCCTGCTAATACAATAAATCGGTTTCTGAGATCCCCCGATTCAAAGCCTTCAAGTTCAGGGCTTAAACCCAAAA
ET33-mi50	3:**TTTGCATC**TTCCTTCCTCTGTTGTTTTGAAACAACAACTAACCTATTGACTAAAATGTGGTACTGCAGAAGCAACACAACTCACTTTCATTCATCTTAAGCGAGCTGTTTAAATTTGAAAACATACTTTTTTTCTATTTTTT<br>CCAACACAACTCTTTTGGTTTTAATAGACAAACATAAGTGAATTGTTTAAGTTAAACAGTTATCTTCTTTGGACCAACTAAAGCCATAATCCATTTTTTGAGTGTAAAGCTTTTAAAGAATTCCCAATGATTTAGTTCAATTTG<br>GAGCAGCA
ET33-mi51	5:**GGCTTAAA**TCTGTAAGAATACAGTGTTTAAAACTGTTGATTCATCTATAAAAGAGTCGAATCATAGTGCTTCAAACGAGTCGCCTTGATACCGACTCATTAGGTGTTTCGCCATGACGTACGAACGAAACAAAGTTTTTCAC<br>GTGCACGCGCAAACCCGGGAGATTTCAAACCTGAGGCCCCGCCCTCTGACGCAGTAAACCCAGACACACACACACACACCCCCACAAACACACGCACACAAACATGCCGGTCGATTGAAGTCACACTGCAGATGGATATATTGA<br>GTCTCTACCCAAAGATGAAACATCAGCTTTATAACCAAGCAGTTGGAAACTTCTGGAAGCTACATGCTACAAAAAATACTTCATCTGATTTTGTTAAAGGAAGGATCAGTAAAGAGTAACTGATGGACGTCGGGATGGGTTTCT<br>TCCTACTTTTCTGAAGTGTAAGTACGTGCGGTTAAAGTTGCCTCGTTGACTCTAGCTTGCAAATGTATTTAGTTGTGATTTGTTACTTGTAACCGCGTGTACTGTATCAGGTTAACTGGATATATTATCTTATCGCGTGCAAAG<br>TCACGTTAAAAACGCGACGCGTGCTGTTTGTTTATGGAGCTCCGTGCTAGAGTTCTGCTCCCGCGATTTATTC<br>3:**TTTAAGCC**TTAGCTGGATACTTCACTTCACTTAGAGCTGTGTTACACACTACATGGAGGGGAATTTTCAAAAACCCATAATATGGGCTCTTTAAATATGTAGCGCATTGGCCCTCTAGACCGAGTTTGGACACCCCTGCTCT<br>AACCACTGACATTGCTCACAATACATTGTGCATTGGGTGGGAATTTGATTAGAACTACAAACGAGGGTGAAAAGTGTAAACAAACTACAAACATGATTCACACACAAGACCAACCGTAAAGTAGGCTTCTGTAAAGATGTTTGT<br>ATGACTGTTAATTAATATCTAGCCAACTGGAAAATATTTAAATAGTGTGTTAGATTTTAACTGCAACAACATTACTAAACTTATATAAAGTGATGTTTGAACATTAACATCTGAATGCAAAATTATCCAAAGACCTGAGAAGAT<br>TAACCTGCTGCTGCTTCTCCAATAAGGTAGGAAAATAAATATGAAAGAAATGTGGATGATGTGTGGATGATTGACAGGGCTCATAACTAAGCAACATAGCGATCTGATAACAAGGAAGTGGTATGTCCCAAAGCTTGCATACTC<br>TTTTGTTACACACTCGAAGGAACATATTTTTCCCTCACAAAAAAGTACATACTTTTAGGTCCAAGTATAAGTATACAAATTGGGACACAGCAATAAGTCTGCACTGTAAAAAGTATGTTAATTAACCATTACTCTATATTGTGA<br>TGCACAAGTGTTTTCCATTTAACTACAGTTGCATTTCATTGCATCATGGGTGTTTTATCTCTGCTCTGTTGACTTTTGATGTTAGAATTTTATTCAGTTTAATTTGTGTTTGTTTCTATAGCGCTTTTACAATG
ET33-mi52	5:**TCCATATC**AATCTACCCAATGACCTACTTTTGAATATGGGATATTCTATAAAGTGTGAAGTCAGTATTTCCAATTGGAACTATAATTGAAGTTACATGAGGATTATAAAAAGTATAAAAAGAAAAAAAAGTATTAAAAGTCC<br>TAAAGAAGTGCTACTGAAAACAACATAACCAATTCCACATGAATTAAATTACCTCACAGAAGCCGCCTGTCTGCCTATTTTTTTACTGATTTGATTTTATATCTCATGCTGGTACAGACCAGCAGCCAAAAACATACATAAAAC<br>ACCCATTTGTTTTGATACTGATTGATTGCGAATAAAAAAAAAATTACCAAAGTTTGTGTATTACAACTCCAAACCAAAGGAAACACAAACTGACTCTTGAAAAGTTCAAGACTACACAGTTAAATGAACAACAAAGTGGGATTT<br>CCACTTCTAGAACACTTGAATAATAACTTAAAAATGCAAACAATGAGCACAAACAATTCCAGTAATGTTTTAAAATGCATTCTTGCATAAAGCCATATTATATTTCACAGGGGTGTTCAAGACCCTTTCAGTTTGTTGCATTCG<br>GTTTTGATGTG<br>3:**GATATGGA**GACACAACAGGATACTTAAAAACAGTCACAGACAGGAGGTCTGGAGATAATCAAAACTGAGCTCTGATCCTCCATGATGCATAAAGTCAAGTGTCTTCTATAATCTGTGTTACCCAGCCTCACTGAAACTTGTT<br>GAAACTCTAAACGCAGCTTCAGAGAAGGCAAACCGGTCGGTTGCTGTTTGCATATGATGAGATCATAAACTTACCACAATATTTACATGTTCCAAATGGGCGTTTCTTCCCATTGGAACAACGTCATGAAACAGTTGGTGAGGT<br>AGATTGGTTTTGAAGATCTTGATATATTTCATAATGGCAAAAATACCCATTTTAATGCAATCATTTGCACCGGTGATGTCTAGACCAGTGGTGTCCAAACTCGATCCTGGAGGGGTGATGTCCTG
ET33-mi53	3:**GAAATATA**CATCTGCTTTCTGGATGAACAAACCAATTTTTTTAATGCAACTTGTACTTTGCCACCACTCAGGCCTTCATTTTACACCAATACGCAAAAACAGTTTTCTAGCGCCATCTTGTGTTCACAAACGAAACACCTAT<br>TTTGGTTCGTCTTTTTCACTTCGCAGAAAATTTACGTCCATTTCAAACGAGTCCAGGTAAGTAAGTTCATAAATAATATTTTATTTTCAGAGCATCTGCCATTCTCTCAATACAGCTGAATAAATACACATAGCAATTATACAT<br>TAATTCATTTTTTTCTTTCTGTATATTTTACACTCATTTTTTTTACTTGTTCAAACTAAAATGAGCTGAAACAACACAATTCTTGAGATTTTATTGGGACAACTTATTTTTTTATGTTTAATGCACAAATTAGCTAAAAGTGTT<br>AAGTTAACTTAATTGATTTGTGTTGGGATAACATGAATACATTGTGGAACCCTGCATTTCTTATAGTTTACACCGTCTTTTGAATAAGACCATCAACCTGAGTCATCTCATAGAAAATCACCTTTAAGATCTGATATTTAAAAA<br>ACAGAGATCTTCACTGCCTGGCTGAGATACAATATAAAGTGGGTCAGA
ET33-mi54	5:**AATTCGTA**CAATCTAATTCGTACAATTCAGTACGACTTGCTCATCCCCCAGTGACGGTTGGGTTTAGGGATGGGGTTAGGTGCCACGCCTCCTTTTTAAAATCGTACAATTTCGTACAACTGAACTAGCCTGAATTAGCCAC<br>TAAACTGGCAAAACGTAAAATACTTATGTTTTCTCGTAAGATCAGGCTTACATTTCTTTTCAATTCCTTGCCATATTTTTAACAGCTAAACTACAGCA<br>3:**TACGAATT**CATACGAATTAGCCACTAAATCAAAAAGTTACGAATTGCCGTGAGATCGTGTTGAAAATTTTATGGTAAATTAAGGTAATAAAAACATCATAAAAAGTGTCATGCAGGGAATTCTGGGAAATTTAAATTATAGT<br>TATTCAATTTATACATTAGATTACCTTTGTAATCACAGATGTGTCTTTCACAGTGTAGAGCAGTGCAGTTATTCTGCAAAATAGTACTCTGTTAGCAAGATCTAAGTATTTTTCAGGTTCAGATTAAGGGATGTGTCATGATTT<br>AATTATATTTAGCCAAAAACTGTTCAGTAGTCCTTTTAAGCGATTTAAGTTCTCATTTGAGATTAAAAAGTATTGTTAATGCAAAATGCAGACTTTCAATATTTAATTACCAATTAAATTTGATTATTATTATTTTGTGTAGTC<br>CAGGAATATACTAGTACTAAAAAAATACTGTAGGGGGTTTAAATATTTTACGCACCTTAAAAAGGCCAGTAGTTCACCCAAACGTGTAAATTCTGTTATCATTTATTCATCCTCCACTTATTCCAAACCTGTTCTTTCATTTCT<br>AGGAAGGTATACTGAAAAATGACAGGGAGAAAAAATAATCTTTCTACTATGGATGTCAGTAGCTGTTTTTTCCTAACATTCTTTTATAAATCTTTTTTA
ET33-mi55	3:**GTAACAAA**ATCCATTACAAGTTACTTTTTAAAGCACGTGTTTATTAATCAGTGGAATATACTCCTAGTCCTGCTAGTCAGATCTGCCTTTTGGCAACAAGTCTAAATGGCCACAGTACAATAATAATAAATTGCAATTTTAC<br>AAGTAATATTTTAAATATATACTATCTGATAAATTTATACTTATATTTACTGAACATATACTAATTGTTCCTTGAAAAGAGTTGGGGTTTAACCCCGGTGTCCCCACTGGCCTCTGTTTATCATGGCCTCCAAACCCATTAGTA<br>TCTAACTTTAGTCTGAGACATGCAATCTCAGGCACAATGCACTGAATGAAGCTAATGCCATAACAGTGAAGGGTAAGGTTAAGGATTAGGTGTGCACATTAAAAGCATTGCATGAAGCTCAGCTCGCATATCTGAAATCGCAAA<br>GCCTCTCTTCTAACCATCCCCATGTCATAATTGGCTCCATCACTCCTCTCCACCTGCTGTGTGGTGTGCGGCTTGATGCAAAATGGCTGCCACCACATCATA
ET33-mi56A	5:**TGCGCTGG**TTGGTGCTGTGTGTTCAATTGAATTGCGCTGATG<br>3:**CCAGCGCA**TGCAATACCGCCGCGGACGCACTGAACGGTGGCTTATAAACTCCACAACTACATGTGCTGCTTGGCGAACTGAGAGAAAACTACAAGCGATACCTTTCTCCTCTTTTCCCTCTCACTGTCATCCGATCTTTTCA<br>TGTTCCTGAAGTACTGTTTACGCTGCCTCGGTTTCATAGCGCGCAGCGCTGGGTTATGTAGTCTTGAGAAGTGTGCCGTTTTCTTGTGAAATTATAGTGAGTAGATTTAAATATGAAATAAGCAATGTTTATAATGGTGTATGA<br>CACGCGCATGGCAATAAACGGCGTGTTGTTGTTTTATGTCAGCGTCTTGTTCTTTAGGTCCTTCCCAGTATTACGTTAAGAACTACGTTTCCCAGAATCCTTGCGCCAGGAAAGCCACGCTTGAAATTTGAGTCAAACCGGAAG<br>TGGATATAGGTGCTTCTCACTTGTGGTAAAGTGGCGAGTTTGAAATAGTGTGCTCTTGCATTCTTGAAGTCATATTTTTAGCGAATTGAA

ET33-mi56B	5:**TAGGAGGT**ACATTTTCAGAATGAGCCTGGGTTGACAGTCTTAATGCTTAATTGTCAGACAACATAGATCTTTCACATTAATTTGATCAGTCTTCCCGGTACAATTATACAACTGACTGTCACTGCTAAAGAACAAAGATTAC<br>AATATAACTCGTTTAAACTTTAGCCAACCCCCTGGGTCTTCCTCGATTAAGTACTGTAGTCTTCGAAAGGGAATTACAGCTTTCATCCCCCGTGCCTTCCTGAGACTGTACAGTGGGGACTTCTTCCATAAGCGCTCTCCGATA<br>AATCCCTGATATGCTTTGCCTAAAGTTTTGCCTAATATTGAGTCCCATGCAAAAATAAAGCAGCGGGTTGACGCAACTGTTGAAATACGCTGTGCCCTTAGCAATAGGCAGGCCCACTTTGACTATTTGGTTTTTCTTGTCCAC<br>CATCTTGGCGAGCAGCAGGCATTGATATGGGGCCCACCACAGGAAAAAGGCGACC<br>3:**ACCTCCTA**CATACATTTCTGGAGACCATGAAATACGCCCCAGGAGCTACGTTTTTTAGCAGTTTTTGTTTTCGCAACGCCTTTTGAGATCTCAAGTTTCTTTCGCCATTCGCACCTGCTGTTCTTGCGTAAACCCACCAGAG<br>GCCACTGTTGACTGACTGACTGAAAGATCGACTGACCCACCATCCTCCTTCCCTAAACACAACCAATTTAATTGATTGATCTGTCCACCCACTTTCCCAACCAAACCAACCATTTTCAAGCAATCCAGAAAAAGAAAAGCATTC<br>GTCTGATTTTTATCATGTTTTTAGATTGTACCACATTCTCACCCTGTTATTTACTGTTTATTTATTATTTTTTTTGGATTCTGTCTTTGTCCTACCTGCTTTCTGGAACCGTTCTTTTAGACTTGAACCCTGTCGTCGTGGTCA<br>AGTCCTCTCTGCGTCTCAAATCTGCCAACGTACATGGCGAGCTAACAGAACAAA
ET33-mi57	5:**ATTGTGGC**CTATCTCTCCTTTGACAGTGAGCAGTGGAGTGAGTGGTGAGTGTATGGATTATTTATTTCTTTATTTATTTTTACAGCCAGAGCACCAGGCCAGAAGCGAGTGGCTGGCACTGAACCTGACCATGAGAAAATGA<br>GGCGTGAATGTCAATCACTTATCTCAGTGTTCCAACACCATCCTCTTCACCCACAG
ET33-mi58A	5:**CCCTCTGT**TATAAATTGCCCAGAGGGTGGGGTAAATATTAAAATTTTTACTCCAGGCACTTTTGGCAATACTGCACAGAA
ET33-mi58B	5:**CCGGTGGG**TTCAGCGGAATTAGCGCGAGTTCTTCTCAAACACATCACGGATCAAAAGGCGCGCGCCGCGTGGATCATGGCTGGCCATTACCGTCGCTTAATAGTAGATTGTGTCTTAATTGGAGGAACGAAGGAGACTGAGA<br>ACCCCCATGGAGGGGCAAACATATCCACTGTCTGTGGTGCCCGGGGAAAGCAAAAAAAAACGAGGCTCTTCTCGGAAGCCTTGACGTCAATGCGTGCACTCATGAGCCTGTGTAAAGAAAACCAGCAGCAACGAATTGTTTTGC<br>CCTATAGGCGTGTTGGTTAGGTTACCACAGGAACCACATTCAACCAGCACTGGAATGCACAATATTAATTTGCCAATGTAAAAAAAATAAAAAATAAACATGATGCTAATAATTGTACTTTGTTTTAACTAGATCTGTTTTAAA<br>CGCCCCCTACGCAAATTAACCATGGTTTTACAACAAACAAAAGTTATATTGTTGTTGTTAAATCATGGTAACCATGGTTTTTTACACTACCATATTTTAACCATGGTATTTGTAGTAAACTA<br>3:**CCCACCGG**GACGGATAGACGGCCTCACCGGCTCATGGATCGCGAACCCGGTGACTTGGGACGCATCCGAAGCTGCATTATTTACGTAGTTACTATTTTTCGCACACCAGCACGCCGCGCATGCTGTTAAGTGCAAATGAAGC<br>TCAGACTAACCTTTACCGACAGTATTTTTCACAGCCTGTGTTTTTCAACGCTAATTGCAATCATGTGTGCGTCGAGCGCGCGCACTGGCCTTTGGCGGTCTGAGACTACAAACATATTTAGACACACCGCTGATGGCGCTTATT<br>TCTGCAACGTGTGACCACAAACTACGCCGAGGCCTTCTAAATTTATATACGTGCAAATTAAGAAGAATATTATTCTGGTACACAATAAATTATTGCCAGTATGCAAATAGTAGTCGAGATACAATAAGGAACCATGAAAATCTG<br>TTGGACAAAGGGATAGTTCACCCAAAACTGAAAATTCAGCCATAGTTTACTTGTATTTGTCACAAACCTGTTTTTGTTTCTTTCTTTGTTGATCACAAAACAAGTTATTTTGAAGACTGTTGGAAACCCTTAACTTTTGAGTTT<br>CATAGTACTAGTTTTCGATATGGGTGTCAATGGTTAATGGTTTGTTACATTTTTCAAAATAACTTTTGTGTTCAAAGACATTCATTCATTCATT
ET33-mi59A	5:**CATCTATG**TAAGCACATGGGCTTGGTGGGAATATTTCAAATTTAACTTTTAAATATAATACTTATTTTTATAATATTGAACTATTATCAGTGCATTTCAGGATTCTGCTTAGTCTCACAGACATGAATTCTGCAGGATACTT<br>GTGAAAGGGCAAACGAAATAGCTCAGTTGGGAGTGTGTTAGACTGAAAATCTAAAGGTTCCTGGTTCGATTCCGGGTTTCGGCAAAACAGTGCTGTTGTTCTATTTTGTGCACAAGGGTCAGGTGACTGAAAGTCACCCAAAAA<br>GCCTAGCTGCTTCACCCTTAAGTCCACCTGCTTCCACTTGACCAAAACGCTATCTTCCTCCCAAATAGTCACGTTGCTTCTGGCGATGATTCCAATGGCCTGATCGACAGCACTCTATAATGCTATGTATTTAGCGCTTTGATG<br>TCCCACTGCGGACACGCTGCTGAATAAAAGAAAACTGTGACTTGATAGGGCTTCTGAAGGCTTGCAATGATATGTACTACATGGAAACCGGCAAAGCTGACGTAAGTAGTTTCGCTTTAAACCCTCAAGGTGCCAC<br>3:**CATAGATG**TTATTGATAATGCCTGTCAAATTTCATTTTAAGTTGAAATGTCAAGGGGTGTAACTGGTTACACCATTTGACTCGTATAGCAGGAGTCTTTCTGTTGGGGGTCAATTTAGTCAAATATCATTAGTTCACGATAA<br>TGATCAAAAATGCGACATAAATTAGACTACTATTTGGAACTATTTGTGTTCATAGTGTAGATTATTTATTTATTTTTGTTTGAGAAATAGGTGCTTAATCCACAACCTTACTATTAAAGCTGTCAACCTAGGATAAAGTTATTT<br>TACTGCACAACATGAATGTATTTTTTTATGTTAATCCATGTTTACACAATATTCACACAATAAAATGTACACACTCTCTCATTCACACACTAGGGTTGGGCATCTAAGCTATAATGCCGATCCGATACGCATCTCGATACAAGA<br>ATACGATCCGAAATATTAGCGATACATTTGTGACATTGCGATGCAACACGATACGATTCACACCCATATCACGATACAATGCGATAATTCAGCACTAGCTAATTAGATCAAGTTTATGAGATTATGTGAAAGAGGATGCTGTGC<br>CATTGAATGAGTTTAATTATTTATTAACCAAGCAAAACCAAGACTTTTTTTACAAACTGGCTTTTCTCTCAGAGCCAGTGTCAATTTACAGTAGAGAGCCATTTTAGAACATAAAGCACATATTATTTAAGTATTTTCAAGATA<br>AAAAGAAGTGTTGTG
ET33-mi59B	3:**CCCTTAGT**ACACACACTCTAAAATAAAGTGCAAAATGTTTACATTTTTCAATAAAAAAAATTACTTTTCTTG
ET33-mi59C	5:**AGCACTGG**GCAATACCCATGAACACTAATTCACACACACACACACACACACACACACACACACACACACACA<br>3:**CCAGTGCT**GGGTTGCAGCTGCAGCTGTGTAAAACATATGCTGGAATAGTTGGCGGTTCATTCCGCTCTGCAGAGCACCCTGATGAATAAAGGGACCAAGCCAAAGGAAACTAAATGAATGAATGAATATATATAGTTGCAAT<br>CAGAATTATTAGGCCCACTGATTTATTAGCCCCCTAGTGAGAGATTTTTTTCAACACATTTCTAATCATAATAGTTTCAATAACTCATTTCTAATAACTGATTTATTTTATCTTTGTCATGATGACAGTAAATAATATTAGACT<br>AGATATTCTTCAAGACACTTCTATACAGCTTAAAGTGACATTTAAAGGGTTAACTCGGTTAATTAGGTTAACTAGGCAGGTTAGGGTAATTAGGCAAGTTATTGTATACGATGGTTTGTTTTGTAGATTAGTGAAAAAAAATAT<br>CACTTAAAGGGGCGAATAATATTGACCTTAAAATGGTGCTTAAAAAATTAAAAACTGCTTTTATTCTAGCCAAAATAAAACAAATAAGACTTTCTCCAGAAGAAAAAA
ET33-mi60	3:**TATGTATC**TCATGAAATATGCTTAAAATGTCTCTTAGTTCATGTAGGATAACCTCAAGAGACTCTAAAATGTACAACACATAATAGTCACACATGAGATTATCAGACCTTTGAGTTTAAGTTAAATAAACATACAAATGTAT<br>TTAAACTTTTATATAATAAAATCATGTTGGACCAACTCAATTGCATTTAATTGTGTAAATGTTTTTGTTAGTTGGTGTACAAATGTATTGGATGGAAATCCTGCCCTCAATTAAACTGAGCTCCTCCAATGAGTTATTTTTTAT<br>TTATTTTTTAGTTTTGTGACTGTGTTCGCAGCACTCTTTCCTTAACAAAGATACTCTGGGTTTAGTTATAAATCAGTGAAGATTTTTTTTATACTACAGATTTGTTTATTGTAAATAAAAAACAAACAAAAAAATTACATTATTC<br>AAAAAGATTATGCTTTTTTTAACAACAACTAACGTCAATCTGTGTAGCAACAGACAGTTTTACAACATAATAACCCTAAAGCCACAAAAAATAAAGACAATTAATTTAGCTGTAAAAAATATCGGTTAAATAACAGTTTCAGTA<br>TTTTGTGATTCACAAAGGTTTTCTGTTTATTTGCGGTTGTAAATAACATTATGGGACTTTGATCTCTCCTCAGTAAAATTTTGATGTTAAAAATTCCACTTTACAGTTTAACAAAGTGACTTTTATTGACATTTTAGGAGTTTG<br>AAATAATATAATGTAT
ET33-mi61A	3:**AGAATATG**TTACCTTAAAAAAACAATTAGTAAAACCAATTTTTTTTTCAGTGAGTCAAAAGCTGAAGCACACTAGCGCTAAATAAGAGCTCAGGTATCACTCTGCTAATTTTAGCTCAACTTTAAAAAAATCTTTAACAAGG<br>GTCATAAATCTGAGCAGTAACTCAAGCTTTTTTTTCCACCAAGGATGAAAGACCCGGACTGAAACGGACGAAAACAAACATGGCGGCTGAAATGTGTGTTTGCGTTACGCTCCTCCAAGGCAACCAAAACAACAAATACGCACA<br>CTGGCTGCTCTCAGCCCGACGTCTCACA
ET33-mi61B	5:**AATGCAAG**CCATGTTGATCAAAAGCGAAGTGGCATTTGCTTTATTTTTGTAGTGTCCTTTCAGTTTTATGCCTTTCCTACCGCAAAAATAAAATCATTGTTGTAGTTATGAACACCTGTATCCTTTTTTGAAATTGTGCATA<br>CCTCAATGCTGATTACAAATGACATGGGTAGGAAATAGGACTATTATGTTGTTCCTTGCCTTGCAACTCTTGATTTCTATGAAAACACATGATATCCAGTGGGACTCACAGTAAAGACTCACTTCTCTTTGAGAAACCTCACTT<br>CAAATACAGTAGTCAAAGGCTACACATTACCTGATGGAAAGTGCTCGTTGATTGGCCAGTTGTCCACCTGTAAAGTGGCATTTCCCCCATTCCTTGTGAAGCGCACCACATGATACTTTCCATCATTGACTTCTGTAGTCATC<br>TCTTTGACGCTGATGTCCACTGTGCCGATGTTAAATGTAACACACACCTTTCCTTCTTCCTGGAAAATAAAAAAAAAGAAAAAGCCTTG<br>3:**CTTGCATT**GCTTTCGTAATTGACCTTTGAATTGTCCATATGCTGAAATAATCAAGCCGGCGCACAGTGGCAGGCCAAGCCGCTGCCTTGGTCCACCACGATTAGTGCCATGACATCTGGCCAGCTGCTTTCGAGAGGCTTTG<br>AAAGACAAGAGGAACAGCAGAGCACACATTTAAGACAGTAACGCGACAGCTACAGCAGCTGTGGGAGAATGAACCTCAGACCTTTGTGACATTCAAATAACATTCAAAACCCTAGTAGCATTGAAGTTGTAGTACGGTGCTTTT<br>AGGTTTTCCGCAACATTTTGAAAAATTGTTTTCAGTGAACAAACCATGCTACAACTGACGAGACCATCATGATTATCCTACTTAGGGCTCTATCATACACCCGGCGCAAGGCGCGGCGCAATACTTGTTTGGTAGTTTCAGCT<br>TGGCGCAAGAGTCGTTTTGAGGCGTTGCGCTACACTGTTTAAATAGCAAATGCATTAGCGCTCATATGTGCGTCCATAGGCGTTCTGGTCTAAAAAGGAAGGCGTTCTGAGGCGCACTGCTGGCGCGTTGCTATTTTGAGAAAC

	TATAATAGATTTTTCATTAGACCAG
ET33-mi62B	5:**ACAATGAC**TGTGGGCCTCTCTGCCAGTCTGACACAATGAGCACAAGCCCGGCCGGCTGTTAGTTTTACTAGGTCACCAGCAAACACATTTATCCTTGAGGTGAGCCTGGTGCTTCAGGGAGGGCGAACACAATGGCGAACTTGTCAAAACTAAATTGAGCTGAGCGCGGGAAAAAAAGCGCGCGGTATTTGTCCAGAGAGCGGGAATCTACGGGGACAAGCGACTGAGCGACTCACAAAATGTTTACAAAGGCTGTCGGTGCATTTTCCCCACAAACCTGCTACCACAGCAAAAATGACCCTGTCTGGAGAGGGGTTTGTTAATATTTGAGAGTGAAAAGGACCGGTTGCTAAACAAACACAATCAGGATGATGAGACCATGGAGACAGTGCTGTGGAGATGTTGAGGTATTTTAAGTGATGTGCGGTCAGGTATGCGTGACCCTAAAAACTGAAGCTGTAAATTATTTACTTTGCTCGTTTACAGTTCAGCATGCTCTTTGTAAGGTTTTGTTCATAGTAAATAAAAACTTCAAATGTGTGATAAAACTGTAAAA<br>3:**GTCATTGT**TCACCACACACACATTAGACAAGTGTTATTTTTGGGTTAAGTTACACCAAGTCTTTGTCTTCAGCACAGAACTACCAAGA
ET33-mi63A	5:**CTGGAGAC**ACAAAAGACTCATCATAAATCAAGAAAAAGGGGTAAAATAGGTGCCATTTAAAGGTATAGTTCACCCAAAAATTTTAATTTTGCCATAATTTATTCAGTCTTTACTTGTTTTAAACCTCTATGAGTTTCTTCTGTTAAATACAAAAGAATATATTTACCTCTAACCATTGACTTCCATAGTTCTGTTTTTACAGAAAAAACTCTTTGGAACCACTTCAAAAAGAGTGTGTAAATGTGTAAATATTAAATATTTTGGAAAAACTATCCCTTTCATTGAACATTGTTACCAGCTAATAGGTATCATGCTATGACTAGACAGACAAGGTCAGTTATTGTCTGTTGGTGCGAAAACAAACAGTAAAATTGGGTCAAATTAATGTAATCCAATGTTTTACTGGTAGCTAAGCATATAAAACCCTCAACAGTTTGGGAGTGTGAAGTACAACCATGTAAATGACAGACATTTCCAATCTAGCTGTCTGCAATAAGACTTTTCTTTCTGTGGTTTAAGAGCATTAACGTTGTTTTATGGAACAAGAACGGCATGAGCCACAATGTCTTTGTTGGCTAC<br>3:**GTCTCCAG**AATGTATCTGTAACGTTTCAGCTCAAAATACCCATCAGATTATTTATTATATAGCTTTCAGAACATTGGATTTTCTGCTCTGAACAACCTTGTAGCTGTTTTTGTTGCCTGTGGCTTTAATGCTAGTTCTCCCCATCCACCCCTTCCCACATGCCTGTCAGATTGTGCCTCAATCTTCACCTCGGCTGCGTCAGATAAATAGCACAGTGACTGACATGAAGGAAGCAGATCTCATGTAAAGTTTGTGAAAAAAACTACAGTATGAACTTTCCCAATGATTATTTTATGTATTTGTTGTGGAGTTAATTCAAGCCTTTCTGCAAAGTCGTTACAAAGTTTACGCACACACAAACAACACACTGTA
ET33-mi63B	5:**AACCTAAA**ACAGTAACTGCAAGACTTTACAACAGAAATTATAAAGGCGTCCTGATTTTAGGGATGAGGCGCCACCTGCTGGCGGAAAAGTAACATTTTTAAAATGCACATATTATATGGTACTCTCTAGATTTCAGTATCAACGTGAGTACATTAATAAAAGACGACAGACTTTTCTTTTAGAGGTTAATTAACAGTTTTTCACCTTTAAACGTGAACAATGCACTGACGTCATTACGTACGAAACGTAAAGCGCGCGCTTGGTATATATATTCATGAGCGTAAACGTCATCGATCTCTTTGCCTGTGTCCGCCGTGGTAGGCGGCATGATAGTGAATTGACTGTTAAACGAATCCAAAAACATCAGGCAAAATGGTGAGTCGATTTAATAAGTTATTTATTTTATACGTTAGTAACCTTATGCAGTTTGACGTTTTGTTGCGATTTAACCCTTTAGAATGGTTAAAATAGACTAAAATGAAAGCGCAGTACATGCTGTTTCGGAAGTGACTGGTTTGAATTAACGTTTGTCTTTAGAAGTTTTTTTTTC
ET33-mi63C	5:**GTCTATTG**GGGTAAAGCTAAACTTTACTTGACACTGGCCCTCCAG<br>3:**CAATAGAC**ACACCTAAATAGGCTAACGAGGTATACTAGAAACTTCCTGGCAGATGTGTTGAAGTTAGAGTTAAACTCTGCAGGACACCAGCCCTCCAG
ET33-mi64	5:**TTTATGAT**TGACACGATTCGAGGTTTTGGTTTTAACCTAGAACAGCTCATATCTACTCAAAGCAGGCATGTTAAATTCTCAAAGGCCAACATTATTAGTTGCTAAAACTTAAACAAAATATTATTCCTTATTTGTGTGTGTGTGTGTGTGTG
ET33-mi65A	5:**TTCACTAC**AGTATAATCAAATTTAAACTAGATTGCATTCCAGCAGAAGTATTTTCATCTTAAAATGAACAGTTGACATGATTATAGTTGGATTTCTATTTCTACATATCAAACGCATGCTTTGCACAGGGAGTGTGAGCAGTAAGTGGTTATGAGACATGTAAATCGAGTGCTAGAATCAGTCCACTGGTGTTATTAATAACTGCTCATGAAATCAATCAAGCCTGTTCTATGCTCCTCCACAA
ET33-mi65B	5:**TGAATGAA**TGAATAAATGAATTTATGATTGAATGAATGATCAAAGTCATTTTGTCATTTGGGGACAAATGTAAGCGTTACACAAGAAAATTAGAATAATAAGGTATTTGTTAGTTTTTAAATTAAAAAAAACCCGGACATGCCTCTGATTGGAATATGATAAATGAATGTTACGGCTGTGTTGTTAATTCTCTGATTGCATCTGTCTTTTCTCACCAAACCCGTGTTCACCAGCTCCCCCAAGTTCAACCCTCTGAAATGGAGCTGTGAAAATGTCCATCTGTGTGTGTTTTCCCTTGGCAAACGGAAAGCATTTCTTAGTGAAGGTCTTGCCCTGCTTCTCTGGAGTAATTGGTTATTTAGTCAAGATGAATTATAAGTGTCTCTGTTTTCCAAAGAATGTCTGTGTTATCTTATGTTGTTGTGGGATGAGCCAGCAGTTTCGCAAAAACAGGGATGCATGTTGTTGCTAAATGTAAAAGAATTTTGACAGACTTCTTTTGTAAATACTGTAGAATGACCGGATAGGATGCTCCATTAAGCCAGTCTGTCATAAAAATATAATTGAAATATCCCCTTTTTATGTTGATAACAGAGCTAATAGAGGTTTAGGTTTGTAAAATGTTCAAAAACTGTACAACCAAGACACTGTATCATCCATGAATTCAATCCGGACAATGTGGGTGTGC
ET33-mi66	3:**CACGTAAT**TTGTTTCTTCAAGCTTTGTTTCTTAGCTTATCTAAGTTCTAGTTGCAAGGTTGTACTCCATTGATTTATTTAACTTCAACTGTTTATAACAACTTTTTTATTAAGTTAAAGGTTTGACACTGATTTGAACTTGAAGTGGCGATGAGATCTTAAAAACTTCTGCGAAGGTCTTTAAAAAGTCTTAAAAGGTATTGAAATTACTTGGATTCCAGA
ET33-mi67A	5:**GTCTTTCA**TCCAGCGAATTCATCCTCGGTAAGTGTTTTAAAGTCAAATTCTGTCCGTTGAAGGATTGTTTTGTATGTATTTCTGTGTATTGAGTGTGTAATTTAAAAGAATTAACCAAGTGACATTAGTTGTAACGTTAGGTTCTTATGTTATGTCACGCTGTCCGTTAACGAATGGTATTTTAAACAAGAAATAACAGACGCCAGGCGGTCATTGTAGTGTTTGCGAGGTATTAGATACAGTGAGTTTATCTTGAGTGAGTTTTTGACCATACGCTAAGTTACCTGAAAAAACATAACTTTAACTTATTGTAACTGCATTAATGCATTCTCTATTTCAGCAC
ET33-mi67B	5:**CTATCTAT**CTATCTATCTATCTAGATAGAGTCTAGATGACTCTTAAAATCTGTTAATCATGGCAGTCTTTAAGACAGCATCGAACCTTCTTTACACTACACGTTTAATCTGGTTTCATTTACACCATTATTGCTTAACACAAATTCATAGCCTTCTTAAATGATTCATGCCCTCAGCCAGTTAAATCCTTTGAATTAGGTCACTAGCAAAGAACTGGACGTGATTTATTCGCGTTAATGTGCAGAAC<br>3:**ATAGATAG**ATAGATAGATAGATAGATAGATAGATAGATAGATAGATAGACTAAAAAATGCTGGGATCAACTTGTATTGGGACAGCATAAAGGAATCAAGTAGACTTCAAGGAATCAAGTTAACTTATTATTATTTTACAAATTTAATAGGATTGCAGATAAAACAATCAAGTTGCCCCTTAAAAAACTTAAGAGTTGTGTTGTTTCAGTTCATCTTAAATAAATAGTTTGAATAAATAGCAAACAATTGTATAGACAGATAGGTAGATAGACAGAGATAGAAATTTCCCTGTTGAAAAAGCTTTGTATAGAATATAATGGGAATTTTAGTGGTTTTAATGTATTCAATTCATCTTTATTTCTAATGCAGATTGTGTCAAAGCAGCTTAACATAGAAGTTCTAGTAAATTGAAACTGTGTAGTAGTCCCTGCAGATCTCTACTGGTAATTTGTTGCTTTTGTTGGTGTCTTTTTAACCAATGGCATACTAATGGCAAATGTGCCAAAATACAGAGTAACCAGTAAAATTCATTGGTTTTAATGTTTAAAAGGTAATGATTTATAATATCTTCATGGCATTTGTAGTGGAAACAATTTGAAATGGATAGACAGACAGATAGAAATGTCCCTGCTGTAAAAGCATTGTAAGGAATATAATGGGAATTTTAGCAGTTTTAATGTAAACCATTTTAGTGGATCTTTACTGGTGATTTGTTGTCTACTATTGGTGACACTGTTTAAACCAAATCCTTATGGCATATGTCCCAAATCACAGTA
ET33-mi68	5:**CCACTGAG**CCACCATGCCACCCATTAAGGAGGAGAAGTAAGGGTGGGAGGGGGATTCTTCAAAATGAAAATGCCTGTTATATGGAACTTAGGGTATTTATAGTTACTTAGGAATTGTCTGATTGGTAAATCGTAAATTGAATAATGCGGGACCGACTGCAAGCAATCATAAGCACGTGATCCTGTCAAAATTAGTTTATAAATAAACTTAACTTATTTATCAAAGCCATCATCTTCATTATACAGACAA<br>3:**CTCAGTGG**TTAGCACTGTTGCCTCACAGCAAGAACGTCACTGGTTCTAGTTTTAACCAAGCCAGCCGACGTTTCTGTGCGGAGTTTACACGTTCTCCCCGTGCTCATGTGGGTTTCCCCCAGGTTCTCCAGTTTCCTCTCACTGTGCAAAAACATGACACTTAAGTTAATTGACTAATCCAAATCAGCACCATAGACATGCTCCAAGTAAGTAATTATCTCTTAAGAGCAATCACTATCTGATCATGAGCTACTACAGCAGGGAAGTTCTCCAGATCTACCTGAGCTCAAACTCACCTCTCGCCTTGCAAACGGGAGGGAGTCCTGGGCTCGAGGATCTTATAAGCTCAGGGCTCTCTCTCGGGACAGCATGCCAAACAAGCTTTATAATCAATCATCAGCTAAGTGCAACTCTTGAAATGAAAAAATAAATGGGCAG
ET33-mi69-1*	5:**CACGGCGA**TGTATCTGAATGTAACACGCTCAACTAATAAAAGACAAAGTCTGCCAGTCTCCAATTCGTATACACTGTTTTGACAAAAATGCTTTCTGCACACACAACTTGCCTGTATCTGTAGCCCTTGACAGCATTGCGGGGGGAAAACCATTCAACAAACCCTGTAAATCATGGAAACAAACGCGTGACATTTCACAACCACCTAAATACTGCATGCCGTGTGCTGTCTCACAGCATGGCAGGTCTGTCCGTATGGGTCTATCATCGAAAAGCACGCGCAGTCACGAATATTTAAGAAGCGGGCTCTACTCATAGGATTGGAAAACTCGGTCTGTGAATAATAATGAGAAACTAACGCGTCCTCTCT

	3:**TCGCCGTG**CAGTTGTGTTCACACGTTTAAAATCTTCTAGATTACCGGCAGGGCTAAGGGTTAGAACAGAAAGGGAAAGAGACCTGCTGTACTGCTGTCACTGTTTCTGCATTCAAAACCAGGGTTAAGGAGGAGAGAAGTTC<br>TCCTCATTTATATTGCAAACATGATTATCTTTATAATGATATAACAAATTGTGGTTATGTTTATATATAATATAAAATTACAAATAGCCTATCATATCTGTAACATGGCAATAAATTGCTGTTTATTTTTTTGCATTTTTATTT<br>TGTTAACTATAAAAATCATGCATCTTCTCACGTTTTGTGTGAGATTTTTTTTCGTTTTGAACTGGTGAGCTAACTTACAGTTGTTTGCCCCCATCTTCTTTccctggttttctagccatgcccactgctccatcctgctcacag<br>cgctctatgaccattaggcttgggcggtattatggtaatatggtagtcaaggattcaatattatgaaAATATAATTTATTTAATAATTAAAATGCTTATGTGTTGTTGTCATTACGGGACAGCGTGGAGGA
ET33-mi69-2	5:**ATTCCGTG**TACTTAAAAGGCCACCCTATGACATGATAATTGCAGATTTGCTTAATTGTTTTTTGTTTTTTTATTATGCAGC
ET33-mi69-3	3:**CTTCAAAA**CAGCATTAGCATTATTTAATTGACTGTGAACATTGTTGTACTGCTAACATGATTTTCCCCATTTAAAATTAGCAAAAACTATTTTTCTGAAATGATATCATTAAACATAAAGTTCGAATGTGCCAATATAAAAC<br>CAGCTTTACCTGAATCACGTCCGATTACTGTAACGTTACCTTGATTTAGCAACTAATGCAG
ET33-mi70-1	3:**ACTGTAGT**AGAGAGAAATGACATGAGCAGAAAGTCTCAGCGTAACACCCAACATTGTGACTCACACTCATTACACATCACAAGAGGACTGATGGAGAAAAGAGATTCACCTGTTTAGGGCTGCTGTTGCAACTACGAATGGC<br>TAAATAGGCTCATACATAATGCGTCTGCGTGTATTTATCTCCACAATCTTTTGTAAAGAATTTTGGAGGAATGTTCACAAAAGCCTCATAAGAACAGAACAGAGGAAACTTTGCCTTCTCAAACTTGTTCACCCAGTCAGATAT<br>TTTTTTAAGTGCACAGCTCTTCATTTTGCCAAAAGCATATTGGATTTAAAATAAAAAGATTC
ET33-mi70-2	5:**AACGTCAT**GGTCATTCCAGTAAGTCCATTTAATTGGTACTCTCTTGTTTGGGCTTCAGGAAGAAACTAATTGGAGTATTAATGTACAATTCGATGTGTAGGACATGTACCTGAATGCCGGAGGTGTCACAGTCTCTTACTTT<br>GAGTGGCTGAAGAACCTGAACCACGTCAGCTATGGTCGCCTGACCTTCAAGTATGAGAGGGACTCCAACTACCACCTGCTGAGTATGTTTTACACTTCCTCCAACTACCTTGTGTCAAAACCCCCCCTTGCACCTTTTGCCAAT<br>ATGATTACAAGTGGATGATGCTAAATACAAGACCCTTTCTACGTGTGGTATTAAAATGCGTTTTCATCATTGTCGATAACCCAGTGATTAGTGCCCCGATATATAGAGCAACAGCACTTCTGGTGTCCTCAGTTTGAATCCTGT<br>CTCCTGGACCTTTCCCGATCCAGTCCCTAACCCTCTCTCCCATTGAGTTACCTGTCATTTTACGGTCCTATAAAATATAGGTTAAAATTCTTCTAAAAAGAAAAATCTTCCAAAAAAAAACTTTTTGTCAATCCAGTAGCATGG<br>ATATATTATATATTATATAAAAATAAACTGGTCCGTAATCTATGGCCAA<br>3:**ATGACGTT**CCTCTCAATGAAGATTTTGTCAGCATCGGGTGTTGTGGGACCATTAGCACCTTCAGCAATAATCTACATCAATTTAAAAAGTAAGAAACTGTAAAAATCGATGAGTGGAATTTAAGCATAACAAGCTCAGTTGG<br>ATGTCGGCACTGCTGTCAGACCTTACCTTTGATATTGTGTGCATTTTTCCTGGTCAGCTGTTTCTCTCCAGCTGCAGGGATGAGAATATCACACTGAGCTTCCAGAATGTTTCCTTCATATGGCTGATAGTTGGGGAAGCCCAC<br>AATGGTGCCATGTTGCTAAAAAGAAAGCATAAATAAAGGTGTTTT
ET33-mi71A	5:**GTAAAAGC**GCAATAGAATTAAAGGTGAATTTAATTAAATATTGTCTGTAAGTACTTTACATTGTTGACTATCCCTTACATTATGAGCACAGTAAGTACATTGTATTTATAGTGTGATAGAAGTACAAAGTATTTAACCAGTT<br>AAACTCTGCGGAGTTGGTACCGGGTCCGTGACGTCATCGACTTTCACTTTAAGATTTAAAGGGCTAGCATTGCACCAGGCCCCCTTAAGGGGTTTTGTTTGAAAGTGTAGAATCTATATTTTATGCACATATGCATCACTTTGG<br>TTTTTATTCTACTGTATAAAAGTTATTTACACTTAAATACACAGTATTTTGGATACCCGAGCTAGGTATTTTACATTGGTTCATTTTCATATTTTTTCATATGACATTTACATTACATTTACATTTAGTCATTTAGCAAACGCT<br>TTTATCCAAAGCGACTTACAAATGAGGACAAGGAAGCAATTTACACAACTAAGAGCAACA<br>3:**CTTTTAC**AATGTAGATTGTGTCAAAGCAGCCTCACATATTATGGTAAACTGAAACAGTCTCAGTCCAGTTTAATTACAGACATTTTACATGCAGGTATTTATTCAAGCAGTACAATATATTGATATGTATTCACATAATTGT<br>AACAAATTATTAGTTTCAGTGTAAGCACATATTAGTTGAGGCTACTTGACATAAAATAGGACTAAACATTTGAACGGCTCAATGGCTTGAGTAAACACTCAAGAATGTATTTACACAACCCAAAGAAGTCGAAGAATTTCCGAC<br>ACAGCCATATTGCTGACTTAAGTAGTTATTTCGCTGAATGCGCTCAATCTAGCCAAATCCTCGGTGTACAAAATTGGGAACACGTTCCAAACCAACATTGCTTTTAATTCCTTTCTTTGTATGCGGCTCAAGTGCAGGAAAGAG<br>ACACAAACACAGAGGGAGGGAGAAGGAAGTGCGTACTGGTCCTGCTTATGTGCTCACTGAAGTCCCACATGGGATGGGATTATTTTCAATACGTCATTCAGAGAGAATGCATGAACTGTTTTCTTCGTGCCGAATGCCGTCCTC<br>GGGGTGTAAATCATGGGGGAGCGGGATTCGGTTCTTAACAACTACAATAATTTTGTTTCTTTGATGACCAAAAATAGATGTTGAAAATAATCTGACTACACACATTTTTGTGCTGTTTGC
ET33-mi71B	5:**TTGATGAG**AACAACATATTCCACTTTTTTTATGTCTTGCCCACGACTTCCTGTATCATGTTCACTTATAATATAAAGTGTTTTGTTTATTTTCTGTGTGTCCAAATC
ET33-mi72-1	5:**ATTATTTT**CTAGACAGGGCGTAATACTACTACTGAGCCTAAAATGAATGGATTCCTCCCTAAAACACACCCTACAGTCAAAAAACCTCTCTGGGGGCTGCTGATTTTCCGTCGCTTTCCATGTCAGTGCTTTTGAAAAGTAT<br>CTGCCAAGAGTGCATTGACACAAGCATTTAGAGGACGTGTGGACTAGTTCAGTTCCATTTTTTGCTTGAGCGATTACAGTACATGTCTGCGCGGCAGGTGAGAGAACTCTAAAGTGGCATCTTCCTTAACATCCATTCACCTGT<br>GAACGGCTCCTAATCTATTTACTACTTAAATGAAAAGACATCACTTCAAAGGCTCTCTGTAAATATA
ET33-mi72-2	3:**ACAATATG**TGGAAATGAGGAGGAAGATTCTGAGGTTAATATGAATCGCAATCTGGTTTATATTATACAATAATAAATATTGACCAAAGTTAGACCAAAGTGCATTGCTGAATGACAATGAATCAACTTAAAGTGTCCGGTAA<br>TAATAATTCACAGGTTTCCCACATGTTCATGAACACCAAATTTAAGGACTTTTTAAATCACTAATGATTTTAAATCCTGCACACTTCCTGACAAAAATGTTGTCCTCGATCC
ET33-mi73	5:**TTCATCAT**ACATAAGAGGAACTTTATATTATTGTGGTTTATTTCCTCACTCGCCACCACAGTCTAATATCTGTCATGTCTTGCATCATAATTTCTTATATTGATCAATATAGATTGAATTGTAATTCAGCAAGAAATCTGCT<br>GGTATTTAAAGCAAATGACCTAACGTTATTCATTAAATAAGCCAAGACAAATTTTGAAGCTACAGTGGTACGTGTCCTAAATGCACACAAATCATTTTAAACAGTTGTGTCCATCCCTTAAACAAAATAAAAATGTATGCTTTA<br>GGAAAGTAAACATATTACATTTTCCATATGCACCAGATGTCATATTTAGTTTAAAGACAAGAAACACCATGTTAAAAAATGAAAATAAAATCACGTGGAGTAGAACAGAAGTAATTTAAACAAGTTTTGAAAAGTTAAATGCGT<br>TTTAATGGGAATGTAAAATGATTTTAAGGTGCAGTAGGTGATCTGCCAAAATGCTAACTGGTTAGCATAATATATTTGAAAACACTGTCCCTCCCGTCGTCCAAAGCCACGCCTCCAGAAATTGCAAATGCGCACATTAAAGAT<br>GACTGTAGACAACCCACTAGATTATGTCATTCGCCAGTTAGAAAACTGTATTAAATCTATCTAGCACGCTTTCAGTAGTGTAGTTAGCAAGCCAAACTTGTCATCACTCTCTCATCAGGCTCACTCTCTCATGGGCTTCTTCCT<br>AAAGCGACTACTGTATGCTTAGTGGTTGGCCGGGTTTCAGGAATTACACCTATTACTAAGCCATGCTGCATGCTATTCA<br>3:**ATGATGAA**TAATGGCCACTAAAAAGTACGCAGATAAAGACCAAACAAATAAATTAGAGAGGGAAAATACGCAGCCATATAAAAGTGTCATGCAAGTTACGTGTGTATGTTTAAACTGCACAGCACACAAAGGGAACAGGGTC<br>GTACCTTGTCCAGAGGAAACTTCATTGCAGTCGCTGCCAATTCAAGACAAATAACAGCTTTGCTGGTTGCAGTCCCAGAACCCAAGCCGACACATTTCACTTGGGATAATCGCATGTACTCCTCTGCTTGACTGAAAACAAATG<br>GATGAGTTGTCAAGATGTTCTGCTACCAATATTGTCCTCTCAAAGCAAGCGACGAGCCACTAAATAGTAAGTTACACTTGAACACTCACCTTAACACTTTAATCGATGTTATCCCAATCTTCGAAGCAAGCTTACGAAACAACT<br>CTTTATCCATTTTATAAACCCTTTACTGCAACAACACAGCACAA
ET33-mi74A	5:**GTTATATA**ACTTATTCCTCATCCTATAAAACGAAGCAGGTCGAGTAAAGTGAGTCGGAGATCAAGGCTGACATGCGGCTGTGAGTCTCTCACCCTGACCCTCAAATCCATTCACGAAGCACATCCTCCTGTCACAAAACTCC<br>ACCATCCACAAACTGACATGATTTCCCATCATCTGCACTAACAGAGCTCGAACTGAGCCCTATAGCCAACAGATGGCGAAAACTTTATAATGTAATAAGAAATAATTACCATATAAATGTTACAAATAATAATTACATATAATT<br>GCTGTTGTTTTTATAAATGTTATTCATCTGTAAACTGTTTTAAAATATACAAATTCATATTTGAAGTTGACTAAACAATTAAAATTAAGCAAAAGTCTTTCGATTGTTTGAGATTGGTTTGAGGTAATTATTTTGTTAGCCCAA<br>GCTATAAACACCGTTTTGGTTATTTTTCCCGAAAAACTTGCGCGTTGCATTTTAAAAAAAGACAAATTTCTTTACAATTGTTTATGGTCCAGCTTTAATCCACACAATATTCTGAAGTAAACTCATGATAAAACGTTAGCAGGT<br>TTTCTTCTCACAATATTAAATAAATTAGCAGCGTGCCCGTGATGTCTTAAATGGTTACAGAAAAGGGAAACGAATGCAAGAAAACAAAACAAAGCAAAACGTTAAAATAATAGATTTGACCATAAAAAGAAAAGCACATTTTTT<br>TAAACAGGTTGTTTTTTTAAATCCGAGGGA<br>3:**GGAATAAC**GAGCCTGTTCGAGCCATGCGGGACACGACGGAATCCTTGAGACGCAAATGTAAATCAAACCGAAATTTGTAAACAGCCTCTGCGGTTACAATGCAATACGAGAGGGGACAAAGTATTATCGTTGCTCTTTAAAA<br>TTCGCAGGTTATGTAATCAATTATAATCTTATTATAGCCTAAGACTTTATCTGCCTTTATTTTTAGAAGAGTATTACTTTATCTACATATTTAGCTTTGAAACAATTATGTCATATTCAACGGTGTAAAGAGACTCATTGAGCT<br>CGTTTTCCCCAAATATGACTTATTAAAAACGAGATTCAAGTCAATATAATGTCAATTAAGACTTTGTAATTTCTACACAGGTATTACATAAATGCAAGCACACAAAAACACATCATTATTATATACAAATGGAATAAATTAACA<br>ATTGATATTTAACCCTTTAAAAATGTAGAAAATATTAACCTTATCATGAAAATGTATTATATAAGGATATTACCCTACTAATGCTAACCTTCATAATGGGCTATTAACAATATGAACGATGAT

ET33-mi74B	3:**GTACTTTA**TAACCTCATTCATATCCCCCTGGCTACGTTGCTTCACTTTCTTAACAATAGAATAAAAACATGAGTAACTGCAAAATAAATAAACTTCATTTTGATATTAGCAACTTAACAGACACCAAAAATGTTGAAGCGTC<br>GTGTGGCTACATATGTATATGAGCGTCATCTTCACTGTATGCATGTGTATAACAAAACGGAGCCTATAAAATTACTGCCTTCTTTCATTATTATTGAAAATTTTAAAAATACTCTCTCTTTTGCTGAATATCACTTTTAATAAT<br>CAATAATGGCCATTATGAAAGTATAATGTACAATAAGTTTATATACTGTGGAAAATAAATGCAAGCCAAATGTGCAGAATCCGTGTACATAGTTACATAAATTGATATATTAACTTATCTTTGCGCTCAGCCGAAACACGTTAC<br>CTGCAGGGGTGATAGCGTTCCGGCACCACGCCGGATTTCCGGCGTACTGTGGCTGCGGGGAAAAAAAATCAGGTTCACGGATATTGATCTGTCATTTCTCTAGATTCACAGAATTCACTCACTGCTGAAAGCGCCGGATTGGT
ET33-mi74C	5:**GTTAATCC**TCTCTCTATAACAGTTCAAACCACTGTGAATGCATAATTGCGCCAATCAATCTGTGGCAAAAAAAAAA<br>3:**GGATTAAC**TGTAATGAAAATGTGATATCAGACACTGCTGATACGCCTATAAGACTGTAGAAGGTCACTTTAGTAATAATCCATGCACTTTTTCATAGATTCAAAACATCTCAATTATCCTGATTTTATTTAATTCTCTCGAT<br>TAATTTCTGCAAATGAAAGAACATTGTGATTTCTGAGGTTGAAGACGTCCCAAAGACAGGTATGGTATGTATTAATTGTTAAAAGTTTTAATAATATTACATTCAGATATGTTGTTGAGGTGAAATCAAGACATCCTGGGATTT<br>TGAGGTATATGCTGAAGCATGCTAATAGGACTTTTAATTTGCCAGTAACCTGGGTTAAGCGCTTCCGGCAAACCATGCCAAAGGAAAAATAAGCACGTTTAAGGTCTGTTTTCTTTAAAAAAAATCAGCGATCTCCACTGGCTGG<br>GTGATATCCGATATAAAGTGGGTCTCAGATTGTACTAGAAGCACTGCATTAAAATAGATTTTTCCCGCCATGTCTTTATAATATGAGCATTTATTTTACCCCGGTGGATTCAATGGAGCTTCTGCGCTAGCCGCCGATTCTGAC<br>GTCCGAGTGCGAGTAAACGGCTTTATGTCTCGTTTTACGCTTGAACAACTAAACGAATGCCAAAGTTTATTTAACTGAATGTATTTTAATTACATCACAACATGGATGCTTTAACAGGAACCGTATAAAGTGGAAAAAACTCAC<br>AATAACCGGTCACCGGAAGTTTATAAGTATGGAAACAACACTAGCTCGACATATATCCAATTATATTCATCTGTCTCCATATTCTACTGCATCTCAGAATATTA
ET33-mi75A**	5:**GTGTAAA**CTTACAGGAACAGGTCAGAAGATGACCTCTCCAGTTATCAGCTAGGATCCCAAGGGGGCTCCGCGTTGAAATCAAACTGCTCACGGTAGACAGAAGTCCCTAAGCTGTTCTCGTAGTTCAGGTACACCTGCAAAA<br>ATAAATAATCAAACAGTGTATGTTAGGATGGATGGATGGATAGAGTAGTATAACAAACATAAAAGATTTAGATTATAATATTATTAAAAGACCTGGGCAATGATTAATTGTAATATGGATAAAAAAACTTCCACATCCCAAAGT<br>TTTACCACGATCATTTCAAGTGAGGGAGGCATCGATACAATTGTTCGTACGTATTTCAATAAGTCATAGTATTTTAAAATAAAAATTAACTATTAATTTTATCTGAACAGAGGCTTG<br>3:**TTTACAC**ATTACTGTATCCTCGGTGTTTTAACGCAGGGGCTTTCAAACTTTTTAATCTCAGGGACCCCTAAATATGATGATTTCTTACAGGGGATCCCTTTGCCTAAAGCTTAAAAGTTGCTTATATATTTCAAGTATAGAG<br>ATGCAAACTAAAAGTCTGTATAAAGACATTGTAGTTCATATTTCACAGGCATGCAAAATGGTATTATTTTAAAAACTATTTATCTTGGCAACTCACTATAGCCAAAAATGATTCTCAAAATTTTTATATTTCTTAAAAAAAACA<br>AAACAATGAAATATTTATATTAACCGAACAAGATAGCTTTTCTAAATAAGACACTGGTAGATGAAAAGCTAAACCTGGACATTTGTAGTTTCTGTAGTTTTTTCCTGTTTTGTATTTATGGAAAACTGCTAAATATTTACATTA<br>AACTTATGCGTTTGTTTGCTGGGTTGTCTAACAAAGGCACACAATCTACCTTTCGTGCTGCTTCCTCTCCAAATAACAATGTTGAATGATGCC
ET33-mi75B	5:**ATCTCATG**TTTTGTTTAGTTTGATAACAGAGGTGTCTCTGTAAGAATGCACAGATCTATAATGAAAGCAGTCCATTACTTTAGAAACTGTTTGTGCTCATTTAAGAGAAAATAAGCTGTTTAAAATAAAACATGCAG<br>3:**CATGAGAT**TCATTTGCTGCGCACTTGTTTGATAACAGAAGTGTCCCTTTAAATGGCGCGTACAGACGCTGATCTGGGATCAGTTTATTGTATGGAGCGCGTCTGTCAGTCAGCGCTTAAACATGCATTCACTGGTTCAGAGG<br>TTGCAAAGTGAAAGGCGACAATCAGCGCACAGTAAAGAAAGTGGGATAGAATAGTAAAATATGGGAGAATTTCGGCAAAAACGGGAGGGTTTACGTGAGTGAAGTGAACAGGAAGTGTTTGTGGAGAAACAGTTTTGCGAGAGA<br>ACGCAAAACATCTTTGCGAGGGAACGCAAAACATCTTTGCGAGATAACGCAAAACATCTTTGCGAGGGAACGCAAAACATCTTTGCGAGGGAACGCAAAACTTTGCGAGAGAACGCAAAAAACTTAGAAATATATATTTTCCTC<br>CCACCCATTTTTTTTCTTTCACCCATTATTTTTTTTCACTCACCCATTTTTTTTCTCCTCCACTACGTCCCTTCCGGGGTTCCGTA
ET33-mi75C	5:**TTAACAAT**TAATCTTTGCCCAAAACTGGCCCGCGCTGTACTGTACAGAAGAGCAGGCGAACGCCGGCACCGCAGCTCGCAG<br>3:**ATTGTTAA**TTGAGAGGAAGAGGGGGAGAAACTGCATTAGCATGCCAGGACAAA
ET33-mi76A	5:**CTAATAAA**ATGTATTTCGGCTACAATTATTATAATTTTCTAATAAAAGTCCAGAGCTTTGTTTTTTTTTTACCA<br>3:**TTTATTAG**TAAACTACTACTAACTGATAAAAAATTAAATGCCAACTGGCCAAGCTAGGACTTGAACCAGCAACTTTTTTGCTGTGAGGCAACGGTGCTAACCACTGAGCCACTGTGCCACCAAAAAATGAAATTATGGATGT<br>TTAATAAAATATTCACATTTTAAAATGTAAAACTGTTTTCTGTATATCTCCCAACGACAATGGAAAATATTATTGTATTGCATTAAAGCTATAATTTTTATTGGAAATTGTGAATACTGCTTTATATAGCCATTACATTACATA<br>TTACTGCATTTTTATTAGTGCATATCAATGTACATTAAATTTTTATGAGTTTGCAGCA
ET33-mi76B*	5:**ATTCTAGC**ACATAAATCTTAGCATTAGATCAGTGGTTCCCAACCTGGGGTCCGCGCCCCCCTAAGGGGGGCGCCAGAGTTCACAAGGGGGGCGCGGGAGAGGATAAGTGTTGAGGTAGAAAAAGGCATAAAAATGCTCCATA<br>TTATTTAGGGCTGTACAATTAATCGAAAATCCGATTTCGATTTCAAACGACTGTAAAAAAGCATTAATCGAGATAAACGCTTATTGCATCATATACCGCCATCTTTCTAGTTGTACACGTGTTGCTCCTAAAAGCCCGAACGAG<br>CGCGTGCTTATGTGTGTGTGCGCAGCAAGCACACTCAGTCAAAAGCATGCGGCTGGTCAGCATTCGCTCATTCGCACTCTGAGACGAGCA<br>3:**GCTAGAAT**TACATGCAAATTAGTGTATATTTAGTTAAACAATGCCTCATTTGCATATCTAAACATATAATTGTAAGAATATTGTAAATTATTTGCAAACGTTTGtcattcttaaTGTTATAAATCAACTGGAGATAACTCTT<br>AGCTATTTTTAAATCCAATTCACCTGCGTTGTTTTGCTTTAAAGGAATAGTTCACCTAAATAACAAAAGTTCTGTCATTTACTCAC
ET33-mi77-1	3:**TATTTATA**AAGTGCTTATTAATGCCTATTAGAGTTAATACTTAACAAATAATGAACTAACTATTTGCTAATGCTTAACAAATGATTCATAGTGTGTAGTTATTATGAAGTGTTACCCATTTAACTGAACACAGTGCCTAAAC<br>AGTGGCTCTCATGAAACAGATTTAGTACTCAAATTGTACATTAGGTCTTTGTAACATCAGACCATTGACACAACACTGTTTACAGTATTAGTTTAACATCTTGTTTTTAAGAGAAAGCACACGTTGCGTACATCCAGTCCCAAG<br>CTCCATGAAAACATTCTGAATTGTGTCAAAAGTGTACCGGAGTTCTTTGGGATAGGAAAAGTTGAGTGCATAAAGAAGGCCAAAGAGGTATGCTACAGCACTTGAGGTATCTGGAATGTCTTCCAAAACAACAACCTCTTCC
ET33-mi77-2	5:**AGGACTAA**TCTAATGCATGGCTAGAGTTAGGCTTTTTTTAAACTTATGATTTAATTCACAGAAGGCTCACTGAATGTCCACATGGTGGCACTGCGTCTTCAAAGACAGTCAAGGATGCTTGCAGGCACAGATAATCATCATG<br>CTTTGGTGAGTGTAACTCGTCCTGAAAAATGTGCTTTGCTTTTCAGTCTCCGCTCCGAGAAACCAGTCAAATCTGTGCCTCTAAGTGCATATCATTTTCAACCAAATGCTTTTGTCTAAAATCTGAGGCAAATTTCTCAAACGC<br>TTTTCTTCTGATCTTGGCTTTTAACTGTCTTCGAGGACCAATGAGTGCTTTACTTTGATGTGAACTAACCTTTGTGCGTCGCCTCTACCAACTTCACAAGTCTCTTTTTGCATTATGAATGCAAGCATTTTTTTTTCTTTTTAA<br>TAAAAAAAATGGAGTGTCATGGTTAGCTTTTTGACTAATGAAAATCTACCCACTCTGTGACTGGTGTGCTGTAATTCATGTTATGTCATTTCAAAAAGTTGGGAAAATTACATATAAAATAATCTGCTGTACATTTCGAGGGGT<br>CCAGTTTAAAAAAGGTCATGATTAGAA
ET33-mi78*	5:**GTAAACAT**TGATGGACAGCCATTTTTAGATCTCTTCATAGATGCTCAGTTTGGTTTTAGTCAGGGCTCTTGCTAGGCCATTCAAGTTGTTGTGAAGCCACTCCTTCGTTATTTTAGCTGGAATATCAAAAAAAATTGACAGT<br>TAAATATATCATATATATCACAATATATATATCTGAATATTTAGAACCATGTGATATTGCAGTTTTTGCAAAAAATGCCAACAATTCTGTGTTTGTCAATATGGGGTACTGTGTATACATTAATGAGGAAAATATCAACTTAAA<br>CGATTTTAGCAAATGGCTGCAATATAACAAAGAGTGAAAAATTTAAAAGGTCTGAATACTTTCCGTACTCACTTTAAGTCTGAAAGTTTAGGGGGAGTATTAATTAATATATAATGTTAATACATTTAATATAAGCAAGAAAAA<br>CCAATAGATGCATTCATACCTTCAAAGCCCTAACAATTGA<br>3:**ATGTTTAC**CATGCAATGTGAAGAACTGGAGAGGCTCTACAAGGAGGAATGCCAGTGAATACCCAAATCCAGGTGTGAAAAACCAAAAAGACTCATGGCTGTATTAGATGCTTTAAAAAAATACTGAGCAAAGGCTCTGaata<br>cttaagactatgcaatatttcagtttttattaaatatttctgttaatttatataTTTTCCATTATGAAAGTAAAATGCATTGGCTGAATGGCGGAATTATTTGTGAAAACTGAAAAAATAGATTTATTTATTTATTTATTTGAT<br>TATTTTTTTATTTATTCTCA
ET33-mi79	5:**CCCATTGG**TCGTTTAGATCCTCCTTTATCACTATTGTTTCAGGTTCTTCATATTCCACCTTTCCTTGCTGTTGAACACATGAAATAAGGTTTTGTGTTACTACATAACCCTGGCTGGCACATATAATATAAAAATCATTTTG<br>ACATGTTAAAGCTTATCTAGGAATTTGGTAATTGTAATGAATATGTAAAATGTGTAGGAATCACATTGTTCCAAATATCTGATGCTGGAAAAAGGGGGTAAAAACCACCACTATATGTTTTACTAGTATTTAGCAGTGAATATT<br>TTTGCTACAATTTGATATGTGCAATTAATGATATTAAAGTTTTTCTGTGCACATTGGTGAATGTATTTTGGAAATGTTTTAAATGTTCTCACTGTCCTGAGTAAATGCTTAATTTATGACAAGCAAATGTTTTCATAAAAATAA

	TTGTTTCCTACATCTGCATTCTTTTGAA<br>3:**CCAATGGG**AAATAAGCCAGGCACAAACTATAACTGTGCAAGATGGTAAGTAGAGGGCCAAAAAGCAGTCCTTTAATTGCTGCTTAAAAGAATGTTGGGGTAGATGTATAGAATTTAATGTTTAACCCCATAACTCTCACAGT TTTTCTGTTGTGTGTAAACCTGAAATTGTCCAGACAATCTGTGTATCATGTGCAAATGTTATTTTTAAGTTTGAACTCAAAAATAGTAGTCACAGATAAGGGGTTAAAGAGTTAGTTTACCC
ET33-mi80	5:**TATATATA**TATATATATATATATATA
ET33-mi81	5:**TGTGTGTG**TGTGTGTGTGTGTGTGTGTGTGTGTGTGTGTGTGTGTGTGT
ET33-mi82	5:**CGTTACAC**CAATGTTGGTGGGTTCTTTGCCCTCAATTAGCTTATATATCGACTCCAGTGCTTCTTCTTTGCTTTGACCTTTGAACCTCTTTGGGGCCAATTCTTCTAGAGCTTTCTGGAACTCCTCATATGTTATGACCCGA GATGTCTTTGCTCTGAATCATAAGGTATATGGGGATTTTCAATATCTTGTGTACATTTCCAG<br>3:**GTGTAACG**GTAAGGGCACTTTTCGGAACCTTTTTTAAGAGTGTAAATAACATTTAGAAATGAGTGGAAATCAACATATTA
ET33-mi83*	5:**CATATTCT**CCATTTTCTTCCTCTAAATTtcggtaagTGGTACAGTAACTTGCAAAATAGTTATCATGGCGATGACCAAATAAACGAGTTTATTATAAAATCCTGTGTTTAATATGTGTTTGGTAAACATCTCTTCGTCAAGC AATCTTCACAGAAGGGCTAATAATTTTGACGTCAACTGTAAGTAAACACGCCAAATGAGAACAGATGGGTTAGATTTGGGCGCCATCTTGTGTGTGGCAAACGCACGTGCACCCGCGCAGCGTCATGTTCATTCATTCATTCCC TCCTTCACTCATTTCTAAT<br>3:**AGAATATG**CATTATACTATATCCCCAACGCAAATAGACAAAGTCTACAAATTTGATAGAAAGATTTTCTGAATGAAGATGGTCGCTAGTTTTGACATGAGAAAACAATCTAAGGGTAGCTACTTGATAAATCAAGTTCAGTT TGATTCCATCGGGCGGATTTCGCGGGAAACAGCACAAA
ET33-mi84	3:**ACTGCAGG**GCATCAGTAAAATGACAAAAATGCAACAAATAATCCTAATCAGTTATATACAACAAATCATAAATATTAGTATAAAAGTATTACAAAAAAAAAAAAAAAAAAAA
ET33-mi85A*	5:**TTTATTAT**TTATTTCACGTCTGAATGTGTTTTAAGCAAAATGTTCTCAACCAAGCTTATTTTGCAGCCCTCGTTTTGAGCATAGATGGTGTAGTTTCAGCTTTAACTTACTATGTATTGCTTGTAAACACTGGGGAAACTAA TTGCCTTTTCTGTGCAATATAGCAAAAGTAGGATATCAGTTGCATGCTCTTTCAGTGAGTTAAGTTACAGTATGAGCATTAAAAAAACCAATGTGTACATTAAATCTGTTTATCTGCATGACATTGGATCAAACAAGTATTGTAC AAGCCACAAATGTACAATTGTCAACATTTCTTCTACTTCATGTTGTTTCAAATCTCATCTTTGGAAAACAAATAGAGTCTAAACATATAATATCAAAGCTTTCATGAAGAATTCATTGATGCTTGAAAACATTCATAAGCAGCA GTTTAATCCAGGTCTTCTGAGGGGACACAATTGCTTCATGTGATAAACTTGTATCTTTGTgatctttgtAGATTTGTAACAACAGCAG<br>3:**ATAATAAA**TACTCACATATTTTAGAGAAAGAAATACTGACGATGAAGGAAATCAGTAGTGTCGCTTTCACTTGAAATGATAGAGAGAGCTAACTAGTATTATTTGAGATCCATTTAATTGGTTTTCAGCGGTTTTAGGTGTT CCTGACTCTCCTACCGTGATGCGGTATAACCATAATAAAACTGCTCATCAGG
ET33-mi85B	3:**GACAACCA**TACACTCTGGCATTTACACACACAAACTACAGCCAATTTACCTTATTTAATTCACCAACAGCACATATCTTTGGACTGTGGGGAAAACCGGAGCACCTAGAGGAAACCCACGCGAATACAGAACAAACACCTGA
ET33-mi86A	5:**TGGACCAC**ATGGGAAAGCTAGGTTGCTGCCGGAAGAGGTGTTGTTGAGGCCAACAGGGGGAGCTCAACTGTGGTCTGTGTCCTAAGGCCCCAGTATATTGATGGGGACTCTATACTCTTCAGTGACGTTAAACCGAGGTTCT GACTCTGTGGTCACTTAAAATTCTTTCAAAAATCAGCATAGGTTTAACCCCTGCACCCTGGCCAAATTTGTCCTTCTGTCCATCATGACCATCCCCATATCATAATTGACTGTGCGTTATGCTTGTTTTAGGATATTTAGAATT TATTGCTGTAAGGAATTTTCAAACAAACAGTTTGACATAAAATTTGTTTTAATATATAATTTAAGTATACTTTACTGTCACAGTTGATCAACTTAAAGGAATAGTTCACCCCAAAAAATGTTAAATCTGTTAATTTACTTCAT TTAAAATGAAGGTGAATTATTTTCCTTCAGTTGAAAATTACGGAGGAATCTTATCTAAATTCTTGGTGATCCAAATAATCCAGTCAATGTTTACCGTTTTTACTATTATTATTAAACATAAACATATAAAAACAAGTCCAAATC AATGCCCATGGCTCTAGGCAACACACTGATGTATGCAAGTTTGCATGAGAATTGTTTGGACTTGTTTGTATGTGTTTATGTTCGATCTCAAAAATGGATGACCACTGACATAATACAG<br>3:**GTGGTCCA**CAATCAAATTCACAGCAAGCAAAAGACACACACACACACACACACACACACACAC
ET33-mi86B*	5:**ATACTACT**AAATCTAATGTTGGCAAAATCATTAGTGTACTTTAAATCCAATCTTATAGAGTGGAGGAACTAAGACGGTAGGCTAATCGGTTGCTAGCATAAAACTGGTTCCCTCAATAAAATCCCTataggATTTTCACATA GGCTTTTCGAAAAATGCAAATAATAAGCACTGTGTTCAAACACTGTTCATTACACTTACACGTTTGTCCAGTCGGATAATCCTTAAACGAAAATACAGCTTTGAGCACTTTTGGATCCTTAATGCAAACGCAAGTATTGAAAAG CTAACATATAGGCTATAAACAGTATACAGTGCCCTTGTGGAACTCGCCTGTGACATCAGCAC<br>3:**AGTAGTAT**CAAAAGACGATGTATCAGAAGGATGGTCTTGATTAATACCTCTAAATAATATATGGGAACCAAGATTATAATAGTTTTGGATTTTTCATTAGTTTTAGTTTTTATTTCGTTTTGACATTTTGTTTTCAAATTAA GTTAGTTTTAATTAGTTTTAGAGGTAGATTTACTAGTTTTTATTATTTTTTTTAAATTCTGAAATTGCTTAGTTTTAGTTTTTTTTTTTT
ET33-mi87	5:**GCAGCCAG**CAAATAGAGCATTACAGTAATCCAGTCTCGAAGTCATAAAAGCATGGACTAGCTTTTCTGCATCTGAGATGGATAGCATATTTCGTAATTTAGCGATATTTCTCAAATGAAAGAAGGCAGTTTTTGTGACATGG GATATATGGTTTTTAAAAGTTAGATTGCTGTCTAATATGACACCCAGATCTTTTATAGTAGAGCTAAAGCTAAAGCAGCCACTACTTTATTTTATCCTGTTTATGGGGATTTGACAAATCAGTCCAGTGATTCCTGTCCAAGGT AAATCGCATCAAAACAAACGACCTCAAGACATGACCCAATTGACGCAGCCGAGGCTCGATCCAGTGACCTTCTTGCTGTGCGGTGCTGTGAGGCAGCGCTATCCACTGCGCCACCAATAATATGTTATTCATATTAGCTTTTTT AAAAACTGGATAACGCTGAGGAGGGAGCTGGAAGTGAGGGGGTTGATGAAGAGAAAGAAGGATTTGAAGAGCAGAAATAGCAGTGAAGAAGTAGGGCAG<br>3:**CTGGCTGC**CCAGCATCCTCTATTAACAAACTTCAATTAGTACAAAATGCAGCAGCCAGAGTTCTGACCAGGTCTAGAAAATATGATCATATAACCCCAATTTTATCCTCCTTACACTGGCTGCCTGTTAAATTTCGTATTGA ATTTAAAATATTACTTCTCACCTATAAAGCTCTAAATAATCTAGCTCCTGTTTATCTAACCAACCTTCTGTCTCGCTACAAACCAACTCGCTCCTTAAGATCTCAAAATTCAGGGCTTCTGGTAGTACCTAGAATAGCAAAATC AAGTAAAGGAGGTCGAGCCTTCTCTTTCATGGCTCCTACACTCTGGAATAGCCTTCCTGGTAATGTCCGAGGCTCAGACACACTCTCCCAGTTCAAAACTAGATTAAAGACCTATCTGTTTAGTAAAGCATACACTCAATGCAT CACCTAGCGGGTTCCACACTGGCTTCTACATCTTGCTTATATACACTATGAACAGCA
ET33-mi88	5:**TCTTTCAT**CCAGCGAATTCATCCTCGGTAAGTGTTTTAAAGTCAAATTCTGTCCGTTAAAGGATTGTTTTGTATGTATTTCTGTGTATTGTGTGTGTAATTTAAAAGAAATAACCAAGTGACATTAGTTGTAGGTTCTTATG TTATGTCACGCTGTCCGTTAACAAACGGTATTTTAAACAAGAAATAACAGACGCCAGGCGGTCATTGTAGTGTTTGCGAGGTATTAGATACAGTAAGCTTATCTTGAGTGAGTTTTTGACCTTACGTTAAGTTACCTGAAAAAA CATAACTTTAACTTATTGTAACTGCATTAATGAATTCTCTATTTCA
ET33-mi89A	5:**CCTACTTC**TCAAACTTAAACAGAAACACTTCTAGCTGGGAGACAAACCTGGAAAACTACTTGCAAGACAACTAAAAGGAGAACGAGCCAACAAAGTAATTCATATGGTAAAAACAAAAACAGGTGATTTAATAACAAATCCT GATGAAATAAATGGTTGTTTTAGGGAATTTTATTCAAATTTATACACTTCTAAAGCTAATCACAATGACCCAAATTACGACAACTTTTCCTCAAAATTGGACATGCCAAAGCTCAGTGAGGTCTCTAAACTAGAACTGGAATCC GAAATATTAATCGAAAATATATTAAAAGCAATTCATCCCTCAGGGAAAGCCCCAGGCCCAAATGGCTTTGGGTGTGAGTTTTTTAAAAACCTTTAGCAAGAAACTAGCCCCCTATATGCTGAGAATGCTGCAAGACTCAATTAA AAAAAATACACTTCCAGATTCATTGTATGACGCAAATATCTGTGTAATTTGGAAAAAAGGGAAACCTGAGACCGATCCAGCCAATTACAGGCCCATTGCCCTTTTAAATCTTGATCAAAAAGTGTTGACTAAAATATTAGCTGA TAGACTGGCACGACACATTTCTACAATTATCCATCCAGATCAGACAGGCTTTATTCCAGGAAGGTTCTCATTCTGTAATGTCCGCCTTTTACTTAATATTTAATATGCAAATATTTTATAAATTAACCAACAAACCAGCTGCAA TTCTCTCATTAGACGCGCAGAAAGCCTTTGATCA<br>3:**GAAGTAGG**TTACTTATCTGTCTATTTAGGATAGAATTATACTCATATTTTAATTTTAACATTTTATTATAATCAGAATTGGATAGTGACTTTTTGTATGCTTGTTCATAGTCCCCGCATTACAGCTTTTAAAGTAAGACGGA GTCTGAGACTTCACCATTATCATTAGTTGAGAAATACTCATCCATTTTATCATTTAAGTATGACAAAAAAGTTGGATCTTGGATTAACATAGGGTTGAAACGCCAGCAATAATTACGCTTTGGCAGAATATTTTCAAGTTGGAG

	CGTGACAGGGCTATGGTCAGATATTATTATGTTGTTATATTTGGTTTTGCCAATGTTAGAAATCAGTTCAGATGCTACAAGAAAGTAATCGATTCGAGAATATGATTTATGCACGTGTGAATAAAACGAGTAGTCTATATCCGT<br>TGGATGTTGAAGCCTCCAGATATCAACTAGATTTCTAGATTTAATTAGGTTATGAAACATTTGCACTAAAGTAATCGTGGGTGTTGGTTTTGTAGAAAGTCTATCCATATATGGGTCTAAGTAGCAATTAAAATTGCCACCTAT<br>TATAAGGTTAGTGGTATTTGTGTCTGGAATTATGTTAAATATTTTCCGAAAAAAATTTGGGTCATCAAAATTGGGACCGTAGATGTTGAGTAAAGTTATGGGGAGCGAGTTGATATGACCGATCACCATTATAAACCTTCCCTG<br>AGGATCAGCAATCATTGATTAGAGGCGAAATACCACAGTTTTGCGAAA
ET33-mi89B	5:**CAAGTTGT**CCACAAAAAACGAAAGTATTTCAGCTCAATTTAAATAAGTTTAACTTAAATAAGTAAATTTGCAAATAGCAACATCATTTTTTTTAGTGTGTGATTTTTTTATCTTGACATTCCTATTTCAATATTATGCACAC<br>AACTTGTACTATCTGTATAAACGTGTTTTACAGCATTCTGGTTATGGAAATAGCATGAAATGAAATAATTTGCCAGTTTAATTATCATATAAAATGCCTAATTTGACAGAAC<br>3:**ACAACTTG**TTTTGTTTCTTCCACAAACATCTGTAAAAACTAATAAGTTTACTTAATTCCTTCACATAGTCCCAAAACAAATTAATTGTGTGGAATTCTTCATTTTTACAGTGCAAGTCAACAAATTAAAAATAAAAAACATC<br>AAAATAATAAAATACTTTATACATTTTTTTTTTTTAAAAAACCCCT
ET33-mi90	5:**CTTGTACA**GTAAGTCAATGAAAGGTTTTCTGTTACATAAAACTGTATGCACACAACACCTAAGGCCCCGTTTACACTGTCAGGCCTTGATGCCCGATTCCGATTTGTTGCCTATATCTGAATATTTTGCCTGTCCGTTTACA<br>CATTCTTTTAATTGTGACCCATATCATGTGTTTACACTTGCCATACAATTTATGATGTCGCGCATGCATAAAGGCTTGTTTTAGCATCTGCGCCACACTAGCCACGGCGGAAAAAATTGCTTTGTTTTTAGCTCTGCGAAATAT<br>GCGAAGTGTAGTATAAGCAGTGAATGGTTCAGTCCAAATTTACCCCCACTCATTTTATGTAATGGTATGGCCCTGATGTGTGTGTGTAGTTAAAAATGTATCATTTGCCTGGGTACGCGCTGGTGTTGGAAAGAATTAAGTTGT<br>ACTATGTGTAGTCCGCAGTGCGTGTATTAATAGCAACAAAAAGCAAAA<br>3:**TGTACAAG**GAAGTCTTTCTGAAAGATGTGGGTGCAAACAAAAGTTGAACAATGAAGTCCACAGACTTCAGTCTGTCTTTGTCTCAAGTCCTGATAATGTTGAACAATGTAGATCCTCAAAGAAAGCACATCTTCTAGTGCAT<br>ACTTCTTTTGGGAATACCTTAAAATGAAATTACATTTTTACTTTACTCTAAAGAGTATTAAGTAGTGATATTTGGGTTTTTTAGATCGCCGTTAGCCACTACCAACTTAAGAGACTATTCTGTCTTAGGTCCAACAAATAAAAC<br>AACAGCTTTACATCAAATCATCATCAAAACCAATACAAAAAAACATCTCACAAAAAAGAAACAAATTTACATTACAATAAAACTCCAGAAACAAATGTCAAAAGCATACATTTACACTAAACCATAACAACAGAGAGCAATACC<br>ACAACCAAACAAAGACAATTTTACATCAAAGCTGGAAGCAGCGATGAAAGGGACCTCGCATGGCCTTAGGATGACAGAGAACAGTGGCATGTAGATGTCTTCAGGAGAGGAATCTCATTGAACATGAGTTTTTTTTGGTAGATC<br>GGGCATTGTGTGTCTGAGTTATAAGTACTTCCTCCTCCATGGCAATGTTTTCCCCCACAGTCCAAAAACATGGGGTATAGGTGAATTAGGTAGGCTAATTTGTCCGTAGTGTATGAGTGTGTGCGTGAATGTGTGTGTGGGTTG<br>CAGCTGGAAGGGCATCCGCTGTGTAAAAACTTGCTGGATGAGTTGGCGGTTCATTCTGCTGTGGCGACCCCGGATTAATAAAGGGACTAAGCCGACA
ET33-mi91A	5:**GCCGTTGT**GCAAAGCACCATACACACTGGACCTTACTCAAATCTTGTGATAGTGATCAAGTTCAAGAAATCAACTCAATTTCGATATACAAATTTAGAATAATAAAAAAACACACTTGCATGAATGATGTTAATGACATAAA<br>TTCCATGACATAGGCCTGCTATTTGGATGTAAGCATGTATGAATGTAGACTAATCAAATAAGTAAACCAAAAATCTAAATAAATCAATATCCTATAATGATGTAATTGCAAATATTTTTAGTATTGAACATAGGGCTACAGTCA<br>ATGACAATGACATTATTTGTCTGTACAAACTCTGAAATTGTTGGTTAATATTGCATATTCAAGAGATATTGCACAAAGAAAAGATTTTTTATCTATATAATCAGTGATGCTTTCTTGATTCAAAAAATGTATGAATTCTCCAGT<br>GACTCAAACAGATGATGATTTATGAACCATTGCCTCAATCTCGTCAG
ET33-mi91B	5:**TGAACACG**TTTCCCAGTTTGGCATCCTGACCTCACTTCAACCTTCTTTTTTCCTGTCATATTACCTTGGCTTTTTATTTGTGCAAGGAAGGGAAACGATAATGTAAGTGAACTGTCTGAATAAAACAGAATCGCTAACTTGC<br>TGACTCGCTTCTTTTGTATTCAAGTATGTTATTGGCGGGAGTAGCCACGCACGTGCTGTAATCAAACGTCATTCGGCTGCGTTTTTAATGGCTGCCAAAACAACAAATAGGTTTATGACTGACTTACCTCAGCTTAACTATTCC<br>TTTATCTTGTCTCCTGGGCTGACGAGTGGAGGCGACAGTGGCTCAGTGGTAAGCACTAATGCTCACAGCAAGGAGATTGCTGGTTCGAGTGCCCTCTGGATCAGTTGACATTTCTGTGTGGAGTTTGCATGTTCTTCCCGTGCT<br>AGTGTGAGGTCAGGTACGGTCAGAACCCATTAAGCAACA
ET33-mi91C	3:**CCATTCTG**CAGACAGATTAAAACAGTCTCAGCCAGTACCTACTTGCACTTAGAAAGGCAGACTTAAGGGCATCGGACCAGGATGAGCTTGCTGCTCTTCTGAAGGCATTAAACGTGTCCACTCTCTGGGCACAAAGTTGAGG<br>CTTGAAGCCGATCTCAAAATGTCACTGAGAGATTACAAAAAAATCAGCTCATGATCTCTTCCTGGTTTAAAAGGACATTCACCTTCACAGTCCTCTGTCTGGGCTGTATTTACGCTGCAGGTAAATGTTGCTCAAGCTGTGAGT<br>AATGTGCTGTTTTAAGGTTTAATCTGTGCCAATTTCATGTGTGGTATTGAATTTAACAGTAATCAAATGTTTAGCAATGCAACCAGCAAGCATGCTCTGCCTTTTCAATGATTGCCCATTAACCCTTTGACAAGTACCATCACA<br>CAGGCCCCGTTTACTTTAGTGCATTTTAGTTTTAAAATGGCATTTTTGATCAAAAACGATCCACGTCTACACTAGCGTTTCACCTAGCGTCTCTGAACATATCTTCGCCCACACTACGCCACCAAAAACGTATATCACGTGACC<br>ATTTGCGCACTCTGGGCATGTGCATTCTAGTATAAACAGAAAGCATGTGTCTCGCTCGGCATTTGGTTATTGTTCATAAACAAACGCCGGTTAAACATTAAACGCGTTGGCAAAGAAAGCAAGAGAGGTATTTTTGTGGACAGA<br>CGACGAGGTCGAGTTGTTACTAAACGTAACAAATGAATAACTGCACAATGACGCCTTGTGTGTGGGCGGGTTAATGGCCGTATGAAAAACGGGCTTTAATTTGGAAACATGAAACACGGGATGTATTCTCCTGTACGCTGGAGG<br>GAGTTTGAGGC
ET33-mi92A	5:**GCTGATGG**TGTAGTGGTCAAAGCACACAGCCTGAGATGTGAAGTTCACACAGCATACCTGGGTTCAAATCTGAGTCCTGTTACTGCTCTTTACTGCTGATCTAAATACAAAAAAACAAAAGTGTTTTTCCTCAATCCTGCAC<br>TGATTACTTTCCTTTCAGCAGTTCCTTTAGTGTGATCCACACAGCAAAGTGAGTTTCGTACCCAAGCCTCTACCGTAGTCTCGTTCACATTGATTGTGTGTTCAGCCACTACCCCATCATGGCTTTCATGGTTCTTGGGTCACT<br>TTGTCTGACTGAATTCCAGCAAATGACAGTAAAGTAAGCAATAGAGGGATTTGAACCCAGGTCTCCTGCATGAAAGCACATTACTTTATGACTTGAGCCACTGAGAGGGATGAATTCAAACTGCTGGAAATAGGTTTTATTGAA<br>AGTTTTGTGAAAGATGTGATGTAATTGTAGCAAGTGAGGGTTTCGATCATTCAGTCTTCTGTAGAGCGGTCAAAGACTCGTCCACTGAGC<br>3:**CCATCAGC**ACACACACAAACACAAACTTCTTTCTCCACTGTAAGGATGACCACATTTACACACTTTGAAAAAAAAAAA
ET33-mi92B	5:**GTGAGGAG**CAACTTGAGTACTGCAGATGCGCTCATGTTCAGCGGTAAACACGAGTGGAAATCAGGGTATTGCCCGGTGTGTGTAGGCTATGTGTGTGTGTGTTTATTGAACCATGCTGCAAAGAAGTAGCCAAAATTAGACT<br>ATTTTCTGCATGTTTGTCATGTTTTTTAGTCAAAACATTATATTTATGTTATATATTTGCGTCAATTCGTTTTTAAAATTTCAATTTAATTCAAAAATAATCGTATTTTTGCTAACATACAAATTTAAATAATACAGATTATAA<br>AGAAAAATAAAATGAAACTCTTTACAAACTTTATGCGTTAATATTCAGCTGTAAACATAAGTGTAAATCGGAATACAGTATGTCTGTGTTCGCGTCTGTGTGTGTTTACGTGTCTATTGAACTATGCTGCATAAAGCAGGTAAT<br>TGTCATCACGTTTATTTATTATTGTCAAGCATATTTAATCAAAACATTTACTTTTTTAGTTTTCTTTTTAGCTTTTAAATGTCAATTGAATT<br>3:**CTCCTCAC**CATCTGTGTTTTGTTGTTGTCCACCATCCCGCGCTTCTCTCCCGTAACTCCCGGTGAGTGGTGACCGGTGCTCCGGTGTTTCCAGCAGCAGAAGCGCGATCACAGAGAGATCTGCTGCCGGAGATCAGCGCTCA<br>AATCCCGCAGAGTCCCTTCCGCTCCGCCCGCGACTCTCTGGCGCTTCCTCGCGCACTGAAAGAGAGAGACTACACAACTACGCACACACACACACACACACACGTAGTGTCTTTTTTCCAATATCAATAACACTGCTCTATTAT<br>GGGTTATTAGTTGAGTTTTGATTCCTGTTCTGAGGACTGAGGATGATTATAACACTCAAACAATAAACAACAGAACAACACACACAGCAC
ET33-mi93A-1	3:**CTTCTATG**AATCAGCGCACTTATTTGGGTACGACGTATCTGGACATTGCTAAAGGCGCGGTTGAGATCTTTATGAAGGTAAAGCATAATTTTACCCCGACTTTTTCAGCTTACTGTTGTACCTTGTTGGAGTTAAGGATCCC<br>TATGACAGGATCAGATGAAATGAATGTGTATTGTTTCGTTTTCTTTAACAGCTGCGTGCCCGAGACCCGGCTAGTAGAGGCGACAGGTACATGCTAGTTACATTCGATGATCCACCATACGGCGTAAAGGTAAATAAATACTAC<br>TAAATTGTGTTTATATGTGGTTAAAGCGGAGATATTTTTTCTGACAGTGAGACTCTACTGTCCCTCTGGTTCGTAGCATCGGGTCAGGCGGCCTCCCGGTGGCGAAAACGGCTAATTTTTGTTAAAATATCCGCAGACCCGGAG<br>CAGCGCAGCCTCTGAACATGGCGGACATTTTGCTTTTGTGTTGGGAAAGCTCTCGAGCGCTGAGATGGAGGCGGAGAGATTTATTATCCACCATGACGTAGCGCGTGACGCGTGCGCGGGCCAACTCGGCCCGGATTGGACGAG<br>CAGCCTGCCCTCTATCTCGTGCCCTCCTCTTGCATTCTCATTGGCCACTGTAGCCAG
ET33-mi93A-2	5:**ATTGAGTG**TGTGCAGATTAGCTCACTGAGTCTTTCATTTTACTCAGATATTAGATTGTCAGAACATTAGATGCCTGATTGCTCGTTGTTCAGTCATCATTTACATATTAGACATTAACCAGATATATATGAAAGTGACACAT<br>TTTGCTCAAACATGTAATCTGGAACGATACAAGCTGAGATAAAACTACTTCTAATACAAATACAATAGATCTAGTGCATATACCATGCAGAACAAACACCTG
ET33-mi93A-3**	3:cttcccatGAGCAATCAGGCATCTAATGTTCTGACAATCTAATATCTGAGTAAAATGAAAGACTCAGTGAGCTAATCTGCACACACTCAATCAGCTTTCACCTGTTTTGTGTTGGGAAAGCTCTCGAGCGCTGAGATGGAGG

	CGGAGAGATTTATTATCCACCATGACGTAGCGCGTGACGCGTGCGCGGGCCAACTCGGCCCGGATTGGACGAGCAGCCTGCCCTCTATCTCGTGCCCTCCTCTTGCATTCTCATTGGCCACTGTAGCCAGAACA
ET33-mi93B	3:**GTTATCTT**CATCTTTTTCTGTATAAACCAAACAAGAGCTCATCATCCTCTTGAAAATTAGTCTTCTGCAATTATTTGTAATTACACTTCACATTCCCTCAGAACCAACACCTG
ET33-mi94A*	5:**CCCTTTAC**GCAGAGCCGTACAACAGAATTTTGCATGCCCAAACTCTAGTGTGACTGCAGCATAAGAGTGTGAAGGTATAATTAGCAAAGTACTGTCACAGTTTTGCTTAAAATATTGCGACACACAACATATGAAAGTTCGA<br>AAAAATTATTGGTGTGCATCTCACTGGCACATCTGTGAGCAAAATAGAAAGTCTTTGTGATGTATCAAGACAGGAGCCACAGTATTCATGCTTATGTCAGCTTACCACCAAAAAGGATGCACTACATCCAACAGGAGTAACTGT<br>GGACCCAAGAAACAAGCAGCGGTTACACTAGACTTTTCTCCACATAGACTTCCATTCATACGCATGCAAATGTGTCAAACCAGAAACGCAGGGTCATGTGTCAAGTTTCACTGGTCGCTGCGGTGCAAAGGTAAAGCTTGGTGA<br>ACTCTGACCTGCGAAATTGCATcacttgactgcatAAGACCAATCGAGAATAAAAAACATGAACTCTCTGGACAGAAATTTTAAACTAGACCAATCACTCACTTTTTAAAAATGTCTAATCATCTTGTTTAATCCTATCCCTAA<br>TCACAGCGTCCATACTGCAGAATTTTGCATGCTCAAACTCGAGTGTGACCACAGCAAAAACTGTATG
ET33-mi94B	5:**AGCCTAAC**TAATGTTAAAAGTTATCATCACTTATTACAATACTTACCATGCTGATTTATGACATTTTGTTAAAATGTTTATTACAAGTTTTCTATAAATGCTCACATATATGGCATCGTTTATAAATACATTAATAAATACA<br>TTTCTGCCAAAATATTAAAGCATTCCATGTAGTCAGGTTCAAAATCTTTGTTTCAAAGGATTTTTATAGTTTTTATATCTCTTTTTATTAATCCACAATGAAAAATTTCCAAACAGACTGGATCCAGAACAAA<br>3:**GTTAGGCT**ATTTGTACCCTGTTAACCTGCAGCAGTGTTTTACCCATGCTATTTTAGAACGGAAGTGAACGTTAAGCTATTATCTTTAAATATGCAAGGCTTTCGGTTTATTAACGCTAGCTATAGTTTAACTAAAATATTTA<br>TTGCCGGAAACAGTAAAACACATGTTGAGGATTACATGATGAGTGCACATTGCGTCATTAAAATTTTCCAACTGTCGTCTGTGACGTGTCCCTTCGATAGCTCAGTTGGTAGAGCGGAGGACTGTAGCGGTTTATCACTGAAAT<br>CCTTAGGTCGCTGGTTCGAATCCGGCTCGAAGGAGACATTTTAACACCGTCCTGACAGGATGTTTTGTTGCCACGGTTATGGTTTGTATTCACAAAGAAATAAGCGTAACGTTAACGTTACCCACAATTATTTTGTTTCTGACT<br>TACAGGTAGGCTCAGCAGCA
ET33-mi94C	3:**CTATTGAC**GTACCCCAACACTATCCCTAAACATAAGCCTCACAGGAAACGGGACTTACTTTATAGTTTATTTATTACTCTAGGGTTTATAAGGCTGTTGAAAAATGGGAACAAGGGCAATGCACTCATGTTAGCCTTTTTTT<br>GTAATTCATAAGTTCGTTTTTTTGCAGAGCGAGTCTATTACGTAAGTGTGTGTAAATTGCTCATGCTGTATGTCGCCTTCTAGTCGTAATATCTGTGTCATACCCATTTCATTATACAAACGGTTGACACACGTACATGGTTAG<br>TTCGAGGTCTAAGGCGTACCTGCAGAACACTGCTGCACTTCAGACATGCAGGCGCTCAGACATGCATGCGTGCTGTGTGACGATGATGATGTATTAGTAGTGCACACTACACATGCAGACTACGCTTACCATCCAGCTCAAGGT<br>AAAATACAGACCGGCACGAGTCCTCCACACCTGACAAACCAGAACCAGTTTTACCAAACACACCAGAGAAACACCGGTGACCATCACATGCAGATTTCACTTATTTCATCTGGTACATGCATTATACTTACACTGCAGTGTGTT<br>TAACAGGAGATTCTACACAACAACAACAACA
ET33-mi94D	3:**GCAACAAT**GATGAGTATTTTTACATTACTGTGCATTAATAAAATACAGTTTAATGGGTAATTTAATAAACACTTGGCATAATTACTGTTTATGCAGTACTGTGATGATTGGGTTTAGGATTGGGGTGGAGGTAGAGGTTAAT<br>AAAATACAATTTAATGGGTTATTTAATAAATTAAGTAAATAATTCATGTTCTGGCCGCAGCTGTATCCCTTCTAGCAACACATTGAAAGATCATTTACGAATAAAGTAACTTAGTTTTAAATAGATTTTCAGGGTTTTATATTT<br>TCAGGTTTAAGTTTGTATGTTTTAAAAAATATATATATAAAATACAAGCTATAATATTAGATATGGAAGATTATAAATACTATTTTCGAAATTAACTGGAAACAAGTGTAATAAATTACTGAAATAAATAAATAAAATCTAAAT<br>TTAACTAATAGAAAATTACATAAATGAACTAAAATTAAAATATAAAGAGAAAATATAACTTTTTAAAATATAAATTATTAAATAAATATAAATTCTGAGTAAATGCTCAATAAAAATAAAACAACAGCGAGTGTGCTCAGAAAA<br>CAGGCACATCATCTGATATTGCATTAATATTCAGA
ET33-mi95	5:**CTTCTGTG**GAGAGAGGAAAACCTTAAAGAAGGACAACCATGTGCAGCAATCCACCAATCAGGCCTGTATGGTAGAGTGGCCAGACAGAAGCCACTCCCCGCCTGGAATTTGCCAAAAGGCATCTGAAGGACTCACAGACCAT<br>AAAAAAGAAAATTCTCTGGTCTGATGAGACTAAAATTGAATTCATTGGAGTCAATGCCAGGCGTTATAGTTGGAGAAAAGTAGGCACAACTGATCACCAAACTGATAACATCCTCACAGTGGTGGCAGCGTCATGTTGGGGGGA<br>TGATTTTTAGCAGCA<br>3:**CACAGAAG**AATGTTGGAGCTCAGACAAAGTAACCATCAGGTTATTGATCACCTCCCCCAATAACGTCCTTTTCCTCCGAACACTCAGCTTAGATGGCTGGCCAGCTCTAGGAAGAGTCCTGGTGGTTCAAAACATCTTCCAC<br>TTACGATGATGGAGGCCACTGTGCTCACTGGAACTTTCAGCGCAGTAAAAATGTTTTTGTAACCTTCCCCAGCCTTGTGCTTCGAGACAATCCTGTCTACGGAGGTCTACAGACAATTCCTTTGTCTTCATTCTTGGTTTGTGC<br>TTTAACATGCACTGTCAACCCTGGGACCTTAAATAGACAGGTGAATGCTTTTTCAAATCATGTCCAATCAACGGAATTTACCACAGGTGAACTCCAATTAAGCTGCTGAAATATCTCAAGAATGATCAGTGAAAATAGAATGTA<br>CCTGAGCTCAATGTAGAGCTTTGCGGCAAAGCCTGTGAATACTTGTGTACATGTGATTTTTCAGGTTTTTTATAAATTTGCCACAATTTCAACATTTTTTCAATTTCTTTTTACACATTCTCATTATGGGGTATTGTGTGTAGA<br>ATTTGGAGGAC
ET33-mi96A	5:**GAAATTAT**GTAAATGGAAACTATGATTTCAATCAATGTATGTTTTGGTACAACACAGTCAAGTATGAATACCTTGTTTTTATGCACGTTTTTACCAACACATCTCATAATGATGACAAACAGTCTTTTGAGGCTGTTTTAAT<br>GTATTAAACAACATGGGCATTTACATTATAAATGCAATCTAATTAAATCCATTTTTGAGTACCAAAATCTAGGGATGTAACGATTCATCATGAGCCGGTTGAAAATCAATTATTAAATTCAAATATGTGACGATTCAAACCAGT<br>AAAAATGTTAAATGAATCGCCATACATATTTTGAACAAAGGGGGTGCTGTATTTACAAGCACAAACTCGCTTTACCTGCACATCAGCGCCAAGCAAGAGCTGGGCGACAGAATAAAATGACAGTGGTGAAGTGCTGCAAACAAA<br>ACACAAACTGTGTGCAAATACTGCATAAAGACATTTCCTTACACTAACAAAACAATGAATTTGATGCAGCACAGTAAAAAACTATTCTCAAAGATGTCTATCACAAAAAGCTATTAATAGGCCAAACCACGCAGTCGGAGGATT<br>GGCAGCTCCGCTTGCTTCGATGAGTCCTAACTTTACACAGTGTTCGAACTATATCATGGCCTTTGGGGAGCCAACTTTTTAATTTTTTTTTAACAGGAGGGGTTATGGTTGCTTTGATCTTTAGAGTCTCTTGCCACAATGATG<br>TAGGCTAAAAACAGACAGGATTCCTGTCCTTACAAAGCAATGTATAAACAAAAAGTAGCTAAATAT
ET33-mi96C	5:**CAATCCTC**TAATTTGGAATTCCACTTTCCCAAACTTGGGGAATTTTATGCTTTCAGGCAAATCCTGAATTTCTGCAGGACCAGTTGCAAAAGACTGGCACACTGAGACAAGTCTTGCATTGGTGAGTGCAAATTTATGAGTA<br>TACTGATAATTTTCCAGCAAATTAAAGAAAAATTTATTTTCCTTTGGCTTAGTCCCTATATTCATTATTGGTTGCCACTGTGGAATGAACTGCCAACTTATCCAGCAAATGCTTTACAAAGCAAATGCCCTTCCAGCTGCAATC<br>CAGTACTGGGAAATATCCATACCAACTCATTCACACACATCCACAACAGCCAATTTAGTTTATTCAATTGAAAAAAAATTAAAAAAAATCATAATTTGGTTTCATAGTGAAATCTTTTAATTTAGTAAATCTGATACCTGGTAC<br>AGTGTGTGACATTGACATCTATCTAGAGCTCTTGCACCTGAAAGGCCAGTGACCTACAAAACATCTTTTAGCTTCAATTTGGCAAAAACACTGACATGTTGGTTCATGTGGGTTTAATATAGGGTGAGCTAAAATAAAACAGCG<br>GCCCTCTAGGAACAGGTCTGGAGGCACTTTGTCTAGAGGAAACATGAGATGTACATCTTATGACTGATGGAAAACAGCACTGCGCCTAAACATGCAG<br>3:**GAGGATTG**GCAGTCAACGCAAAGTGCCTGAATTGTCCCAATTAGGTCTTTTGTAGAGTTACATGAAGCCTGAAACCAGGGTTATACTAGATTTTTGTAGTTTATTTGTTAATTTCAGCTTTACTTGGGTAAGATTCTTTTAT<br>CCACAATTCACAACTTTTATATTGTCCCAGTCTTCCTTGAATTTCCCAGTCATTTTCCAGTAAATATTCTTGCTTTTGCATTATAGGCATTTTTTTTTTATCTGACTGGGTTTTGTTGCATTTGTTATAGCAGGTAAAATGAAA<br>ATAATTATTATAGCAGGTAAACTGAAGTCGCATCAAAATGAAAAACACTGACGTTGTCCAGCACTGCTCCTGCACTGTATTATTAACAATTGGAATCTTAAAATACCTTTCACAAACTTTATTTTATTATATTTTGATTTTATA<br>TATTTTGGGTGTGTCTTGACACATTTTATTCTCT
ET33-mi98B	5:**GTCAGGAC**CAGGAGAGAAAAAGAAAGAGAAAGAGGAAAACCACATGAAGAGACGAGCGTTTCATATCATTCTAATTAATACAGTGAGCACGGCTTTTATTTATATTCCATCCACTATCCCAGGACTGTTTTACATTATGACT<br>TATCAATATTTTCAGGAACCTTTTACTTTTGGTTTTTTTTGTTATTTAGCGGCTGGATTTGTTCAACCTGTTCTCTATCTGAATCGTGTAGGAAAACTCCCCTGGCATAAAATATCCTGTTGTTTTGTTTTTAGACATTGAATT<br>ACTACACTTAACAGCATCTATTCCTGATTATTAACAGCCCCTCTCTCCTAACAAATGAAATGCCTTATATTAATGTTTCAAAAAAAATGAGTGAGAGTGATAATAAATTTACTGAACCTTCATGTGTGAGTGAAAAGTTGCTTAA<br>AAAATGTTAGGGTGACTTTATTTTTAACTTTATTTTACTGTTTAT<br>3:**GTCCTGAC**TGGCTTCAGAAGCTCTGAGAACAGCCACAAGACAAAACAACAGGAAGGAAAGGAAGAGGAGGAACTTCGCAGAGAAAAAGCATATATAAATATACAAGTTACAAGTGACTCTAGTAAATACGCAGCACAAACAG<br>CCACCAAGAGTGAACATCCAAGCCGCTGCACAGCAGATCATTCTATATCTGAGAGGTTTGTACTTCAGAAAGGTTACAGGATGAACCACTGCCAGGTAACGCTCAACACACATCAGACACTGAAACACAGGACGGCCAGTGACG<br>GTTAATCCTAACATAAAATATTTTAAAGCCCTGAAACTTTTAAACCAGCCATAAAATATTTGGATCAAAACGTTCAGTAGAGAGTTGGCAAGCTCACAAACCGACAGATTGAGGTTGAAGATCTCCAATGCAATTCCACTTCCT<br>GTTCCAGTGACGATGAGCCATACAACATAAGAGTGTGTAGGAAAACCAAACAAAAAGTTAAAGGTGTACAAACAAATCTCCAGGTTTTTCAGTAGACCGGTGGAAGGAGTTGTGTAGAATGCAGAAGTTGCAGTTGTGGTGAAG

	TTCTCTGTACAGTTACTCATCTTCACCTCCTTTGTCTATCAGCTGATTATTAAAAAAATAAAAAAAACAATAC
ET33-mi99	5:**CTTGTGGG**GGGAAACTGAAGCACCTGGAGGAAACCCACGCCAACATGGGGAGAACATGCAAATTCAACACAGAAATGCCAACTGACCCAGCCGAGGCTCAAACCAGCGACCTCCTAGCTGTGAGGTGACATTGCTAGCCACT<br>GTGCCACCGTGTTGCCCTTAACATGTATTTAGTATGATAAAACAGTGCTTCTTCTCCAAGTGATGTAAAGCAAAAAAACAGGAAGCGGTGGATGTGGAATAATAAAAGCTCTGCTGTCTCTAATTTGCAATTGCTTCAGTGGTC<br>TCGTTTTAGCTTCTTTAGCCATTCTCTGAT<br>3:**CCCACAAG**TCCAAAGACATGCGCTATAGGTGAATTGGGGAGGTTAAATTGTTCGTAGTGTATGTGTATGTCCTGGTCATCCAGGTTGTTTTATGACCCACCTCATTAATATTAGATGTTAAAAAACACAAACATGGTACAGC<br>TAAATATCAACTTTGATTGAAGCCCTGAAGTAAGTAAATATTTTTTAAAGTTTTGTTGTAATGCTGCTCTGTGTAGTATAAAAATACACACAACATATATAATCTTCTTTCAGCATGTTTTCCTATTTTCTAAAAAAAATTTAA<br>TCAAGAGTGAAAAATGTCGTAGCATGATGAAAAACACTTTCAGAAATAAAGTTACAACGTCTGTCACTGGGGCAGTACCTTTTCAAAAATATATATTTATTTTTTGTTCCTATCAGGTTCATTCCTTAAAAGTGGGTACCTAAA<br>AAGCACAAAAGTGTACCTCATAGTGCCGTAAAGCTAAAAGTGTATTCTAAAGGTACAAAACTACCCTTAAGGTACTAATGTGCTGCTGTAATGTGCAAATTCTTTAAAAAAGTACAACCCCATTGACACCTTTTGTACCTTTGT<br>TTTTGAGAGTGCAGGACATGATCTAATTAAAAGAGCTTATTTCTCTGGATTTGAGCATTCTTCTGAACATTTGGAATATTGTAAGTATAAAAATCAGCCATATCACCCTGCAGCTCAAGAGCGGTTGCTCACTGAAGCTAAGCA<br>GGACTGAGCCTGGTCAGCAGCTTAATGGGAAACTACATGGGCACACTATTGTTTTGTAAGTGGTTCA
ET33-mi101-1	5:**TTGTTAGT**AAATCCAAACGTGAGCGATTCTGAGCATGAAATCAGGCGTACGCAAAGTTTTTGGGCGTACGCAGCGTTGATACATGAGGCCCCAGATGTTTTATAATGTGCTCAGCCTGACGTTTTGTCCATTCACACAGGTT<br>TTAAGCATCACATGATCTCTTTTAACAAAATCACATGACCTTTTTTAAAGCGCATGCTGGAGTTTGTGGGGTAAAAGAGTTTCCATTGCAGTTTATGCACATCTTTTCTTATCGAATAAAAAGTTTATCCTACCCAGTTAACTT<br>ATGCGCATTTTTAAAATTTATGCGCATCATGGCGTTTCCATCAACTGTTTTTTTATGCATGCACACAAAGGGTTGCCAACTTAGCAATTTTGTTGCTAGATTTAGCAACTTTACATAATATCCTAGCAAATTTTTTCAAAAAGC<br>ACCTAGCAACAAATTTAGCAATTTTTAACATTCATTTGGCTACTTTTAGCATTTTTTTTTTA<br>3:**ACTAACAA**TGAACTGAACGTGGGAATGTGCGCAGCTTCACGGCAGCTTTCTGGCTGGCGTACGCACATTTTTTGTGCGTTTCTGTTTTATTTCCATTGGCGACTCCTAGAGGCAGTTGTGTTAAATTCCTCTCTACAAAGTG<br>TCTGAGCCTTGCAATGGCAGCTGTATGAGACGGGTTCAGCAGGTATGTAAGGTTTCCATACCATACATTTGACCAGCTAAACATTAAAGCACAATTTGCAGCAGTCGCCTGTTTTCCCAATGTAATCTGAGCTATCTACTGCAC<br>ACACATTGCTATAAATACACTATCTGAAGATGAATTTGCATGCGTGAATCAGAAACATTTCCATTCAATAAATGTGCAAATAAAATATGATGCTTATTGATATGAACTTATTGATGATTCCTACTTGTCTTTCTCGTGATAAAT<br>AGTTGGCAAAATCTAATATGTAGCGGGGGAAAAAACAAGAAAGAGTTCATCAGACGCTGGATTTGAGCCGAGTTCATGCTCGAACGTATCAATTCATGATCACATTTGTCTTACGAGTAGCGCCACTGAGACTGTTAAGCGTCC<br>TGCAACATTTTACAGATATAAACCACACTATTTCTTTTGTTAATGCACTCAGTGCGATGTTCAGACCCAACTGTGTTGACCGCATCAGCTAAACTCTCCCACTCAATTTTTTTCTTTTGTTGTTAATTCCGGAGAACAAACTTG<br>CAAATAAAACCGCTTTTCTCCGGTCTACCTCCGAAAGCA
ET33-mi101-2*	3:**GTGTTTTT**ATACACTTACCGGCCACTTTATTAGGTACACTTTACTAGTACCGGGTTGGACCCACTTTGGCCTTCAGATCTGCCTTAATCCTACATGGCGTAGATTCAATAAGGTACtgggAATAATCCTCAGAGATTTTGCT<br>CTTTATTGACATGATAGCATCACACAGTTGCTGCAGATTTGTCGGCTGCACATCCATGATGTGAATCTCCCGTTCCACCACATCCCAAAGGTGCTTTATAGGATTGAGATATGGTGACTGTGGAGGCCATTTGAGTACAGTCAA<br>CTCATTGTCATGTTCAAGAAACCAGTCTGAAGTGATTATCCTGCTGGAGGTAGCCATCAGAAGATGGGTACACTGTGATCATTAAGGGATGGACATGGTCA
ET33-mi102	5:**GCCTGAAT**GGATGACTAATCCTGGTTGTAGGCTGCGGAAACGTCAGCCTAAACAATCTACTACATTTAAATGATTTCGGGAGATATTAAAGTTAAACAAGAATTTTACATTTATTTGTCAGGAAAAGATTTTCCATTGCAGT<br>TCACATAATTGTACAACATCAATTACTCAAAGATACATTTAGGAGTTAAAGAGGAATACCTGACCATGTAAAAACACATTGCAGCATATAAATCCTGATGCAAAGCTCAAAGACTCACAACCTGCAATGGGGTATCCTGTCTAC<br>AGGATCCCACGGACACTTCTGCACCGTTTGCCTAAATACTCAAATCATTATAAATTCAAAACAATAAAAGCTTCACTAAAACTCTTGCCTGGTCATGAGTTGATGCGAAAACCATTTTGCCTGGAGTGGAGCATCTGGCAAAGA<br>TCTTTTCAAAGTCAAAACCCAAATTAACTGATATAAAGGAGATTGTAAAATATTACAGTGAAATTAAGACCACTTTACCAATCACACAGAGACATTTAAAATCACACCAAACATGAATATAAAGCCACAAGATACACCATATTA<br>AAACTTAAAGATTCGGTGTGAAATGTGAGTGAGCTGCTCTGGTGGAGAAGGAAAAGTCCAGGGC<br>3:**ATTCAGGC**GCAATGGATGGAAGCATGGGCACAGCATTAGAGACCTGTTGATTTCTGACAATCACTGATGATATCATCTAGGGGCTAAATCAAACTTTCTTTAAGTATAAGCAAGCATGGGGCGGCCTATTATTACTTAAAGG<br>AAATTAGCATATCTGCTAAGAATCGGAACTGGCGATGATGTGTCCGTGAGCAGATGAAACTGGCCAGCATACTGACTCTAAGCGACTTTGAGTAAACGATGAAACAT
Gateways-1	3:**GCGGCTTC**CTATTCAAACATTTACACACAAACACACACACACAACCTTCTCCATTTATGTCTATGGCTGCATATGCTTCAATTTAATTTTACCCTCATATTAAGCCAGTCTGTGAGTCAGAGCTGGTTTAGTTTGCAGCTGT<br>AAGTGTAAAATGAGCATATCGTTTTACACTTTCACGCATTAAGACTGAATTTCGCACAGTAAATGGATGCTAATCAAACGTCTTTCTTGTGAAATATGGCTCATTTCCTCCACACGAGCCAGACTGTGGCAACTGTTGGCACGT<br>TGAGAAAACACCTCCTTAAAATCTCCAGCACGTGATGCTTGGAAGCAAAAACAAAAGACGGCGCACAGCTGGCAAAACAGCGTAACTGCACCGGCAGCCAAAAGCAACAGCAAATCCAACAACGGCCCAATCTAGTAGCAGACT<br>CCTGCCGAGCGGAGCAAATGGAGAAGATCCCGGATTTCAAGAAGAGCGGCTGCTAATGCTCACCAGAGAATTGCACAAAATATGAAAAGGACGCTAGTTAAGTTGCGTGGTTATAAGTCACATATTTAGTATGAGCTTTAATTA<br>ATTGGCATCCCGTCTGGTCCACATTGAGGCTCATTC
Gateways-2A	5:**GAAAAACG**CCACGCTGTCTCTGCGTCTGGCACCTGAGGTGTGTTTTTGACCCGTTTATTTGTTTGTAGTCATGTTTGAAGTGCAGCAGTCGCGTCCCTTTTGTCTAAGCGTCCACTTCCTGTGCTGGAAACGGAGGATTTTA<br>CAGTTTTCCCTCAATTGCTTTGGCACATTTTTCGAAACAGCCTTAAAATTCTTTAATTGTGAGTGCAAATGTTAGATCGGTTTGCTGGAACGTCTTTATTGCGTGTCTGCAAAATGAAGTAACTCACCCAAAACATTTGATCCC<br>TGCTTCAAAACCTATTTATCGGGTCAGAAATTTGGCCTAAAATTTGTATTTTTCATCATGTGTGCCTCGCTGGTCAAAATGATTAGCTGGTTTAACAAATCTGATATTTGTTGACCCTACAAAAAAAATCCAGCTTAAACCAGC<br>CTAGGCTGGTTTAAGCTGGATTTTTCAGCAGGGGAGAATACATAAAACAGTGGTGTCCAAACTCGGTCTTGAAGAGCTGGTGTCCTGCAAATATTAGCCTCAAATTGCCTCAACACACCTGCAAGGATGTTTTTAGTGAGCCTT<br>GATTAGCTAGTCCAGGTTTCTCAATTCCAAGAACGCA
Gateways-2B-1	5:**TATATATA**TATATATATAGTGTGTGTGTGTGTGTGTGTGTGTGTGTGTGT
Gateways-2B-2	5:**TATATATA**TATATATTTGCTTGTAATTTATGATGTCTTATTTTATGCACGGCATAAAAAAAGGTCCCCCCAAGTCAATCTAAATAAATGAAATATTTCCAAATAGAGCTATTCAAATGCCCTGGCAAAATTATGTTCATATC<br>ACAATCAATCATATTCAATCACTTCTGCAAACTAAATTAAATGTGGACACCAAAATGTAGTGTCTCAAAAAATAATCAATAATGTTCTTCTTTCACTAATCTTTGCTTGAATGACAAGTTATTTTTAGTGTTATTTTCTACTAA<br>AGATTGTATCTCCAAACTGTTTCCATAGTATCGCAGGGGTCTGAAT
Gateways-3	5:**GAGAGAGG**TGTATGTTGTATGTGAGAGAGAGAGAGAGAGAGAGAGAGAGAGAGAGAGAGTTTTGGCACG<br>3:**CCTCTCTC**TCTCTCTCTCTCTCTCTCTCACATACAACATACACCTCTCTCTCTCTCTCTCTCTCTCTCTCTC
Gateways-4A	5:**CTGATAAA**ATACAAGCCTTGTATTAGCCTTTTAGATGTAATTCATTCATTTTCTTGTCAGCTTAGTTTCTTATCCATGTGTCTCCACATGGTCACCACAGCGGAATGAACTGGCAACTTATCCAGCAAGTTTTTACGCAGCG<br>GATGCCCTTCCAGCCACAGCCCATCTCTGGGAAGCATCCACACACACTTTCACACACTCACTCTTACACTACGGACAATTTAGCCTACCCAATTCACCTGTACCACATGTCTTTGGACTGTGGGGAAAACCGGAGCACCCAGAG<br>GAAACCCACGCGAAGGCAGGGGCAACATGCAAACTCCACACAG<br>3:**TTTATCAG**TCTTTTTAATTTAAAAAATGGCATTATTGGAGCATTGGtGTTGCCGCCTTTTTGAATATATATTTTGCTCTTTTTTCTGTTTGCCTGAGCCCCCAGCTAAAATAACGTGCATGCTTTTGTACGTTTTTTTCATA<br>GGATTATGGATGTAAAATAAAATCTTACGTTGTAACTTCAAATAAACAGCTAATATCTTAATAATAAGCGGGTAATACACCAATAGTTAACAGCATGAAATGTGACCTACACAGTAAAAAATGCATGTTTCCACACAATTCATT<br>CATGTTGTCCCA

Gateways-4B	5:**TCAGCCGC**TGACCATCACCTGGAGATGACAATATACTGAGATGAAGAACAGCACACTTTATAGCAATCAATGCTCACTTCACTAACAACACCGCACTCAAACAGACCACATGATGAGGGACACCCACAAACAACACCACACTG AAACAGACCACATGATGAGGGACACCACAAACTGAGTCTCAGTCATCTCACTGCATTCACAGCATATATAAATAAAATGATAAACATTAATGCAGTTTGTCTGTCACGATAATTTCTGCTTTAGACAGCTATTAACTTATTATT TGATTTATTTATTTGCTTAGTTAATAGTTAGTTAGTATTGAAACGTTACCAGCACGCCCGGCCCCAATCAATTTGGCGCCCTAGGCAAGATTTCAGGTGGCGCCCCTTCACATCGCAGTAAATTCCACTGCTAGTGTACATGCA GTCATAAGAAACTAAATAGATTTTTGATCAACTACTGCCTGCTGGGAAAACATCAAATAAATATGTCAATGCAATTAACATTATTGCTGAACAAATCTAATATTTACACATTTGCTTGAGGAAGAAATGATGGGGACAGTTAAT GCAGTTTAAGAATTATCCGCACTCTTCCGGTTAACGGAAGCTCTGCTTTCACTTTCGATTTCCTGACTCTGTT
Gateways-6	3:**AATTAAAT**TTTGTCGGTTACAATCTTTTTTAGATTCAGTGGGGTCCACTGTGAACATTAGGCCTATTAGGGAATCTAATGTTTAACATCAGTGCATCAATTGTTCATTCATTTCATGAATTATTTTTAATTGAGTATCTCTA CATTTTTATTATTTCGTATGCAGGCCTGTAGCCAGCCCAGTGAAAGAGGGGGTTCTTTTTTTCTCAAAAAGTAAACCTTTTTGCAATTATATGCATCATTTTCTATTTAACTATGAGATTTAAATAAATTTTAAACACTATTTA TAGCTAAATTAGCTTGTTGGTTGGTCATCATAACCACTTTTTGATGTACCAAAATTATTTACTTAAAATTTCAAATAAAGAAATATGAAAAAATACTTAATTTTAAAATACAGATTTGTTACATCATATATCAATAGAAAAAAT ATATATGAATTCATTTTAAATGAAAACAAGCCTATTGTCATTTATTAGGCAAAT
Gateways-7A	5:**CCCAGCGA**GAGCTGAGTTCAAGCCGTCAGAAGCGCCTCTGTGTACCTGCTCAGCCACCATGAGGAGCCTCATTAACAACATGCAAAGTGA<br>3:**TCGCTGGG**CTGCTGTCGCTGATGCTGTTTTATTGTATTTCATGGAAGGTTTGGCGGATAATAATTCGCAGTCATTTCTGACGTGCTCTCATTTCAGACACGCGCATGAAACACATGAAATGGGGGGATGGGGTCATGCGCGT GTGGGGCCCCTGTGCCTTGCTCATAACCAGATTTGAAATCTTGACACTTCATAAAAATAAGAATTATTATAAGTTTGTAAAATACGGTTCCCTAAAAAAAAGAAAAGCAATTGTTATATGTGTATCATGTAAGTACGTACAGTTG ATTTAATTATTAGAAAATTGTTTGTTTTCTTTTTTTCCCAAGTGATGTTTAACAGAGCAAGCAAGGACATTTTCACAGTATTTCATATAATATTTTATCTTATGGGGAAAGGCTAGTTTTTTTATTTTGGCTGGAATAAGCAGT TTGATTTATTTTTTACAGTTTGAATATTGAAGGTCAATATTATTATCTCCTTTAATATTTTTTTTTATCCGCTTGTTAAGTGTGACATCACGTGAAGCAGCTTCCAGGTCCAAGCGCACTATCAAACTGTATGGGGAGACTTAA CAAATGATAATAATAAATGTTTACAAAGCGATGTAATACTTTCACAAAACATGATCGATATATATCCATGCCTAATATCAAATGACCCAAATTATAACTGATTATTTTTTTATTAATTCTTAAAATATTGATGTTGTCT
Gateways-7B	5:**GCCATGAA**GGTCATTAGGTTCACTGAACTCACCACTGTTTACTGATTATAACTACAGAGACAAGGACACAGGAGCGCTTTAAAGCCACGATTTATCACCGGACTCTCATATGTCAGTTTATAAACAAACCGGCTGATCGACG TCACTTTAAAATGCTCCAATGTCACTGTATCTGTGGTTACACTCAGTAAACCGGTGAGTTCGGTGAACCGATGACCTTCACGGCCAATCACAGCCTCACTTTCTGTTGAGCATGTGAACACAATGGTAACTCAGCGGTGTTTAA GGACATGAACAGTAGTGCTCAAATGTTAGCTGCAAAAGGACGTTTTTAAAATTTCAG<br>3:**TTCATGGC**CAATCACAGTCATTTCTGTTGAGCACGTCAACACAATGGCAAATCAGTGGTGTTTAAGAATGCTCATTAGCGCTTAAATGGACATTTTTTAAATTACAGTATCGTTTGGTACTGAATTCCAGTCCACTAGTCCA CACAACATCAAGCTGTAACATCAATAGACATTGATATATTTGTTGTTTTTAGGGTTGCGTTGGAAAGTAACAAAATGCAGCATCTGTCCAACGTTGGACTGACATTGGGTTCTGACGTCAACCGAATTTTCATTTCCAAACAAA ATGTAACGTCCCACAAAGTTGGGGTACAACAGCAATCTGACGTCAATTGAACACTCAGTCCAATCTGGAGTCAAAATAAGTCCAAAAAACACAACTCTTATAAATGCTGGCTCATATTAACCCTTGTTGGGCCAAAATGAACT CAATCTTGAATTA
Gateways-7C-1	3:**ATCCTGGC**ATGCACAGAGCACCGCCAGCCAGATAGCGTAAAAATGAGTGAGAGAGACTGATTGCCCTCAGCCTTATATACGGGAAATCTTAATGATGTCACTTGCGTGATCCAATCCGGACAACCGTTCACAAACTCCACCT AAAACTCAAGTGCAAAAAACACCAAACCATACCAAAGACAAAAAAAAAGACAGACCCACGTAACATGCAGTGACAGAAACAAGCTTCCACAGATATTATTATATAATTAAT
Gateways-7C-2	5:**CATCCACA**GCACACCAGCCACAACGCTTTCTGGATCTCTTGATTGCGGAATGCGTAAATGACTGGGTTGATGACTGAGTTGTAGGTGGCGGGCACCAGGGTGGCGTACGTGTAGAGCGGTGGGTATGTATAATCGGCTATGA GCGAGTATACAGTAAACGGCATCCAGCAGGCAGCGAATGTGCCCAGAATTATCGCCAGCGTGGATACGCCTTTACGCGTCGTGACGTAATGAGGGCTTGCTGCTAAAAAGTGGTGCTGGAGGGCAATTTGGTGCGCATGCCGCA TGACGATTTTGCAAATCTGCACATAGAGTTGTAGCATGAGGCCGAAAAGTAAAAGGAAGGACACGGATAAAACGGCCACGTTGTTTTTCGTTAGGGGCCACACCACGCTGCAGTTTACCTCCTGCCCTAAGCAGTTAACACCGG TGACTGGGAGCAAACCCAAACACAAGGAAACGCCCCATAAAAGCACCAGCATGGTG<br>3:**TGTGGATG**TATCCCTGCCAGCGTGGCCCATCGGGCACGGACCCCCAGCGACGTCTGATGTAATGTGCCTAGGGAATTGCACAGGTCATGGGACAAGAAAGGGCATGTGCGTCACCCGTTTATGTCTTCCCCTAAAGCCCAAA GTATACTTTGGTTTTTACGCATATGCAAAAGTCTGCGTACACCTGAATCGTACGTTCGAATATCTGCTTTGAATTGTTTTTGCGCCGCTACACTTTGGCCACAAGGTGTCTACAGTAAACATATCGGGAAAAAGTG
Gateways-8A	5:**TTCTCTAC**TGATGATGATGATGATGATGATGATGATGGTGCTGTGTGTGTGTGCAGATCTACCAGCGGCTGGTGGAGGAAGCAGGAAACGGCTTGGAAGAAACACAGGGCCGCTGCACCAACTTCCTGCTGCAGTTCCAGAA ATACCTGATGAAGATTTGCCAGGCCAG<br>3:**GTAGAGAA**ACACTGATGTAACAGTTAAACAAAACACAGAATGGTGTGTGTGTGTGTGTGTGTGTGTGTGTGTGTGTGTGAGAGGGGGGACAGACCCACACACACACGTGAACTCTCCCCGTGCCCATCTGCCA
Gateways-8B*	5:**GCTTTGTG**CCTTAGCAGGGAAGAGCAAACGGGCGCAGTGCTTGCTGGGTAAATGAAGCATCTGAGAAAAAACGATTCTCTCAGATGGTTTATTATGTTGTCAGCTTCATCCTACACATACGCAAAGTCTCACAAGAGAGTTT TATCACTTTTTTTTTT<br>3:**CACAAAGC**ACATCTAGGCAGGGATAGGAATTCAGTCTCTGAATTGTATTTGTCTGCAATATCGCTCCAGCTTGGAGCCGTTGCTACACGAGCTCCAGACATGATGACTTGCAACGGTCATGAGGGTGTTAAAGAAGTTCAAG CCTGGCCTATGTCATTTTCTATTACATTAGTTATACTTACGTTTCACTATATATATATTTTTTTCAAAGCGGCTTTATTATTTATTTATTTATTTATttatttatttatttatttatttatttatttatttatttatttatttatt tatttatttatATTTTAACAAGAAAGGATGAAAAGGTAATCGGGCCTGACTCAGTCTTTCAGAAGGTTCCTGTTCTGCAAAACATAGAAACAAACAGACACCCTTAGCATTTCTTCCATCAACACATGAGAGCAGCA
Gateways-9A	5:**CTTTCACG**GTTGTTTTAAATGGAGAAACATTGATGTTCTTTAAAATAGGTCACAGTTCTTCATTCTTTTTGCAAACTGTGGGACTGTAACATTGGGGAGGAAGGCTGTGCAGCTCTGGCTTTAGCACTGACGTCAAACCCGT CACACCTCAAAGAGCTCAATCTGAACAGCAATAAACCAGGATGTTCAGGAATTAAGCAACTCTCTGCTCTACTGAAGGATCACAATTGTAAACTT<br>3:**CGTGAAAG**TAAGGTGATCATTCTTAGATCTCCTTAGACAAACATACTTCTAGATGTAGGACGACCTGGATAAAACCTTAAAATCTTCTTTTCCAAACATTATTTTATCTGATGGCACCTAAGAAGCTGGAATGAACTTTCAA GGGTGGAGACCCAGGTGGTTTGACAAGCCAGAGCTCTTCTTCTGTCTTGGGATTGAACTGTGGAAATGGCGTACTGACTCTGATATGGCTGTCTGACTCTGTCAGGACAGTGAGATAAAGAGAGTGCGGAATGGGACAAGATTT GATAGATGAATGCGATGTTGGCGGAATGTCCTTGTGTATGTGGTTACAGGAGAATACAAATATTTGTTGGACACCCCA
Gateways-9B	5:**CCCTCGCC**CAAACATCCACCGACATCCACCCACAGTTTGTACTTTGATTTGGACTTGAGGCTCCTGTCGCTTGATCGCAACGCTGTATGTCTCTTTATTATGTTTTCTAACAAGGTATACATGTTGTTCTGTAAAGTGTGGT ATTTCTAGGGCTCATTAATGTGGTGCAAGTTATGTTGTCAAATGATGATGGAATTTTTTTTTGTTTTATTGTATTTAAAGTACTTTAATGGACGTAATTAACCCTGTACTTTTATCCTATATTATATTGCTTAATATAACTGTA ATTCTTATTAA<br>3:**GGCGAGGG**CCGCATGTTATGTTGTGATTTGATTACCTTTCAGACAGGTGCAGTAATTTAATCATCCATTCCACAGTTTGATCTAGCAGGTGCATTGGTTAGATAGTTAGCCCATTATGGTATGGCAAGTCGTTTACAGAGTA TAATCGCATTAAAATAACAGTGAATCTTTTGGTTTGCATCAGTGGTTCGGAGCTTGAATCGAAATTACCAAGTCACGTGATTTCATTAAACGATGCTTTGCCGCTGGTGAACCTCTGATGAACCCGAACGATTTTCAG
Gateways-10A	3:**GCCCTTAG**CCCTATGCCTTCCAACTAAAAAGAATTGGCACACCCCTACCCCTTTACTTGCATGTGCAAAACATTGTTTGACTCCTACGATTCTTTAAAAATCGTTCATCGAGTGATGGTACCGGTTTGGATACGTCTGTACA AGCATCGTTACTCTACCCTCCAGTATATTCATTTTGCTACTGTTGTGGTTTTAGATGTTGTTCTCCTGTCTGACCTCCTGAATGAGAAACAAATTTGGTGGAAGATACATTGCATGTCAAAAAAGATGTTCCAGATGAGTTCCA GAACTGATTGGGTAATGGACATGGACCACAGGGAGCTCAATAGGAACTCAAAAAGAACTGTGTTTGGTCAGATTTTGTTTGAAATTACATCATTTCAGTTTGTTCTTTGCTTTAGTTTGAGGTATATCAGGGCCTTAACATTTT ACATTTTAGCATTTAGCAAACACCAGTCAACACACACAACTGGAGCAGTTGGGAGGGTTTGGTGCCTTGCTCAAGGGAATCATCTCTCAGACAGTGCTGGAACTCGAACCTGTGACCTTTTT

Gateways-10B	5:**TGCTTTCA**GGAGGCCATGATGGACCATTCTCTGCAAATGATTTCCTACATCGCCGACATTGGGAACATTTTAGTTTTGATGGCCCGCAGGAAGCCGGCCGGTCGTAAATCAGCAAACTCAACAGATAGCTCTGCGGCGCCAC<br>CGAAAAAGTGCTGGATGATCTGCCACGTCTTTTCTTCAAAGGACGTGAGTGATTTATTTATTCACTGACATTGAATTTATTTGTGTGTTATGTCCACCTGCATTAAAACTTGCTTCGCGGTGAAATAAGGATTATGTTGTTATA<br>ATTTCTGCATTTTTTGAGTAACAATCACACAGTGTGAATTATCAAAATCGCAATCTGGGAGAGTCACCATTGAGTCGCTGACACCCATGAGCTATTTGGCATGCTAAACATCTGGAGTTGTCTGTGATTCAAAATCATACTGTG<br>TGATGTGTGTTTAACCTCACATTAATTATTTTTTTATTTAATCCAAAAGTAATTGATCTGTTTGGTATTGTAATAATGTTATATTTGTAAAAAAAATACTGCATTTTTTAACTTGCAAGCATTAAAACGTCTTCAACTACAGCG<br>3:**TGAAAGCA**TGAGAGAAATCAGACACACACCAAGGCAGACACAATCTACAGAAGACGGACTGTCATCAAACCTGTGTGTCAGTACTCAGCACTTTGATTCTTTGTGTGGAGATGAACAGATCCACCTCTGTCATGGGCTGAGA<br>CTCTCCTTCAGGGGCCTGTATATTACACAAATGAGCATACAATACACAAAATAAGTTACGATTAGCAGGTGCTTCAGTTATGATAATACTGTAAATACTACAAGATACAGTAATATTACATGATAATAGTGGTAGTGGCCGCCA<br>GTGTAAAAGGCTTGACCACAATAAAATAATGCTGGTTCCTTAATGGTCCAAGGATACTTATATTCATCAGGGGTCGACACAACA
Gateways-11	5:**GTTAAATG**TTACATATAGCTGCCGCTTGGATGGCTGACAGCATCGTGAGGTTAAATGAAGGAAAATGTTTAGATTCAGTAGCAAACGCTGCTACCTGCAAACCACGTCCCACAGAATTCACAGTGAATGGCTTTAGTCATTT<br>TCATAGCGGAATTGAAGCCACTGGATATCTTTTATCCACTCTTGGAAAAGTGTAAATGGTGGATTAACATTCAGCACATGATCACTAATAAGATGTGCCGTTATTAGTAACTGTGGGTAAATCTGTCAGTGTTATTTTCATTCT<br>CTCATCTTCAGTGCTTCACATTTTGGCGGTCGTAGCTCCTGTCAATTGAAGCCAGAAGGCACTGACCGAGTGATTGACGGCTGATATTAGCCAATC<br>3:**CATTTAAC**ACAGCTCATGAAAAGACCGTTATTACTATTAAGACGACATGCAAATGATAAGTTATATATCAATATAAATGTGGGGTGGGGCGATGAATCGCGAAGCCGGCTCAGCAGTGTGAAATTTGTCGGCCATCAGCGAT<br>GGATGATGGCATCATCTATCAGCACAACCCTAGTATAAATATCTAATAATGTAAAATAAGTAAAAAAATGCATATGCATACATAAAACTACTTCTAAAATAGGGCTGTCAAAATTAATTGTTTCTTCGGTGCACCGCGATGCAG<br>ACGTGGACAATTCGGTATCGATTCAGTAATAACCATAACCGGTTATTATGTAGTGACGTCATTTATCTCCTATGCGCTCTGTCGCGAGGGAGGTGAGCGCCGTTATTTACAACACTCAGCCAACTCAGGGCCGGAGCGTGGCAT<br>AGGCACCATAGGCAAATGCTAAGGGCGCTGTATATCCAGTGGGGCGCCAGAAATGAGCGCGCTTCAGTTGGTTTTGTTTTCGATTTTTCTGACACACATTCAGTTATAGACGGCAATAACTCAAAAAACGCTTACCCTAAAAAG<br>ATCTAAAGTGCGTTTCCTACAGAAA
Gateways-12A	5:**AGTTCGAG**GCTAATACATCTAAAATAAAGCTACTTTGATGCATGGTGGAGCCAAAATATCAAACGGTGACTGTGATTCCACCTCATTAAACATCTGAAAGACACATTTCCATCACTTTTGTCAATGTGTGAACAGCTTAGCC<br>TAATAAAATAAATAAATAAACGTCAATAGGCTATCTATTAAAGCCTGCATATTATTTTTGTTGCGGTTTGATGCTGTTGAAAATTGCGTGTAGTACTTGTTCTGATTTTGTTGGTACTTAATACTGGTATCAGTATTTGCCTCA<br>ATTATTGAAAAGTAACCTCACTTATCTTTTCTGTCAGTCTTTTGACTGTTGATTTGGGCTATTTTATATACAATAACCATGTGTGAAGCAGTTCACTGTTCTAAAGTACTAATAACTGGATTTGTGCTGTGACGACTCTCTTTC<br>TTCAGCTCTTCTTGTT
Gateways-12D	5:**ACTTCCAT**ATTGGAAAAACGAATCGTATAGAAGTCAATGGTTACAGGGTTATCAAAATATTGTCTTTTTCTGTTCAGCAAAAGAAAGTCAAAGCAGATTTGTGAAAAGTAAACGTTGAGTAGATGA
Gateways-13	5:**ATAATAAG**TGGTGTCAACTAAAACCCTGCACTGCTAGTTATTGATTAATATAATCAGTGTTAAAAGATCAATTTGGTCCTCACTTATTTTTGCATGAAGAGCAATTGATGGCCTAAAGCTCTCTCTTGTTTGGTCTTTTTGT<br>TCCATTATCTCTTGTAACAAGCTGAAGTTGTCTCCAAAAAGTTCTGCCATGTCACACTTTATCT
Gateways-15	5:**TGTGAGGG**TAAATCTTACTCTACTGGCCTTAAAGAGATAGTTCAACCAAAAAATTAAAATCCCTGTTTTGGAACAAATTACTCGATTCACATGTGTTAAAGCACCCTCAGACAGCTTTGGAACAAA<br>3:**CCCTCACA**GCTCTATATTAAGTGATCTCTATGACACTCACACCTCTAAACCTCACTCCTATCTGGGTCACGGCACTAGCGTCACCCCTCTGGATCTACTAATATTACTTGAAGCTGGATTCAACTGGTGTAACCAGCACAGG<br>AAGTTGGGGGACCTAAAAATAAAAA
Gateways-16	5:**CCTCTCTC**ATGCCCCAGTGCACATTCAAGGTCATGTTAGATCATCTTTTTTTTTACTCTGTTCTTGGCTGGTATCCTATACTGTTATGAAAACTTTTTTGTTTACAAATCTGATTAAATAGCAATTATTCTCCAGTCTTCAT<br>TGTCACATGATCCCTCAAAAATTATTGTAATTATCAAAACATTGCTACTATTTGTTTTATTTCTTTTTTTCTAAATTTTTTACATTGATCTGACCTTTATGTGACATTTTGTTAATGCGGGGCATCCAGTAAGATTTGATAGCG<br>CTCCCCTTTTTCCCTTTTTTTATGTTACAACCC<br>3:**GAGAGAGG**TCAAACTGATCATGAGATTGCATTTGCTATATTGATTCTCATGTTTATTTTTCTTTGTAAGCCCTGCAAACAGCATGAGAGCTCTGCAAGATTACTCTAGACAATCTAAATTTAAACCTAAAGCTAAAAAAGTC<br>CCTATCTAACCTTAATGGAATTATTAATGCAATTCTCTCAGAGACTTTATCTCAGAAAGGCAAATCCATAATAAGTCTGTATCC
Gateways-17	5:**CTTTAATG**ATACCCAGAGGCGCGGCAGCGACTGGAGTCCGCGGTGTTTAGGTGTTCTGTATTGTTATTTTAGCTAATGGCATTTGTGTGATAAATGTACAGATTACTCACAGACACAATCAAACAGTCGAAATTTTTCTGTA<br>GAATTAAAAATCATATAAAATCTGGCTGGATATGTTGTGTTTTCCCTCAATAGATTCAACATTAAAT<br>3:**CATTAAAG**GCACTCATAAAGTCTACAATACATCATTTAAGCATTATTCACATAAGGAAGAACAATAAAAAGCAGATTATTCATGAAATGCGATAAAATCAGTGTCAGTTTGTATTCGGAGTGTTTATTATCGTGAAAGCGTC<br>TTCCCGTCTGGATTTCTGGATGCGTCGTGACGTCAGCGCGTCCTGACCTCTCGCGAGCGCAGGAAACTGGAGCAGCGACAAACTCTCGCTGTTAATTTTTGTTTTAACTCTTTTTTTACGCGTTAGTTATTGTTTTTTGAGCTC<br>AGACCGGCCGTCGAAAGACTTTATTCGCGGCTAGTATCGTTTAAAGGGCGCTTTGGGCTTTTAAAGTGGTCGATTTGTACTTTTATCCCGGTGTTTTTCCTCATGGATCAGATGGTGACGCGTCAGAATATTTGACACCAATAA<br>ACGCGTTTCCTTTATTCATATTTCCATAAAGCAACACGTCGCTAAGCGCCGCTTTTAACTCTTGATTTTAAACGCGAAACTCCTTTATTCCTGCGCTGAAGCGAGGATATTCGAGATTATTCCTACTTTAGCTGCTAATGCTAA<br>CTAGCGCAGGCCGCTGTTGGAGTTTATTTATATTTGAGTGTTTTTGTATTTATGTGGGTTATATGTGTGTCTGAGAGTATGAATATTCATGAGAGCACGATGGAAATCTGTTATTGATCAGTGATGTCTTCTCCAT
Gateways-18B	5:**GACATCCT**CAATAAACTACAGTACATTTAATATAAAATAATGATCCAGGTCTAAACGATACAGTTTGAAAAGGGTTTTAAAACCATAGAGCAGTCTCTGGCAGAGAAGTGAATAACTCATCACATTCTTACGAAGATATTTT<br>ATTCATTAAAGTTATTATTTTATTTACTTGAATACAGACCTACTTCGCCTGCCTGAATGTGAGTTTATTGCACGCAGCGCTGTATTCCACAGAACAAA<br>3:**AGGATGTC**TGATGACTAGCGCGCTTCTGCTGAGCCAGCTGTGGTAGCTTAGCAACCGAGTCGCGACGTCAACACACAGAATCTGTCAGCACAGTTTAGCACGGTTGTCTAACCGTGCCGAGAATTCCGGGCCGAGAACGGTT<br>TGTAATCGTGCCGCGCCGTTCCAGGCTCAGTGGAGAAACAACCGTAACCGTACCGAAGTGTTCTTAGAACGGTTTGGCACGATAGTGTAAAAGGAGCAGTAACAGCTGAGAGGCTAAACCAGAGACATGATTTGTGACGCAAAC<br>ATCAAATGTAAACAAGGCAAGAAGTCGTTCAAATGTCTTCACTCGTTTATTTTCCAATATAAAACATACCTTGATATTTTGACAGTGTAGAATTTGATTTGGGCTTAAGTTTGGTTTTGTTTGGTCAACAAAGAGTACCGCGTC<br>CCCGTTCACCCCCTCTCTCTCCTTCCTAAGTTGTGAATGAACAGGTGCCACCTATAAGCGCACTACGTAACCCCGGTGAC
Gateways-19A	5:**CATGTATA**CAATCGTAGATTAATTATAAAGCTCAAGCTATGAAGGAAGTTTTATTTATTTATAACGTTAACTTATATCGGGGAATTATTTTATGCTACGGAATGCAATTGCTTGACTGACTAAGCAAGCATTGAACATCCCA<br>ACGCAGTCAACTAACATCAAGTTAAGCTACCCGGTTAGTCTGCTAATAATTATTGGTCAAAGTAACACACATACTTCACAAACACCAGTTAAGTGGTGTTTACTTGCCTGATTCTGGAGCAATCTTTCTTTTTAAAAGTTCTTC<br>CTAATCTCAGGGAGGATTTTGAGGAAAGTTTTCAGCGTGTCCAACACATACAGCTTCAGCAACTCACCGCTAGATGTCGCCAACACGTGCAGTTTCACCAATTCACCACCAAATGTCACCAATACATGCAGTTTCACCAATTCA<br>CCACCAGAGGACCAACACATTCAGTTTCCAGTTCACCACCAGTTTACTG
Gateways-19B	5:**AACTCCAC**ACAGAACCACCAACTGATCCAAGGATCGAACCAGCAACCTTCTTGCTGTGAGGCAACAGCACTACCTCCCACACGAAAAGAACTATAAACTTATATGTTATGTGACATAAAATTGGCCGTGACATATATAAAAT<br>TGGCCGATTTCCTCTATG
Gateways-20	5:**TTAATAAA**TGACTTGCACCTCAAAAGCAGTAACGCGTGGTGGTGTTTAAAGCTGTGAGAAGCAGAACGCAAATGCTCTTGACAGTCTTGGGGTAATTAATAATATAATAACACTAATAATCAAAGGGTTAAGGCGTTTTAAA<br>ATGACCAAAACAACATTTCAAATGTTTTACAGTGCACTTAGCATGCTGGTTTGTCCATTTACACACATTTCTATCATCACATGATTTCTTATAACAAAATCACATGACCTTTTTATGCACATACTGGAATTTGTTCGGTAAAAG<br>TGTTTCCATCATAGTTTATGCGCATATTTTCTTATCAAATAAAAAGTTCATCCTACTCATTTTATGCACACAAGTTTTTATGCGCATTTTTAAAAGTTATGTGCATCTTGGCTG

	3:**TTTATTAA**ATAAAGATTGACGCAGCTTCTCCTACCGCAGTAAATTCAGTTTTTACTGTTGATATTTGGCACCAGGAAGTGATGATTTTGTTCTCTTTGACTTGTTGGATGGAAACGCTGCTTTATTCGCGCATCTTTATTCGCGCAATATTTTTAGCTGCAATATGTGACTGTAAGGTCTTACAAATTATATGGTTCTGCGATATGTCAGCCTTCCTGCATCCAAAGTAATCCAGCACAAACAACTGAA
Gateways-21A	5:**AGGAAAAC**ACAGCCAGTGTTTCTGTTTTCACGCTGCTTGTATGACTGCGCGGGTCGCTCGTCTGTTCGTGCCTCGGCGGGGTGTGTGAAGCCCAGGAGCGGTTCTCGAGCTCGCCGGCTCCCCTTTCAGACCATCGGCCTCTCGAGGTTTCTTTGTGCTGCCCCAGTGATCCTCTGGCGGACTCGGAGGACATGGCGGAGACCGACGGAGCCGCCGCCTTCACGGAGCTGCGCGACCCGGACTGGGATGAGTCGCAGCTTCGACAGTATACTTTTCCAACCCGACAAATACCGCGACTGTCCCATACGGATCCGCGTGCCGAGGGGCTCATCAATAACGAGGTAAGCTAGCATATTTATCCTGCATTATTAACGCTGCAAGAGTGAGCGACCGACGGAGGAGAGGAGAACAGCAAACGTCTGTTTGTGGGTGCAAGGTGCTTTAAAATTAAAAAATGATTTACCCAAACTCAAGCAGCAGGACAGTATTAAACGCTTTGTTTGGATACAAAATGCTGAAATTAATGCCAAAACTATTTGATGAGTTGGGTCATTACGTCAGATAAAAAA<br>3:**GTTTTCCT**ACTCATAACACCCACCGGAAGTAGGCGTTTCAAAATAAAAGTCATTCTATAGTTTCTTGCTGCCTGGTGTTATTCGTTATTCGTGAATGAATGGCTGCTGAGCAATAAGGAAAGAATTACAAACATGTCAATCGTTCCAAAAAATGCAGCACAAACAACTGAA
Gateways-21D	5:**TTTCCCAC**ACTTTTACCAGAAGAGGTCCTCATCGAGCTGATTTAGAAAGCTTCGAGTAACGAACCTTTTTCCGATACAATTGAGTCGAGCGCTTCACTGGTTCAGAAAGCTTCATTTCACCATCACTACTGCGGGGAGGTACTGTGATTCAAAGCGACACCCCCTGGCCTAAAAAATCGCTAGCGCGTCACAACAGCACAAACACCTATC<br>3:**GTGGGAAA**GCACTGTGTTGCCATAATGTCAAATGTTCACAACTGACCAACTCATTCATGTAGTAATAAAAAAACAGCAATATAAACAAATTACTCAATTACTGTAGTTTTTACACAAGATATATTACATTACACAACTGATGATTGTAGTAAAATCCAAGGTATTCAAAAGTTTATCATAATACAATTAGTCCCGCTCCAAGCAGGGATCGAACCAGCGGTTTCTGGTTGGGAGGCGAGTGCTCTAGGAGGACGCTATGTGAACGATGCTTCAGGGTTGCATTCACCAAGGGCGTATTCCAGAAAGCAGGTTATGTGACATACCCGGGTAAGTTTGAGAGTAAGTAAGTGGATAACCTCAGCTTTCGGTTTCAAAAACGAAGGTAACTTTGTGGGTATGTATGTAACCATAGCAACTCACTCTCTGAAGATAACCTGCTCGGTAGCAGGTTATGTTCCAGTGTAAGTTTGTGT
Gateways-23-1	5:**AAAACTAC**AATGTTTTATGTCATTGAACATAGGTCATTTTTATATGCGTTTAAATTCGATTTATTATGACAATATGGATGAACATACAGTTCCAATAACTGGTTTGTCAGGAAGTTTTGGTCTACGTTTAACGTTAGCGGAAGTAGCGTCAGACATTTAGTCGTCCTTACGTCGGATTTTACTCCCAGCACCAACACCTGAA
Gateways-23-2	3:**CTCCGTTT**ACTGTTTTTAGAAACGCGTCAGTAAAATGTGCTGAAATTCAGTGAAAACAAAACACCTGGAGATAAGACACATATCTTTAGAAAGCTTGAAGTGTCTACTTTTAAATGCAGCGAGTGCATGTTGAAAACAAAAATTTTGATTGACAAAGTAATCAGTATGAAACCAACGCTATGTCTGGTTTCTCAGTCTGAGCTCATAAGAGTTAATGCTACCACGCCCACAAGGTTGTGATATTACAAATAGCCTGCATGAGATGCAGGCAAGATGTAGACTGTGTAAGGAAAAACTTGTCATTAAATTAACTGAAACTAAAATGAGCAAACACTCAACAAAGAAATATGGGAGATGTTTCTAAAGAAAGCTTGATATGTCTACTTTTGCATTAACCAGTTCAAAGTCGAAAACAAATAATCTCTTTTTGTGTAATCTGTATAAAAGTAAGGACAGATTCTCCTGTCGCCTCATTAGAGCGAACGT
Gateways-24	5:**GATAGCAG**CTTATGTAATGCACGAACACATGACAGAAGAGTGGGGAAACAGATTAAAGTCTGAATTTATTGACAGCTGAAATCCTCCTTCCTCACATCATCAATAACCAATAATCAAAACACAATAATCAATAATCAGAGGAGACCTGCAGATGCAGACTCACAGCACAG
Gateways-25	5:**GAAAGAAC**GAAACGAAAGAAGAAAACCCACAGCAAAGCAGAGAATGATTTGTGTATGTTGTTGTGTAGAGACATGAAAAACAGTGACACACGTGTACCCACACACAATAGAGCTTGAAAAAAGGCTTATTATACCAAAATGAAAATTAAACTTAAATGTGCTTTTGAAAAAAAA
Gateways-26	5:**TTTTTTAG**GGATTACAAGTGTTTCTTGCAAAGTCTTATCCCTATTCTTGAAGGCAACATATTGTGTGCTTAATAAAATGCATTAATGTTATTGGGAGCTCATCCCTTGACTTATTTTTTAGTTTGAACCACAAACACAAACGTTTCATATGTCCAACCAAATGTTTTAAACATTGGCTGCTGAAAAAGGGTGAAATGCACCTTTCAAATGCTAAACAGCCAAACGTAAGAAAGTCGATTAAGGCCTTAAATGACACAATTCGGGCTCTTTTTCCTTGCAGCTATCACAACCTCAATGGGTACACATTAAAACCAACGGGATTCAGTAATTGTGGTTTACGGCAGCTGCCACAAGAGATTAAAAACACCCAGAATGGTTTCAAATGGCGTTTTGG
Gateways-27	5:**CCAAAGAC**ATGCAGTATAGGTGAATAATAGGTAACTACATTGGCAGTAGAGTATTAGTGTGTGTGTAAATGGGTGTCAGTCACTTTGTGTCACTGACTCGGTCCCAGCCATTCCCCTCGCTGGCCAGCAGAGGTCATCATCACCGGACTACTGACATTATGTCACCCTCACAGACACTGATTGCCACACACCTGCTCCACATCACAAGCAGCACAAACA
Gateways-29	5:**GATACTAG**TATCCATTGATAAGTACTTGGTATACAATAAGTACTAAATAACCAATAAATTTCTTTGAGTACATAAAAAGTATTTATTAGTCAGAGTTGAAATACTTACTAGCCAAAAACAGAAGCCCAAATCTCATATTTGGTACTAATAACTAATGAAATAAATTCTAGAATCTAATGAATATTTATTAGGACCCATTTAATAGGTACAAGTATCTACCAAATAAGTATCCAATGAATGAGTACTAATATCTAAAGCAGTGGTCTCAAATCAATTCCTGGAGGGCCACAGCTCTGCATAGTTTAGCCCCAACCA
Gateways-30	5:**ATCCCTGA**ACAACATGAAAATTATATTAAACACACAATGATTATTTAGCAGTTTGTATTTATTCCCGAATTAAAAGCCTACTGTTTTATGTCAAATTTGTTTTAATAACTGTGCAATTTATTTATTTATTTTTTTTGCCACAGGTATGGCCGGCGGTTTGCCACACTGCTTTCCTTGTTTCTGCAGTTTCTGTCTGGAGTGGGAGTTGCATTTTCCCCTAACATCTATGTCTACATTGCCCTCCGTTTTGTTGTTGGCACCACAATATCCGGAATCTCTATCAACA<br>3:**TCAGGGAT**TGTATTCTACACGTACTTGTCTGCCATTGGCCCAAACAATAGGGCTCCAACAAGAAGACCAGCCATGTAGATGGACTGAGAGGCCTCACTATAAACCTTATTTTCACACACC
Gateways-32*,**	5:TATATATCATATAACACGCCCAAAGCTCATTATTATTTACAGTAACGTAACATCAATCGTATCAAATTATTATCCAAACCATTGTGACAGCA<br>3:**GTGTTTTC**CAGGTAGAGTATCTGAAAGGCTACTATGCTAGCAGTTATGGAAGGCTAATGAAGTTATTTTGCTCTGGAGGACTAACGTTAGCAGCTGTTGCTCAAAACATCGAGACAAAATAACCAGAATTACATTTAGTTTTTTGTTTAATAGCAAGTATAATTGCCGCTAGAACCAGATAAAATTGCATTGGGCATGTTTTGTTTATTTGTAACGTAGATTTAAAAGACTGTATATTTCATAACACTTTATAAGTACTTGAAACGACACATGCAATTGTACATAACATTTAGATTTTATTTAAAAATGCATTATACATGATATATATGGTTCATTTTCAGTCTTTtcgttcattGCTGTCAAATGATCCTTCAGGAATCgttatcaacaaaaaggaATTTTGTTGATAAATTTGATGTTTATGAAACTATTATCAATGTTTTTTTAAATGTGCTGCTAATTATTTTTCTGCAAACCAGGAATTTCAGGATTTGACCAAATTAAAGATACATTTATTTTGTTTTAaaacctcTTACAGATAGTTTTTCAGAATGTAATGCATTTTGCTAAATAATGTTTATTTCTTTAAAAAAAAATTCTTTTGAAAAAAGCTTTC
Gateways-33	5:**CCTTCCAA**CAATACCTACAGTGGATATTCAACTACACAAAGCAACAAGTCAGTGCAACCTGCTTTTAATTCACTAAGGGTGTGATCACTGAAAGACTGATGGGCCTGTTCACGCTGAATTGATTATTGCTCTTTCAGCTCCAAAACTGCCCTGTTTCCTCATTAGCCCAAGCCAATTGAATTGCGCTGATGCCCAAAACAAACACCTGAA
Gateways-34A	5:**CCTGGTAC**ACAGAATTGAGATGTGTGTAAATATAATGATTTAAAAGCATTTTTTTTTTTT<br>3:**GTACCAGG**GGCGGACTGGGACAAAAATTCAGCCCTGGTACTGTAGCCCCACCAGCCCACAGTACCACACCGATACAG
Gateways-35	5:**GGAACGGT**TCTCAGCCGGGAGGGAGTTTCGAAAGTGTCAAGCCCTATGGAGGAAGAAAAGCCACCGGGTAGGGGACAGACTGCTTCACAGAATGTGTCGGAGGCACCGCAAATTTATCCATGGATGCGAAAACTTCATATAAGTCATGGTAAACGGCAAACGTAGAGGTTTGCTTTTATTGCGTTTGTGTTCTGAGTGTTAATGTAAGGTGTGATTGGCCTCCTGCCAGTTTTACAGTAAAAATTTTTTTAACTTTATATGAATATTGAAATATTAGAGTTAGTTTTTCATTTTGGATATTTATATCGAAAAATATGCCAAATATGCATCCTGGGGTCTAATGACTCCATGTTTCTAAAGATTATTTAAAACTGATTATATTCTTTAAAAACGAAGTGCAATTGTATGATCAATTAATTTGTCGATTGTGGATTAATAAATTGGTGGTGTTATTAAATAAATTATAATTTGTTAGAG

Gateways-36	5:**CATTTATT**ACATAGTTTGGAACCCCAGCCAGTGAAGACAAATGGCATTAAGGTGCTCTATAAAAAAGGTCATGACAACACATGTTTTAAGTAACTTAATTTTAATGAGTTATTCAGGTCTCAACTAAATTCTTTAAGGTAATATGTTTAGTCAGTTTGATCGATGTACGTTGAGATGACTAGAAAAGTTTATTTGATTTGAATAAAAAATAAGGGCAGCAAGACGTTTTTTTTTTGCAAGGTTTATTTTAAAAATCAGAATTACTTTAATTCTTTCTTTCTTACTTTCAACCCCTTCGGGTATGTCCCTTTTTTCTTCGGGGATCCCCACAGCAGCC
Gateways-37A	5:**CATTGGTG**CAAGTTACGACTGACATGCTTTTTTTTT<br>3:**CACCAATG**CAGCGCTTATACTCGGGTAATGATCACAGCTCAGGGACAGTATAAAATATAAACCGTTTTAAAGcATCATCATGCTAATGTACAGGAAAACTACCAAGTCAATGATGTAAAAGTGCCACTATTCAGCTTATTCAAAAACTATGTCATCAAACTACACCAATAATAAGCTGAATACACTGAATTAAAAGTGTCTaTTATGAACATTTGTATATGTTTACA
Gateways-37B*	5:**GAAATGAA**ACTGTCTGAACCTGCAAATGAGCTGAACTTCATGTCTAAACTAGTTTCACTTCACATTTCACACTTTTATAGTAAAATTCCTGAAGTTCTGTGGTTACCTGTCCCATAACTCTCAGCCATATCAAGCTCAGTTACCCAGAAAGCTGCCTAGAAACTCAAATTTCCAAGTACCATAGCAGTTGCCATCCACAACAGCTTTTTTTTTTT<br>3:**TTCATTTC**TCAGCACTGACGGCCACATTGATTACTTACTTTCAAGTTAAtaaacGCTGACTTTTTAAGAGGCGACTTCATTTGAGTGAGTGTGTTTAAAGTTTACTGCTGCTCGGTGGATTTTCTGAAACAGATATTGATTCTCTTTCTTATGTTGCAGTTGCATGTTCATGCATGTCATTTAACCCAGAGACAAGGAGGATCTCCGTAGGGATGGAAAATGGCACTGTGTCAGTATGTCAAATTATTATTATTTCCAATCACAAAGTGCAGCTGCTGCGGTTCTCTTACACCTACTAAGGTTCATTAAGACAGGAGCTTCTCTTTGCAGGAGTTTGTTCTGTCAGAAGACTACAACCAAATGACTCCTGCTCGGACATATC
Gateways-38A	5:**GTTGATGA**CCTGTTGATATGTGCTTAAATGAAAGATGTGTGTGAAAGACACTGTGACTCTCCTTAAGCATTTGGCTAAGGAGGGCCACAAGGTTAGTTTGACAAAATTGCAGTTTGGTAAACAAAAGGTAACTTTTTTGGGGCACGTCATTACACCACACAGCAAATCTCTGTCTGAAAAAAGGGTGAGTGGTATAAAAAATGTACCAAAACCACAAAAAAACAAAAAAAAACAAATGTTGTCTTTTTTGGGTATGTGCTCATATTGTCCCACATTTATTCCAAATTATGCAATTTTGGAACAACCCCTGAAAGCCCTAACACTAGGGAAGGGGATGAAATCCACTGACAAACTAGGGGGGACAACAAAGGCAAAGCAGGCATTTGTAAACATGAAATTACAAATGGCTGAACCCCCTGCATTGGGTTTACCTGTACCAACAAAACCATTTGTTCAAATGGTAAATCAAAGGAGTGGGTTCATGACGTCTTTGCTCCTACAGAACCATGAAGGTAGACTGTTAAACAATGTACTGGTTTGAATGCA<br>3:**TCATCAAC**ATACTGTAACAAAGCAGTTCCAGCTGTCAGGGTCAAAGGTTCCAAACCTCTTAACAACGCTTCATTTTATAAAGTGAAACAGAACCTGACACAGACATGTAAAGGTGTTGCCTTTGCCATTGAAATTAAATGCAAACCAAAACTGGCTGTCTTTGTCCACTGGCAGACTGAAAAATGCATTAGCCAGATCAACCACTGAATAGAATTGTGATTTTTCAGGAATTTGTGA
Gateways-39A	5:**ACAGTATT**TAGTTACTTTGTAACTATTAACACCCAAAACCGACTGAAAGCTAGCATGACCATATGTCCCTGTTTTTTTCTGAGGTAAAATAGTTTTTATATTCGCACATTCAAACTTTGGTGTTAGCGTAATAGTAAAGCAGCATTTAAAAAAAACTAATTCAAACTATTATTATAATGATGTGCTCTTATGAAACAACTTGTGACATGGTCGCTCTTTTGTTTACTACGCCACTTTGTATATTCAGTCTGAAATGAATGTAAGTATGACTTCAAAACAAAATTAACCTTATTTATTAAAATGTAAGCAATACTGTACTTTGACAATCATTCTTTGCTAATACACTGTAATAAGCAAGTCAAAAAGTAAAGACAGCAAATGTTTACATGTTGCCTTTAACCTATTTTTAAAAGCTAATCAGGTTTCAACTCATTTTTTTAAGCTATGAAAAGTTTAACTTTTTTAAGTCTGTTTGATTATTGCAAGTTGATGACAAGAAAAGTTCATTTGATTCAACTCTAATATTTAAA<br>3:**AATACTGT**AATTAAATTGTTTTTCTGCTAAAATATTAAAGTAAAGGATTACTTTTAATTTTCAGCCAATTTAGTTAGAGTAATTTATAATGTACTTGCCGTAAATACTGTACATTTATCAGTTCTGTAAAAATCAATTCAGAGAGGCAGAATAATTTCTGTTGTTTTAACACTGCTTACAATAAATTATTTCTAAACACAAACAAGTGTAAAAGGACAGCCAAACACCTGA
Gateways-40	5:**TTCATATG**AAACTGACAGTAACGAGACTCACTGATGTCATCCGGACTTTGATTGGCTTCATCCATTGACATGTACACCATTTTCTCTCGTATGGGCAGTCCAGAAAAATCCACCAGCTCCACGGTTTCATCCACATCTAGCTCAGCGTCTCCTATGTCGACTGTGCCTCCTGTGAATCACCAATAACTCTGTTAAAAACTATACTAGTAAACAACTAGAAATGATTTTATAAAAGGATATTGCAAACAATTTTTTAAATGCAAATGCAAATTGGATTTTCAAATGCATTTTCAATATGTGGATGGATAAACTGTAACATAATCCAAATGCAGTTGCAAATCTCGCATTGCACTTTCGTGACTGTGTTGTGACTGCCCCATTTCAAATAAAAAAATCCCAAACGTCCATTGTCTTACAATCTTACAAGTTATGCACCTGACATATCTAAAAAACAATATTAATGGCTGCATTTGCTTATTCTTTTTCCCTGCATCGTGCATGTAACCTGCCAAAGTGGAATCGCAAATACTTTTGTCATTTGAATTTCCGA<br>3:**CATATGAA**GGGGATCTGTAAGGGGTAGTTCACCCAAAAATGAAAAGCGGTTGACTTGCTTGCTTCGGTTGACAACAAAGCCAGAAT
Gateways-41A	5:**CATATATA**GTAAATCTGCTGGTTTTAAACATACAGCATAATAAAAAATACAGGTATGTAACAACCATAACATTACTCTTATGTCTTTGGACAAATATGATTAACTTTTGATTACTTACACTAAATGTTGACTATTGAAGGTACATTTATAACAACTATTTGTAGACTACTGTATTATCAAACGATGTTGCATACAAATGCATTCAACAGACTTAAATGACAGTGTATTGCTGTAGAGGACAAATAAAACACGGGACATGTGTTTAGGAAGAGTATAGTTTATTTTGTTTGACCTGCAAACTTATCTAAACAAGACAAACTCTCAGAAAACAGACCGGAAACATTATGATGCGCGCGCTCGTGGAGATAACGCGCGGTCGTCTTCAACTGTTGTCTTCAATAGTCCATCTTTGCTCAACAACTCATCTGAAAAACAAAGCAAAACATTACAACATTCAGACAAAAAGCAGAGAGCATAGAATCACCAAGAGGCGATACTCTAGTGCAATTTGGGAAAACAATAACGCGCTTTTTGAATACATTAAATAAAAAAAAAAGCCGCTGTT<br>3:**TATATATG**TAACATGTCATGAACTTACAGTTATTCAAAAAATTGCTTAAAATATCTTAAGACATGAATGGGTGTTGTCTTTGGTTTTACGTAAACCTACTGTAAGTTTTAGGGTGAATACAGCAGTTATAGGCAACCTATATCATAATATTGGTAGTCGGAGTTTTTTAATGACCCAAATAATTAAAACGTATCTTCCTCAA
Gateways-41B	5:**TTTGTGGA**CCACTTTCAAAGAATTTGTCATCGTCACCAGTTTGGCTGGTGAGCAGCCAGCTTGACCAAAATTAAAATGAGGACAAACCAGTCTAACCAAGAATAGAGAAATAATAGGGATGTCCCAATTGGGTTTTTGTGCCTGAGTCATTTGATTTTGAGTATCTACCGATAGTGAAACCCAATCCGATACTTCTATAATACATTTTTAAAAAAGAA<br>3:**TCCACAAA**GAAGGTTTTGCTTGAAGACACCACCAAACAAGCATCCGAAAGTCTTTTTAAACAGGGATGGTGTCAGCCTCATTCAAATTCTTCTTTGGCCTCTCACAGTTCAATGCTCTGAGCTCAAATCTGTGGTCATTACTCTCAGCACCCCCACCAACATCCATTTGTGCGCCCGTGTCTCAGTTTGGTGCCATCCAGTTGGAACTGAATGAAGACACAGGTGTGGAAACCGCCGAAGGTGTCAGGAATTGTGTGTGCGTGTATTATTGATTTGTGGCGATGTATGCTTA
Gateways-42A	3:**AGTGTGTG**TTTATGGGTCTGGAATTTTTTCCTGATTAGCTTGGCTGCCGCCTGTATGGTTTTAAATGCCATTCAAATGTATGCAGGTCTACTTTATTACTCCTTTATTCAACTCACGCCAACAAAACCACTAGGGGTAAAGACTCTTGCTGTGTCGCCGTGGTCTTGAGCAGCTGAGAAATTTGTTAAAGAGGGAAAAAGAGACTCAGTGTGCTTCCAACGCAAATACATGTCATAATGTTGATAAATGATTTCAGAAGATAAAAAGAACTCATTGCAAAGTGGAAAAAGTTGAAGGGATGAGCACTCCCAAAAGTCTGGCTTCTAACATCTTATAACAAAAAAAAACTTTTAAGATCTGCCATGTTAAATTTTTGACCAAAAAGAAGACAAACAAACCCAGCAGG
Gateways-42B-1	3:**CGCAGAGG**TGTAAAGTACTTGAGTAATTTTACTTGATTACTGTACTTAAGTATTATTTTTGGGGATTTTTACTTTACTTGAGTACAATTAAAAATCAATACTTTTACTTTTACTTAATTACATTTTTTTAGAAAAAAAAGTACTTTTTACTCCTTACAATTTTATTTACAGTCAAAAAGTACTTATTTTTTGGAGATCACTTCATTCTATTTTCCCTTGCTATTACCAAACCAATTGAATTGCGCTGATGCCCAGTTTAATTTAAATGTTATTTATTCTGCCTATGAAAATCGTTTTCACATTATATGAAATTGGTCAGACATGTTCATTGGTCCTTTGGAAGTGACGTCATGTCACATCTATTACCACAATGCACA
Gateways-42B-2	5:**CCCATTAG**TTCTTTCAGTCTCTGTTAATGTGCTGATAATCTGAATCAGGTTTGTTTGGTTAAGGAGAAATGGAAAATGTGCAAAGCTGTTGGTCCTCCAGGAATGTGGTTGAGAAACACCGATCTAAAGAACATTAGCATAAATCACTAGAATACATCACTATCTCATTGATTTTGTAAAAACAGTGATGCCATGTCCATATATATTACTCGTCCAGTCTCTAGGAATGCATAAGCACTTCACAACATTGCAAAACTACAGGACTATGCATTATGCCACTTTTGCTTATCGGTGTGAATGGGAATCGCCTTTACAAAGTTGCCACCTGAAATAATCATAGAAATGTTCTCAGTATGCTGTTGTTGTGTAAACATACCCTCAAACACAGTTTATCCTCACATTCTACCACCATCTCTACTCCTCATTTAACAGAGAAAAAAAAAAAAAAA<br>3:**CTAATGGG**TGTGACAGACAAAGGAGACATCAAAAACACAGTGTTGGTGGCTTCCGGGACCGTGGTTGAGAAACACGGATGTAGATGATGGTGGAAAATTTTTCAAAAATATGCAATTTGAAACCTGTTAGCATTGTCAGGGATTGGTTAAAAAACACACACACACACACACACACACACACACA

Gateways-43-1	5:**CTGACATT**TGAATCTTTGATGCGGCATCCATAGTGAATAACGTAATTATGGGGCGAAAAGAGCAAGAGAGACGAAGAGAAAGCAGGCAGCAGCGTCATTGTCGCAAAATATTGAACATATGTTCAAAAAGACTGGCAAACAG<br>GGGCTGGCTGACGTTACTTCCTAACAGATCTGTCCCGTTCAGGTTCTAGGCTGGCCGCATTAGCCGGGGCGTCCTGTATATTTGCCGTTATTGATATGTCTCGTACATGACCTCACTTTCCCTATTAATTTTTTGACTGCTGTG<br>CCATTACAATTTTGGAAAATAACTGTCCGATAAAGGCTTTACTACCAAGCGAAGTCCTCCGCTGTCAACTGTCACAGCAAAAAGCGCTGATCCCGTCCCGTCCATGTTATGAGGCTGTTTACACCTGCTCACTTCATTCGCTTT<br>ATTCTGATCTGATATTAATCCAATCGCTCAAACCACTTCAGATTGCGGTCTGGGACACCCTCCAAACAACACTGGACAGGTCTAATTAATG
Gateways-43-2	3:**GCTTCTTT**TAATTAGACATAAAAACTTAGATGTAATTTAACACAAGAATGTGAAGAGTGTGCACGACCCCCACAGTTCCTCTTTTCCCGCCGCATAGACTTTAGGATTTCTGAACTTGTTTTAATTAATCTTTGCTACTTTT<br>AAACGGTGTTCATCCTCACAGACAGCCTTCTAACTCAAAGTGACTCTCATAA
Gateways-44A	5:**CATCTATG**GTGCATTTGTCAAGCCTGGTCACCTTTATTTATATAGGGCTTTACTCAAGCAAATCTTACCAAAGCAGCCTAACAGAGGTAATACTAAGTTTCATCAATGTGCAGCAAGTTCAGTACATGCAGTTCCAAGCATT<br>TTATTCCATTATATATCTAATGCTGCTGTTTTATTGAACTTGTTTTTTTTACCTAGCAAACATGGTTAGAAGTTGTTAGTGATATTTCATTCTTATCCATACATGTCATAATGAACATAAAGCATGTCTGATTTCATGCAAATG<br>ATATAGTGATGTATTTTGTTGTTGCGTTACCATCATTACATAAGGCCAGTTGTAACTGACCTGTTATGGCCTTTATCTGACGTTGACAGTGTGCAGCATTGACTCTCTTTTGAGATCTGTCTGTCTCAAAGGCTGTCTCAAAGC<br>CAGTATAAATAATACACTAAGACAGCAAACTAGCTGCTGTGTCTTTGTCAGCATTGTAATGGAGGTAAAGGACAGCAAGTCCAAAGATACTGGGCATCAGCGCAATTCAATTGCAAAAA<br>3:**CATAGATG**CCATTACAGCAATCACATGAGGCCAATAGGACCTTGTAGCTCTTGTAGAAAGCGGTCTCATTCATAACAGCACGTTATCGGCCTATTGCTTTTGCCCTGAAAAGCTTTTACCTACACGGCTTCATTTCTAGGCT<br>CAATATTTCTATTGCAAACTGAAGGTTTGAATGAGGTATTTCATGATAGAATGAGGTATTTCAGATAGCGGTAAAGTTGGTTTTATATTTGGATATTCAGAAATTCTTCTTAATAATATAATTTCAGAAAAGTAATATTTGTTC<br>ATTTTAAATAGCGAAATCATATTAGCAGACAATGACTATCAAAAGTACAACATTGTTTACAGTGAATTAGATAGTGTTCATGTTGTTTAGAAGATGCCAATTCCGATCGAATATTCAAGGTAACATGGTAAACAATATAGAGAC<br>TGCTAAATGAACGAATGTACGAACATAAAACCAACTTCACCTCTATGAATTTCGGAAGTAACATTTAGCACATTTAGTCCTTATGTATTTATCACAGACTACTCCTACAATCGTCACCAGAATGCTTCTTTTTGAATT
Gateways-44C	5:**GATGCAGT**TGGATTTGCATTAGCAAATAAAGGCATAACCAAAGATGTTTAGAATCAACACACAGCTCTCTAAAACTTGGCATGAATCTGTCAAATTAAGTGGTGTAGTATTTGAGTTACATGACTGATGTTAAAATGAAGCC<br>ACCATGTGCATCAATTCTCTGGATTGACCGGCTGTCAACTTTCTGAACTGGCTCACACCCAAAGCTCTACAGCTGCCCTACATTTCTATGTGCAACACCAATAATATATAATTTAAAATTCCTGGATGTGAGCCAAGTGTGATT<br>TTCCATTCAAAGTCAAGGAAAAGCAAAAAGTCCCTTTTTTAACCTACCTTCTGTAACAACCTACTGTATACTAAAACACCTCAAATCAAACAGATCCTCAATTTCTGTTTCATAAGTAAACACAAATCAGTTCAGTTTCAAATG<br>AATTTATGCCCTTCTTCAAGCTAAAAAAAAATCTGCAAATG<br>3:**ACTGCATC**TAATTACGAGCAGGAAATCATGATATGAAAAACTGCTTTTGTTTAAAACAACAATTTCTTTGAAATGATAAGAATTGGATTTTGAGTTTAGCAGGGTCCAACCCTATTGCTCATTTTCTTGATG
Gateways-44D*	5:**ACACTGAC**ATTACCTGAGTCACATGAGGGGtGGcgggggattGGGAATAGATTGGGGCCACATTTGCCTGCTGTGTGAATGTAGGCTAAAAGGCACTATAGTATTATTTGGGAAGATAAACTTCACTAACTAAAAGTTAACT<br>GACTTTAAATGCTATGTATTGGCTGAGTTTGAACATTTATATTTATATAATCAAGCACTATTTTGGAAAAATGGGTTTGGTTAAACTGCAGTTTCACAGTGCCTATTGCAAAACTTATATATTGTGCCTCCCTAAAGAAAATCT<br>AGGTAATCAGTTCAAATAGCAGCAAGCTTCTAGTGAAGTATTAAAATGACAACACATCTGCATAATTCAAGGCTCTAAACTTAATGTTTATCATTTTGAAACCGTAACAACAACAAAAAGAATGCAGGTACACAAAAAACACAT<br>TTAAAATGGACTTGTCAGGAATGTGTGCCAGTGCATTTGTAAAGATTATGTTTCTATGCTAATCGACAGTCATGCATACCTCGTTCTCATATTGAATTTCCAGCATGGCTCTAGAGCTCCCGAGATCCTGCATTACTGACTCTG<br>CTTGCTTCAGAAGTTCATCTCGGTTTATCGTTCTCTACACGTATGAGAAAAAAAAAAGGTTAGACAACAGAAAAAAAA<br>3:**GTCAGTGT**GAACATCCCAAACCGCATGGAGTCTGATCTTTTCAGTTCAGATTTGTGCCACTTTCATATGTAGTCTTAAATCAGAGACTGATCTGATGTTTTTGTTTATGTCGGATTTCATGTGACTTTTACATAATCTTCGA<br>GTGACTTGCGTCATCATTCTGTGCTGCAAACATTCTCTACTCCTCAGCAGCATAGTAGAAAGGCACACAGGGCGATTACTGTACCTTAGAAAGTTGGTTTTGAAGGCAAAACTGATGCTTGTTAACTAATCTGGTTAATGATTG<br>AGGCACGGGTTAATCGCGAAGCGTTGAGTGTCGTTGGTGTTGCGGATActttgcaGAACGTTCTCTCCGAGAGAAAAAAGGGCAGGCGCTTGAGCACTTAAACCCCCGTTCTGCACATGTCTCCTGTTTAAAACCAAGTGAAAT<br>TAAAAAACTCTACAAACGGAGATAATCCAACTCTGCCTTCACAGTGCAGTCTGCGGCTGCTCATTAACCCTTTTTGAGTTTACTACACCGGTGGTCAGAGCGCCacgcactgcGGTTGTCATaaatcaCAAATGATCAGTGTTT<br>TCAATCGCAAGAGACTTATTTTCGGTCTCAAAT
Gateways-45A	5:**CTACAAGC**ACACCTAAAGTCGTCTACTTAATAAGTGCATCTAATGGAACAAATGAAGGCACTTCTGCTTCTGTATCAAAGAGCCAAATAAACTGATCAAATGTTACCACAAAGCAAAGTAAAAACATTAGGTAGTACGTCAT<br>GTGTTTTAAAAGCTAAGCTAAAAAATAATCTACAATGCTAGCTAGCTTGCACCAAATCATAACTTGAATTTTAAGCAATCTGTATTTCAACTCTTTACTCTTTTGTAGGTTAAAACTAGCTGCACATGAGGTTTTCTCCTTTAA<br>ATGTGTCCAAATATACACAAACATTCTTTTTTTTT<br>3:**GCTTGTAG**AGGTGATTGTAGTTCCAATTTATTTATGCATTTGATAGACGCATATTACATTGCATTCAAGCTATACATTTGATCAGTTTAGGCATTTCCTGGAAACTTAACCCATGGCCTTGTCATTGCTACGCTCTAAAGAA<br>TTGCTGGGTTGTCCATTATCATATCCATATTTGGTTGAAATAAAACCAGCATTGATTGGAGTGTAGCAAGGTGTTTTAGGGATAGTTCACTCAAAAATCAAGCATATACTCCACTATTGCTTCCCCTTAAGTGGTTCCAAACT<br>AAAGTGGAACAGTGTTTTTGTCCTGTTGAACACAAAAGATATTTTGAAGACTGTAGGTAAGCGGTAGCCATTGACATCCATAGTAGGAAATAAAAATACATTGGAAGTCAATGGCTAGCTTTTTTCATTTCTGGGTGAACTATT<br>CTGTGAACAGTTGTATTTGTTTTGCTGTTTGAATGTTGAAACCATTTCAACAGAATAATTCAGAATCATCTTTTGTTGATGGGAATGTACCACA
Gateways-45C*	5:**GCATGCAG**ACTACTGAACTGATGCGGTGTGGCACCTTAAGGAGTCGCTCGCGCTTCAAAAGATACACCAGCGCTATAAACGTGAACGAGGAATGCACTCAACACAGATACACACACACATTCAAACTAGCAATTACGGCTTA<br>TCGGATACACACACATACGGTCCACTATCCTGGAATTCTGCCAATGGATCCGATCAGAA<br>3:**CTGCATGC**AGATCTGCGCTCAGCCTCATATGCACTTCCAGTCCTCGATGACGTCACCGAGGGTGCATACAAGGCTGAATGCAGGTCTGGGGGAAACCTTACTTGATCCGAGCCAAAAATCAGCTGGTCGAACCGGTATTTGA<br>TCAATAGCCTATAAATGGCTTTTACAGTGCTATTTTATTTGGCTACATTTAAAGACGCACATAACCAATAGCCTATAATTCATTCACAAAGAAAAGTGTTTTTTATTTATTTATTTAAAAGCAAGCAAAACAGTCTCCCCAGGT<br>CATTTGATAATTGGTTTGATTGACAGTCAATCACGAGGCAACAATCACCAACACGTTGTGCTCTGCGTGATTGGGATAACTGAAGTAAATTAATATTCTCATTCCACTCGGTTTGTTACACGGCATTCCTTTTGTCAGGTAATT<br>TTCAATTCCTGACTGTAAGCACATTGAATGTTTGATGTTCAATGttcaatgtttgattcattgAGCTTGTTATGTAAATTTTAGTTCAATAATTTCAGTATTTTTTT
Gateways-46	5:**ATAATGAC**TTGCATGAGTGGCTGCCTCTCATTCCTGATCTATGCTCCTCCACAA
Gateways-50A	5:**GTGCGTGA**GGAGGGAGGACGTCTTGCTCATGCTTCTATAATCGATTCTAAAAACAAATATCGAAGCAAATTTGACAAAAAATGCCAAAAGGTTGTGGAATGAGCCACTTTATTATGTAATATTGTGACTAAACTCTGCCTCC<br>GCAAAAAAAAATCAATGGTTCTAAATCGCAGCCTGTTGAGCTCCACCCACTGATTTAGTAGGAAAATAATGAGATGGGATTATTATCAGAAAACAGTGGTTGATTTTTATTTATTTTTTTATATATATATTTGAATTTTTCAAT<br>GAATCCATACACATAACTGTACTTTTAAACCTTTAGGGGCAGATAAAGTCATTAAAACCAACACAGGCTCATTGTAAAAACGATCCCCTGGATACATTTCTGGAGAATGCAAATTATGTAGCCAGAGCTATGTATGACTGCATT<br>TTGTCTTTAAAA<br>3:**TCACGCAC**TCATATTGCACTTTATGTTCCAGGCTGAAGCACACTTACATCATTATGATGCGAGAGAAGCTGTTAAGCGATGACCCCTACATGCCCACCGAGCACAAGCCAAACTAAGTCTAGCTGTGCCCCAGCTCTTCCAC<br>GGACAGCTGACGAGTCAGTCTTAGGGGGTCAAACACGCTTCATAGGCACGATTCAGATCATCAAGTGTGAGTACATCCTTACAGCACAA
Gateways-51A	5:**AGCAAATC**TAATCTTGGATTTTATTTTAAATGAGTGTGCGTGTAAAACAATATAATTTGCAAAAGGGATTTATGGGGTTCTTCTCTAAAA
Gateways-51B	3:**ACCCTAGC**AACTGTCCCGTAGACTTCCATTACAAAAGACTCTCAATGACTTTACACGGGATCAAACTTTGTGAGCTCATAACTCTGCATCAGACAGGCATAGAGACTAATGGCTGAGCTCATTTAACTCAGTCTAGCCAGCA<br>GCCAATCACTGATGGCCTTTTGACTTTCTAGCCACACCCCTAACTACCACATACTGCACCCTAGCAACTGTCCTATAGACAGTAATTAGCTTTGCTATCAAATGCTTATAATTCTAACATGCTAATGACATGCCAATCATGCTA

	GCAACATGCTACTCATATGCTAATCATGCTAATGACATGCTAACTCATACTGGAAACATGCTAATGACATGCTAATTTGTACTAGAATCATGCTAGTAACATGCTAATTCATACTAGACTCATGTTAATAACTGGCCTACATGC ATATCTTTGCTTAGCAGTGTCCTATTAACTTGGGGGATGGCTCATCTGACTCAGACAACCAATGAGCATCTCAATATGATAATGAAGCTCACAAGCCACGCCCCCAAATTGATTCCATAGACTTCCATTAAAATAGGCCAAGAT GCATATCTTTGCATAGCAGTGTCATACAGACATGGGGGTTGGTTCATTTGACTTAGACAGCCAACCACATTAAAGTGCACTCTGTAACCTC
Gateways-51C*	5:**ATGACGCC**ATGTGATATGTCCAGCTGTCCGGTTTGACGGCTCCGTTTTCAACTCCGCCCCCGCCTGCGCTCGGCTAATCTCGTTTATAATCGGCTGCAGCTGTGCTTAATGTCGAACGCACCTTTTGTCTATTCTATGTTTT GATTTATTTTATGATTATTTTATTGGTTTGATTATTATCTGTGCTCTGTCCTGTAAATGTAAATGAAAATGGTCGAATTTTGCCTCGTTGTATTTGTGCTACTGTAATAAACAAATTAAAACACGTGACCAGAGTTCTGACATG GAAACGGATGAGGCGATTTTCAAGTTCAAAGGTGAAAATATCTTAAAGTAATAAAGAAAAACGTGGACAATATTTATTTAACTGGGTATATGGGCCCTGTATTATTATACCTAATATTTATTAAAAGATTTTCTGTGTTTAATA ACATTTAAAAAAAAAA<br>3:**GGCGTCAT**CATCATTTATTTATATATTTATAAAAACAAAAGATTTACAATTAAACCAAAAATACACAGGTGGACATACCCTCTCACCTGATGTATCAATACAAATGTTAATTTACGTGCTGATATGTATGTCACGTGACATA CTtgcaagttgcaAGTCTTACTAAACTCACGAGGAAACATGAGGATAGGGAAGATATGGTGACAAAAAAGTAAGAAAAGAC
Gateways-52A	5:**CTTATTTT**CACATCCCAAAGTTGACAGGAAAGACATGTGGCTCCAGACAACACTCTGTCCTGAGTGTATTTCTTCCAATGACTAATTGTTTCACTACATAAAACATCAATGTATCATCAGGCACCACATGTATTACTTTCAT TTTGACTATATATGCTTTTTTTTT<br>3:**AAAATAAG**GGATACCCACTGCTTCTATATTTATTTCAAAGTGTTTTCATGCCTAAATAAAATAAAAGCACAGAACAAATTCACATTTAAAAGCAATGGGCGCCCCCTGGTGGTTCGGCGGTATAGGTTGCGTATAGGGAGGA TTCGCTCTTTAATGTTTATTGCCACCGTTCATAGAAAGATTACAAATATGGGAGAAATATGGGAAAATACCTTTACGGGATGATAGTAGGATAGAATTCCGGAATACGGGAGAATCCTGGCAAAGACGGGAGGGTTGACGGGTA TGCCATAGGTATTAATTTAGTTTTGCCTGTGTATATGTGGTTTTTAAGTCCTGGAAGCCATTGACTTGCATTATAAGAACCACCAATAACCACAGTCTTAATAAGTACATTAAAATAAAATTTTAATTTTTGATCATTGTTGTT TTGTTTAATGTTTAAGTGAAACTTTTTAGAGTGTATTTATATGCATACCAATGAAGTTCATCTATTAAAAAATGCCAAACTTGCACCTCAGCACTTAAGCGGTTATTATTTTAAAGCAATCAGAAAACATTAATTTTGTATTTA CAACTGAGTTTATTTTGATTTGGGTTGTCTTATTATAAGATTTAGTTGAATAATACTTCAACTTCTGGCTCATTTTAAAG
Gateways-52B	5:**CCCACAGG**CTTGGTCAGAGCGCGTCTCTGATTGGCTACAGTTGTTTCCCGCAGGCGTTTCCTTCTCATTCGTCCAATCAGCGTTGATAAATTTGCATAGCGGCTTCGTGATTGGACGGGAGATGTTGTGGCTCTTTAGAGCG CCAGAAAACAAGCTTTAGCGGGTGATTCATACTCGCCTGACCAGCGGTAGCACTGAAATAAGAAGATTACAAGCTCAAATCCTCTGATTTACAGAGAAATAATGCTCATGTGAAGAGGAAAACGAAAGATCAGCCTTGACACAG GATTTATTTACGTTTTTAGGATTTTTTTAATTACAAGGACGGAGGTTTAAGGTAAGAATATACCTGATCTACATCATCCATCTGACTTAAAGTTACGTAATGTAATAAAAATAGTATAAATCAATTTATTTTGTTAGTCACAGA TGTTTATTTTAGTATTCAAACAATCAGAAAAGCTTATTATTTATAATAAGCGGTAATTTATATTCCATGTATAGCTAAACATCTATTATATCTAACCTATATACGCAAAGTGTAAATATATATTATATATGCACTTATATAATG ATAATATAAATTATTTATAAAGTTAAATCGCATTGTTTATCTTCTTATAATATTACTGGTTTAGTTACCAGGCAACTGG<br>3:**CCTGTGGG**GGTTTGTTGGCGGGGCTGTAGGCGCGCTCTCGCATCGATTAATATTAAAGGCGCGCCTGAAGTTTCTTAAGGCGTATCTGTAACTCAGCGGGGACATAATTAATCAGCACATATGATTAATCGACACGATTTCA AAAGCAGATGGTCTCCTGACAGCTTGAAATGACAAGCTCAGGCAGGAACAAAGGTGATGTTTGACACAGGTGGGGTTTTGGCGCGGCTGACGGGTTTTCTCGCGGGAAA
Gateways-52C	3:**CACACCTC**GCCACCAAACTCTCTTTCAGGACTGGAGCTTTCATTCAGTTCTGTACAGGATTACAACTGACGCAGGACGTATTTGATATCTATTGTAAAAGGAGAAAGTGACGCACATTTCAACTCTCAATATATTTCCCGTC GAGTGTGCGCCTGGGTAAGTGATTTTTCCCCCACATATTTTACAAATAGCTCTTTGTTGTTCATATTCAGAAGCAATACTGATTTTATAATTTGAGACTGACTTTTTGTTCTATTATATCGGAATTAGAAGTTGATAACCTCAG TGATGTTTTATTTTTATTTGCTATTAATTCTTTATTTAATTAAAGCAATATCACATCTGTACATCATACCTTATCTGATTATGTAGCCTACTGTAGTATATGTGCATAGTATTAGGAGAGATAAAACTACTAATCCTTGTGGAT TGGCCTACATACACAG
Gateways-52D	5:**GTTATGCC**ACACCGATGCTTAACTGTTTTCACATTGACGTAATCACAGACCAAAATTTGACCTACAAATAGCTTATACAGAGCAGGCTAGGTGTTAATTAAAACTTTTTGTTGATTGCGTACATTCTTGGGGCAAGATAACA GCACAGTATTGAGCAATGTGTCAGTTTTTAATCTTGCTTTATTCTTACTGTACTATAGTTTAATGCTGATATCATACTTCCATTATTGTCATCATATTTTCATTCTGTTTTAGTGGTTGGAAGTTTGAAAGCTTTGTCATTTTC AACTGTTTTAACTGCTTGAAATCATTCACACAGGTAAGTGTATTTCTGCTTTTTGTTAGCTGTTCAATCATTGTGTTTATACACTCCTACACTCTTTAAAAAGTTCCAAAATGTATTTTTTGCAGCAGTTCCAAACTCTAATTT ATGAATTCAAACACAAGTCAAAAATACTGACACTCATTACCGAGTTTATGCACCATAATTTTAAAATTTCATATTTTTTATTTTACAATTATCTTTTCTACGCTTTTACATTATATCTTATTG
Gateways-53A	5:**AAAACAAG**CCCACAAAAAGGCGGGACTGGGCAAGGCGGGTAATCGGGAGGTTATGATATCTGCATTTACTTTCTACCTTGTCCCAAAGCTGAAGGCAAATATTTCCAAA<br>3:**CTTGTTTT**GCATTGTCAGTGAACGTTTTTCTGTTTTTTTTTATATTATTTCCAAGAACCAGCATGTACAGTTTTTTACAACAAAATCACTTTTCATTTCTTAACAAGAAATGAATGAATGAGAAAA
Gateways-53B	5:**ATGTAAAG**TCAATGGGGACCCCTTTGGGACGTCCTAGCACCCCAGGGGTACAACTTATACCCCCTTTGGGGGTATGTTCTCATACAGGCTGCAACCCCCTACAGATGTTGCGAATCCCATATTTCTACAAATTTCACACTTG GCGCTATGACGCTTTAAAGGTCGTCCCATTTCGCGGGGGCGTTAGTGCTGTTCTAGAAAAATGAATGGGAGTCAATGGGGCCCATGTTAGTCAATGGGAGCACTTTGGGACGTCCTAAAGCCCCAGGGGTGCAACTTTTACCCC CTTGCGAGGTATGTTCTTACTTAGGCTGCAACCCCCTACAGATGCTGTGAATCCCATATTTCT
Gateways-54*	5:**GTTTTACA**TTTATTTATGTGAGACACAGAGATAGTTTCAGCACAGTATCCATTTTAATATTGATAATAGTAAGGAGTTGTATAATTTTATTATATAATATTTTACGTTATTTGATTGATTTATATTTTAATTTATTTAATTT ATTTGTTTTTGATGTGTTCACAGTGAATCACAGCCTTTCCTACACAGAAATTGCAGTAAATGGTGAAAATATCCCATCATAAAGACTAGAAAAGCATCTAAACTATCAGGAGATCATTCAGGTTGGTGAATATATATAATATTT TTCTTTTTTTATATATTTGCACTGATTTCAGGTGTAAATTATTCAATGTTACTGGGTGTTACGGCCCTTACCTAAATACAACTCTTGATCCTTAAACAAAGTAACTAGGGTTGGGCGATGTTGAACAATTTGGCATCGTACCAT GTCTAATGTGAAACATTGTGATGTATGATGGCATCATTGTTGCAGGCGGCGGTGAATTAATTATTATGAATAAAGAAAAAATTCTCAACTAATTATTATTTGTAGCCTAC<br>3:**TGTAAAAC**ACCATATTTGATTATTTGAATATTATACAGACTTCACACACGAACCTATTTAAGATTTAGTGGATTAAACTGAATATATGTCAGACATAATGTTGTTACAGTTAATCAAAAGCAATATTACGCTAACAGACATT AATTCTTTCGTTTATAAAAGGCTCTTACTCTTTTCGCTGCCATGTTCCCGCCGAGTTTTCcttacatctgtagaatCTCCGTACTGCGCATGCGTGTTTCACACTGAGCGGCAACTTCCTCCAGAGGGCGCTTTAGCATCGAAA GCCAAAACGCGGTAAATATATCGCTTCAGTTTAGTCAGCAAAGTATTTGTCCGTAATTGTCTGAAATCATCATCTAAACGTGCATAACTATTACAAATCATTTATAAATACTATAAAACAATATAACCAATATAAAGCA
Gateways-55	5:**TTCACTGG**ATAATATACTTCTGCAAAATTTGGATGAACAGATATCTTGGTTTTTTTGAGTCCATCCTTGGAAATGCATAATTTCTAAAGCACATTTGAAACATAAAGAGACCCAAAAGGGATGTGATGGTGGGGAACAAAAC AAAACAGTAATAAAAAAATATGAATTTTTAGGTTTTTGCATTCCTTTGCAAAATTGGTGTTTAACAAACACCTCCTGCTCACTTCTGTGAACCCTGCAGTTTTTGCCAGTATTCTCCTTTATTTTACTATTACAATACAATTCT GCATATTTTACCAAATCATCTTTGCGAGGGAGCAAAAACTTTTAAAAAATTTATTTTCCTATCACTTTTTTCTCCCATCAATTTTTTTCTCCCACCCAGTTTTCTTTTTTCCACCCATTTTCCCCCACCATCATGTCCCTTCCG GGTCTCCGTAGAATCATATGAAAGTATGCTGCATGAATAAAATAAATAAATAAAGAAAAATTAAGGTATTAATAATTTTT<br>3:**CCAGTGAA**CATAGTCAGCCTTTGAAGTGGATCAAACTAAATGATCAAAATTGTCCTAAAACAAGATTAGATGCTGTTCAATAGTTTTAAAATTTCC
Gateways-56A	3:**GCTATTGA**TGATAGTCACTTACTGCCTTCACCATAAGGCAAAGCAGCTCCAACTCCCACTAAACACTCGGCTTATGCTAGTTTTGTTGAATAAAATCAGCAAACAATGCAAAAGAAATATGACAACGAGATGCTGCGCTGCC AGAAACTGGTATTATTGTCGGCTAACGTTAGTGAAAGAGTCCTTCATAACAGAGATTCATTCACAAACGAATCGCTCCTCCGTCAGTATGAGCAGTGAAAGCAGGAGAGGAGCTGTGTTTCAGGACATGATTAGATCAAATTTA ACAGGGAGGGTGAATAGTACATTTCTGTACACACAAACACAAGCTTTTTGTCAGGAATGCCCGTGCGGTCACTGATCCATCAATGTAGAAAAGTGATGTCAAATTATAATTTTCGTAATATAAAAGAAAATTGCATGCTAACA TCCAGGAAAACTCTTGATCATAGATATATGTGTGTATGTGTATATCTCTGGCTTTGGATGGCCACAGTCCTCCACTGTACCTTGGTCCCGCATTCATTTTAAAGGAGCGCTACCCTGTAGCAAAATGGCGGCTCTATTGACACA

	TTCCTTCCAATAGACAACAACAGGGTAGGCGACATCTACTGTATATATCTATG
Gateways-56B	5:**TTGAGCTG**TAACCATCTCCTCATCTGCTTCGGGTTGGGTTTCCTTACTGGGTTCTGATAGAGATGTAAGCTGAACATCAACATCATCCCGCATCTCGATTTCTGGAGGACATTCGGGTGGAGGCTCGGGAACGTCTAGACTC<br>AATGCATCCTCCATTGGTGGATCAAAAGCTGGTGGTGGATCTTCAACTGGCTCCGGATCTGTCTGCTGGTCTGGCTCCGCATCAAAGACTGGCATTGCATTGAGAGACACTTCCTCATCATCTATCAAAGAGAATGAGTAGATC<br>TGTCAATATTGGTAAAGGGGTGAGGTAAGAATTAACACAAGAGTAGCTATAATTAGTACATCTGTGGCTATATTTAGTACAAAAATTAAGCATTTGTGCGAGTAAATTACATATAAAGACAATGCAAATGCATGAAAATAGGCA<br>AATTATTGCAGTGGCACAAATTACATGGAATGTGCAGCGTGTTTGCCGGTCATGCACCAAATTCACCTTAATCACATCTACCGTGCAACTTAAAATAATTGAAAAACACCCCTGGAGTAATGTGGTGCTTCCCGCTTGGTGTGT<br>TTGTAATGCATTAATAATACAAATTCGAAACACTGTAATAAAAACTATTAAATACTAGTCTCTTTTAAAGGCACAGGGTGTCTTTTTTGCCACTAGAGGGCGTGTATTCACAACAAACAAAGGCGTATTTTGATGATACTCT<br>3:**CAGCTCAA**GGAGATACACCAATGAATGATACAACTCTTGGAGAAACTGCAGAACAAGACACTGGTATAATGGGGGATCATGGAGAGACGCTGGAAGAAACTATGGCTGAGACTCCATCGGAAGTACTGGACAACCGTGATGC<br>TGAACATACTGATGGAGTAAATGAAGAGCATGAAGTAGAAACAAGCTCTGAACAGGTGGATCTAATGGTAGACGGAGATAATGAATGTTCCCCAAGGGTACGAGTTCAGTTAAAGCGCAAGAATCGACCGTGCTCGCTTCCAGT<br>ATCTGAATTAGAGACGGTTATCGCCTCGACGTGTGAAGAACCCGAAACGCCGCGCTCTCATTACATCCGCATTCATCATCTACTCCACAGTCTGCCGTCAGCTCAGTGCCACCGAGATGAAGAGGAAGATGCACCTGATCCCGA<br>ATCTTCAGAAGAAATCACGATCAAACCTCAAGAAGATAAAGAAGACGAAGATGAGGTATCCGAAGCTCAGTCTCCGTCAGTTGTTCCTGAGTGTCCGAGACCGTGTTGCAGGCGAATTTTACCACGTAGCCTCTCAATCGAGCG<br>TCTTTCTGAACTCAACCAGCTTCTGGAGGGAGAAAGACCGTCTCCGTCCACCCTCCGGATATACAGTGAAGACGGGGATTCGGGAAGAAGAAGGGAGCCTCGGGAAAGCGAGTGTGAATTTTGTGATAACTCCTGCTATAGTAC<br>GTCATGTTACAGCACGTCCTGTTACAGCA
Gateways-56C*	5:**CCAACTCA**TTCAACATATTTTTTTACACAGCAGATGGCCTTCAAGCTGCAACCCAGTACTGGAAAACACCCATACACTCTCATTCTCACACACACACACACACACACacacacacactATGGACAATTTTATCTTATGGAGC<br>ACCAAAAGGAAACCCACGCAAACGTGGGGAGAACATGCAAACTCCACACAAAAACACCAACTGACCCAGTCGGGACTCAAACCAGCGACCTTCTTGCTGTGAGGCGATTATGTGGGATTTTTAAAAACAAACTAAAATATTTA<br>AAAATATAATCTTTTCAGTATGTCTTTTTATTTAATATCATTTAATGCACTAAAATTATTAAAAACTAGGGCGGGGTGTTATATACACATATAAAAACATGAATACTCAAGTACATTGCGAAAATACAAATTTGTTCACTTGTT<br>TTGGATGCATTTGTGATTGTGATTAATCACGGCCCAGGATTAATAAAAACTAAAAATAAATGACATAAAATATTTCATATATTAAAAATATAAATTAATATATTTTTTAAATTCATCATATTTTTATTTAACAAATAAATAAAT<br>TAAATATATTTTAATCACAACAAACTAAAATTATTTTAAAAAATACAAAAAAAAAATGTAGTGTACAGCTAATTTTCTAAAAC<br>3:**TGAGTTGG**TGGTTCATACCACTGTGGCGACCTCTGATTAATAAAGGGACTAAGCCAACGATAAAATAAATGAATGAACAATTTGAGATAAGTATACTACTTTTGGGTGGCACAGTGGTTAGCTATATGGCCTCACAGCACTC<br>AAGTCTCTGCTCCTCCAC
Gateways-56D	5:**ATCAAACC**ACTTAAAATATTTACCATCCAAAAGGACATACAGATGTTACTACTTCAATGTGTTCCTTGTGCCGCCATTCATACCGCGGCGACGGCGGTACTTGATGCGCCAGCAGCATTTGACCGCTGGGTGGCACTTTAAC<br>CACAGATATTCAGCTTTGCCTTTTCACAATGCTAAACAGACTGTTCTGTAGGCGTAGCTTACTGTATGTGATGTGATGTGTTCAATTAAAATATAATTTTAATTTAGTTTTTCTGGTAATAGACATGCTCTTACACTATACTAT<br>GCTGTAGAAAAGATACATGGTGAGAAGTCAAAAAGCAAATATAAGAATTAAGTTATTAGCCTTCAGTGCCCAATTGAATTGCG<br>3:**GGTTTGAT**TTCTATTGAGTTTATTTTAGGTAACTATCAGAGGAGGGTAGAAACTTATTTGTACTCGTGTAGCTTGATGACTAGCATATACTGTATACACCAGAATGCATAAAGGGTTATAATAAGGGAGAAGATCATTGTAT<br>GGACACTGTCACAATATGTGGATGATTGAATTATCTACAGCTTCACAGAGCTCTAGTTCAATTAAAAGACTTGAGTGGAGACCATTGCTCCAAACAGTGAGATGATGACTGTTTTTAGTGTTGACTGGATAGAGAATTATTCTG<br>TAAACAAGCACAGTTGAGAAACAATATCAAAAACACGCGAGAGAAAATGTAAAAGCATTAAATTAGGGGTAAAACAAAGTTATTTAAATTGAACAGTGGTAAAGTCAGATTAAATGCACCGTTGAACTACAGAAAAAACAAACA<br>TCTTATGCAGATTTATACTTTCACCTGGCACAG
Gateways-57A	5:**ACAAACAG**TGTCAGTGAAGTAGCAGGAAGTTTCCTGAAGTGAGGCACTCGGTGACCAGGCCATCCGAGTCCACCGCGGTTGCAAGGTAATAAGCCGTGTTGTTGGTGGGATTCCAGGAGAACACAATGGCATTAGTAGAGCA<br>CTCACGATATAACATTGGTGATTTGGGTGGGCAGGGTGCTGTGTGTTTGAGAGACAGAAAGAAATTCATTAAATTGTGCATTGATTTAGCTCAAAATGAGATAATGTGAAGAATATGAACACTACAGCGGTCAACATTTGAAGT<br>GGATTAAAAAGTTTTTCAAACTCAAATTAGTTTAAAACAAGTTAATGGTGAGTAGTGATGACAGTGACAGTATGACAGTATTGTCATTGTGGCAGCACGGTGGCTCAGTGGTTAGCACGGCTGTCTCACTTCAAGAAAGTCACT<br>GGTTTAAGTCCCAACTGGACTAGAAGGCAGTTCTGTGTGGAGTTTGCATGTTTTCTTCGTGTATGTGTGGGTTTC
Gateways-57D	5:**CTAAAAGT**TCATTTAAATTGAATTGAATTGAGAGAAAGAGAGAGAGAAGGGGGAGGAGGAGGAGCCGCGAGCGGTGCATCGCACATCGTCTCCGCCAAATCATCTGAGGCTGCTCTACTACACCGTGAAACAAAAAAGGATG<br>GTGCCTTCCACAGTTCGTTTTGTACTGGCAAAACAGCACAAAGAGGTCGTCTAATGACTTTTTATTTGATCGTCGCGGATATAGCATTAAATGCAAGTACTGGGTCAAGCTGTGTGGGATCTAACGCCAGGCGTTCTCGAGAGC<br>TGTTTGTTGCCCCGTGAGGATTTGACGTCTGTGTTCTGCTGGATGTTTTGGAGAAATCAAGAGAAAGAGGTTGCGGTCATTGTCCTGCAAAATGGACTACTTAACCGAACTGGAGAAGTATAAAAAGTAAGTACATTAAAGCTA<br>CTGCGCGTTTGTTTCGGCATGCGAAACATACCCATTGAAAGCCTAACGTTACACACACTACAGATCTCAACCAGTCAACGTTAGCAATTGAGCTAGCAAGTAATTTTGCAGCTAGCTCGCTAACCCACCGTTTACAGTTAGCCG<br>TTTTCATGATTTCACTGCATATCCCACAGGTCAGAGAATAAATTTAGGTTTTCAGACAAAATACTGCAGTTAGCCCGTTTAACCAAACCAGTAGGTTTGTTGGCCTTAAGGATGAA
Gateways-57E	5:**GGAATGAA**CCGCCAACTTATCCAGCAAGTTTTTTTTGTTACGCAGGGGATGCCCTTTCAGCCGCAACCCTTCTCTGGGGAACATCCACACACACACTCATACACTATAAACAATTTAGCCTACCCAATTCACCTGTACCGCA<br>TGTCTTTGGACTGGGGGGGAAACACACCCACGCGAACGCAGGGAGAACATGCAAACTCCACACAGAAACGCCAACTGAGCCGAGGCTCTAACCAGCGACCCAGTAGGTATTTATCAGCAACATGGGGAAGAGGGGTAAACACCA<br>AAATAAATCATGTCCTATCATAGTTTAAAGAGCCCAAATTATGGGTTTTTGAAAATGCCCTTCTATGTAGGGGGTAACACAGCTCTAAGTGAAGTGAAATATCCAGCTAAGGCTTAAATCTGTAAGTGTACAGGGTTTAAAACT<br>GTTGATTCATCTATAAAAGAGTCAACTCATAGTGCTTCAAACGAGTCGTCTTGATATTGAGTCATTAGGTGTTTCGCGATGACATATCCACAAAAATAGTTGTTCACGC<br>3:**TTCATTCC**GCTGTGGCGGCCCCGGATTAATAATTAATTCTTTGCAGTTGTTTATTGTTTTATTTCTGTACATTTCTTATTGCTGCTGTTGTTATTGCTTGTATTACTGCAATGACAATATAGTCTCTTTAGTCTTTACATTG<br>GTGGATTGGTGTTTATAATAGGTCAGTTTAATTTTTTTTTACATTACTATATGAAATAACAAAACTGACTAATATTCTGTGATTTCTAAAATATCAATAATATAAAATGAGTTAAAATACTGTAAGCCACAGGCCATAAACCAT<br>TTTTTTTTT
Gateways-57F*	5:**TCTGAAAC**CCCCTGCGTGATGGGATTGTCTGATCTTCTGCTACCCATGAGAACTGCTGTTAGCTGTATGGATGTTGTGATAATGTTTGTTCTTCAAGTCATTAGCAAAATCTAGTGAGCTGACTAGCTAGAAAGCGTTCTGC<br>GGTGTCATATGTATGCACCTTTAAAGCTTAAAATATCTGTAGAGTCAACATTTGAAGTGGATTAAAAGATTTTGAAAATTGTCTTAACCCTTAATTTACAACAAGTTTTGAAAACACTTTGAAaaaagtcgagacaaactttat<br>cagcgcttattaaCAGCAGCTGTTACGGTGGCCTAGTGACGAGATCTGTCCTCTGTAAGCGAGATCGTCTTATTTCAAAACGCTCGGTTCAGCCTGTGTGGCTCACCTTTGAACTCTGTTGTCCTCAGGTTATTACGTGATCAT<br>CTTTAGCTTTaatttttttaccCGGCAGCTCGCTCTCT<br>3:**GTTTCAGA**ATCAGCGCAGTGTCCACGCAGAAGCAATCAAAGGGAAGCGTCAGATGTGTTCAGAGGAGAGGTTTGAACTTCAGCTACTGCAATTGGCATGAAAGCATGAGGAGTGGTTGTGATACACGTCTTTTGATGTGTTC<br>GGCTGAGAGGGACTCGCTTCTGTGGGAAAGTGTGAGTCAGTCACTGATCAGGGTGGACCTCTGCATTCAGTTCCCTGACTATCCCAAGGAAAGGGTTGAAAAACTCCTTAAAAATAAGTGTTGAACGCGTCACGACTTTTAAAT<br>CATGGTGAATATATTACTAAAGGAGCTGTTGATGTGAAATGGACCCAGATTTTGGTAAAATAGTTTTGCTATTACTGTATTCTGCATCATAAATGCAGCTGTGGCTCACACAACAGAGGAAAGGTTATCAGCTTTAGGGTTATC<br>AGCGTTAACATGTGGTTCATCGAGTGGAGGAGTCAGGCGAACTGATTCAGTGAGCATTGGAACTGACTCAGACCACAGACTCAAAGTCAGTTTGATTAGCGAGTCATGAGCAACCACTGCTATTGTGTGTATCTCTCTCTGATC<br>TGACACACACGGCTCAGTTCATGGTGCTCTTTGCTAACAAGATGAAGATCTGAGTCAGCTGCACCGAACAAGTGTACAGATGGACGAACAGCTAAACTAAAAGGCGTCGTTAAAGAAACGATGCAGTAAACCCAGAGCTGGCCA<br>ACAGATGCAGAAACCACAAGAGAAGAAGCCAAACACCACAGACATCTGCTGTGCGCTTCACCACACCGAGCCCTG
Gateways-58-1	5:**TCAATGTG**TTATTTGGACCACTGTTTCTCCAGTGCATTGGGTAGTCGGTTGGTTTGAATGTCACTTGTGTGCATTGGATGCTTGGTTTCAAAGGTAAAGGCCTTTGTTTTGCAGTAACAGCAAGAGATAAACTATGAAGTCT<br>TCCGGATGTCCACTCGCAGTTTGGAGACATCAGCCCATGGTCGAACGACTCTGGGAATAGAGGTCTGTACAACACCGAGGAGGACTGAGGGGTTAATTATGGTCTATTTCAGCCAGTGCAATGACTTGTAAAAACCCAAATAAT<br>[illegible]

	3:**CACATTGA**TGGTACACGCATTCAAAAACATTGCTGATTAGACTCGAGATGTCCTTGTTTAGGGTGCTGTAACCTATTAGTAGTGTTTCATCAGCAATTCTGTACCAGTGCCAAATGCTCCTCTCAAAACTGATTCCATGGTG AACTGTGGCTTAGCCACGAACTGTATATATAAACAGGGCAGCATTTCAACCATCTACTTGAATACTGTACAAAGAACAACAACAACAACAAAAAAAGACAAGAAACGAAAGTATGTCAATCATCACAAGAGTCCAGACCACTTG GGATGACTAAACGGGGAGATCACACGCTGGTTTTCTTCTTCGGGAGAACACAATAGGCCAAGAAAGCACTTCCTGGCAGCTCCCTTTGACGATCCGCCCTCATTCACCTCACCTATGTGGGCTGACAAGGATCCGTTTGGTTCA CGGCAAAGTGGACCACCACAATTCACAGAGTCCGCCAAACTGTACTTGACAGAACCTGGATGCGCTTCATCTTTTAGGTTAAAGATACAGTGTTCCTCTAGGCTGGGTCAGGTTTTGGCGTGGTCTGTATTGTTTATGCGCACA TTTAAACGATAAA
Gateways-58-2	5:**CTCTGCTC**TGAGACCCCGGATTAATAAAGGGACTAAGCGGAAAAAAAATTAATGAATGAATGGTTGTACTTAAATAGTCGTTGGCTGCTAATATGATTTCACTAATTAAAATTTGCAAAGAATACTTTTCTGAAATTATATT ATTAGAAAGAATGTCTAAACATACAAATATAAAACTAACTTTATGAAAGTAGGAAAAGCTAAAGGGCAAATTAGTGCCAAATATTAAACAAGGCACAAATTTGAAAAGTTTTAACTGCTTTCTTATTATTCATTCATTCATTTT CTTGTCGGTTTAGTCTCTTTATTAATCCGGGGTCGCCACAGCGGAATGAACCGCCAACTTATCCAGCAAGTTTTTACGCAGCGGATGCCCTTCCAGCCGCAACCCATCTCTG<br>3:**GAGCAGAG**TGAACCGCTAACTTATTCAGCACATGTTCTATGCAGCAGATGCCCTTCCAGCTGCAACCCATCTCTGAGGAAACATCCATACACACTCATTCACTACAGACAATTTTAGCCTACCCAATTCACCTGTACCGCAT GTCTTTTAACTGTGGGGGAAACCGGAGCACCCGGAGGAAACTTACACGAACATGGGGAGAACATGCAAACTCCACACAGAAACGCCACAGAAACCTCGAACCAGCGAACTTCTTGCTGTGGGGCGACAGCACTACCTACTGTGC CACTGCGTCGCCTTAAGTACAACCACGTTTACAGTCAATTAGATAGTGCTGATGTTGTTTTGAATTCATACTTGAACATGCGAAGTCATGAACATGCGAACATAAAACCAACTTCACTTCAATCTTTCTTTTGATAACCTTCAT TCATTCATTCATTTTCTTGTCGGCTTAGTTAGTTGATCCGGGGTCGCCACAGCGGAATGAACTGCCAACTTATCCAGCAAGTTTTTAAGCAGCGGATGCCCTTCCAGCCACAACCCATC
Gateways-59A	5:**ACTAGCAG**TTGATTAGTGCTTTATGACATGGTGCGTTATCTTGCTGGAAGCAGCCATCAAAAGATGGGCACACTGTGGTCATAAAGGGATGGACATGGTCAGCAACAATATTTAGGTAGGCTGTGGCGTTGACTCGATGCGC ATGTGGTACTAACGGGCCCAAAGTGTGCCAAGAAAATATCCCACTCACTAATACACCACCACCACCAGCCTGAACCACTGATACAAGGCAAGGTGGATCCATGCTTTCATGTTGTTAATGCCAAATTCTGAGCAAAGCATCCGA ATGTGTCAGCAAAAATGGAAACTCATCAAAGCAGGCAACGTTTCTCCAATCTTCTATTGTCCAGTTTTGGTGAGTCTGTGTGAATTGTACCCTCAGTTTCCTGTTCTTAGCTGACAGGAGTGGCACCCGATGTGCTCTTCTGCT GCTGAAGCCCATCTGCCTCAAGGTTGGAC<br>3:**CTGCTAGT**AAGGTGTACCTAATAAAGTGGCCGGTGAGTGTAAATCCTTAAATAATCAGTTCATTGTCATACTGTATGTCTGATTACATAACTTGAATCAGTTTCATGCTGGCTTTCTCCTCACAAACAGAAACAAAACAAAC TAAAACTATTGTACAGTTAAAATTTTAATCAGGCTTTAAAGTCTGATTACAGTCTCTCGGTGGGACAGATGTTACGGGTTAATTACGGCACATATAAAGCACTCTAAAATCCCCAGTAAACGCTCCGGCTGGAAAACTCTGTGA TTGGAGGCCAGCGCTGGTGTCAGATATACCTGCACGGTCTCGCTCTCTCTGGGGGAAAATCACAGCGATGAAGCCCTCGTGACCGCTAACACGCCAGAAAAGACACTTCAAAGCCACTGGACACGTGTGTGACCGATGGCCCTT TCCTGCCTGTCAGAGATGAGAGCGGGAAAAGCAGCGGGACGATCTCACACACACTCGAACACACTCTTCCACAGCATCCGTCGCCTTCCCGACCGCTCGCCTGTCAGCTTTATGGTTATGGTTGGCTGATTGTTGAGTGATGGT GGAGTGTGAACAGACAGTGTA
Gateways-59B	3:**GATATTTT**ATAGACCATTGTGCATGTTATTATGATTTGTGCTGCTGGCTATGCATTGCATTACCTGACCATTCAGACTGCACCTCACATGTGAATACCAACCTATATTTATTCACTGCATCCACTAACAATTGCTGAATAAA ACGGATGTTTACATATTTCATATAGCGCTTCCTGAACGACCGTCTCTAGGATTGTGTGTATATTGGTGGCTCCGGGAATGCATTTCTATGTATGAAATGTAATTGTGAGGAACAAATACCTCTGAACTATCAGAGAAAATCAAA AGTAAAGAAATAAGAAGCCCATTTTTCACAGTTAGGATTGTCACAGGCTGGAATTGGCTTTGCTGCGTTTCCAGGATGGAAAGGAGATCTGTGAGCGCTCTGTCAGACATTTATTAATCCAGCAATATCTATTGGTGTGTGTGT GTGTGTGTG
Gateways-59C*	5:**AAACATGC**AAGCACCACAAAGTAGGATTTTTGCCGCATTTTGTGACTGGAATAACAGACGCGGCTTTCTGATGCTACCTACCGAGTGCATCTAAGTTACCGAGAAATGCAGAGTTTTTTTTTCTCTCATTCGCCA<br>3:**GCATGTTT**ACACTCGGAGAGCAGCATTTACAGCAGATTTTCAGTCCATAATATTGTAGTGATATTAATGTACGGTAAACTTTATTAACTTAGAAATCATTTGACTAAggtctgTCTTTAAACACTGAAAGTGTACTGCAGAC GATACCAACTAACTTTGCCTCTGAATAAACCAACAAAGACCTCTGATCGCTCACTTACCAAATCTGTAGAGACAGGACAATCAACACCAACTGGAGCTGCGTCTTTTTTAAAAGGAGACAAGTGGCAAATCCAGACTTCACCAG AGAAAAGCTCTCAGGTAAACAATGTTCCTTAGACACGTATTTCTATTGCACGTCGCATGCACTGTAATCCACAGGTGAGTCCAGCTGCACTCTCATAAAGGTAATATGTAAACAAAACCTTCACTGCAGAAAATAAGAAGAACA ACACTGACGAACCGTATCTCCAAACAATTC
Gateways-60A	5:**GTTTCAGC**TACAGAACTCCAATCCATTATATGGATGTGTCTATAATGATTAAATTTGTATCTTGTGTCCAGATTTACCTCCATTTTCACTCCACTCTGAGCACAGCCTTTATGGAAAAAAAGGCATTAAAAGTCAGTGCTGA ACTCCAGCCGGACTCTCTGGACTCAATAATTTGATCAGCGAGTTTCTCCAGGGGCTTTCAGTGCTGCTGTCAGCACTTATAGGACCCCCTGTACTGACACTTCTGTCTCTGCATTAACAGCATCCAAGTTCGGGCAAAAAAACA CATCTCAAAAACAA
Gateways-60C	5:**CCAACTGG**CCATAATTTGGCCGTTTTGTTTGTTTTGCCTGCCCAGACGAGGCCACTGAGAAGATTAAGAGATTTAGAGCGAGATTATTCTCCGGCCCATCAATGCGCTCCATCAAATAAGCAAATAAAATGGTTTGTAAACA GATGCTTTCAGAGTCAGAACCTTTTAAATCTCTACTCTGCACTGTGACTGTCCTACGTTATACAAAACGACATATTAAATCATCCAAACCAAATTGGCATTTCTGTTCGGCGAGATGCGGATCAGTCCTCGGAGGCATATGTTG TCATGCGGGTTCTCGATAAATACAGCTTGTGTGTGTGTCCTTGAAATTTCTCAAGCCAAAGTGACAAGCCAAAAA<br>3:**CCAGTTGG**GAACTTTGTCTCCACCATTCAGATATTATTTTCTCTGTAAACCTTCGCTTTTTGTCTCAACTTGATGACTCGATCCATTATTGTGATGAATCGGTTTCTGATCAATGCGATGATAATGAATTCTGGGATTTGTT TCAGATGTGTGTTTCATAAATGAGGACGATAACTCACTGTGACGTTAATGAAAAGTATAATCATGTTTGGAACGATTAAGACTATATTCATTTTCTTGGCGAATGTATTTGTAGTAAAGTGATTTTTCAGTTGAACTCTCCAGG CCAGAATTTATATCTGATGAATTGAGTTTGTCATGTACACTTGTATTTTTTTGTTCATAGAAATGTCTATGTTTGTTTTTAAGACATAGGAACTGATGGTGGCACGTGTGTGTGTGTGTGTGTGTGTGT
Gateways-61	5:**ATTGCACC**TCGTCCAAAGTGCGACAGGCTTTACACTTTGCATTGTACAAATATCAGACGGTCCTCTTTGCAGAACAAAC<br>3:**GGTGCAAT**GTTGAGCCGTGTCTTTAAAATGCTATTAGTTCCCTATATGAGTGAAGCCTGCGCAGAGAGGCGCGAGCTCCGCAG
Gateways-62	5:**CCAACCAT**CTATAACACCTAAGCAAAGTATCAATGTTTTCAACTGAATTCTTTAAGCAGCA<br>3:**ATGGTTGG**GGAGTTAGACTATGCCTATAACTACTAATAGTCAATAGAAATTTAATATTTGACATGTATTTGATAATTATTGACCTATGGACTCCAATTCATTATTCAAAACTATAACTGTACAAACAATGCAACAAAAAATT ATTGATTTAAGATTTCATTTTTGCTATTATTCTCTAGGGAGAACAAATAATAATTCTGCAATTTAAAACCAAACAAAAGGTCCTCAGGAGTAGCTTAAATAATGTGTCAGTGCTCTTTGGGTAAGGGATTAATCAGAACAAACA
Gateways-63A	5:**GGTCCAGA**CAGATTTTCTGTTAAAAATCCATATCTCCATGTGCTGGGGAATTGGTAACACATTTTCAGGGTTTTTAAACTTCTTGAAAGTACTTGAATTTAGACATATGAATTAAATGCCTGGAAAGTATTTAAAACAAAC CTAGTTCCTTAAATATGTGAATTTAAAATG<br>3:**TCTGGACC**TAACAGTACTGAACAAGAGCAAGCGTTCTGTTACACTCCATAAGGATATAATTGTACCTGGCAAAATTATATGTCTAGAAGCATTTGCACATCACAGCTTTTTTTCCCCCTCAGCTCTGACTGTTTGAATCCAG TTTTATTTGGAAAGCGTACATGTAATA
Gateways-63B	3:**CCCCTGAT**GTCTACATAGTGAGTGTGTGTGTGTGTGTGTGTGTGTGTGAGTGTAA
Gateways-64A	5:**GACACTCC**AGAGTGTCCTCCAAGCATGACTGGACTAAAATAGTGCGTATCACTGTGAGAATTAAGTGACAATTTGCTAAGGGCATTTTAAAAGATATAGAGCTCTTAGTTACGATAAGAACACATTGTGCTTCACGTTTAAG GGGAAAATGTGCCACAAAGGGATCAAACACTTTAAATCTTAACTTTGGTCATTCTACACAGCTAAAAATAAATCATAAAAATAATCCCTTACAGGGGCGAGGCAGTGGCGCAGTAGGTAGTGCTGTCGCCTCACAGCAAGAAGG

	TCGCTGGTTCGAACCTCGGCTCAGTTGGCGTTTCTGTGTGGAGTTTGCATGTTCTCTCTGCCTTCGCATGGGTTTCCCCTGGTTTCCCCCACAGTCCAAAGACATGCGGTACAGGTGAATTGGGTAGGCTAAATTGTCTGTATG TGTGAAAATGTGTGTGTGGATGTTTCCCAAACATGGGATGCGGCTGGGAGGGCATCCACTGCGTAAAAACCTGCTGAAAAAGTTGGTGGTTCATTCTGCTGTGGTGACCCCAGATTAATAAAGGGACTAAGCCGACAAGAAAAT GAATGAATGAACATCCCTTACACAAATGACTCTTGTCTCATAGCACCAAGGTGCTCACGGCGACCTGAGTTAGATTCCCAGCTCAAGGTCCTTTGCTAATCCTTCCCCTATCTCTGCTCTGCTTGTCTGTAAAAATCTCCACTG TCCTATCCAAATAAAGGTGAAAACCCCTAAAAAAAAA
Gateways-64B	5:**CAAAAAAT**ACATATTTTATTTTACTTTTTGGCTTAGTCCCTTTATTAACCAGGGGTCGCCACAGCTCTATGAACCGCCAACTTTTCCAGCATGTTTTTATGCAGCAGATGCTCTTCCAGCCGCAATCCATCTCTTGGAAAAT ATTTTATTTTTTATCTTGCATTCAATAAAACATTATACAAAGTTAATTTTTATTTTCAGAATAACAACAGTGGCACTGTTATCTCACAAGTACAGTAGTTTTTTGTTAGACATTCTAACAAATGAATACAAATGTTTCAAAGTA AAGACAAAAATAAAAGGAGATTAATTTTTTTTTTTTTTTTTTTT<br>3:**ATTTTTTG**TAGACATAGAACTTTACAGTAACTTATAAGTACAGCACAGAACTTTTTTACTTCTATTTTAGATAATTTAAAGAGTTTGCATATTTATTTTGTTGGTTCATGGCTATATATTTTTAATCATTTTGAACTATTCA CAGCATTTGAAAGGATATCTTATCGTCATTGTTACCTCATTTCTCACTATTTTCATGATTCTTACAATGCAGCAAATCTTAAAATCATTTGATCATAAAGAAAATAATTAACAAATTAACCCACATGCA
Gateways-64C	5:**CACACACA**CCTACTTGACACCGACTACAATGCAGTTATGCATCAAAGGCAATGTAAACCCATAATAGCATCCGCTACATTAATAAAAAAACCAC
Gateways-66A	5:**ATGAAGTC**GCAACATCGGTGCCCCGCTAACGTGAGTAATAGTGTGTTATAGTAACGTAGTAGTGTTGGAGGACACATGTTATTATCGATTGCGCTTCATAACAGGGACAATGACGGATGTATGATTATGTATACAAAAACCA GGTGGTTGTTCAACTTTGCCAGTTAATGACGTCATCTGCACGCCGAAATGTTTGGACTTAATGTTAATGATTCTGCAAATACAGCCTAGGCTTAAATAACTTTATATTACGATTTGTATTTTGTAAATTTATTACCACTTCATA TTTCCAAGTGTGGAAATTCATCTTTGACACTTTCATAAACTCAATCAAGCAACATTCCCTTTACTGTGCATTCATAACGAAACACATACAAGATAGTAGCCTACACACACGTACCCCCTTGCATGTTGTAGTTTTTAAAGATAA TGATTAATTTAACCCTCTATTATTTAATAAATCTTTCAATAACCTCTTAAAATGTGTTTTTCTTAAAAAAAAGAGTCTATATTAGGCTACTTGCCATTTTATGCCTAGTTAATACCAGTCTGGACTTCATTTTGGCTTCAAAAG TGCCCTAATTCTTTGTGGCATAGATTTAGGAAGGTGCTAAAAACATTTCACAGAGATTTT<br>3:**GACTTCAT**GATGAAATTCTCGAATTCTGACTCAAAGCGCGTAGCAGCGAAATCATTTGCATGACCCGAACATGAAGGGCGCCCGGAAGTTCACGGAGCTCGTCGCATAAGCGGAAGTTACCGACAAATTATGAGATCTTTTT TAAAATGTGTTTGCTCTTGAACTTGTTCTCGAGTATGGAAAATATTGGGCAGAAAAACAATTTGTTTATACCAATGCTAATCTCT
Gateways-66B	5:**GGCGTGAT**GAAGTTCCATGATTACTTGTTTGGGAGACCTTTTGTTCTGATCACAGACCATAAGCCTTTGTTGAAAATACTAGGTCCAAAGACTGGAGTGCCTACTCTGGCGGCTGCCAGAATGCAAAGATGGTCTTTGATTT TATCTGCATACCAGTATGAAATCCAGTACAAAAGGTCTGAACAGCATGGAAATGCTGATGCTTTATCTAGATTGCCCCGTGAAAGGGGATGATTCTGTGCATTCCAGTGCTATTTACAGAATTTCTTATTTGGAGAAATTGCCTG TTACTGCGAAAGAAATTGAACACGAAACAGAAAATGATTCTGTTTTAAAAAGAGTGAAAGAACATGTAATGAATGGATGGCCACAATACATAAAGGAAGATGCCCTAA<br>3:**ATCACGCC**AAATACTAAGCCTAAAGCCTCTTTTTCAATTTGAGAATAATTTTTCTCAGCTTTCGTCAACATCCTGGAGGCAAACGCGATTGGCCTTTCACTACCGTCCTTCATGCGGTGTGCAATTACGGCTCCTACTCCTA CTGGAGATGCATCACAAGCCAACAGCAACAGTACATTTGAATTGTAATGTGTCAAAACTTGATTTGACATTAAATGTCTTTTTGCATTTTCAAATGCAATTTGGCATTCCTTTGACCATTTCCACTCTACACCCTTATGCAACT GTTCTGTCATGGGTTGTATGAGGGTAGAAAGGTTGGGAATGAATTTGCCATAATAATTTAGCAAAGCTAGGAAAGACCTCAATTCAGTTACATTAGTGGGTACCGGGGCATTTTCAATAGCATCTGTTTTTTCCTTCATTGGAT GAATGCCCTCAGAATCAATGATATGTCCTAAATATGAGACACTACTCTGGAAAAAAGCACATTTGTCTTTTCTGACTTTAAGATTGTAGGCTTGCAGTCTCTTTAGAACTTCTTCTAAAACATCCATATGCTGTTGTGTGTCAC TTCCAGTGATCAAAATATCATCAAGGTAACAGATTACACCCTCCATACCTTGCAATACCTGATCCATGATTTTCTGAAAAATCGCAGGTGCACTTGACACCCCATACGGTAATCTGTTAAAGGTATACAACCCCTTTTGTGTGT TAATGGTCAGGTATCGTTTG
Gateways-67A	3:**GTGAAAAC**CAACCCCCAAAACCAGCTGAACGTGAGAACAAAGTTGAGGAGATAAAGTGGGGGAGAAGTACATTAACATACTCTCCTCAGGCCATGACCCAACAATAGTAAATATATTGTAGATTGAAATACAATTAATCAAT GTCTCTGTTGATTTCATCACTACAACACTGAAGTCATAAACTGTCAGATGACACCAAACATGACAAAGATATCTGAAGGAAAAACACAAGTAGATGAATTGTGTATGGTGAAAAGCACATGGTTACTCTAAGCAAATAGCAGAG ATGTGTATCAGAGCTGTGAGGAACTCTGCCTCATCAGGAGGACTCATCCCATGCTTGGAATGTCTCAGGCCCCGTTTACACTAGTGCGTTTTAGTTTTAAAACGGCATTTTAGAATGAAAAGATCCGCGTCCACACTCGCGTTT TACCCAGCGTTTCTGAACTGCTCTCCAACCACACCAAAACGCTGAAAACGCACATCACGTGACCACACAGACACTCTCGGGCAAGCGCTGGAGCCCATCTACCCAGATGAGAGCTCTGCTAGTCGGACTTCTCATCAAGCATCT CCCGCTGGATCTAATCTCACTATATTTATTAAACGTGATATTTCATTCATCTTGTTGTCTTTATCTAACAACATATTCCCTGACTTTGGTCATAGGAATCTATTACTTGTTCTCAGGTAACATGTTTTGGCTGAGCGCAAGATA AGTTAATGGTTAATGTAACCACGTAGCCTATTCTGTATATTGACTGATCTTTGCCTTTATTTCCTATAATGTATAAACTTATTGTATGTTATACTTTTATAATGGCCATTATCGATTATTAAAA
Gateways-68A	5:**CTCGTGAG**AAACATGTCAATCAAACTTACAGCACGTTCAAGTTCCAAAATAGCTTACTCGACTCCATTCCCACGCGTTCTGCTTATTAAAAGTAACATGCATAACCCAATCTGGATCCTGTTTGACCTCTATAGCACTCAAA TGAACCCAGTGGAATAATATACGCTGTTATTATTCGTGAACGCACATCACAAAGGAAGCCACCGCCGCGCGCGCGTGTATGTGGAGCAATTACGCACAGATTACGCATGGCTAAAACCACAGAAAAGATACACCAAAGCTTGAA TTAAACTCACCAGCGAAGGTAAATCCCGAAGAAAAGAAGAAGCAGATCCATGTGAGTACAACCCCGGATCTGCAGCGGACAAAGGGACTCAGGTGTAACGGCATCTTTATCACCTTCTGCTTCAAGTTTAAGTCTGTAAAATGA GTATCTGCGGTCTTGTCCAGGCTCAGGTTTGGTAATATCAGAGCGATGAGCGCATTTATAAGTCCTCTTCAACACGAATCCTCCGCTGTACCTTTTGCCATCAGCGCGTCT
Gateways-68B	3:**CTTAAGGG**GTGTTACAAATTATTCTACTTTTAAATACAAGTTAAAGAAATGGCTAAAAGCAAATCAAGTTTGTAATCATGTGTAATTCTTTTGTCTAACATTTTAGGAAATTCTTATATACATGTTTTTATCCCTTTTTGTC ATATGTAACTTTACTGTTTTTGTATGTGTAATGACGTGTTTTTTTTTATTTTAGGTTGCCTTTTAAAATCTGGCAGGGGGCAACAAATAAAAATTAGCCCTTGTGGCTAACTCTGGCTTATTTACAGTTTTACTGTTGATTAAT GAGCATTGTCCCTGTTAAATAAAATAAATTCAAATT
Gateways-69A*	5:**ACCGTGAT**GTGACCCTCAGCTCTTTAAATGTGGGGAAACTGCAGTGAGATGATGTTGCTGATGCAGTTTTAGTTTGATCTCTTGACATCTATGCAAGCATGTTATTTCCTTTAATAATCGTAATATGTTTTAAAATACTATG TAGCAGTGGAGTGCAAGTCCTTTTTTTCTGCAGCACCTGATCAAGAAGTTTTTCTATTCATTTATTTTTCCAATAAGGTTTATAGAAATATGCTTAAATTTCTTCATCATTTATTCACCCTCAAGTGGTGCTAAACTTTTATGA CTTTCTTTCATGTGCTGAACACAAAAGCTGAAATACTGACTACTGTAttttctcaaatgaaagtaaaaagtctggCCTCAAACATGTGTTAAGTAAAAAGTATCAGTACAATTTCATATTTAATTTACACTGGTAATAGCCT CACATTATATTGATACATTTATAGAATAACCTGACATTTCTATTTTTTATCTAAAACATTGTGTTGCCTTATCTTGTGTAGCCAAGCAAAATTACTCTTTTTTTTTAATCAACACTCATAAAAGTCCTGTTTTTGTTACAGCAT GTAGTCTTAAATTTctataaataataaatgtcttcaatttAGCACTTCATTTTGTCCCAAACGTATACAAGTTTGTCTTTTTTTTT<br>3:**ATCACGGT**AGATGCCAATGCCTCTGATCTGGTCGAGCTCTTGGATCCGATGCTTGAAATTCATAAGGTGTGATCCGTTTACTGTCACTTGAAATTCAGTGTTGGTGCATAAGATTTGCATCTGAAATTAAAAACAGATGCTA TCAATTGCATATACAAACTTAGCAAACATGGCAGATGTCAAATGCAAAACCTTTCAAAAGAGCTTTGTCTCAAAATATATTTGTTTTATTGATGCTGTTTCAGGAATTCTTTTACCTCTGTTTCCACACTGACATCCAGCTATC TGATATTGTTTCCCATAGTAATTGTGTTTGATTCACAAAAGTGTTCAAGTGTGTGAGCTTTGTTTGACTACAGTTGAACCACCGAAGTAACAAGGAATGTTTTGACAATGTTTGGCTCCTTTTCTGAACTTGAGGGAGCTTTCG AATTTCATACAAAATATCTTAATTTGTGTTTTAAATAGGAATGAAGATCTCAGCATTGGAATGACATACTGGTGGGTGATTAATTACAGACTTTTTACTTTTGGGTCAAAATAAAACTAGTAAAATATAAAATAAAAGAAATGT TTGGTGTCATACTCACCTCAAAAGGCATTCCTCGGAAAAAAATGGAAAGGAGGAACCCCACGCTCTTCTTTGCCCCAGATGGATCCAATCAGACTGTTACACCAATCCTTTGAATTTCATTCTTCACTGAAG
Gateways-69B-1	5:**GTGCCGGC**ATGCGGAGGCACCACAGCGTGCGTTTTAACTTCAAAGAGGTTGATGGTAAGCTCCTGGGGATGTCCCGCCTGGACTTCTCCAGGAAGCTGATCCAGAAAGCCTTAAATTTTAAACCAACCGATTTGAATTGCTT AATGACTCTGCCCCTGAACAAAGGGTTTGACCTGAGTTTTAAATCTGCTTCCCTGCTACAAGATTTTTGGCAAAAGTTTGAAAGTCAAAAAACCCAGTTCTCCATTTTTTCTGTGGAAAAGCTTTCTGACAATTCACTTAAAAC AGTGGTTGTCAGGATGTTTAATGAAACAGTGAGTGGAGAGGATATAGCAACATGGCTGGGTAGATTTTGCACGGTTCGAAGCCAACCGGTCAAGGTGTTGGACGAGGACGGCATTTGGAATTGCGCTTGGAGAGTTCCCATTAA ACAATGGGAAGATCCAAGCGGATTCCAGGGCCTTCGTCAAATACCTTCAATGATTGTGTTGGGAGAAAACCGAGGCTACATTCACTACCAGGGAATGCCAAAGCTCTGCAGGAAATGTGGCGGGATGGGGCATTTGGCTGAATC TTGCCAAG

Gateways-69B-2*	5:**CTCCCTAT**ATTTGTTTACCATGCAACTCTAAATATTTGCTCTGAAATTGCATAGTCAGTTTGGCTTGCACGCCGCCGGACAAGCACTAGTCGCTTCTAACACATGAGAGAGGTTTCTTTTGCGGTGCTCGTGTTTAAGGAGGCATGGCCCTGGGCGGCAGGGGAGGGACTGTGATTCAAAGAGTTATGCTTAGCTGTTAGCATTGTGGACGATCACCTACTGCACCTTTAAGCATTAACTCTACACTAATAAACATTAGTAAGCAGTTTATAACTGCAGCTACAAATGCTGTATTCTTGACTTTTCACATTTATAATGTGCCTAATGGTTGTATTTTCATATTTTTTGCACTAAAACAACGCTAAAAAAGAAAACACATTCATTATTTATTTGTTCTTTTTTTGCTTGCAGTGTGTGAACTGAGGCATTATTATTGCCTTTAGTTGATGAGTAACTAAACTTCTCTTGATGACCAACTAAACTTCTCTGA<br>3:**ATAGGGAG**ACTAGGCGGAATAGTGTATTACTTGTGTTAGTTGTTAAACTAATTAAAATCTACATCATCAATGCTGTAAAAGGTCCCTTACAAAACTGAAAATAGTCTTATCAATCTTTCACCGGAAGATTTGAGTGGCTGAACAACACTTCTCTGTAGATATAAACCATTTGTAACAACAACATCTGCTCTGCGTGAAGCTAAAACAATTCTAAAACAGAACATTATCTGTCAAAGAGAAATCCTTCATATATTGTGTCATCCTTTCTCCAGCGTACAAAGATAACTGCAATATTGATTGAGGTTTTTAAAAAGTTTTGATTCAGCAGGTTTTTACAGATCGCATGCGTGTTCTCTTTCCCTCTCTCGTGAACGCGCGGCACAAACAGTGGTCGGCggagctgtgtacaaaaggcTGCACAGTTGTTCAGATATGCATTTGCTGACAGACAGTTTCGCCTACTTCTCTGAAATATGAGAGATGTCGGCCAGAATCTTTTTAATTAGATGAACATTTTTTAGTTTTATGCCTTACCAAAAATATAAAAATACATTTAAACACATTAAGATCATTTACTGTAATCATTACTATTAGACTGTGAAGAGACTTACAACCAGCAC
Gateways-70	3:**GCTGCAGT**TCACTTAATGGCCCAAGGTGTCGCTAAAAACAATGGATTCTGAACTCTACACAAAACCTATTTTTGAATTTCTTTCAAAAATAACATGCACAATAATCCTATAGATAAATTGTTTAACGTATTATCTATAAAATGCATTAAAAAGGAATATGGCAAATTTTAATTTTACCCCATCCCATTATTTTACCTTTATTTCAGCAAAAATACATAATTACCATTCACACCACACATACTTCATTCTCCCCGCATGCTTTTTTAAAATTTAATATTAAATGTGTGTTTGCA
Gateways-71A	5:**TGGATGAA**TCAGCATTTGTGAATGAATCACTTGAACGAATGATTCATGATTCGAGATTTTAATAATTTATTAATCAAACAGTTATTAGAACAATTAATCAATTAATTGTCCATTATGTCACAGATTAATATGTAAACGTTGATTATTCAGGATAAAATATTTAGTTGTAAATAAAACAAGAAAATCACTTAATCCAATTAAATCAACAACTCATCAACCTCTCTCTCTCTTTCTCTCTCTCCTTCTGTCTCTCTCCACATTGGATTCAAAGTTATTTTGTTCTCAAAGAACTGAAGTATGATGGGAATTATTTCGG<br>3:**TTCATCCA**GTAATCAAACAAGTGAAGACTTTATGGGTGAGCTGTTGGGAGAATCGGATTTTTCAGTTTTTCCAAATCAATGAATTGTCTTCTCAGAGATTTGAGGAAAATTATAACCTGTATGTAAACACAAACATATCTGCATTCTCAATCTATGCAGAAATTGCACACACACAAATCTCTAAACACACACAAAGAACATAAACCCACACTCAAGCCAGCATGAAATATTCAGCACACTGCATTGTGAGGACCACAGTTCACTGGATCAGACTTTAAATGGAGGAATTCGCTGCTCTCTCTCTCTCTTTCTTATTTCCCCTCTTTTTGAACAGCTCTCCACTGTTTCTCCCCTTCAGTTCTACACTTTAGTCTTTTATACTCTGTGCATGCAACTCGCCTCTTCTGCTCTCCCCTTCCCTCAGTTCTGTCACTATACATCCTTTCTTCTCCACCGTCTCCCTCCCATTTCTCTCCTCCGTTCTTTCTCTCTCTCCTTCCCCAGAGTCTGACAGCAGGAGACTATTACACTCACAGACGGAATCTCTCTCCTC
Gateways-71B	3:**GATGTATT**TAGCAGGAGAGCTCTTCTTAATGCTCTTCGTTAAACAAAAGTAGCCGTATAGGATTTGTGTCAGTCAGCGATATGAAAAGAATTGATGAGAAACGGGCTGAAAGGGAAAATGCAGCTTTTAGCCGCGGAGCGAGCGCGGGGCTATTTTCTGTTTGGCTGGCTTTTATAAAGCTGCACTTTTATTTGGCCTGAGCTGCGGGAGGGCCTGTGTGACGCGCCGGAGAAAGAAACGAGCTGTGATGATGACGAGCCGTCAGTGTTTTCAGTGCTTTCAGGATGACGAGGAGAGACGGAGAGCTGAAATCTGACCTCCTCTTATCCACATTCTCTAACTGAGAGAGAAGTGTTTGGCTTACAGAGAAACTCTCAGCGATCGGCTCTGAAAATACATCTCCTTTTTCTTTAGTTTGACGGAAAATAAATGAATATTATTCATCTATATTTCTTTAACGTGCATCAAAACCTTTTAGTTTTGAAGCTGTTGTGCATTTCTGCTGATGTAAAAGTGGGCAGGAAAATCTGCACAAGTACACTTCATTGAACATGTGCATCGGGCCACTTTATTAGGGTACACCTGACTAGTGCCGGGTTGAACCCCCTTTG
Gateways-71D	3:**AGTCTCCT**CATAAAGCCGGAGTATCAGCGCGCTGCTACATGGAAAACATATCATTTGATTTGCGCCTCCTAATTGGTTCTCGGGATATATCAGCTTTAGCTGTCAAAGGCAATTGGCGTTTTGCCAACAAACCGTTATTTCTGAATGCGCTGACGTTGCGATTTTTGGATGATTAGAAGCAAATCTATTTGTTGATGGTGTATTACATGCCTAAAGCATTCACTCAATGTCATTAGCCCATTTTTGGCGGAAGTGCCATTTAGCGGCTTTTGCAACTGAACTCCTCGTGTGTGTGTGTGTGTGTGTGTGTGTGTGTGTGTGTGTGTGT
Gateways-71E	5:**CTGTTCAC**CCCCCAAAATAGAAAATTCTGTCATTTATTCTTCCTCAACCTATTCCAAACCTGTTTAATTGAGGTAAAGTTGGTTTTAAATTCGCACATTCGTTCAGTGACAACTTGTGACATGCTCCCTATATTGTTTACCATGGTAATTCACGTATGACTTCAAAACAACATTAACACCATTTTTTGACTGTAAACAATGTTGTACTTTGATAATCATTGGCTGATAATATGATTTCACTATTTAAAATTTGCAAATAATATTTTTG
Gateways-72*	5:**CATACATA**TACATATATATATAGGGATATAACAGATCACGGGTGATCTGCTTTTCGAACGAATGACAACCCACGATTCGGAACACACGTGAGACACAGATTAAAAcataTTTTTACTTGTAGATTAATCCTAAATTTGCAATGATCGCAGAGAGATTGTGTCATTCAAATCTCATGTATGAAAGCATTTAGGCTTTCATGTAAAATATAATGATGATAGAAGAAGTTGTGGTGGGTTATTTGGAATATGGTGAGTTTCTTAGGTTAAAGGTGTTTTCTGTCACTACTTGTTTAGCCTGCAATAATGCATTCAGTAAGCTGCAAGATTAATCATTAAAAGATTGTGATTTTAACACCCACACAGCATTAATTATAAATGATGGTGATTTAAAGAGCCCCTATTATGGGTTTTTGAGAATGACCTCTAATGTAGTGTGTAGCACAGCTCTAAGTGAAGTGAAATATCCAGCTAAGGCTTAAATCTGAAAGTGGACAATGTTTAAAACTATTGATTCATTTATAAAAGAGTCGGTTTGGGAACAAATGAATCAC<br>3:**TATGTATG**TATGAATGTGATAGTGATCATCATATTAATTGCAATTAAATATGTTTCCCCAAATATCTTTTGCTCATAATAAAAATAATAGCCCTTACCCGTAATCTAGTGTTTAGGATTCAGCTCTCTCATGTCACAAACTTCCTTTAGAGGGCAGTTGTGAGCTAATGGTCAGTGAGTTGGATTTGTAACCTAAAGGTTGTTGCTTCAATTCTTGGTCCCGGCAAGAATTAAAGACAAGGGCAAACTAATGAACTGCCTTTCATCTCCACCTTCGATACACTGCTCACTCTCTGTGTTTGTGTCTTTTTAACTCACTTAAAATGTGTGTGCACTTGGATGGGTTAAAAGCAGAGGACCCATTTTAAGTATGGGTAAAACGATATCTTCACTTCACTCACAATTAACAGAGCATTGGTAGAATTTTAGTCCATGTTAAGAATAAATGCATAATTATAGTCAAAAAAAACTATTTGTAGACAACAAACTGATCTGAATAGTTCAAGCATGTCTTTACCTTACAATAATGGATTTGGATAATGGATTCTACACATGAGATTTTCTATTAGCAAAGTGATTTATGTACATATTATTTTACATGTAAAGGCAAGGCAAGGCAAGTTTATTTATATAGCACATTTCATACACAG
Gateways-73	5:**TTCATCTT**AAAACTATAAATAAATAAATTCTCAAAAAATTGGTTGTCATTAGCATGATACTGATATCTGGGTTACAATGCAATATTGTTTTTTTTTT
Gateways-74	3:**TCTGCAAG**TCAATGATAAACGTTCTGAAAGTGCCGTTGCAAAGTTGCACCGTAGCTGTTTCCTTCCCGCTGCAGCCGAAAATTATGTCCGTCTTCCCCAAACAACAGAGTCATACATTATGGAAAGTATTCCGAGGGAACAGAGCGGACTGGAAAACGTTGACTTGGCAGAATGTCTTTGATATTTGCTCCCGGGACCATTTGTCTGACTGCTGCAATGTACCCATCACTGTGTGAGATCCAACAACTCTACAGTATGTGACGAAAAAAAAAAAAAAAAA
Gateways-75B	5:**GTTATATT**TCAGTTTATGTGTATTATTTAAAGTAGCCCACAGGCTGTTTGTTTGGTGACACATTAAAACTTATTTTAATTCACATTTCATTTAAAGGCTCATGTTTAGGCCTAGTGGCTATTATTAAATGTCTATAAACAATGATTTCTTTTGTTTTCAATTGATGTTTAGTTTGTGGTCTTGTCCTTCATCCTGAACAGGTTTACACTTTTCAACAATATTTCTCTTAAACCTTTTTTGTATCTTCCTGGTACTTGTTTTAGTTAAAATAAGTTGAAATTATTATATGTGAGACTTACAACACATACAACACAAGTATTTCAAAGCTAAGGTAAAACTATTTTGTTTGGATTGTTTTTGGATTGACTTTTTCCCTAAGGCCTACCAAACTATCAAAGTGTCACATTGTGATATATTTGTTTTAATTTATTAAGTTGTAATTTGTGTGAACCTTAATCTTTTATTTTATTAATAAATATTCATCAATGCTCAATGCCATTTTCTTCCCATTGTTCCATAATCCTATCATTTAAAAGTTATGTTGGCAAAGCGGCTTCTCACCCCGTCT<br>3:**AATATAAC**TAACACACTGAAACATTGTATGTATGGGCAATTATACCTGACCATCTCGGTTGTCATTTTCAGCTTAGTCCCTTATTTATCAGGGGTCAACACAGTGGATATTGTCGGCTATATATCGATATATCGGCATAGTGTTGATCTTACCCATTCCTCTGCAGGCCGCTCATCTTACAAATACCACCAGTTTTCTTAAGACACCTAATAATAGATCAACAGAGGCCTCAACTATGTTTACTATGACCATTTTAAAAATAAAAAAGCTTCAGATATTGTGGATAGGGTTGCCACCCGTCCCTTAAAATATGTAATAGTCCTGTATTTAAGAATGAAATTGCGCGTCCTGTTTTGAATCAATACGGGACGGGTTTTGTCTCATAATTTTATAATTCTTTAAAAGTAGCTTTTCATGCAAATCATCCCACACGCATTTACTGAAGATACCTCATTTCCTACTTTTGATTGGGTAGTACCTGATGTCATCGTTAGTTTAATTTTAAATTAGGTTCATTGGTATTTTCTCATCTCCTCTAGGTTACAGCAGCTGAGTTAAGGCAAGTGTACCAAACTATTAAACATAACGTCAGCTAAATCAGTGCTGGAGAGTTGTAAAAAAGAAAGACTAACTGCATCTGTTTACACAGATTGCAATAAAAGACAGTTTATTTTAATTTCCAAGTGGTTATACTTTCGAGAAAACATATTTTAAGTTGCGATTTTACCACCACTGGGTCTAGGCCCCCAAAGAATAATGGCCCCCGCTGCACCTA
Gateways-76	5:**TTCTCGCG**TCAACGCACCAGAGGCTGCTATCTGACTGACAATTAATTGACTATGCAACCGGCCAATCGGCTGACCCACCCTCCTCCTTCCCTGAACCCAACCAGTTTTACCGATTGACCCGCCCGCCCGCTCACTTCCCTAAACCCGAGCAACAATGTAAAAAAACCCTCGTCTGTTTTTTTTTT

	3:**CGCGAGAA**CAGCGTGGGCGCGAACGGCACTCGCCAGATACGTTCGAGATACGAAAAAGAGAACACAGCGGCCTCTCGCGGATTCGTGAAAACAAAAACTGCACAAATACGTACCTCCCGGGACGTATTTCGCGGTCTCCAGA<br>AACATCCGCTGGACTACGTTTTTAGAATGACCCTGGGTTGGACTTTATATATCGAAAACACATTATTTTACACAAATATGAGCACACATTTAACACATTTATTATACCAATGATGCAGCATGGGGAGATTCCGGAGTTTGCAGT<br>AGCTGAAATGCCAACACAACAGCACTGGTCTGCAGTGGTGTAAAGTAACTAATTACAAATACTTAAACTACTGTAATTGAGTAGTTTTTCTCAGGAATTGTAATTTACT
Gateways-77	5:**CTTAATGG**AAACTGCAGCTTTGTCATATCTAAAGATAACAACTGATCTGATGTCAGCTGAAAACAATGGCCGCTCATTAATCTTGGTGACAGGAGGTGTAGACAATATGTTCTAGCCTGGGCGTCTCAAATCATGTAGTATG<br>AGGCTCGGGCCGGTTACAGAATGTTGATTTTTTTTT<br>3:**CCATTAAG**TGTGTCTGGCTGCAACATCAGCCCTCTCAATTGTCCCTATTGTGGTAGTTGTGGATGTGCAGATGTGTGTCCTGAATCCACAATCAGCCCTGAGAGAGGAAAGAAAGTCCATGATCAAAACAGAAAGTATGTTG<br>AAGTCAGAATTATAAGCCCTCATGTATATTTTCCCCCCATTTTCTGTGTAGTGGAGAGGAGATTTCTTCAACACATTTTTAAACATAATAGTTTAAATAACTCATTTCTAATAACTAATTTATTTTATCTTTGCCATGATGACA<br>GTAAATACTATTTGACTAGATATTTTTAAGACACTTCTATACAGCTTCAAGTGACATTGAAAGGCTTCACTAGGTTAGTTAGGGTAAATAGGCAAGTTTTTGCATATCGATGGTTTGTTCTGTAGACCAGCGGTTCCCAACAGA<br>GTCCCGGCCCACAAGGGGGCCTCAGCGAGCTCTGTAGAGGGTCGCATCATATTTGAAAACAAATTTAGCAGTAGACAATTAGGAGAATGATATTGTTGCCTTAGTTCATTTTATCCCCTGGCTGACCACAAATTTGTAATTCAT<br>CCTTCAGAAAAGAGTCCCACTGATTTGCCTTTAGCGGCTAATACTAATGACCCAAATATACCGGACCCATGTGAGAAAGCTCCAAACATTT
Gateways-78	5:**GATTCCGG**TTTCTGGACTTCAAGGACTCGGTTCTTAGAAATGATTCCTCACAGTTGCTTTGATTCTTTTCATTTATATTTCATTCTCATATTCTGACAGAAGTAAGTAAACAGTAAAAAGAAGCTCGTTTCAAGTACATACA<br>GCTGAAGTCCAAATTATTAGCTAAGATGCTAATGGTGACCCCTAGTTTAAAAGTGTTTATTTTTATTTTAAATTTGATGTGTAGAGTTTTGTAATTGAGTAAATTCCCCTTCTGAGTGTGATTCATTCAGTTGAACTTCGTTAA<br>ACACAGACTTCATTCAGTCCAGATTTTTCTAAATGTTTTGTGAATATTTGTCTCAGTCAATTGGAAGCC
Gateways-79A	3:**ATTCTGAC**ACACAAACATCTTCAAAATCCACTCCCCACAATCTCTTAATTACATCACTTCCAATCACAGGCTTAAAAAGATGCGATCTGAGTTAATAGATGAGGCTATTTACTGCTCATACTTATTTATAGCTGTGCATAAG<br>CTGTCCAGTGTTCGTCTGAATGACAGGCCCAGATTATGACCATCGGTAAAAAAAAAGAAAAGTCATGAATACAGAAACATGCGAACGGATAAAGATTCGCAGGTCAAAGGAAAATCAATGAGTGACCTGGCTGGAAAATAACCC<br>ACTGACCAATGAAGTGCAGCATTAGTGAAGTGGCACTGTTATTCCCAATGCATAAAATCTTTGTTTACCATCGAGAGAAATAAATCAATAAGTCAAGTCCATTCCTCCCCACAGTGAGAGGGACACATGTTGGGTCTGTTTCTA<br>TAGTGAGCTGCCTGCCTAGAAAACTAGCTATAACACACAACATGTTGGTTTTGAATGAGCTCT
Gateways-79B	3:**ACTATTGA**CCACTGAGAGTGTGTGTAATATAATAACAGCACATCAAAGCATAAGCACAGCAGATCAATGAGCCGACCATGCATCGCCCTCTAGCGGTCTAAGTTTGATATTACAATAACACAGCACGGTTAGCATTAGTGAC<br>TGCAGCATAATGTTGTGTGTGTGTGTGTGTGTGTCTGTG
Gateways-79C*	5:**CAGGAAAG**TGatatcgcATATCGCAGTCCGAGGAAATTAGATCAAAAAAGAAAAAGCGTCCCGCTGCATATTAACTCATCAGTACTTTCAATCCATCCTCATCAGCAGAGTTGAATCACCACAGACAGACTGGCGACACAAA<br>GCCTGTTTAAGTGTATTTAGTATGCGCAGTCATGTCTTTGGTGGATAGATATATTGAAACCGACGTACGGACATGCAATGAGACCTGTGCTCAATTCTGTGGCCTCGTTTGTCCGATCGTTATAAGTGGGTCAGTCACGTGGTG<br>TCCCTTTTGGCTGGGCTATAATGACAGTACAGGGTTATAAAGCAATTATAAAAGAGGACCTGAAGGGTTTTTCACATGTATCAGCATAAAACCTTAATAGGACCTGGATTGTTCTCATAAAATGACACCTGCTCTAAAACAATA<br>TTTCTTAACCTATTGGCACTTTTATGCCTAGGTAAATCTTTTTAATGTAAGCTTATATACTTGTAATGCCAATTATGTTACTTGAAGGAGTTTTTATAAGACTGCCATGGCTGTCATGTATATGGCTGAGATCGTATCCAACTG<br>ATGTTAACAACTGCCATTAATTGTAAATCACTAAGTTATGTAATTATAAATGCAAAGGTGACTATGTTTGGAATTTCATCATATACATCATAATCGGTCTTTGCCTTTGTTATGACAACTTGATATTACCAGGATAACATTGTC<br>ATAAATATGTCATAAACATGATTGTCATGACTTCTTAATAATG<br>3:**CTTTCCTG**TCGAAAATCTCATCACTTTGCAGGGCTCCTTCTGAGCAAGGGGTAGAAATGACTAAAACAGACATTTGCTCAGAGGAGATTCAATTATTTGGTGTGACTGGAGCATGCTTAACAAGACTCTGTCCTTTCAAGAA<br>GAAAATCGAGCAATAAAAAGTTGTAAAAAGCCTCATCTATCTTTGGAATAACTAGGGCTGTTGACTTCAGGGAACAGAGCTATGACGCCTACTAATGCATTTCCATTCCCAG
Gateways-79D	5:**TTTACCAT**TAACAAAGCATTTGTTAGTGTAATTATAAACCTTATTATAGTCTGAACTCCAGCATTATAATGTTACATTTGTTCATTAATGGTTCTTGTAAGCACTAATTTGTTAACTAACGTATTAATTAACACTGTAACAG<br>ACGTAAATAAGCGTTATCTTACATTTCTTTATGCTGAAGGATCATTTTATTTCTATTGTTTATGGGGGACTTTTATTTTGACAAGAAATCGTTTTTTCAGAGCTAAAAAGTTATGAGAGAACAGTATGGATAGCGGGCATTTCA<br>TGACGGTTTAACAGAGCTCGTATTAAAAAGGACGGTTTATAAGTTTATTCCTTGCTGCCAAATATGTTGAGCTCCTTTTAAAGGAAGTGGAGCAAATGACGTAGGCTCAACGTAGCCATTGTGTAACGAAACGATTGAGCGCAT<br>CACCACATTAATATTCACGAGAAGGATTACAAGCTGCTAC
Gateways-80A	5:**ATTTCTGC**TCAGAAATTAGAACGTAACTAAAAACTTTTTAACTTTTATTTAAGATTTACAATGCATATTCAATTGGAACCCCCCTTGTTTAGCAAACAAAATACACTCATCGATCACATTATTAGGTAGACCTTACTAGTAC<br>CAGGTTGGAGACCATTTTGCCTGTAAAACTGCCTCAATCCTTCGTGGCACAGATTCAACAAGGTACAGGGAATATTCCTCTGAGGTTTTGGTCCATATTGTCATGATAGCATTACGCAGTTGCAGCAAATTTGTCGGCTGCAAA<br>TCTATGATGCGAATCTCCCGTTCCACCACATCCCAAAGGTGCTCTATTGGATGGAGATCTGGTGACTGGGGAGGCCATTTGA<br>3:**GCAGAAAT**AGCAAAAAAATTCTAGTAGATTCTACCTGGACCTAGTCATTTGCCAATAAAATTTGAATTTATTTTTATGATACCACAATTATTTTTATTATAACAGTTAGGTAAACCTACAATACAGTTTTACAAAGTCATTT<br>AGATATAAATTTTAGACTACTGAATCTGAAAGTGTTCTTTAAGGCTGTTAGGAGGTCATATATCTGTAGGGTACCATATATTAGGTTAATTTAGTTTGAGCAGTGATCCTGAAACAGGAAAAGGCTTCAATTGTCAGCAGAGTC<br>AACAAGCTAGAAACTATGACAGAAAGGATCTGTCCACTCATGCACAATTTAGGCAG
Gateways-80E	5:**ATCAACAA**ACACCCGTCGTGCGATCCTGCCATGACGCAAACTTTACAAATAAACCCTTATCTGGCGAGAAAGATACCAACCTGACAAGAATTTCCCCTTCTAATGTGTTTTCTCTGTTTTTTTTTT<br>3:**TTGTTGAT**ACACACTGAAATTGGCTTTGTAAATCGACGCCCCCCTAAAATGATGCCTTAGGTGGTCGCAGTTGCTGTGGTGTTCAGAATGTTTTTAAGTGGTTTTCTCTGCT
Gateways-81A-1	5:**CTCAAGCG**TAAAACGAGACAAAAAGCTGTTTACTCGCACACGCCCGTCAAAATCGGCAGGCTAATGCAGAAGCTCCATTGAATATAATGGGGTAAAATAAATGCTCATATTATAAAGACATGGCAGGGGAAATGTAATTTAT<br>TGCAGTGCTTCTTGTACAATCTGAAACCCACTTTAAATCGGATATCACTCAGCCAGTGGAGATCGCTGATTTTTAAAGCAAACAGACCTTAAACGCACCGGATTTTGCATTGGCATTGCATTGGCCGGAAGCGCTTAACCCAGG<br>TTACTGGCAAATTAAAAGTCCTATTAGCATGCTTCGGCATATACCCCATTAAACAAGTGTTGCTCTTCAGTGATTTTTGAT<br>3:**CGCTTGAG**CAACCAAATAAATGCTAAAGTTTATTTAACTGAATGTATTTTAATGACATTAACTAATCAATGCTGTATAAGGAACCGTATAAAATGGAAAAAAGTTCACAATAACAGGTCGCCGGAAGTGTATCAGCATGGGA<br>ACAGCACTAGCTCGGCATATACCCTATACGCATGCGAGCTGTGACTGAATGC
Gateways-81A-2	5:**CTATACTG**CTATTTGCTAATGCAAAATGAAACAGCAAAAAATGTATTGTAGGTCTATTTTAATGGGTAAAATACAATCGCCTACATGGCGATTTTACAAAACAATTTTATTTTAACTAATGTAAGCTCATGGCCCTAAAAAG<br>GAAAATGTAACCTGCAAACAAAATTTAGGCTGAATAAATAAAATGATGGGTAATAGCCTAATAGTTTATAGTGCTGTTTTTACTACTGCCAACCGAATTTCACGGGGAAAAAAAACACTTATGATAGCCTAATATTTTTTTGTT<br>CTGTTTTAATATTATAATAAAA
Gateways-81B	5:**CTCAGCAG**GGGTCCAGCTTTCATCCAGATCATAGAGCCGGCAGTAAAACGATTCAAGACATGCAGGGACTTTACTCTCTTTACTCTGTTTTTTTGCATAACATTTAAATCATTGCAAACTAAATTGGAAAGATGTGTCTCAA<br>TCGTGGAGTGATAGTTGACCCAAAAATGAAACTGACTACTAACATTAGCTATGTTTCCATTCAAAAATGTGAATTAAATTTATGTGCAAATCTGCAATAATGTGTACAATATTTGCATCCAATGAGTCAAAGACAAAAATAGTC<br>ACTGATAAAATGGCATCGAATGTCACAAAGCAAAAAGGAAATTGTCATGGTAGGTGAAGCCACTGTGGGCCTTTTTTCTTTAATAAAAGAGCTGTGAAGCTGTGAATAAAACGCAATGAACGCTGAGCACGTTTACATGGACAT<br>CAATTGACATAAATACAATTAAGACAATACTCTGATTGGGAGTCTATCATGTAATCAGACATTTTTTTATTAACTTAATCTGACTAATGTCATAATCGACCTTAACAGAAATCGAATTAAGACATGTGGAGTATGCTGATTTTA<br>TTGAAGTGCAGCACAGAAATGTGAACACCTTAATCAAAAACTATTACCATCGTGTAGGATTTTC

	3:**CTGCTGAG**ACAATGAGATTCAGATCTTTAACTCACACACATCAGCTCCCATCCGTCCCCACTCTAATTATAGGCAAGAGCTCAGTGACATCCTCTGATCAGGACCTCCATCATGCCGGACACACATATTTCTTTCTGTTTAA<br>CTTTAGTCGTTCCTTCACAGAGCTAAATATTATCCCGTGAAGCACCACTGCACTGTTTCTGCTGTCTAATCCACAGGTATTAGGACTCTGTTGATTTAAATGTTAAGTATACATTCGAAGCAATGTCAATACGAGCATTTCAGA<br>TGGTGTAGACATGTTTAGAGTGAGCTACAAGTTGTCATTTCACAGACAAGTTTATCCTGAGCTATTTACAG
Gateways-81D*	5:**CCGCAGAT**AACCAGGTTTCTGTAAGGCAGAAAATGCAGCAGTTTCTTGTTTCCCATTGGAAGGAAATGCTTGCTCGTAGCTCGCAAAGTTTGTTGTCCAGTGACTGAAGGTTAGCCAGTAAAATGGTGGGGAGTAGTGGAGT<br>CTCAGTCTGATGAGGACACCGGCTCTCTTTCCTCTTTTCCGGCTGCGTTTTCTTCGTCGCTTTGGTTGTGTGACTCAAACAAAGGGCTCTGTTGCTGTGTTTGTAAACAGAGCATTGTTAAAAAACTCAAAGTCCGGTCTTCGT<br>TGTGCAACGAGGGAGCTAATTTCCCAAAGTGTGAGTCTGTCGTAGGTAATGAAAGTGTTTACATCCAGCATGAAAACTACAAAAATACAAGTAAAACACACAAAAC<br>3:**ATCTGCGG**AGGTACCAGACACAGCTGTTGAACTTTCGGGGTTCTCCGCGCACCGCATGGATAGAACGAAAGAACTCAGAGGGAAAACCAAAGGAGGAGGTGTGTGTTTCTTTATTAACAACTCATGGTGTGATGAGAGGAAC<br>ATACACCTAGTTAGATCATTTTGTTCTCCTGATCTGGAATATCTTATGCTTCAATGTTGGCCATACTGGCTACTGAGGGAGTTCACAGCTGTTATAATTTTGGCTGTCTACAATTCCACTCAAGCCAACACCGAGCAGACGGTC<br>AAGGAACTGTACGGGAGCACGAAACCGCACACCCGGAGGTCGCCAATCTCAGGAAAATCGCTCCAAAATACTATCAACGCATTACCACAAACACGCATGGTGACCAGATTTTGGACCATTGTCGTTGCCCGTTCCGGGACGCAC<br>ACAAATCCCTCCCCTGCCCACCGTTTGGCAAATCagatcactcttctgttctgctcttgcctgcttagaggcagaaactgaaacacgaaccaccaaccgtcacgacaattcagagctggacagaccaatcggataccatacttc<br>aaaactgttttaatcatgtggacTGGGATATTTTCTGGGCAGCGTGTGACGACGACATTGACGTGTATGCAGACACAGTTACATACTTCATCAGGAAATGCATAGAAGACGTGGTTCCAACAAAAA
Gateways-82	5:**GCGCTACC**AAGCCGACCCATCACAGAGCTTGCGCTACGCGTCGTTGCGACGTGTAGATAGGTGAACGTCAGCAACGGCGACGGCTACTGCGAAGGGCTATGCGTCAGCGCTGTAGCATACGTCAGCGTTTGACGCAAAAGTA<br>TAAATCAGCCTTAAA
Gateways-83A	5:**TAAATGTT**TGAACCCACTTGAGGGTCAGTAAACAGTGAGTAATTGTATTTTTTAGTGAACTATCCCTTTAAGATAAACCTGCCTGTCA<br>3:**AACATTTA**TATGTTTATTTATTCTCCTAAACACAAAATAAGATGTTTTGAAGAAAGCTGTAACCATTGATTACCACTGTAGGAAAAACA
Gateways-84A*	5:**CCCACACA**cacactgaCATTTTCATTTTTGGGTGAACTATCCTTTTAATTAAGTATTTTTGCCTCACTAGTGCACTTAAATAAATGCTTTAAACATCTGTACATTATCAAAAAATTAGTATTTTTAAAGTTATGTTTCTTTG<br>CACAGATCTATACACAGTTTGTGTTCATTTATTTTTGTTATACACTATACTTTAGTTTTTTTGTGCCATGTTTGCTCAGTGGCTAGCACTGTCACCTCACAGCAAAAAGGTTGCTGGTTTCCGAGTCCTGAGTTGGGATTTCTG<br>TGTGGAGTTTGCATGTTCTCCTCTTGTTGGTGTGTATTTCCTCCGGGTGTTCGGTTTCCCTCACAATCCAAAGACATGCACTGTTGGTGAATTGGATTAACTAAATTGGCTGTAGTGAGTGTGTGTGTATGTGAGAGTGTATGG<br>GTGTTTCCCAGTACTGGGTTGTGGTTGGAAGGGCATCCGCTGCTTAAAACATGCTGGAATATGCTG<br>3:**TGTGTGGG**TGTGGTCACATGATGCGTGTGGACGGAGGGCTGTTCAGAAATGCTACGTTGGTGAAACGCCAGTGTGGATGTGGATCGTTTCCGTTCTAAAATGCCATTTTAAATTTAAGACGTACATTATTGGAGTAAACGCG<br>GTCCAGAGTGTGTATTTTTCGCAATCGGGTTGCTACGACAAGGGCGGGGTGGAGGATCGCAATGCCGGCTCAGCATTGTGATATCTATTGGCCATCGACGATGGCATCGTCTATGGACCCAACCCTACTTCACGGCCAATCGCA<br>GTAAATTCTGTTGAAACCGTGCTCAACA
Gateways-84B	5:**ATTATTCC**AGTTATATATACCAGACCTGAAAAGCATCT<br>3:**GGAATAAT**TAACTCCAATCCATTAAATTATCTGATAATCCATAGTCTTTTTTTTGCATTTACA
Gateways-85	5:**TTAAAGAG**GGTGACCTATATTTGGTTTGGATTGGGAGGTTGAAATGTTTATTTGTGGTGGGATTTTTTTTTCCAAATACTGAAGGAAAAGTTTGGCATGGGGGCACCAGATGTGCAAATTGAGGACGGAAAGGGAACGATCC<br>TCATCTCTTCAAAGGAAGGAAAGACTGAAGGTGTGAACTGAGTCACCCTCTAAAGGGCATCTTTCTATACCGTAACACAGTGCTTCTCAAACTATTTACCACCTCAGGAAATATTTGTCTCTCCAGGTACTTTCATAATGACCA<br>GCAGTAAAATAAAGTAGCGTAGTAGGCCTAATTATTCAGCTACTGCACAGTTCAAAAACTAAACTTAACCTTTTCAACCTCCGTGAGTTGTAAATACTCCAGCTGACCCTCCTGTGGGAGGTTTTTTACTTGGAATCCATTGCA<br>TTCTAAAGTTCTCATGGTGATGTGTCGAGATTATCACGTGACCCACCAGCCCCAGGGATCTATGTTTATATGGTATTTTGTTTCGTTTTGCCCTTTTCTGTAAATATTGACAAATGTTCTAATTATATTGTGAAATTTCTGATA<br>CTC<br>3:**CTCTTTAA**CATGGGATTGACGCAATTGTTGGTACCTTGTCCTGAAGAGCTTGCACCATGGTCTTGTGCACGTTAAGCTTTACTGTGACCTCGGGTTTGCTGGCACAAACATAACAGCTGGCATTTGGAGGGTCCAGCGCACA<br>CGGGACGAGCAGCTTTTTCCTGGGGTTTGGCTGTTTGTTTAGGAAGATCTAAAGAAAAACACAAATTATTCAAAGGTCAGAATGACCAAAGCCACTGATAACCAAGCTAGAAAAAGAAAATTGAAGATGTGTTAGATTAGAGAG<br>CACATGATGCAATTCAGTTTTGATCATGAACTGTAGAAGCAGAAGATCAAGCTCACCGTGCGACACTGTTCAAAGTCTGAGTTGAGAATCTTCAGTGCTTCCAGAACAATGAGACCGGCAATGACGGCGTTAGTGGTGGCTATA<br>GCTGGAATGATGTTGCCTGCCATGGCTAGATGAGAAAACAGCATGGGTTAAAGCCACATTAAAAATCACAAAATTATTTAGTTGAAGCATTCAATTGTTATTAATTTGTTTTAATGCCACTATAATTTGTTTTAATGCCACAA<br>TAGATTCTTACACAAGCTACAAATGTGTTTTTCTTCCACTTTTACTTAATATGAATGTATAAGTCTTGAGTGGCCAATTCTACATTGTATGTGGTGTATGTATATTCTTGTATGTACAAACTGGTCATGTTCATTTTCAAAATG<br>GTAATTATTTTCTTTCATTTA
Gateways-86A	3:**GTGAAATT**TCAGTTTGCGCTCTCTTTCTATGTAAAGCTTTTAGCTCTGATTGAAGACTAAAAGCGTGCGCCGTGGCAGAATGTTTTGAGCTTTTCGGCTTTGTTGAGTCGTATAGATATTTCGTACCAAACATGAACTGCTT<br>ACTTGTACTGAAAAGGTGCATTTGGGGTCAGCATACAAATGACAGAACAAGCTGAGTTTTAAAAACAGCTATTGAGCGTGATATTCAAGTCCAAGTTTGTCTCTTCAGCTAGAAAATGCTCAGGATTTAACAAAAGCAAACTGT<br>CTTTTGTATTAGGGATGAAAATATTTAGCCTTGTTTACTGGCATTGTTGTGAACAGGATCTTAAAGTAGAGTAGTATATCTTGTGCTGATAACAATAAGCACTAGGATATCTCCAGTTAGCTAATATTGACCATATACAAATGT<br>TTATTTTCGTTTTGAATTTCAAAGTTTATGCTTAAATACGTGCATCTATGCATATTGCACATAATATATAATCTTAAACCATTGGTCATCAGTGAGTGTCACGATCATCTGCGATCTAAAACACCATAGATAGCTGGAAAACAC<br>TCACAAGCGGTAATGGACTACAGGTTCACCATTTCATGGACTACAACTCTATACATGCACTCACGCGCACATGTTCCTGATCCTGACTGATTGCACACACAACACCGGAGGATTGTCAAGGACTGATTACACACACTATTTAAG<br>CTGCACGCACAGACACATCTGGACTTAGCATTGCTATATTGTAATGTTTCATGTTTGAGTATGTTCCTGTCTA
Gateways-86B*	3:**CTCAGCAG**GTATGTAAATACAAATACCAAAACATAATACTTCCACACAAACACACATTTAACGTTAGTAGTTTGGTTAACTGTTGATGTTTGTACCTGTtgaattactAACGTTACTACTTCTGTATGTGTGTATTGTTAAA<br>GCATGAACAGTCCTATATGTACAGTTTATAAACATGAAATACTGTTTCTCTGTTTGAATGTAATAATAGATGTCACTTACCTGTAACACTGTGTTTTCAAATATTGTATTTCCTCAGTAATAAACAGTTATAAAGCCAGACACC<br>CGTTTTCTTGGTTTATAGAGACATCTCGCGGTCCTATGCGGCAACACAACACAAAGCAGCACCTTATATTATGAACTCATTAAAATCGCCATCATCATTCAAATTTCCGTTAATATTCAAATCAGTAGGTTAAACCTATAAACG<br>TGTCATATTTAAATTAACCGCAGAATTCTATAAATATTTAGGTTATAATAATATCAAAATCAGTCAGTAATCTCAACAAACAAAAACTATCAAATGTTTTAAATACACAATTTACGAAATTATAAAACATTTATTTGTTGATAA<br>GCTTGCCAAAAAACGGGAGTCGTCGTGTGTTTATCAGTTATAATATTCAGCAGTTATATCACACCTATCTGGCATAAGATTGGCTAACACGGAGTTTCGTCACCTCCTATTG
Gateways-86C	5:**GTCCTGAC**ACATATTGAACTAAACATGAAAAGGGACCAACTATGCCCCATTTACAAGATGTAAAATAAGTCTCTGATGCCCCTAGATTGTGGATGTGAAATTTCAGCTCAAAATAGCCCCCAAATATTTTTTTTATACCTCT<br>TTGAACCCCCCCAGCCCCCAG<br>3:**GTCAGGAC**CAAAGAATCAATAGTTTATCTTGTACAATGTGCCATAAAACAGATTGCCAAAGAGTATAGACTGTTGTAAGATACAATACTCTCTATCTACCTTAGTTCAAAGATGTCTGTCCTTGTACCACTGACACTCGACC<br>TGACCAGGCAATGATCTCCTTACACAGGTTATTTTAGTCCACAGGGGCTCAGGGTTCTTCATGCCTGTAGGCTTTCCTTATGCAGTTAAGGTTTAGGTTATAAACCAAGTTTGCCCTTGTGCGATTATTATTGATGTTTTGTCC<br>TCAGGTGCTGAAGGTAAGGCAGGGATGGCAGCCATAGCAGACCCGGAAAACAATACTGACCTAGCCAAGTTTAGCAGGGACCTGGAGAAGGCCCTGCCACCCTATGCCCGT
Gateways-86D	5:**CACACACA**CACACACACACACACACACACACACACACACACACACA

	3:**TGTGTGTG**TGTTTACCAGGCCACAGTGATGCGCGGGTCCAGGTAGTTGAGTTTAGACGTGCCCAGAGCGATCACCTTGTTCTCCTCACGGTCGGTTTCCTGCAGCTGCAGCTTCTTCAGCTGATCACACAGTCTCTGCACAG<br>CCTTCTGCTTCTTCTCTACCAGCCTGACACACACACACACACACACACACACACACACACAC
Gateways-86E	5:**TACAGTAA**CAATATTAGGGCATCCTTTTAATGATTATTCGTTATTTTACACTCCTCCTGTTATTATAATCCACTCCGGCGCAGTAATAATCCATTTACCAAGAAGCGCTCGAGACCGGCGCGTGCGCATTAAGGAGAACTTT<br>TTAAAAGTCCTGATAAAAACCGCAAACCTGTTTGCTCCTGTCCTCCGCTGCCTTTTTATCTCCCTCAGCCTGCTCAGCTCTGTCCAAGGCGTTCTCCTTGTCGAGCTTGAGCATCTGCATCTTCTTCTTAATGGCATCCATGGC<br>TGCTTTTGGGGTTG<br>3:**TTACTGTA**ATGCCATGTTCATGCTGACTTACTTTTTATTTATTTAAATAGTGCAGGAAAATAGCCATACATTTACTAACCCTCATGTTTTATTCTTGTTG
Gateways-87	5:**GTCCAGGA**CCCCTGTATTCTCGGTCTAGACTTCCTAAGAAGTGCAGGCTGTAAGTTAGACTTGAAAAATGGTATGCTTTGTTTCGAAGGGGGTCCTGAGGTTGTCATGTCCACTGGAAATGACAGGGATATGTCTTTTACAG<br>GCCTTCTAGAGGCAAAATCTTCTCATGGGAAGCCTAATGGGGGGGAACATATACCACACTTGCAAAACAACAACAAAGCTGACAACAGTCCTCAAGGGGTAAAGAACTCTCCTACTTCCTTAGCCCTTGCTCCGCCTGTGGTCT<br>ATCCCAGGGTGGCCCCACAATTCCATGACATGCAAGGGCAGGGAACCAGCACTCTGATTTCCCCTCTTGGGGAGGAGACAGGGTTGGGGGATTTGAGGGAGTTAGGGTGTAAGAATTGTGAGGGCTTGGATGAGTGTCAGCA<br>3:**TCCTGGAC**AGTAGCCACCCACACTGAATGTTGTAATGTCTTCCCTCCGACAGTGAGCTCCACCTCCCCTTTACCCTTCATTGGTGCAAGTTCCCCTGTCACTGTGCGTAACAGAACAGTGGTGGGATCCAGTCTGGCATTGG<br>TTGGTACCAAATCTGGTCTCAAGACCGTTACAGTAGAGCCTGTATCCACCAATGCCACACATGGAACTCCTTCTACTAAAATTGGTACATGACAAAAATCACTAACACATGTTCGACCCACCACAACTACAGACTCTTTGGGCC<br>CATATTTCCCCTCCCCCAACCAAACATGTTTTCTCTTCGTTTGCCCCACTCCTTTGACCCCTGATGGAAAAGTCTCATGGTCGGGATGAGAGTTGAGGGTCCGCACATCCCCGCTCATGCGGACCCCAGGCCGTTTCCCTGGTT<br>CCCCGCTACATTAGGACACTGTCTGACTAAATGTCCAGTCTGACCACAACCCCAACAAACCCTTGGTCTCGTGCGAGGACGTTGCTCGGCTTGAAGTGTCACAGATCTAATTAACTCAGTTATCTCTGAGACCCAGGCTGGTCT<br>TTCACTTAACCCCTCCCCCACATCAGCAGACCTTACTGGAACAATATTGTGATGACTCTCTTCCAATTTAGGGGTTCCACAAACAATTTCTCTCTCCAAAGCCATCTCCAAAGCATCCTGTA
Gateways-88A	5:**AACACAAG**CTTTCTCACCCAAATTCTTTTTTTGTAATTAAAAAGCTAGTAAGATTGTAAATAAAATCAATGACTGATACTTAAATAGAGACTTAAAGACAAAAGTGAATGTAAATGTGTTTACAGATTCGTGGATCTCCTCA<br>AAGTCAGAGTTTCCATCTTCTTCTGCGAGAACTTCAGTTTTATCT<br>3:**CTTGTGTT**TTGAGTGTCATTTGCACACCACTGCAGTAGGCGTCTTACATTATTGACTTTAATCAGTTAATTCATCTTTAATTTAAACAACAATTCGATTACAGGAGTTTGTGGACATTGACACCTATAGTCTAAGGACCAAA<br>AGTTCAGCGACTGCATTCAACTGCTATATAGATATTCTCTATATATAACTGTATTCCATGGTCTGTTGAAATTCTTGATTCTGATTGGCTGGAGGGTGTTCAAAAAAATCGCTTAATGCAGTGGTTCCCAACCTTTGT
Gateways-88B	3:**GAGGAAAC**CTATGCGAATGCACGGAGAACATGCAAACTCCAGACAGAAACGCCAACTGACCCTGCCGAGGCTCGAACTAGCGACCTTCTTGCTAGTGAGGCGACAGCATTACCCAATGCGCCACTGCATCGCCCATCAGTTA<br>ATTACGTTGTTAATATTTTTCTTTCTCTTTGTATTTCCTTTCTATAGTCAACACCTTAATCTAACCAG
Gateways-89	5:**GCGAGGAT**TGGTGATGCAGACACACCTCCGCGCTCCCGCTGCTTTTAAAAGTGCCCCAAATGCGCGTTCACAGCTCCAG<br>3:**ATCCTCGC**CCACTCGAAGCCCTGAGGAAATAATAAAAAAAAATCACTTTCAAGAAGTGCCACAAACTTTCAAGGGCATAGAAGCGAATGCTGGGTCCACATCACATGGAGTCCACACGACACAAAACCTCACCGAACACCCAT<br>TATTATTTTCATTATTATTAATAGGGCTATATCAATTTTCATCAGTTTCACGGTATATTTTTGAAATGTTTTATTAGAACTAAACACCTTTAACTAAAACTCTAAATATGTAGGCTAAATTCATTCATTCGTTCGTTTTCTTTT<br>CGGCTTAGTATAGTAAGGGGTCGTCACCTGGGAATGAACCTTCAACTTATCCAGCATATGTTTCACGAAGAAGATGCCCTACCAG
Gateways-90B	3:**TTGTATAC**AATACCCCATGATTTACTATAGTGAAATTTGAAGTAAACAACATTCATTAAAGAATACTACAGTTTCAAAAACACTGAAGTATTTTTTAATGTGGGTTTTCATGCAACATAACATTTTTTAAAGCATGACCTTG<br>TGAAGAACTATACAATACAGGACAATTTACTGTAAAAACAACTAAAACATAGTATAACAGTATTAAATAACACTATAGTATTTCCTGCAGTATTTTTCTTTCATGCAACATTAGATGTGTTTTCAGCATGGCCTTGTGGATATA<br>GTGAGTTTTAGATTTTACTACACTAATTGTATACAATACCCCATGATTTACTATAGTAAAACCTAAAGTTTACAGCATTTATTAAAGAATACTACAGTTTTTATGCTGTTGTAACATTGATTAGCATACATTCTGTAATATACA<br>CATATCAGGCAGTGTAATATGGTTAAAAAACACGGTGGTATTTTTATTTATGTGGGTTTTCATGCAACATTAGATTTTTTTCAGCATGACCTTGTGAAAAACTTGTAACTTTTGTCATCCGCGTTGAGTTCAGTGTGAAGAAGC<br>CCGACCCATGAGAGAGAG
Gateways-90C	5:**GTAACTGG**ATTTACTCACTTTAAGAAAATCACGTTCTTATGGTGTATTTTGCAGAGATATATATATATATATTTAAACATCTGTAATTGAGGTTTTACTAAAAATGTATATATTGAGAAAATCTCAATAAAATTTGTAATATGT<br>TCAAATTCTATTTTTA<br>3:**CCAGTTAC**CATAAAAGTGAAAAGTTGACTACCTACAAAAGTGAGCGTAACCCAATATAAGGTCCCTGAAGGCACAAAACGTCATAAGACGTTAATATTAGGCTAGATTTGGGTCGTGACGTCAGGTGACCAAAATTCAATGT<br>CCAGCCGGTGTCTAGTAAGCACAACATAATTTTGACGTCCAATAACAATGTCAAATGACGTTAATATTATTTAGTGCTGGAAAGTGACCAAAAAAACAACAACGTCAAGCCAACATCTTAAACCAGCGTCATATTGATGTGTAA<br>GACTGATATTTATTTGTCAGGTATGGCGACCAAAATTCAACATCTGACAGACATCATAGTGGTAATGTCCACTCAACGTCATGCTGTAACATCATTAGAAGT
Gateways-90D	5:**TAGCCTAC**TCTCATGTCTGTGCTAACGCAAAATGAAGCGAGTGCACCAATGCCCATTCTGACCAATCACAGATGTTTCTGCTGAGCTCCTGAACACACAGGCATTCAGCTCATGCTGATATGCTCTCTCATTGACTGCA<br>3:**GTAGGCTA**TATTAACAGTGGAACTAGAATTCTGTAACTTAGAATTACCATACTGCTCATTCTGACCATTCTCATATGAAACGCCATATTGTCACAACATATTTACATTCAAAGCTATTATTGAGATATTAAAATTAAGCTTT<br>GAATGTTTGGCATCATATTTAATGACACCATTACCCTAAAAATATCATCTTATAATATATATATATAAATGTAGTTAAGATACTAAAACACTTAAAGGTCTTGGCCTTCATTTTCACTTTCAAACAGGAGCTATTTCACTGCCA<br>GAATATGCTGTTTTTTGAAAGATATCTGA

To obtain the genomic location of *Tol2* insertions, the flanking sequence reads were blasted against the latest zebrafish genome sequence database (zebrafish whole genome assembly, version 7) from the Ensembl genome browser [http://www.ensembl.org]. In some cases, the flanking sequence reads were blasted against the unfinished high-throughput genomic sequence (htgs) database or trace archive at NCBI [http://www.ncbi.nlm.gov]. Duplicated 8-bp segments of the *Tol2* integration site are highlighted in bold. Asterisks mark *Tol2* insertions with sequence reads containing extra nucleotides (small letters in blue color). Extra nucleotides are present because the DNA sequence exhibits polymorphism between the zebrafish strains Tübingen (used in the zebrafish genome project) and AB (used in our study). Two asterisks mark *Tol2* insertions without a classical 8-bp duplication of integration site.
