# Supplementary material for: Genome-wide analysis of Tol2 transposon reintegration in zebrafish
Source: BMC Genomics. 2009 Sep 8;10:418. doi: 10.1186/1471-2164-10-418 (PMC2753552; doi:10.1186/1471-2164-10-418)
Supplement: Additional file 5 — Target site analysis for 368 genomic Tol2 insertions. Table S4 shows the base composition of an 8-bp integration site in 368 genomic Tol2 insertions. For the region flanking the integration site only the first three nucleotides are shown. [file 1471-2164-10-418-S5.pdf]

Table S4 - Target site analysis for 368 genomic *Tol2* insertions[a]

Nucleotide	-3	-2	-1	1	2	3	4	5	6	7	8	+1	+2	+3
A	91	86	135	88	88	91	151	76	141	156	78	92	106	158
C	53	78	63	115	85	89	49	69	59	46	96	72	100	44
G	43	90	77	99	37	54	92	41	92	73	124	51	71	52
T	181	114	93	66	158	134	76	182	76	93	70	153	91	114
Consensus[b]	T	N	A	S	T	T	A	T	A	A	S	T	N	A

[a] The base composition of the 48-bp region including an 8-bp integration site was determined. For the region flanking the integration site only the first three nucleotides are shown. The random nucleotide frequencies at each position were considered as identical to the genome average: 30% for A, 20% for C, 20% for G and 30% for T. [b] S stands for C or G.
